# Nickel‐Catalyzed Twofold Conjunctive Coupling via Philicity‐Alternating Radical Relay

**DOI:** 10.1002/advs.202521442

**Published:** 2026-02-11

**Authors:** Ji Hwan Jeon, Da Hye Kim, Gun Ha Kim, Gyuhwan Sim, Jan‐Uwe Rohde, Byunghyuck Jung, Sangwon Seo, Sung You Hong

**Affiliations:** ^1^ Department of Chemistry Ulsan National Institute of Science and Technology (UNIST) Ulsan Republic of Korea; ^2^ Department of Physics and Chemistry Daegu Gyeongbuk National Institute of Science and Technology (DGIST) Daegu Republic of Korea

**Keywords:** 1,3‐enynes, C–C coupling, multicomponent reactions, radical philicity, twofold conjunctive coupling

## Abstract

The formation of multiple and remote C–C bonds remains highly challenging owing to difficulties in reactivity and selectivity control. Herein, we report a nickel‐catalyzed twofold conjunctive cross‐electrophile coupling that employs an alkene and a 1,3‐enyne as conjunctive platforms and alkyl and aryl halides as coupling partners. Through a philicity‐alternating radical relay followed by selective radical capture, the reaction enables the formation of three or more C–C bonds. Combined experimental and theoretical investigations provide a rationale for the sequential radical additions to the *π* systems and the selective capture of a radical intermediate by the nickel catalyst. Furthermore, a five‐component coupling showcases the sequential and controlled addition of alkyl and aryl moieties across three conjunctive units, demonstrating the synthetic versatility of the twofold conjunctive coupling.

## Graphical abstract

Herein, we disclose a nickel‐catalyzed twofold conjunctive coupling that enables a four‐component assembly involving an activated alkene, a 1,3‐enyne, and both alkyl and aryl halides. Multiple C–C bonds are selectively constructed through a philicity‐alternating radical relay and the selective radical capture by the nickel catalyst.

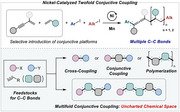

## 1 Introduction

**FIGURE 1:**
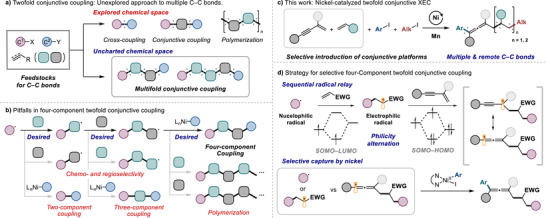
Transition‐metal catalysis for multiple C–C bond formation.

The development of synthetic methodologies enabling unconventional C–C bond formation has garnered considerable attention, as they offer valuable retrosynthetic disconnections for the construction of complex molecular architectures. Over the past few decades, transition‐metal‐catalyzed cross‐coupling [1, 2, 3, 4, 5] and conjunctive coupling [6, 7, 8, 9, 10] have emerged as powerful tools for the formation of one or two C–C bonds. Despite significant progress, methods for constructing multiple C–C bonds in a single synthetic operation via transition‐metal catalysis remain limited, largely due to challenges in controlling chemo‐ and regioselectivity. Instead, the research on the formation of multiple C–C bonds has predominantly focused on polymerization chemistry, whereby a large number of C–C bonds are constructed from monomeric building blocks [11, 12, 13]. The chemical space between cross‐coupling and polymerization, enabling the controlled assembly of a C–C backbone from multiple distinct feedstocks, remains unexplored (Figure 1a).

Transition‐metal‐catalyzed radical relay has emerged as a valuable strategy for multicomponent reactions by introducing radical species and facilitating their cascade addition to radical acceptors [14, 15, 16, 17, 18, 19, 20]. A prominent example is the extension of cross‐electrophile coupling (XEC) to three‐component transformations, allowing two carbon‐based electrophiles to be selectively added to a *π* system functioning as a conjunctive platform [21, 22, 23, 24, 25, 26, 27, 28, 29]. The chemo‐ and regioselective coupling of the three components was achieved through precise catalyst tuning and appropriate selection of *π* systems. Although a variety of *π* systems have been successfully employed in conjunctive XEC, the incorporation of multiple conjunctive units within this context has not been reported. Aside from conjunctive XEC, other examples of multicomponent radical relay have demonstrated the feasibility of sequential radical addition using two electronically differentiated alkenes for carbon–carbon or carbon–heteroatom bond formation [30, 31, 32, 33, 34, 35, 36, 37, 38, 39, 40].

Recognizing the gap between the fields of cross‐coupling and radical polymerization, we set out to explore nickel‐catalyzed twofold conjunctive coupling that employs two distinct conjunctive platforms by philicity‐alternating radical relay approach. While potentially offering an unconventional and efficient synthetic route to the formation of multiple C–C bonds, this strategy remains largely elusive due to several fundamental challenges (Figure 1b): i) During the sequential radical addition, each step must exhibit high chemo‐ and regioselectivity to ensure a controlled transformation. ii) The capture of radical intermediates by the nickel catalyst before the completion of the two stages of radical addition must be avoided to prevent the formation of undesired two‐ or three‐component coupling products. iii) Conversely, the radical intermediate generated through the two stages of radical addition must be captured rapidly by the nickel catalyst to suppress undesired radical polymerization.

Recently, we reported a conjunctive XEC reaction using 1,3‐enynes enabling the chemo‐ and regioselective construction of two C–C bonds through selective capture of an allenyl radical by a nickel catalyst [29]. To achieve selective multicomponent reactions, we asked whether our previously developed three‐component XEC could be extended to a twofold conjunctive XEC by incorporating an additional conjunctive platform while preserving the selective nature of the radical capture. Herein, we present a nickel‐catalyzed twofold conjunctive XEC reaction that successfully orchestrates two different organic halides and two distinct *π* systems to construct three different C–C bonds in a single step (Figure 1c). We demonstrate that the combination of an electronically activated alkene and a 1,3‐enyne facilitates the chemo‐ and regioselective incorporation of the two conjunctive platforms via philicity‐alternating radical relay and selective capture of an allenyl radical by the nickel catalyst (Figure 1d). Competition experiments and density functional theory (DFT) studies support the mechanistic rationale for the twofold conjunctive coupling. A five‐component coupling incorporating three molecules of conjunctive platforms illustrates the extensibility of this new approach.

## 2 Results and Discussion

### 2.1 Initial Reaction Development

To pursue this unexplored transformation, we began our investigation by adopting the concept of radical philicity to control the sequence of radical additions, which is essential for the selective incorporation of multiple components [41, 42]. While such an approach of alternating radical philicity for controlling selectivity has been utilized in radical copolymerization [11, 12, 13], its use in twofold conjunctive coupling remains elusive, presumably because of the difficulty in promoting selective metal catalysis in a system involving diverse radical intermediates. A sophisticated reaction design is thus needed to avoid undesired side reactions that may arise from uncontrolled radical additions and unselective metal catalysis. In this regard, we hypothesized that the use of an activated alkene and a 1,3‐enyne as two conjunctive units could enable the selective construction of multiple C–C bonds when incorporated into a nickel‐catalyzed XEC reaction. Each unit would selectively react with either a nucleophilic or an electrophilic radical to facilitate sequential radical additions via philicity alternation (Figure 1d). The capability of the nickel catalyst to react with a specific radical after the sequential additions is also crucial, because the reaction could otherwise lead to the formation of undesired two‐ or three‐component coupling products or to polymerization. In our previous research on the three‐component XEC using 1,3‐enynes as a single conjunctive unit, we observed that the Ni/bpy catalytic system exhibited exclusive regioselectivity for the selective capture of the propargyl/allenyl radical at the allenyl site, leading to 1,4‐alkylarylation rather than 1,2‐alkylarylation [29]. In the current four‐component twofold conjunctive coupling, we explored whether this inherent high selectivity of the nickel catalyst for capturing the propargyl/allenyl radical [43, 44] can be effectively leveraged in intermolecular competition involving multiple types of alkyl radicals [45] (Figure 1d).

**FIGURE 2:**
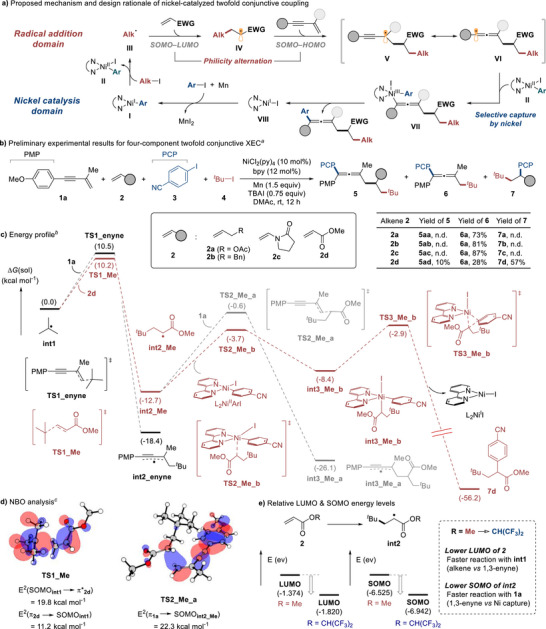
Preliminary experimental results and design rationale based on DFT calculations. ^a)^ Reaction conditions: **1a** (0.60 mmol), alkene **2** (0.60 mmol), **3** (0.40 mmol), **4** (0.60 mmol), NiCl_2_(py)_4_ (10 mol%), 2,2′‐bipyridine (12 mol%), Mn (1.5 equiv), TBAI (0.75 equiv), DMAc (2 mL), rt, N_2_, 12 h. Isolated yields. PMP, *para*‐methoxyphenyl; PCP, *para‐*cyanophenyl; rt, room temperature; n.d., not detected. ^b)^ Preliminary energy profile calculated at the uM06/6‐311+G^**^|LANL2DZ(Ni and I)/SMD(THF)//uM06/6‐31G^**^|LANL2DZ(Ni and I) level of theory. ^c)^ MOs of **TS1_Me** and **TS2_Me_a** showing SOMO_int1_–*π* ^*^_2d_ and *π*_1a_–SOMO_int2_Me_ interactions, respectively, and related orbital interaction energies.

The working hypothesis for the four‐component twofold conjunctive coupling consists of two parts, namely the radical addition domain and the nickel catalysis domain, as illustrated in Figure 2a. Activation of a tertiary alkyl iodide by L_2_Ni^I^Ar (**I**) forms the nucleophilic alkyl radical **III** and L_2_Ni^II^ArI (**II**) [21, 46]. The Giese‐type radical addition to the electron‐deficient alkene generates the radical intermediate **IV**, which has electrophilic character (philicity alternation). The secondary radical addition of **IV** to the 1,3‐enyne forms the propargyl/allenyl radical (**V**/**VI**). Selective capture of the radical at the allenyl site (**VI**) by **II** affords the Ni^III^ complex **VII**, and reductive elimination yields the twofold conjunctive coupling product. Reaction of the L_2_Ni^I^I complex **VIII** with aryl iodide followed by reduction with manganese regenerates Ni catalyst **I** [47].

With these considerations in mind, we tested a range of alkenes **2** in reactions with 1,3‐enyne **1a**, aryl iodide **3** and alkyl iodide **4**, using NiCl_2_(py)_4_ as a pre‐catalyst, 2,2′‐bipyridyl as a ligand, Mn as a reductant, tetra‐*n*‐butylammonium iodide (TBAI) as an additive, and *N*,*N*‐dimethylacetamide (DMAc) as a solvent at room temperature (Figure 2b). The three‐component reaction (3CR) product **6a** was formed almost exclusively in the presence of relatively electron‐richer alkenes (**2a**–**2c**). In sharp contrast, the use of methyl acrylate (**2d**) led to a selectivity switch, allowing for the initial addition of the *tert*‐butyl radical to the acrylate to ultimately give the ester products **5ad** and **7d** in preference over the typical 3CR product **6a**. Although the use of an electron‐deficient radicalophile enabled selective radical addition, there was still a lack of ability to control the selective participation of nickel catalysis (capture of **V**/**VI** vs **IV**), as observed by a poor selectivity toward the formation of four‐component reaction (4CR) product **5ad** (10%) over **7d** (57%).

To better understand the selectivity obtained in the preliminary experimental results, we briefly investigated the mechanistic aspects by conducting DFT calculations (Figure 2c). As anticipated, the addition of the *tert*‐butyl radical (**int1**) to acrylate **2d** showed a slightly lower activation barrier than that to 1,3‐enyne **1a** (**TS1_Me** vs **TS1_enyne**; Δ*G* ^ǂ^ = 0.3 kcal mol^−1^), justifying the experimentally observed selectivity of the initial radical addition (**6a**:**5ad**+**7d** = 1:2.4). The α‐carbonyl radical **int2_Me**, generated from the favored initial step, was then calculated to undergo an arylation via radical capture by L_2_Ni^II^ArI (**TS2_Me_b**, Δ*G* ^ǂ^ = 9.0 kcal mol^−1^) and subsequent reductive elimination (**TS3_Me_b**, Δ*G* ^ǂ^ = 5.5 kcal mol^−1^) with reasonable activation barriers. On the other hand, the desired path involving addition of **int2_Me** to 1,3‐enyne **1a** was found to traverse the corresponding transition state with higher energy (**TS2_Me_a**, Δ*G* ^ǂ^ = 12.1 kcal mol^−1^). Despite the nickel complex being present in a much lower concentration, the preferred formation of **7d** over **5ad** could be rationalized by such a difference in the transition state energies (**TS2_Me_a** vs **TS2_Me_b**) [48].

To obtain intuitive information for the improvement of selectivity, we conducted natural bond orbital (NBO) analysis (Figure 2d) for the key transition state structures of the desired reaction path [49].The addition of **int1** to acrylate **2d** (**TS1_Me**) was revealed to take place predominantly by a SOMO**_int1_**–*π* ^*^ **_2d_** interaction, with the corresponding orbital interaction energy (second‐order perturbations) surpassing the one for the *π* **_2d_**–SOMO**_int1_** interaction (E^2^ = 19.8 vs 11.2 kcal mol^−1^). Contrary to this initial addition step, the subsequent addition of **int2_Me** to 1,3‐enyne **1** (**TS2_Me_a**) was calculated to be dominated by a *π* **_1a_**–SOMO**_int2_Me_** interaction (E^2^ = 22.3 kcal mol^−1^), with no notable contribution found for a SOMO**_int2_Me_**–*π* ^*^ **_1a_** interaction. These computational outcomes suggested that the *tert*‐butyl radical (**int1**) would indeed react as a nucleophilic radical while the resulting α‐carbonyl radical **int2_Me** would behave as an electrophilic radical, which consequently led us to hypothesize that the selectivity would improve by employing more electron‐deficient alkenes. For instance, acrylates containing an electron‐withdrawing *O*‐substituent, such as the 1,1,1,3,3,3‐hexafluoro‐2‐propyl group (HFIP), have a lower‐energy LUMO that may allow for an improved reaction with the nucleophilic *tert*‐butyl radical (Figure 2e). Moreover, the participation of such alkenes may give rise to highly electrophilic radicals (**int2**) having a lower‐energy SOMO, with which a *π* **_1a_**–SOMO**_int2_** interaction would be enhanced to facilitate the desired addition path. Indeed, a dramatic shift in reactivity was experimentally observed simply by changing the acrylate substituent from methyl to HFIP under the reaction conditions shown in Figure 2b, affording the corresponding 4CR product **5** as the major species in 33% yield, without notable formation of the 3CR byproducts **6** and **7** (see Table S2 for details).

### 2.2 Reaction Optimization

**SCHEME 1:**
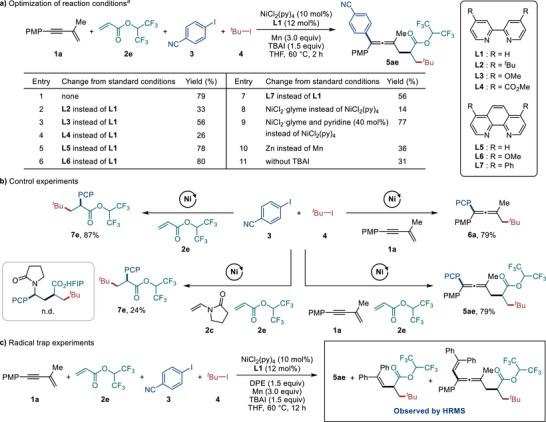
Reaction development and control experiments. ^a)^ Reaction conditions: **1a** (0.30 mmol), **2e** (0.90 mmol), **3** (0.20 mmol), **4** (0.60 mmol), NiCl_2_(py)_4_ (10 mol%), **L1** (12 mol%), Mn (3.0 equiv), TBAI (1.5 equiv), THF (1 mL), 60 °C, N_2_, 2 h. Isolated yields. DPE, 1,1‐diphenylethylene.

On the basis of these results, the reaction conditions were further optimized primarily by changing the solvent from DMAc to THF, increasing the reaction temperature and using a higher loading of alkene (see Tables S2–S6 in the Supporting Information for details). Among the alkenes tested, acrylate **2e** was found to be the optimal acrylate for the 4CR, affording product **5ae** in a yield of 79% (Scheme 1a, entry 1). Under the optimized conditions, we observed twofold conjunctive XEC with excellent chemoselectivity, as the reaction did not generate the 3CR byproduct **6a** and afforded the 3CR byproduct **7e** in only 7% yield (see Section S3 for details). The optimal precatalyst was NiCl_2_(py)_4_, while the ligands **L1**, **L5** and **L6** were equally suitable (entries 2–8). Other precatalysts, such as NiCl_2_·glyme, led to significantly reduced yields. Notably, the addition of pyridine along with NiCl_2_·glyme and **L1** restored the yield of **5ae** (entries 8 and 9) [50, 51, 52, 53, 54, 55]. Alternative reducing agents, such as Zn, resulted in decreased yields (entry 10), and a reaction without TBAI also gave a reduced yield (entry 11). In related XEC studies, salt additives have been reported to facilitate the reduction of nickel complexes by tuning the redox potential of Mn [56]. Additionally, the coordination of anions to Ni^I^X or Ni^I^Ar complexes has been proposed to stabilize them and tune their redox potentials by changing the coordination geometry of the Ni center [57].

### 2.3 Control Experiments

Control experiments using a single conjunctive platform underscore the importance of an appropriate pairing of two conjunctive *π* systems in facilitating the twofold conjunctive coupling (Scheme 1b). Reaction of only 1,3‐enyne **1a** under the standard conditions for twofold conjunctive coupling led to the formation of the 3CR product **6a** [29], whereas the reaction of only acrylate **2e** gave the 3CR product **7e** [58, 59]. Furthermore, the combined use of two electronically differentiated alkenes (**2c** and **2e**) failed to produce the desired 4CR product [30, 31, 32, 33, 34, 35, 36, 37], while affording 3CR product **7e** in 24% yield. Another combination of alkenes was tested by using vinyl acetate and **2e**, giving **7e** in 25% yield (see Section S5.1 for details). The suppressed formation of **7e**, without any other noticeable products, may suggest that the α‑carbonyl radical (**int2**) was formed and added to the other alkene (i.e., **2c** or vinyl acetate), followed by uncontrolled radical additions leading to polymerization (see Section S5.1 for details). These results contrast with the formation of the 4CR product in the reaction using both **1a** and **2e** and demonstrate that a dramatic reactivity shift toward twofold conjunctive XEC is attainable through the appropriate combination of conjunctive platforms (i.e., a 1,3‐enyne and an activated alkene). It is noteworthy that optimized conditions require no significant modification from conventional nickel‐catalyzed XEC, highlighting the potential applicability of our twofold conjunctive coupling to diverse radical‐involved difunctionalizations.

To further investigate the mechanism of the twofold conjunctive coupling, we performed radical trapping experiments using 1,1‐diphenylethylene (DPE), as illustrated in Scheme 1c. The standard twofold conjunctive coupling was conducted in the presence of DPE, and the reaction mixture was analyzed by high‐resolution mass spectrometry (HRMS). To our delight, the adducts of the α‐carbonyl radical intermediate and of the corresponding propargyl/allenyl radical intermediate were observed. The results of the radical trapping experiments provide evidence for the formation of the α‐carbonyl radical and its subsequent radical addition to the 1,3‐enyne. These experimental findings correlate well with the DFT studies, supporting the sequential radical relay mechanism.

### 2.4 Effects of Substituents in the Conjunctive Platforms on the Selectivity

**SCHEME 2:**
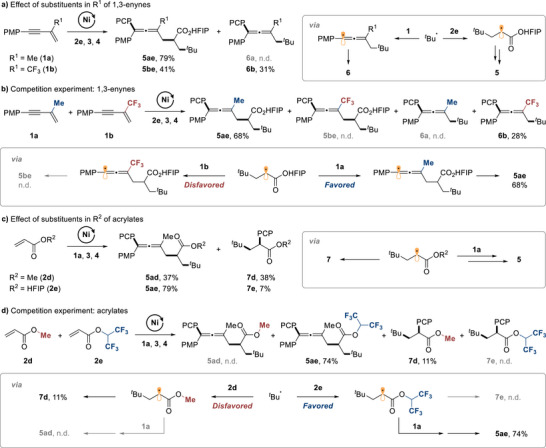
Effects of substituents in conjunctive platforms and competition experiments.

To examine the selectivity of the twofold conjunctive coupling further, we investigated reactions of conjunctive platforms with different electronic properties. When electron‐neutral 1,3‐enyne **1a** was used, the reaction afforded the desired product **5ae** in high yield, without detectable formation of **6** (Scheme 2a). However, when an electron‐withdrawing trifluoromethyl group was introduced in R^1^ position (**1b**), the reaction gave the 4CR product **5be** in a reduced yield of 41%, along with the 3CR product **6b** in 31% yield. This result indicates that the electron‐poorer 1,3‐enyne **1b** effectively competes with **2e** as a radical acceptor to produce **6b**, revealing an effect of the electronic properties of the radical acceptors on the selectivity of the radical relay. The reaction of a 1:1 mixture of **1a** and **1b** produced **5ae** and **6b** in yields of 68 and 28%, respectively, but not **5be** and **6a** (Scheme 2b). The results for the 3CR products **6** reflect the relative reactivity of the 1,3‐enynes toward the alkyl radical. Only **1b**, but not **1a**, can compete with **2e** as radical acceptor (**6b** vs **6a**), as demonstrated in Scheme 2a. However, **1a** dominated the twofold conjunctive coupling (**5ae** vs **5be**), further supporting the electrophilic character of the α‐carbonyl radical, which reacts preferentially with the electron‐richer 1,3‐enyne **1a** via a *π*–SOMO interaction.

Next, we shifted our focus to the radical capture step by the nickel catalyst. The 3CR product **7** arises as a byproduct, if the α‐carbonyl radical is captured by nickel before undergoing addition to the 1,3‐enyne (Scheme 2c). When the less electron‐poor acrylate **2d** was used, the 4CR product **5ad** was obtained in 37% yield, although **7d** was still produced in 38% yield. In contrast, the formation of 3CR product **7e** was significantly suppressed when acrylate **2e** was used, resulting in an improved yield of the 4CR product. Here, the electron‐withdrawing group enhances the reactivity of the α‐carbonyl radical toward the 1,3‐enyne, so that addition can occur prior to radical capture. A competition experiment with a 1:1 mixture of **2d** and **2e** further confirmed the superior reactivity of **2e** toward the nucleophilic *tert*‐butyl radical (Scheme 2d), leading to the preferential formation of the 4CR product **5ae** over the products derived from reaction of **2d** (**5ad** and **7d**). Notably, the rationale for the reactivity tuning of the α‐carbonyl radical was clearly demonstrated in the competition experiment. The HFIP substituent at the R^2^ position selectively suppressed the formation of the undesired 3CR product **7e**, whereas the methyl substituent exhibited the opposite effect, leading to the formation of **7d**.

### 2.5 Computational Investigation of Sequential Radical Relay and Selective Coupling

**FIGURE 3:**
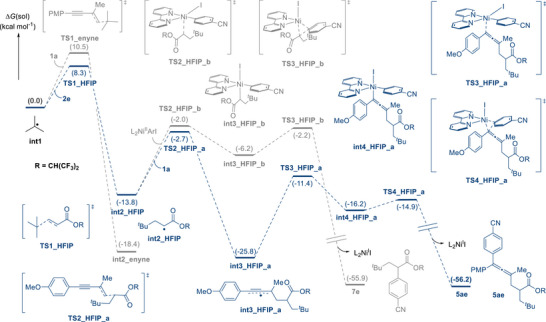
Energy profiles of the nickel‐catalyzed twofold conjunctive coupling. Calculations were performed at the uM06/6‐311+G^**^|LANL2DZ(Ni and I)/SMD(THF)//uM06/6‐31G^**^|LANL2DZ(Ni and I) level of theory.

To clarify the observed selectivity further, we conducted DFT calculations (Figure 3). The addition of the *tert*‐butyl radical (**int1**) to 1,1,1,3,3,3‐hexafluoro‐2‐propyl acrylate (**2e**) was calculated to proceed with a lower activation barrier (**TS1_HFIP**; Δ*G* ^ǂ^ = 8.3 kcal mol^−1^) in comparison with the addition to 1,3‐enyne **1a** (**TS1_enyne**; Δ*G* ^ǂ^ = 10.5 kcal mol^−1^). For the ensuing process, the undesired α‐arylation path was found to be less favorable in this case, with the α‐carbonyl radical **int2_HFIP** showing a kinetic preference for the addition to 1,3‐enyne **1a** (**TS2_HFIP_a**; Δ*G* ^ǂ^ = 11.1 kcal mol^−1^) over capture by nickel (**TS2_HFIP_b**; Δ*G* ^ǂ^ = 11.8 kcal mol^−1^). Given the relatively low concentration of the catalyst compared with that of the 1,3‐enyne substrate, the secondary radical addition via **TS2_HFIP_a** represents a feasible pathway to result in the twofold conjunctive coupling. The difference in reactivity between **2d** and **2e** in our reactions, along with the results of the intermolecular competition experiments, aligns well with the calculated differences in kinetic barriers. The remaining steps of the reaction leading to the twofold conjunctive coupling product **5ae** were computed to be highly feasible with reasonable activation energies (**TS3_HFIP_a**; Δ*G* ^ǂ^ = 14.4 kcal mol^−1^ & **TS4_HFIP_a**; Δ*G* ^ǂ^ = 1.3 kcal mol^−1^) [27].

### 2.6 Reaction Scope

**SCHEME 3:**
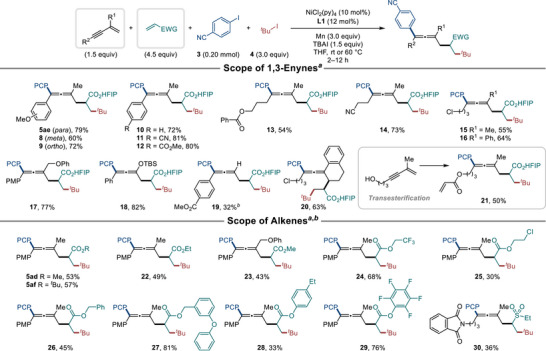
Conjunctive platform scope of nickel‐catalyzed twofold conjunctive coupling. ^a)^ Reaction conditions: 1,3‐enyne (0.30 mmol), alkene (0.90 mmol), **3** (0.20 mmol), **4** (0.60 mmol), NiCl_2_(py)_4_ (10 mol%), **L1** (12 mol%), Mn (3.0 equiv), TBAI (1.5 equiv), THF (1 mL), 60 °C, N_2_, 2–12 h. Isolated yields. ^b)^ Reaction was carried out at rt.

With the optimized conditions established, the reaction scope of the twofold conjunctive coupling was explored by varying the 1,3‐enyne and the alkene (Scheme 3). 1,3‐Enynes with aryl substituents in R^2^ position were successfully converted into 4CR products **8**–**12**, regardless of the substituents’ electronic and steric effects. 1,3‐Enynes with alkyl substituents in the position of R^2^ containing ester (**13**), cyano (**14**) and chloro (**15**, **16** and **19**) group were also well tolerated to give desired products. The reaction also worked with various substituents in the R^1^ position, including phenyl (**16**), phenoxymethyl (**17**) and silyl ether (**18**), giving good to excellent yields. A 1,3‐enyne without a substituent in the R^1^ position afforded the desired product (**19**), albeit in a lower yield. Tri‐substituted 1,3‐enyne also provided the desired product (**20**). The hydroxyl group in 1,3‐enynes underwent additional transesterification with acrylate [60, 61] as well as the desired twofold conjunctive coupling yielding product **21**. It is noteworthy that the resulting product **21**, which also contains an alkene moiety, did not undergo further radical addition, possibly due to the favored radical addition of **int1** to **2e** and lower concentration of **21**. For reactions of other alkenes, the conditions were further optimized by extending the reaction time to 12 h and lowering the temperature to room temperature (see Table S3 for details). Acrylates with methyl, *tert*‐butyl or ethyl groups were similarly compatible under the modified conditions giving **5ad**, **5af** or **22**, respectively. The reaction is tolerated with acrylates containing alkyl (**22**–**25**), benzyl (**26**, **27**) and aryl (**28**, **29**) substituents (30%–81% yield). Vinyl sulfone was also employed, giving product **30**, albeit in lower yield.

**SCHEME 4:**
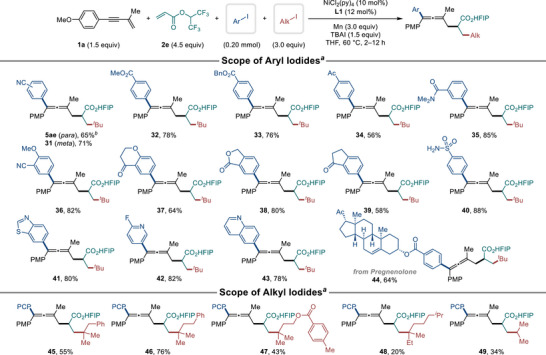
Electrophile scope of nickel‐catalyzed twofold conjunctive coupling. ^a)^ Reaction conditions: **1a** (0.30 mmol), **2e** (0.90 mmol), aryl iodide (0.20 mmol), alkyl iodide (0.60 mmol), NiCl_2_(py)_4_ (10 mol%), **L1** (12 mol%), Mn (3.0 equiv), TBAI (1.5 equiv), THF (1 mL), 60 °C, N_2_, 2–12 h. Isolated yields. ^b)^ Aryl bromide was used instead of aryl iodide.

Next, we investigated the scope of aryl and alkyl iodides with various substituents (Scheme 4). Aryl iodides with a wide range of functional groups, including cyano (**31** and **36**), ester (**32**, **33** and **38**), acyl (**34**, **37** and **39**), amide (**35**), alkoxy (**36** and **37**) and sulfonamide (**40**), were successfully converted to products. The reaction also proceeded smoothly with aryl bromide instead of aryl iodide, yielding product **5ae**. Heteroaryl iodides, including benzothiazole (**41**), pyridine (**42**) and quinoline (**43**), also afforded the desired products in good yields. To explore the application of our twofold conjunctive coupling in late‐stage functionalization of bioactive molecules, we used an aryl iodide derived from pregnenolone. Despite the presence of an alkene moiety in pregnenolone, the reaction successfully yielded the desired product **44**. Attempts to use electron‐neutral or electron‐rich aryl (pseudo)halides resulted in complex reaction mixtures, and the desired 4CR products were not detected (see Table S7 for details). Alkyl iodides with phenyl (**45**, **46**) and ester (**47**) groups were well tolerated, giving the desired products. However, reactions with a bulky saturated alkyl iodide (**48**) or a secondary alkyl iodide (**49**) resulted in reduced yields. It is noteworthy that the substrate scope of 1,3‐enynes and organic halides in this twofold conjunctive XEC closely mirrored that of our previous three‐component XEC using 1,3‐enynes. Substituents on 1,3‐enynes were well tolerated regardless of their electronic properties, whereas the reactivity of aryl iodides forming desired 4CR products was dependent on the presence of electron‐deficient aryl groups.

### 2.7 Five‐Component Coupling via Double Alkene Incorporation

**SCHEME 5:**
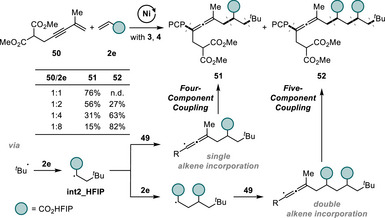
Nickel‐catalyzed five‐component coupling. Reaction conditions: **50** (0.30 mmol), **2e** (0.30–2.40 mmol), **3** (0.20 mmol), **4** (0.60 mmol), NiCl_2_(py)_4_ (10 mol%), **L1** (12 mol%), Mn (3.0 equiv), TBAI (1.5 equiv), THF (1 mL), 60 °C, N_2_, 2 h.

Despite substantial advancements in multicomponent radical relay, to the best of our knowledge, no examples of controlled incorporation of three or more conjunctive units have been reported [16, 62, 63]. So far, existing studies are restricted to either the incorporation of two conjunctive units or polymerization. To our surprise, we found that our twofold conjunctive coupling offers a controllable approach to double alkene incorporation by simply varying the molar ratio of reactants, when the less reactive 1,3‐enyne **50** was used (Scheme 5). As expected, the reaction using equimolar amounts of 1,3‐enyne **50** and acrylate **2e** produced the four‐component coupling product **51** in a yield of 76%. This result clearly shows the kinetic preference for the addition of electrophilic radical **int2_HFIP** to 1,3‐enyne **50** over addition to another molecule of **2e**. However, increasing the amount of **2e** shifted the major pathway toward five‐component coupling (5CR) involving two repeating alkenes, such that the 5CR product **52** was obtained in a yield of 82%, when an eightfold excess of **2e** was used. The notable switch in the product distribution with increasing concentration of alkene **2e** may be explained with the 1,3‐enyne's reactivity. In comparison with other 1,3‐enynes having aryl or alkyl substituents in R^2^ position (Scheme 3), 1,3‐enyne **50**, which is less conjugated and to some extent electron‐deficient, likely has a lower reactivity toward radical addition of **int2_HFIP**. This reactivity difference, combined with a large excess of **2e**, provided an opportunity for the double incorporation of the alkene to outperform the typical kinetic path. As for the four‐component coupling, the highly selective reactivity of the propargyl/allenyl radical species toward the nickel catalyst plays a critical role in terminating the radical relay process, preventing polymerization and undesired addition of the propargyl/allenyl radical to radicalophiles.

## 3 Conclusion

In summary, we have developed a nickel‐catalyzed twofold conjunctive XEC approach that enables the sequential incorporation of alkenes and 1,3‐enynes, facilitating the formation of multiple and remote C–C bonds. This method provides a platform for twofold conjunctive coupling via philicity‐alternating radical relay, which is controllable through the combination of distinct *π* systems and a nickel catalyst. Pairing an activated alkene with a 1,3‐enyne promotes the sequential radical addition via alternation of radical philicity during the two stages of addition. The selective capture of the propargyl/allenyl radical, from among various radical species, by the catalyst is crucial for the successful execution of the novel twofold conjunctive coupling, achieving high yields as well as excellent chemo‐ and regioselectivity. The mild reaction conditions aided in establishing a wide functional group tolerance and a broad scope of conjunctive systems and electrophiles. Experimental and computational studies elucidated the effects of the *π* systems on the selective sequential radical relay and on the radical capture by the nickel catalyst. The unprecedented five‐component coupling, achieved through double alkene incorporation, highlights the potential of the present approach to enable previously unattainable multicomponent transformations. We anticipate that this nickel‐catalyzed twofold conjunctive coupling opens access to a previously unexplored chemical space in radical multicomponent reactions.

## 4 Experimental Section

### 4.1 General Procedure for Nickel‐Catalyzed Twofold Conjunctive Coupling

In an N_2_‐filled glovebox, a flame‐dried 4 mL screw‐cap vial containing a magnetic stir bar was charged with NiCl_2_(py)_4_ (0.020 mmol, 10 mol%), 2,2′‐bipyridine (**L1**, 0.024 mmol, 12 mol%), Mn (0.60 mmol, 3.0 equiv), TBAI (0.30 mmol, 1.5 equiv), 1,3‐enyne (0.30 mmol, 1.5 equiv), alkene (0.90 mmol, 4.5 equiv), aryl iodide (0.20 mmol, 1.0 equiv), alkyl iodide (0.60 mmol, 3.0 equiv) and anhydrous THF (1 mL). The vial was then capped and removed from the glovebox, and the mixture was stirred at 60 °C at a stirring speed of 1500 rpm. After 2–12 h, the mixture was concentrated in vacuo. The residue was purified by flash column chromatography to afford the product.

## Conflicts of Interest

The authors declare no conflicts of interest.

## Supporting information




**Supporting File**: advs74189‐sup‐0001‐SuppMat.pdf.

## Data Availability

The data that support the findings of this study are available from the corresponding author upon reasonable request.
