# Supplementary material for: Nickel‐Catalyzed Twofold Conjunctive Coupling via Philicity‐Alternating Radical Relay
Source: Adv Sci (Weinh). 2026 Feb 11;13(20):e21442. doi: 10.1002/advs.202521442 (PMC13067834; doi:10.1002/advs.202521442)
Supplement: Supplementary file 1 — Supporting File: advs74189‐sup‐0001‐SuppMat.pdf. [file ADVS-13-e21442-s001.pdf]

Supporting Information

Nickel-Catalyzed Twofold Conjunctive Coupling via Philicity-Alternating Radical Relay

Ji Hwan Jeon[†], Da Hye Kim[†], Gun Ha Kim, Gyuhwan Sim, Jan-Uwe Rohde, Byunghyuck Jung[*], Sangwon Seo[*] & Sung You Hong[*]

J. H. Jeon[†], D. H. Kim[†], G. H. Kim, J.-U. Rohde, S. Y. Hong
Department of Chemistry, Ulsan National Institute of Science and Technology (UNIST), Ulsan 44919, Republic of Korea
E-mail: syhong@unist.ac.kr

G. Sim, B. Jung, S. Seo
Department of Physics and Chemistry, Daegu Gyeongbuk National Institute of Science and Technology (DGIST), Daegu 42988, Republic of Korea
E-mail: byunghyuck.jung@dgist.ac.kr, sangwon.seo@dgist.ac.kr

[†]Both authors contributed equally to this work
*Corresponding authors

Table of Contents

1. **General Information** S2
2. **Preparation of Substrates** S3
3. **Optimization Studies** S6
4. **Experimental Procedures and Characterization Data** S14
5. **Mechanistic Studies** S44
6. **Nickel-Catalyzed Five-Component Threefold Conjunctive Coupling** S52
7. **Computational Studies** S54
8. **References** S71
9. **NMR Spectra** S73

1. General Information

Materials

Commercially available compounds and solvents were purchased from standard suppliers (Sigma-Aldrich, Thermo Fisher Scientific, TCI, BLDpharm, or Combi-Block) and were used as received unless noted otherwise. Tetrahydrofuran (THF) was deoxygenated by sparging with N_2 and purified by passage through two packed columns of molecular sieves under an N_2 pressure (MBraun solvent purification system). Deuterated solvents (acetone-d_6 and $CDCl_3$) were purchased from Cambridge Isotope Laboratories. $NiCl_2(py)_4$ was prepared according to a previously reported procedure.[1] Abbreviations: DCE = 1,2-dichloroethane; DMAc = *N*,*N*-dimethylacetamide; glyme = 1,2-dimethoxyethane; TBAI = tetra-*n*-butylammonium iodide; TDAE = tetrakis(dimethylamino)ethylene.

Instrumentation

Thin layer chromatography (TLC) was performed on Merck TLC silica gel 60 F_{254} glass plates. TLC plates were visualized under a UV lamp (254 nm) or stained by a potassium permanganate or phosphomolybdic acid solution. Flash column chromatography was performed using Merck silica gel 60 or a silica gel cartridge on a Biotage Isolera system. ^{1}H, ^{13}C{^{1}H} and ^{19}F{^{1}H} NMR spectra were recorded on a Bruker Avance III HD spectrometer (400 MHz for ^{1}H, 100 MHz for ^{13}C{^{1}H} and 377 MHz for ^{19}F{^{1}H}) or a Bruker Avance NEO 600 spectrometer (600 MHz for ^{1}H and 150 MHz for ^{13}C{^{1}H}). ^{1}H, ^{13}C{^{1}H} and ^{19}F{^{1}H} chemical shifts are quoted in parts per million (ppm). ^{1}H chemical shifts are referenced to the residual undeuterated solvent resonance [$\delta(^1H)$ = 7.26 ppm for $CDCl_3$ and $\delta(^1H)$ = 2.05 ppm for acetone-d_6] and ^{13}C chemical shifts are referenced to the deuterated solvent resonance [$\delta(^{13}C)$ = 77.16 ppm for $CDCl_3$ and $\delta(^{13}C)$ = 29.84 ppm for acetone-d_6]. Multiplicities are reported as follows: singlet (s), doublet (d), triplet (t), quartet (q), quintet (quint), septet (sept), multiplet (m). Coupling constants (*J*) are given in Hz. High-resolution mass spectrometry (HRMS) data were obtained on a Q Exactive Plus Hybrid Quadrupole-Orbitrap mass spectrometer from Thermo Fisher Scientific or an AccuTOF DART 4G mass spectrometer from JEOL.

2. Preparation of Substrates

The following substrates were prepared according to previously reported procedures. The spectroscopic NMR data matched those in the literature; **1a**,[2] **1b**,[3] **S8**,[4] **S9**,[5] **S10**,[2] **S11**,[2] **S12**,[2] **S13**,[6] **S15**,[7] **S16**,[8] **S17**,[9] **S18**,[10] **S19**,[6] **S21**,[2] **S30**,[11] **S44**,[12] **S45**,[2] **S46**,[2] **S47**,[2] and **S48**[2].

Table S1. List of Substrates

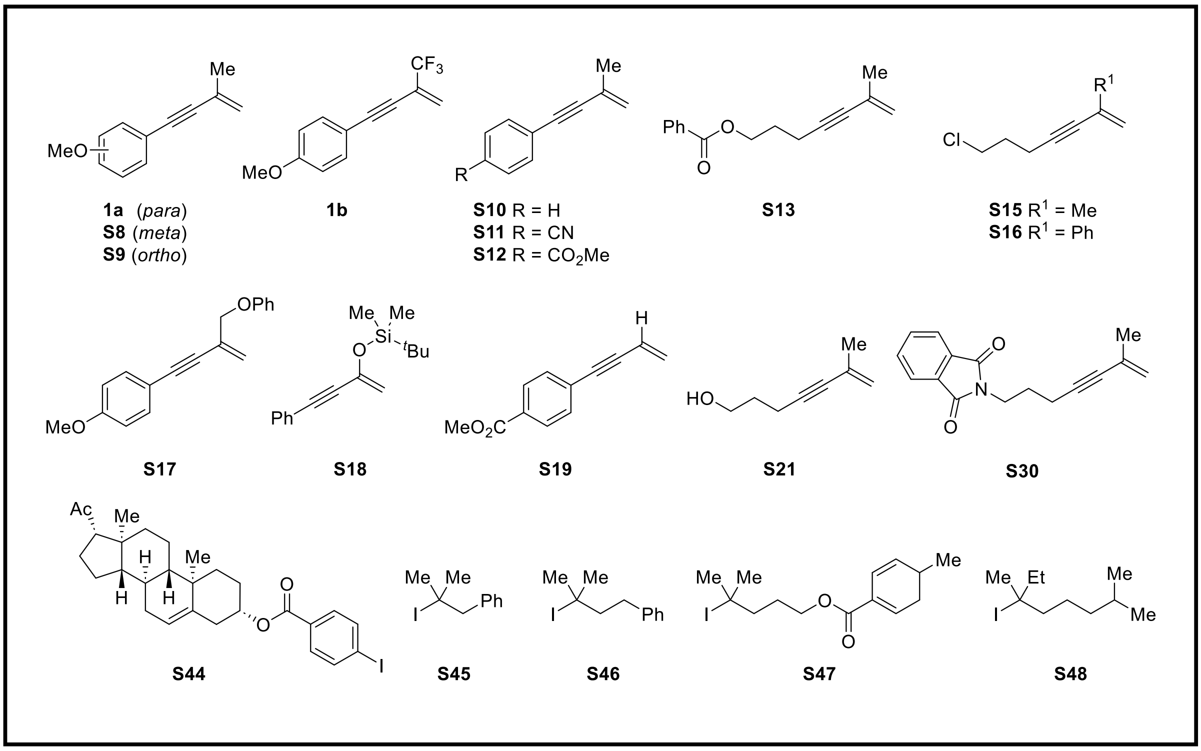

Scheme S1. Synthesis of 1,3-Enynes via Cross-Coupling

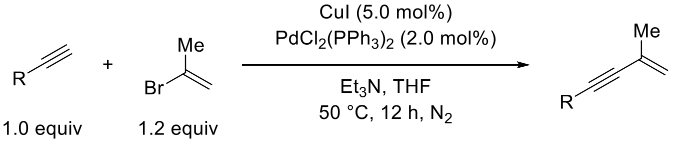

General Procedure I. A Schlenk flask containing a magnetic stir bar was charged with CuI (5.0 mol%) and $PdCl_2(PPh_3)_2$ (2.0 mol%). The flask was sealed, evacuated and backfilled with N_2 three times. THF (0.5 M) and Et_3N (0.5 M) were added to the reaction flask. Then alkyne (1.0 equiv) and 2-bromopropene (1.2 equiv) were added to the reaction mixture. After stirring at 50 °C for 12 h, the mixture was diluted with sat. aq. NH_4Cl and extracted with ethyl acetate. The organic layer was washed with brine, dried over anhydrous Na_2SO_4, filtered and then concentrated *in vacuo*. The residue was purified by flash column chromatography.

6-Methylhept-6-en-4-ynenitrile (S14)

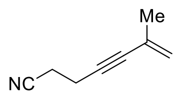

Method **General Procedure I**; 4-Pentynenitrile (237 mg, 3.00 mmol, 1.0 equiv) and 2-bromopropene (436 mg, 3.60 mmol, 1.2 equiv) were used. Purification by flash column chromatography (ethyl acetate/hexanes, 10%) gave the title compound as a pale-yellow oil (118 mg, 33%).
^{1}H NMR (400 MHz, $CDCl_3$) δ 5.25 (s, 1H), 5.20 (quint, *J* = 1.8 Hz, 1H), 2.70–2.62 (m, 2H), 2.61–2.52 (m, 2H), 1.86 (t, *J* = 1.2 Hz, 2H).
^{13}C{^{1}H} NMR (100 MHz, $CDCl_3$) δ 126.4, 122.1, 118.4, 84.4, 84.2, 23.5, 17.7, 16.7.
HRMS (ESI): *m/z* $[M+H]^+$ calcd for $C_8H_{10}N^+$, 120.0808; found, 120.0807.

Dimethyl 2-(4-methylpent-4-en-2-yn-1-yl)malonate (50)

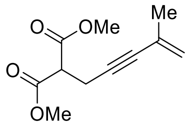

Method **General Procedure I**; 1,3-Dimethyl 2-(2-propyn-1-yl)propanedioate (510 mg, 3.00 mmol, 1.0 equiv) and 2-bromopropene (436 mg, 3.60 mmol, 1.2 equiv) were used. Purification by flash column chromatography (ethyl acetate/hexanes, 10%) gave the title compound as a colorless oil (599 mg, 95%).
^{1}H NMR (400 MHz, $CDCl_3$) δ 5.21 (s, 1H), 5.16 (quint, *J* = 1.7 Hz, 1H), 3.80–3.73 (m, 6H), 3.65–3.56 (m, 1H), 2.90 (d, *J* = 7.8 Hz, 2H), 1.83 (t, *J* = 1.4 Hz, 3H).
^{13}C{^{1}H} NMR (100 MHz, $CDCl_3$) δ 168.3, 126.5, 121.3, 84.2, 83.7, 52.6, 51.0, 23.4, 19.2.
HRMS (ESI): *m/z* $[M+Na]^+$ calcd for $C_{11}H_{14}NaO_4^+$, 233.0784; found, 233.0783.

Scheme S2. Synthesis of 4-(5-Chloropent-1-yn-1-yl)-1,2-dihydronaphthalene (S20)

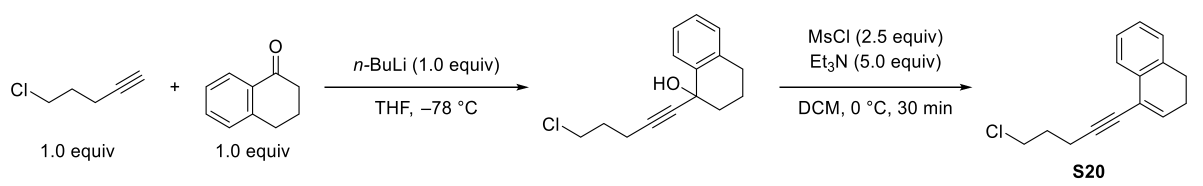

(Step i) A round bottom flask containing a magnetic stir bar was charged with 5-chloro-1-pentyne (308 mg, 3.00 mmol, 1.0 equiv) and anhydrous THF (0.5 M). The solution was cooled to -78 °C and *n*-BuLi (1.6 M in hexanes, 1.0 equiv) was added at –78 °C. After stirring at rt for 20 min, the solution was cooled to –78 °C and 1-tetralone (439 mg, 3.00 mmol, 1.0 equiv) was added dropwise to the reaction mixture. After completion of the reaction, the reaction was quenched with sat. aq. NH_4Cl and the residue was extracted with ethyl acetate. The organic layer was washed with brine, dried over anhydrous Na_2SO_4, filtered and then concentrated *in vacuo* to afford the crude, corresponding propargyl alcohol. The residue was directly used without further purification.

(Step ii) The residue was dissolved in dry DCM (0.5 M) and the mixture was cooled to 0 °C. Then Et_3N (5.0 equiv) and methanesulfonyl chloride (2.5 equiv) were added in the order. After stirring at 0 °C for 30 min, the reaction was quenched with sat. aq. NH_4Cl and the residue was extracted with ethyl acetate. The organic layer was washed with brine, dried over anhydrous Na_2SO_4, filtered and then concentrated *in vacuo*. The residue was purified by flash column chromatography (ethyl acetate/hexanes, 5%) to give the title compound as a colorless oil (486 mg, 70%).

^{1}H NMR (400 MHz, $CDCl_3$) δ 7.55 (dd, J = 7.5, 1.5 Hz, 1H), 7.27–7.21 (m, 1H), 7.18 (td, J = 7.5, 1.5 Hz, 1H), 7.13–7.08 (m, 1H), 6.40 (t, J = 4.8 Hz, 1H), 3.73 (t, J = 6.5 Hz, 2H), 2.80 (t, J = 8.1 Hz, 2H), 2.68–2.59 (m, 1H), 2.43–2.32 (m, 1H), 2.08 (quint, J = 6.5 Hz, 2H).

^{13}C{^{1}H} NMR (100 MHz, $CDCl_3$) δ 135.3, 134.7, 133.1, 127.7, 127.5, 126.7, 125.0, 121.9, 89.0, 79.5, 43.9, 31.7, 27.4, 23.7, 17.0.

HRMS (ESI): *m/z* $[M+H]^+$ calcd for $C_{15}H_{16}Cl^+$, 231.0935; found, 231.0937.

3. Optimization Studies

Table S2. Preliminary Alkene Screening[a]

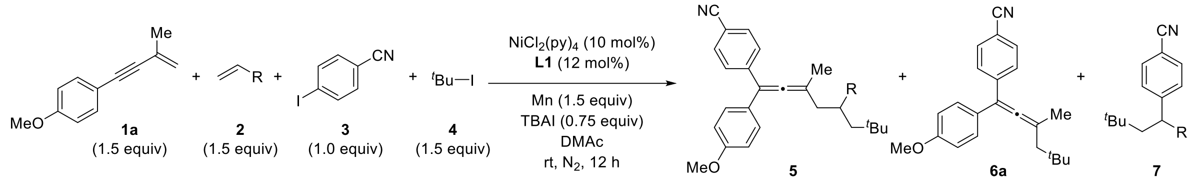

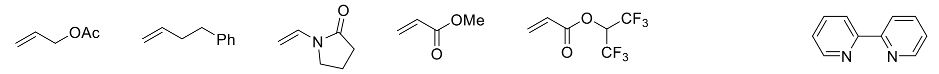

2a **2b** **2c** **2d** **2e** **L1**

Entry	**2**	Yield of **5** (%)	Yield of **6a** (%)	Yield of **7** (%)
1	**2a**	**5aa**, n.d.	**6a**, 73	**7a**, n.d.
2	**2b**	**5ab**, n.d.	**6a**, 81	**7b**, n.d.
3	**2c**	**5ac**, n.d.	**6a**, 87	**7c**, n.d.
4	**2d**	**5ad**, 10	**6a**, 28	**7d**, 57
5	**2e**	**5ae**, 33	**6a**, 5	**7e**, n.d.

[a]Reaction conditions: 1,3-enyne **1a** (0.60 mmol, 1.5 equiv), alkene **2** (0.60 mmol, 1.5 equiv), aryl iodide **3** (0.40 mmol, 1.0 equiv), alkyl iodide **4** (0.60 mmol, 1.5 equiv), $NiCl_2(py)_4$ (0.040 mmol, 10 mol%), **L1** (0.048 mmol, 12 mol%), Mn (0.60 mmol, 1.5 equiv), TBAI (0.30 mmol, 0.75 equiv), DMAc (2 mL), rt, N_2, 12 h. Isolated yields are given.

Experimental Procedure for Table S2: In an N_2-filled glovebox, a flame-dried 4 mL screw-cap vial containing a magnetic stir bar was charged with $NiCl_2(py)_4$ (18 mg, 0.040 mmol, 10 mol%), 2,2′-bipyridine (**L1**, 7.5 mg, 0.048 mmol, 12 mol%), Mn (33 mg, 0.60 mmol, 1.5 equiv), TBAI (111 mg, 0.30 mmol, 0.75 equiv), 1-methoxy-4-(3-methylbut-3-en-1-yn-1-yl)benzene (**1a**, 103 mg, 0.60 mmol, 1.5 equiv), alkene (**2**, 0.60 mmol, 1.5 equiv), 4-iodobenzonitrile (**3**, 91.6 mg, 0.40 mmol, 1.0 equiv), 2-iodo-2-methylpropane (**4**, 110 mg, 0.60 mmol, 1.5 equiv) and anhydrous DMAc (2 mL). The vial was then capped and removed from the glovebox, and the mixture was stirred at rt at a stirring speed of 1500 rpm for 12 h. The mixture was diluted with Et_2O (30 mL), washed with brine (30 mL × 3), dried over anhydrous Na_2SO_4, filtered and then concentrated *in vacuo*. The residue was purified by flash column chromatography to afford the corresponding product.

The reactions using **2a**–**2c** afforded 1,3-enyne dicarbofunctionalization product **6a** in a yield of 73–87% without any sign of alkene dicarbofunctionalization or four-component twofold conjunctive coupling giving **5aa**–**5ac** or **7a**–**7c**. However, the reaction using acrylate **2d** afforded the four-component reaction product **5ad** in 10% yield, along with formation of three-component reaction products **6a** and **7d**.
Upon changing the acrylate substituent from methyl (**7d**) to 1,1,1,3,3,3-hexafluoroisopropyl (**7e**), a notable formation of four-component reaction product **5ae** was observed with negligible formation of **6a** and no detectable formation of **7e**.

Methyl 6-(4-cyanophenyl)-6-(4-methoxyphenyl)-4-methyl-2-neopentylhexa-4,5-dienoate (5ad)

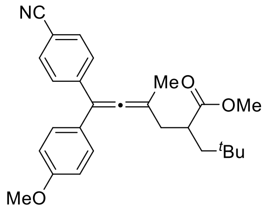

The product was prepared by **Experimental Procedure for Table S2** with methyl acrylate (**2d**, 52 mg, 0.60 mmol, 1.5 equiv). Flash column chromatography (DCM/hexanes, 10–50%) afforded the title compound as a pale-yellow oil (16.0 mg, 10%).

^{1}H NMR (400 MHz, acetone-d_6) δ 7.79–7.71 (m, 2H), 7.53–7.44 (m, 2H), 7.23 (dd, J = 8.9, 5.3 Hz, 2H), 6.96 (dd, J = 8.9, 0.9 Hz, 2H), 3.82 (s, 3H), 3.44–3.38 (m, 3H), 2.76–2.66 (m, 1H), 2.56–2.46 (m, 1H), 2.35–2.25 (m, 1H), 1.91 (s, 3H), 1.73–1.58 (m, 1H), 1.37–1.30 (m, 1H), 0.80 (s, 4.9H), 0.78 (s, 4.1H).

^{13}C{^{1}H} NMR (100 MHz, acetone-d_6) δ 204.95, 204.85, 177.04, 177.00, 160.24, 144.08, 143.96, 133.00, 132.99, 130.54, 130.49, 129.75, 129.69, 129.36, 129.27, 119.42, 114.85, 111.14, 111.10, 109.12, 109.00, 102.47, 102.33, 55.63, 51.76, 51.69, 46.92, 46.76, 40.85, 40.06, 40.02, 31.18, 31.13, 29.60, 29.58, 18.88, 18.80.

HRMS (ESI): m/z [M+H]$^+$ calcd for $C_{27}H_{32}NO_3^+$, 418.2377; found, 418.2380.

4-(1-(4-Methoxyphenyl)-3,5,5-trimethylhexa-1,2-dien-1-yl)benzonitrile (6a)

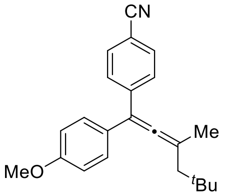

The product was prepared by **Experimental Procedure for Table S2** with methyl acrylate (**2d**, 52 mg, 0.60 mmol, 1.5 equiv). Flash column chromatography (DCM/hexanes, 10–50%) afforded the title compound as a pale-yellow oil (36.7 mg, 28%).

^{1}H NMR (400 MHz, $CDCl_3$) δ 7.63–7.56 (m, 2H), 7.46–7.39 (m, 2H), 7.23–7.18 (m, 2H), 6.93–6.87 (m, 2H), 3.83 (s, 3H), 2.10 (s, 2H), 1.93 (s, 3H), 0.91 (s, 9H). The spectroscopic NMR data matched those in the literature.[2]

^{1}H NMR (400 MHz, acetone-d_6) δ 7.79–7.71 (m, 2H), 7.54–7.46 (m, 2H), 7.26–7.21 (m, 2H), 6.99–6.94 (m, 2H), 3.82 (s, 3H), 2.20–2.10 (m, 2H), 1.95 (s, 3H), 0.91 (s, 9H).

4-(1-(4-Methoxyphenyl)-3-methyl-4-(1-methylcyclohexyl)buta-1,2-dien-1-yl)benzonitrile (7d)

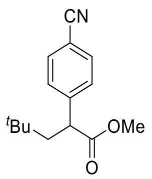

The product was prepared by **Experimental Procedure for Table S2** with methyl acrylate (**2d**, 52 mg, 0.60 mmol, 1.5 equiv). Flash column chromatography (DCM/hexanes, 10–50%) afforded the title compound as a pale-yellow oil (56.1 mg, 57%).

^{1}H NMR (400 MHz, $CDCl_3$) δ 7.59 (d, J = 8.1 Hz, 2H), 7.43 (d, J = 8.1 Hz, 2H), 3.70 (dd, J = 9.0, 4.1 Hz, 1H), 3.64 (d, J = 0.9 Hz, 3H), 2.28 (dd, J = 14.0, 9.0 Hz, 1H), 1.55 (dd, J = 14.0, 4.1 Hz, 1H), 0.88 (s, 9H). The spectroscopic NMR data matched those in the literature.[13]

^{1}H NMR (400 MHz, acetone-d_6) δ 7.78–7.69 (m, 2H), 7.64–7.54 (m, 2H), 3.84 (dd, J = 8.8, 4.3 Hz, 1H), 3.63 (s, 3H), 2.28 (dd, J = 14.0, 8.8 Hz, 1H), 1.60 (dd, J = 14.0, 4.3 Hz, 1H), 0.90 (s, 9H).

1,1,1,3,3,3-Hexafluoropropan-2-yl 6-(4-cyanophenyl)-6-(4-methoxyphenyl)-4-methyl-2-neopentylhexa-4,5-dienoate (5ae)

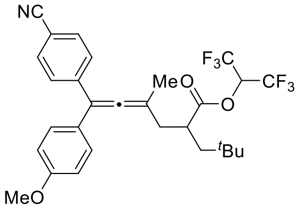

The product was prepared by **Experimental Procedure for Table S2** with 1,1,1,3,3,3-hexafluoroisopropyl acrylate (**2e**, 133 mg, 0.60 mmol, 1.5 equiv). Flash column chromatography (DCM/hexanes, 10–50%) afforded the title compound as a pale-yellow oil (73.1 mg, 33%).

^{1}H NMR (400 MHz, acetone-d_6) δ 7.79–7.73 (m, 2H), 7.53–7.46 (m, 2H), 7.28–7.20 (m, 1H), 7.01–6.94 (m, 1H), 6.43–6.15 (m, 1H), 3.82 (d, J = 2.6 Hz, 3H), 2.96–2.86 (m, 1H), 2.63–2.54 (m, 1H), 2.50–2.37 (m, 1H), 1.95 (d, J = 1.5 Hz, 3H), 1.79–1.68 (m, 1H), 1.50–1.40 (m, 1H), 0.78 (s, 4.5H), 0.76 (s, 4.5H).

^{13}C{^{1}H} NMR (100 MHz, acetone-d_6) δ 205.46, 205.22, 173.87, 173.79, 160.40, 160.38, 143.85, 143.56, 133.07, 133.04, 130.59, 130.49, 129.85, 129.73, 129.19, 128.95, 121.77 (q, J = 281.6 Hz), 119.40, 119.37, 114.96, 114.89, 111.41, 111.30, 109.57, 109.37, 101.62, 101.41, 67.26 (sept, J = 34.0 Hz), 55.66, 55.65, 45.24, 45.09, 40.33, 40.25, 39.54, 39.52, 31.11, 31.03, 29.41, 29.38, 18.72, 18.59.

^{19}F{^{1}H} NMR (377 MHz, acetone-d_6) δ -73.58 – -73.70 (m).

HRMS (ESI): *m/z* $[M+H]^+$ calcd for $C_{29}H_{30}F_6NO_3^+$, 554.2124; found, 554.2121.

Table S3. Preliminary Optimization[a]

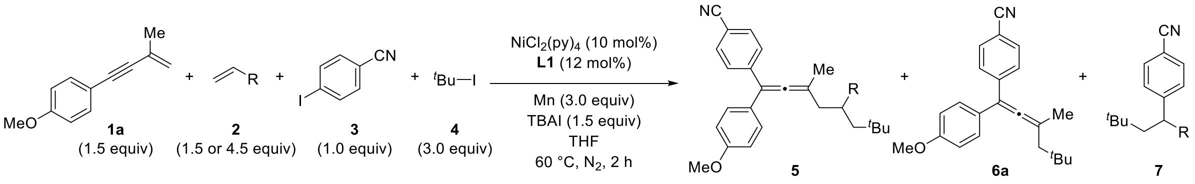

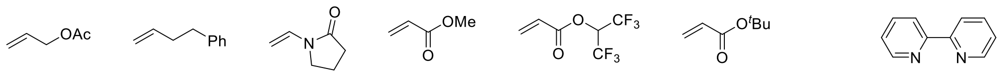

2a 2b 2c 2d 2e 2f L1

Entry	**2**	Yield of **5** (%)	Yield of **6a** (%)	Yield of **7** (%)
1[b]	**2d**	**5ad**, 37	**6a**, 22	**7d**, 34
2[b]	**2e**	**5ad**, 61	**6a**, n.d.	**7e**, 15
3[b]	**2f**	**5ad**, 34	**6a**, 31	**7f**, 28
4	**2a**	**5aa**, n.d.	**6a**, 67	**7a**, n.d.
5	**2b**	**5ab**, n.d.	**6a**, 80	**7b**, n.d.
6	**2c**	**5ac**, n.d.	**6a**, 68	**7c**, n.d.
7	**2d**	**5ad**, 59	**6a**, n.d.	**7d**, 37
8	**2e**	**5ae**, 68	**6a**, 14	**7e**, 5
9	**2f**	**5af**, 43	**6a**, 28	**7f**, 24
10[c]	**2d**	**5ad**, 37	**6a**, 15	**7d**, 38
11[c]	**2e**	**5ae**, 79 (79[d])	**6a**, n.d.	**7e**, 7
12[c]	**2f**	**5af**, 54	**6a**, 13	**7f**, 24
13[b,c]	**2d**	**5ad**, 53[d]	-	-
14[b,c]	**2e**	**5ae**, 73[d]	-	-
15[b,c]	**2f**	**5af**, 57[d]	-	-

[a]Reaction conditions: 1,3-enyne **1a** (0.30 mmol, 1.5 equiv), alkene **2** (0.3 mmol, 1.5 equiv), aryl iodide **3** (0.20 mmol, 1.0 equiv), alkyl iodide **4** (0.60 mmol, 3.0 equiv), $NiCl_2(py)_4$ (0.020 mmol, 10 mol%), **L1** (0.024 mmol, 12 mol%), Mn (0.60 mmol, 3.0 equiv), TBAI (0.30 mmol, 1.5 equiv), THF (1 mL), 60 °C, N_2, 2 h. Yields were determined by ^1H NMR spectroscopy using dibromomethane as an internal standard. [b]Reacion run at rt for 12 h. [c]Alkene **2** (0.90 mmol, 4.5 equiv) was used. [d]Isolated yield.

Table S4. Ni Precatalyst Screening[a]

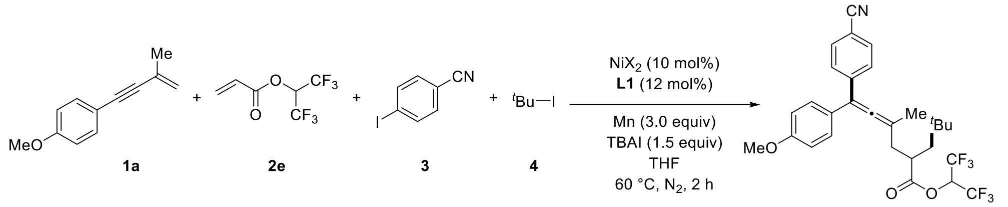

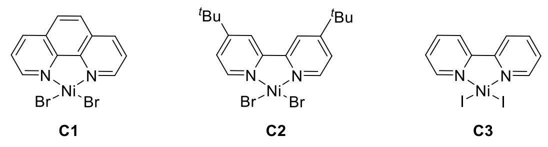

Entry	Ni precatalyst	Yield (%)
1	$NiCl_2$·glyme	14
2	$NiBr_2$·glyme	13
3	NiI_2	n.d.
4	$Ni(OTf)_2$	14
5	**C1** without **L1**	18
6	**C2** without **L1**	17
7	**C3** without **L1**	24
8[b]	$NiCl_2$·glyme and py	77

[a]Reaction conditions: 1,3-enyne **1a** (0.30 mmol, 1.5 equiv), acrylate **2e** (0.90 mmol, 4.5 equiv), aryl iodide **3** (0.20 mmol, 1.0 equiv), alkyl iodide **4** (0.60 mmol, 3.0 equiv), Ni precatalyst (0.020 mmol, 10 mol%), **L1** (0.024 mmol, 12 mol%), Mn (0.60 mmol, 3.0 equiv), TBAI (0.30 mmol, 1.5 equiv), THF (1 mL), 60 °C, N_2, 2 h. Isolated yields are given. [b]py (40 mol%) was added.

Table S5. Ligand Screening[a]

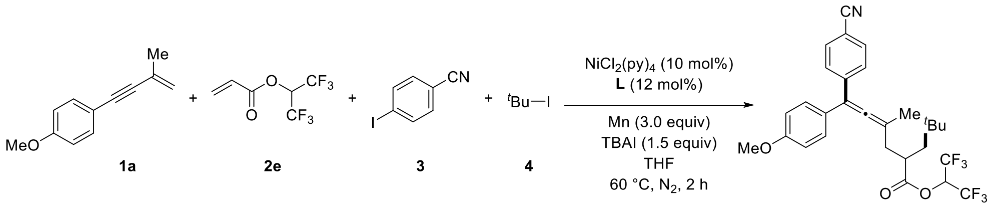

L

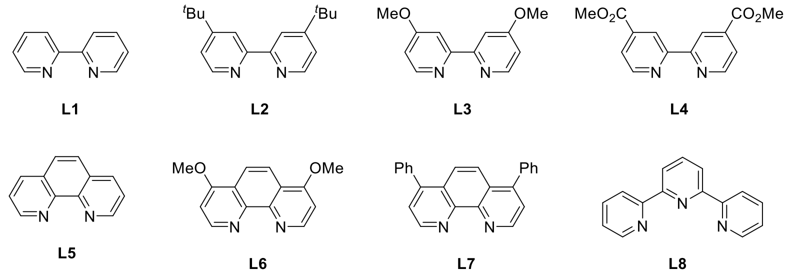

Entry	L	Yield (%)
1	**L1**	79
2	**L2**	33
3	**L3**	56
4	**L4**	26
5	**L5**	78
6	**L6**	80
7	**L7**	56
8	**L8**	n.d.

[a]Reaction conditions: 1,3-enyne **1a** (0.30 mmol, 1.5 equiv), acrylate **2e** (0.90 mmol, 4.5 equiv), aryl iodide **3** (0.20 mmol, 1.0 equiv), alkyl iodide **4** (0.60 mmol, 3.0 equiv), $NiCl_2(py)_4$ (0.020 mmol, 10 mol%), **L** (0.024 mmol, 12 mol%), Mn (0.60 mmol, 3.0 equiv), TBAI (0.30 mmol, 1.5 equiv), THF (1 mL), 60 °C, N_2, 2 h. Isolated yields are given.

Table S6. Variation from Standard Conditions: Temperature, Solvent Concentration, Reductant, Additive and Stoichiometry[a]

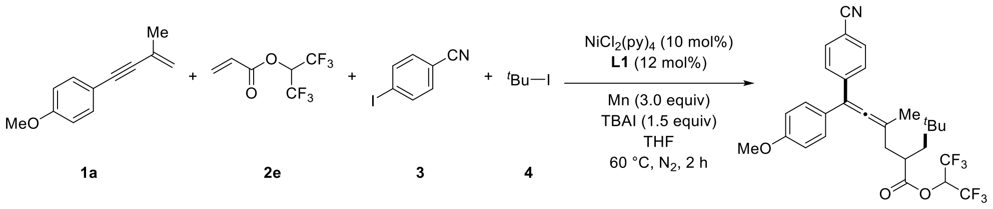

Entry	Variations from the standard conditions	Yield (%)
1	none	79
2	40 °C	n.d.
3	50 °C	66
4	THF (0.4 M)	48
5	THF (0.1 M)	46
6	1,2-dimethoxyethane instead of THF	43
7	DCE instead of THF	10
8	Zn instead of Mn	36
9	TDAE instead of Mn	n.d.
10	TBAI (3.0 equiv), THF (0.1 M)	60
11	acrylate **2e** (3.0 equiv)	52
12	without $NiCl_2(py)_4$	n.d.
13	without **L1**	n.d.
14	without Mn	n.d.
15	without TBAI	31

[a]Reaction conditions: 1,3-enyne **1a** (0.30 mmol, 1.5 equiv), acrylate **2e** (0.90 mmol, 4.5 equiv), aryl iodide **3** (0.20 mmol, 1.0 equiv), alkyl iodide **4** (0.60 mmol, 3.0 equiv), $NiCl_2(py)_4$ (0.020 mmol, 10 mol%), **L1** (0.024 mmol, 12 mol%), Mn (0.60 mmol, 3.0 equiv), TBAI (0.30 mmol, 1.5 equiv), THF (1 mL), 60 °C, N_2, 2 h. Isolated yields are given.

Table S7. Attempted Substrates: Aryl (Pseudo)halides and Vinyl Bromides[a]

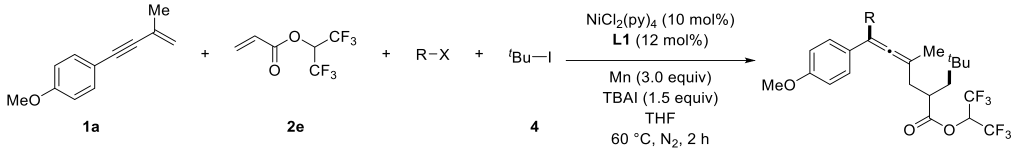

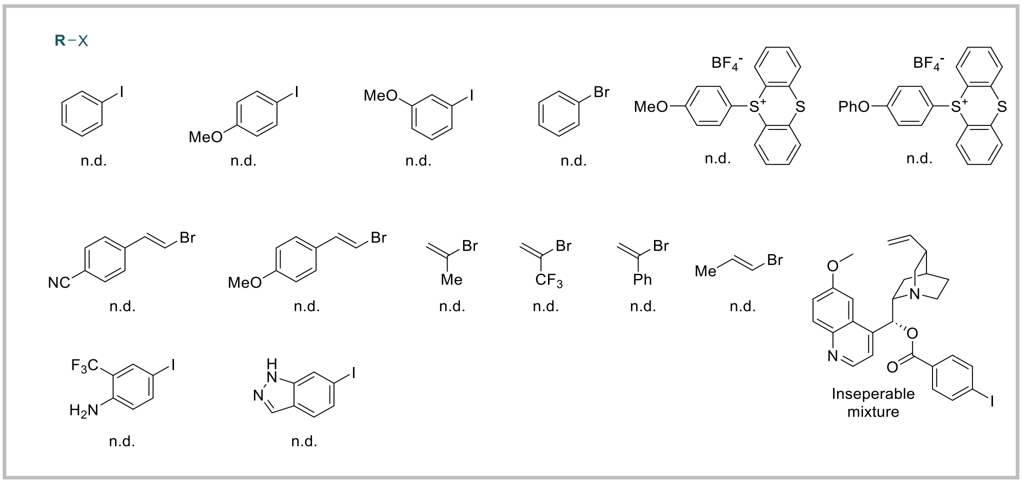

[a]Reaction conditions: 1,3-enyne **1a** (0.30 mmol, 1.5 equiv), acrylate **2e** (0.90 mmol, 4.5 equiv), R–X (0.20 mmol, 1.0 equiv), alkyl iodide **4** (0.60 mmol, 3.0 equiv), $NiCl_2(py)_4$ (0.020 mmol, 10 mol%), **L1** (0.024 mmol, 12 mol%), Mn (0.60 mmol, 3.0 equiv), TBAI (0.30 mmol, 1.5 equiv), THF (1 mL), 60 °C, N_2, 2 h.

4. Experimental Procedures and Characterization Data

Scheme S3. Nickel-Catalyzed Four-Component Twofold Conjunctive Coupling (General Procedure II)

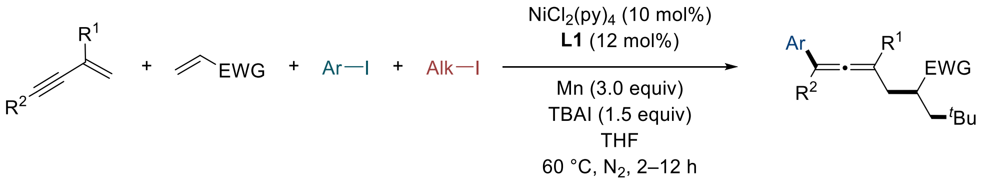

General procedure II: In an N_2-filled glovebox, a flame-dried 4 mL screw-cap vial containing a magnetic stir bar was charged with $NiCl_2(py)_4$ (8.9 mg, 0.020 mmol, 10 mol%), 2,2′-bipyridine (**L1**, 3.8 mg, 0.024 mmol, 12 mol%), Mn (33 mg, 0.60 mmol, 3.0 equiv), TBAI (111 mg, 0.30 mmol, 1.5 equiv), 1,3-enyne (0.30 mmol, 1.5 equiv), alkene (0.90 mmol, 4.5 equiv), aryl iodide (0.20 mmol, 1.0 equiv), alkyl iodide (0.60 mmol, 3.0 equiv) and anhydrous THF (1 mL). The vial was then capped and removed from the glovebox, and the mixture was stirred at 60 °C at a stirring speed of 1500 rpm. After 2–12 h, the mixture was concentrated *in vacuo*. The residue was purified by flash column chromatography to afford the product.

Scheme S4. Nickel-Catalyzed Four-Component Twofold Conjunctive Coupling with Various Alkenes (General Procedure III)

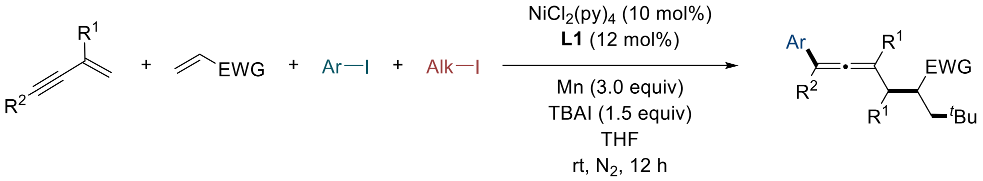

General procedure III: In an N_2-filled glovebox, a flame-dried 4 mL screw-cap vial containing a magnetic stir bar was charged with $NiCl_2(py)_4$ (8.9 mg, 0.020 mmol, 10 mol%), 2,2′-bipyridine (**L1**, 3.8 mg, 0.024 mmol, 12 mol%), Mn (33 mg, 0.60 mmol, 3.0 equiv), TBAI (111 mg, 0.30 mmol, 1.5 equiv), 1,3-enyne (0.30 mmol, 1.5 equiv), alkene (0.90 mmol, 4.5 equiv), aryl iodide (0.20 mmol, 1.0 equiv), alkyl iodide (0.60 mmol, 3.0 equiv) and anhydrous THF (1 mL). The vial was then capped and removed from the glovebox, and the mixture was stirred at rt at a stirring speed of 1500 rpm. After 12 h, the mixture was concentrated *in vacuo*. The residue was purified by flash column chromatography to afford the product.

Characterization Data

1,1,1,3,3,3-Hexafluoropropan-2-yl 6-(4-cyanophenyl)-6-(4-methoxyphenyl)-4-methyl-2-neopentylhexa-4,5-dienoate (5ae)

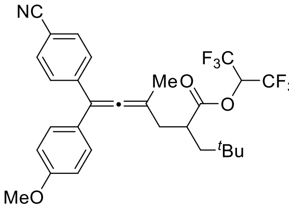

The product was prepared by **General procedure II** with 1-methoxy-4-(3-methylbut-3-en-1-yn-1-yl)benzene (**1a**, 51.7 mg, 0.30 mmol, 1.5 equiv), 1,1,1,3,3,3-hexafluoroisopropyl acrylate (**2e**, 200 mg, 0.90 mmol, 4.5 equiv), 4-iodobenzonitrile (**3**, 45.8 mg, 0.20 mmol, 1.0 equiv) and 2-iodo-2-methylpropane (**4**, 110 mg, 0.60 mmol, 3.0 equiv). The reaction time was 2 h. Flash column chromatography (DCM/hexanes, 10–50%) afforded the title compound as a pale-yellow oil (87.6 mg, 79%).

1,1,1,3,3,3-Hexafluoropropan-2-yl 6-(4-cyanophenyl)-6-(3-methoxyphenyl)-4-methyl-2-neopentylhexa-4,5-dienoate (8)

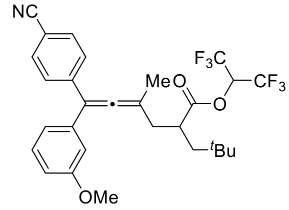

The product was prepared by **General procedure II** with 1-methoxy-3-(3-methylbut-3-en-1-yn-1-yl)benzene (**S8**, 51.7 mg, 0.30 mmol, 1.5 equiv), 1,1,1,3,3,3-hexafluoroisopropyl acrylate (**2e**, 200 mg, 0.90 mmol, 4.5 equiv), 4-iodobenzonitrile (**3**, 45.8 mg, 0.20 mmol, 1.0 equiv) and 2-iodo-2-methylpropane (**4**, 110 mg, 0.60 mmol, 3.0 equiv). The reaction time was 12 h. Flash column chromatography (DCM/hexanes, 10–50%) afforded the title compound as a colorless oil (66.7 mg, 60%).

^{1}H NMR (400 MHz, acetone-*d*$_6$) δ 7.80–7.72 (m, 2H), 7.55–7.48 (m, 2H), 7.36–7.28 (m, 1H), 7.00–6.79 (m, 3H), 6.38–6.17 (m, 1H), 3.79 (d, *J* = 2.2 Hz, 3H), 2.98–2.86 (m, 1H), 2.67–2.55 (m, 1H), 2.48–2.40 (m, 1H), 1.97 (d, *J* = 1.8 Hz, 3H), 1.80–1.68 (m, 1H), 1.50–1.39 (m, 1H), 0.79 (s, 4.5H), 0.75 (s, 4.5H).

^{13}C{^{1}H} NMR (100 MHz, acetone-*d*$_6$) δ 205.67, 205.43, 173.85, 173.76, 160.93, 160.89, 143.36, 143.08, 138.64, 138.38, 133.09, 133.07, 130.55, 130.48, 129.89, 129.75, 121.76 (q, *J* = 282.2 Hz), 121.71, 121.60, 119.38, 119.35, 115.23, 115.13, 113.85, 113.75, 111.48, 111.37, 109.79, 109.60, 102.05, 101.77, 67.25 (sept, *J* = 34.1 Hz), 55.53, 55.52, 45.34, 45.01, 40.31, 40.21, 39.44, 39.41, 31.12, 31.01, 29.39, 29.36, 18.60, 18.43.

$^{19}F\{^1H\}$ NMR (377 MHz, acetone-d_6) δ -73.57 – -73.69 (m).

HRMS (ESI): *m/z* $[M+H]^+$ calcd for $C_{29}H_{30}F_6NO_3^+$, 554.2124; found, 554.2128.

1,1,1,3,3,3-Hexafluoropropan-2-yl 6-(4-cyanophenyl)-6-(2-methoxyphenyl)-4-methyl-2-neopentylhexa-4,5-dienoate (9)

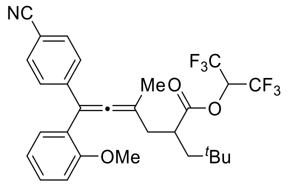

The product was prepared by **General procedure II** with 1-methoxy-2-(3-methylbut-3-en-1-yn-1-yl)benzene (**S9**, 51.7 mg, 0.30 mmol, 1.5 equiv), 1,1,1,3,3,3-hexafluoroisopropyl acrylate (**2e**, 200 mg, 0.90 mmol, 4.5 equiv), 4-iodobenzonitrile (**3**, 45.8 mg, 0.20 mmol, 1.0 equiv) and 2-iodo-2-methylpropane (**4**, 110 mg, 0.60 mmol, 3.0 equiv). The reaction time was 12 h. Flash column chromatography (DCM/hexanes, 10–50%) afforded the title compound as a colorless oil (79.7 mg, 72%).

^1H NMR (400 MHz, acetone-d_6) δ 7.71–7.63 (m, 2H), 7.45–7.30 (m, 3H), 7.25 (ddd, J = 7.5, 4.1, 1.8 Hz, 1H), 7.13–7.06 (m, 1H), 7.04 – 6.98 (m, 1H), 6.44–6.02 (m, 1H), 3.80–3.65 (m, 3H), 3.03–2.89 (m, 1H), 2.63–2.48 (m, 1H), 2.44–2.29 (m, 1H), 1.93 (d, J = 3.8 Hz, 3H), 1.83–1.63 (m, 1H), 1.58–1.36 (m, 1H), 0.85 (s, 4.1H), 0.68 (s, 4.9H).

$^{13}C\{^1H\}$ NMR (100 MHz, acetone-d_6) δ 205.60, 205.56, 174.02, 173.78, 158.19, 158.15, 143.76, 143.60, 132.82, 132.75, 132.11, 131.99, 130.45, 130.42, 128.28, 128.15, 125.67, 125.61, 121.58, 121.79 (q, J = 284.2 Hz), 121.49, 119.55, 112.41, 112.34, 110.56, 110.54, 106.19, 105.86, 101.01, 100.75, 67.27 (sept, J = 34.2 Hz), 67.21 (sept, J = 34.2 Hz), 55.83, 55.79, 45.61, 44.78, 40.30, 40.10, 39.37, 39.17, 31.23, 30.90, 29.43, 29.35, 18.50, 18.46.

$^{19}F\{^1H\}$ NMR (377 MHz, acetone-d_6) δ -73.54 – -73.70 (m).

HRMS (ESI): *m/z* $[M+H]^+$ calcd for $C_{29}H_{30}F_6NO_3^+$, 554.2124; found, 554.2129.

1,1,1,3,3,3-Hexafluoropropan-2-yl 6-(4-cyanophenyl)-4-methyl-2-neopentyl-6-phenylhexa-4,5-dienoate (10)

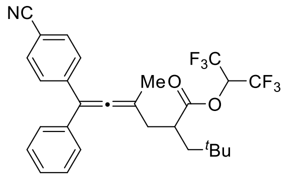

The product was prepared by **General procedure II** with (3-methylbut-3-en-1-yn-1-yl)benzene (**S10**, 42.7 mg, 0.30 mmol, 1.5 equiv), 1,1,1,3,3,3-hexafluoroisopropyl acrylate (**2e**, 200 mg, 0.90 mmol, 4.5 equiv), 4-iodobenzonitrile (**3**, 45.8 mg, 0.20 mmol, 1.0 equiv) and 2-iodo-2-methylpropane (**4**, 110 mg,

0.60 mmol, 3.0 equiv). The reaction time was 2 h. Flash column chromatography (DCM/hexanes, 0–50%) afforded the title compound as a pale-yellow oil (75.4 mg, 72%).

^{1}H NMR (400 MHz, acetone-d_6) δ 7.76 (dd, J = 8.3, 4.2 Hz, 2H), 7.50 (dd, J = 8.3, 3.4 Hz, 2H), 7.45–7.28 (m, 5H), 6.44–6.11 (m, 2H), 2.92 (dd, J = 6.1, 2.8 Hz, 1H), 2.60 (dt, J = 7.9, 5.4 Hz, 1H), 2.51–2.37 (m, 1H), 1.97 (s, 3H), 1.82–1.64 (m, 1H), 1.48–1.36 (m, 1H), 0.78 (s, 4.2H), 0.75 (s, 4.8H).

^{13}C{^{1}H} NMR (100 MHz, acetone-d_6) δ 205.78, 205.54, 173.84, 173.74, 143.45, 143.18, 137.27, 137.00, 133.10, 133.07, 129.88, 129.74, 129.53, 129.46, 129.42, 129.30, 128.57, 128.51, 121.76 (q, J = 281.7 Hz), 119.36, 119.33, 111.51, 111.40, 111.39, 109.90, 109.69, 102.01, 101.76, 67.26 (sept, J = 34.2 Hz), 45.26, 45.08, 40.31, 40.20, 39.48, 39.40, 31.10, 31.02, 29.39, 29.37, 18.65, 18.47.

^{19}F{^{1}H} NMR (377 MHz, acetone-d_6) δ -73.59 – -73.68 (m).

HRMS (ESI): *m/z* $[M+H]^+$ calcd for $C_{28}H_{28}F_6NO_2^+$, 524.2019; found, 524.2020.

1,1,1,3,3,3-Hexafluoropropan-2-yl 6,6-bis(4-cyanophenyl)-4-methyl-2-neopentylhexa-4,5-dienoate (11)

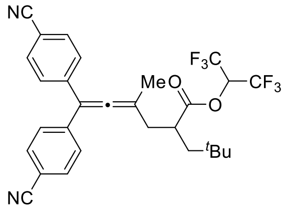

The product was prepared by **General procedure II** with 4-(3-methylbut-3-en-1-yn-1-yl)benzonitrile (**S11**, 50.2 mg, 0.30 mmol, 1.5 equiv), 1,1,1,3,3,3-hexafluoroisopropyl acrylate (**2e**, 200 mg, 0.90 mmol, 4.5 equiv), 4-iodobenzonitrile (**3**, 45.8 mg, 0.20 mmol, 1.0 equiv) and 2-iodo-2-methylpropane (**4**, 110 mg, 0.60 mmol, 3.0 equiv). The reaction time was 2 h. Flash column chromatography (DCM/hexanes, 10–50%) afforded the title compound as a colorless oil (88.9 mg, 81%).

^{1}H NMR (400 MHz, acetone-d_6) δ 7.85–7.76 (m, 4H), 7.58–7.48 (m, 4H), 6.29 (sept, J = 6.2 Hz, 1H), 2.98 – 2.89 (m, 1H), 2.68 – 2.60 (m, 1H), 2.55 – 2.45 (m, 1H), 2.00 (s, 3H), 1.82 – 1.68 (m, 1H), 1.46 – 1.36 (m, 1H), 0.78 (s, 9H).

^{13}C{^{1}H} NMR (100 MHz, acetone-d_6) δ 206.37, 173.68, 142.35, 142.09, 133.35, 133.30, 130.10, 129.94, 121.72 (q, J = 279.5 Hz), 119.22, 119.20, 111.99, 111.88, 108.91, 103.26, 67.26 (sept, J = 34.1 Hz), 45.36, 40.19, 39.24, 31.10, 29.37, 18.35.

^{19}F{^{1}H} NMR (377 MHz, acetone-d_6) δ -73.59 – -73.70 (m).

HRMS (ESI): *m/z* $[M+H]^+$ calcd for $C_{29}H_{27}F_6N_2O_2^+$, 549.1971; found, 549.1975.

Methyl 4-(1-(4-cyanophenyl)-5-(((1,1,1,3,3,3-hexafluoropropan-2-yl)oxy)carbonyl)-3,7,7-trimethylocta-1,2-dien-1-yl)benzoate (12)

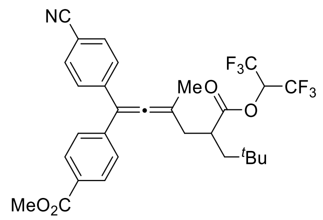

The product was prepared by **General procedure II** with methyl 4-(3-methylbut-3-en-1-yn-1-yl)benzoate (**S12**, 60.1 mg, 0.30 mmol, 1.5 equiv), 1,1,1,3,3,3-hexafluoroisopropyl acrylate (**2e**, 200 mg, 0.90 mmol, 4.5 equiv), 4-iodobenzonitrile (**3**, 45.8 mg, 0.20 mmol, 1.0 equiv) and 2-iodo-2-methylpropane (**4**, 110 mg, 0.60 mmol, 3.0 equiv). The reaction time was 2 h. Flash column chromatography (DCM/hexanes, 10–50%) afforded the title compound as a pale-yellow oil (93.0 mg, 80%).

^{1}H NMR (400 MHz, acetone-d_6) δ 8.07–8.00 (m, 2H), 7.86–7.74 (m, 2H), 7.56–7.49 (m, 2H), 7.49–7.41 (m, 2H), 6.36–6.23 (m, 1H), 3.89 (d, J = 2.0 Hz, 3H), 3.01–2.87 (m, 1H), 2.63 (ddd, J = 14.6, 5.8, 1.9 Hz, 1H), 2.48 (dd, J = 14.6, 8.3 Hz, 1H), 1.99 (s, 3H), 1.80–1.67 (m, 1H), 1.49–1.37 (m, 1H), 0.78 (s, 4.0H), 0.77 (s, 5.0H).

^{13}C{^{1}H} NMR (100 MHz, acetone-d_6) δ 206.23, 206.13, 173.75, 173.70, 166.86, 166.85, 142.83, 142.55, 142.17, 141.90, 133.26, 133.22, 130.55, 130.51, 130.29, 130.20, 130.07, 129.92, 129.40, 129.25, 121.79 (q, J = 281.7 Hz), 119.28, 119.25, 111.83, 111.72, 109.31, 109.20, 102.81, 102.70, 67.27 (sept, J = 34.1 Hz), 52.40, 52.38, 45.31, 45.27, 40.24, 40.20, 39.33, 39.27, 31.10, 31.08, 29.37, 18.48, 18.38.

^{19}F{^{1}H} NMR (377 MHz, acetone-d_6) δ -73.58 – -73.70 (m).

HRMS (ESI): *m/z* $[M+H]^+$ calcd for $C_{30}H_{30}F_6NO_4^+$, 582.2074; found, 582.2073.

4-(4-Cyanophenyl)-8-(((1,1,1,3,3,3-hexafluoropropan-2-yl)oxy)carbonyl)-6,10,10-trimethylundeca-4,5-dien-1-yl benzoate (13)

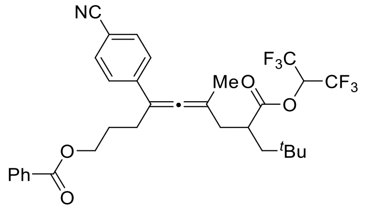

The product was prepared by **General procedure II** with 6-methylhept-6-en-4-yn-1-yl benzoate (**S13**, 68.5 mg, 0.30 mmol, 1.5 equiv), 1,1,1,3,3,3-hexafluoroisopropyl acrylate (**2e**, 200 mg, 0.90 mmol, 4.5 equiv), 4-iodobenzonitrile (**3**, 45.8 mg, 0.20 mmol, 1.0 equiv) and 2-iodo-2-methylpropane (**4**, 110 mg, 0.60 mmol, 3.0 equiv). The reaction time was 2 h. Flash column chromatography (DCM/hexanes, 10–50%) afforded the title compound as a colorless oil (65.8 mg, 54%).

^{1}H NMR (400 MHz, acetone-d_6) δ 8.03 (dt, J = 8.5, 1.3 Hz, 2H), 7.76–7.69 (m, 2H), 7.66–7.59 (m, 3H), 7.51 (dd, J = 8.5, 7.1 Hz, 2H), 6.41–6.25 (m, 1H), 4.52–4.38 (m, 2H), 2.92–2.84 (m, 1H), 2.73–2.62

(m, 2H), 2.59–2.51 (m, 1H), 2.45–2.32 (m, 1H), 1.90 (s, 3H), 2.10–1.99 (m, 2H), 1.82–1.71 (m, 1H), 1.58–1.46 (m, 1H), 0.88 (s, 4.8H), 0.79 (s, 4.2H).

$^{13}C\{^{1}H\}$ NMR (100 MHz, acetone-*d*6) δ 204.14, 203.86, 173.84, 173.69, 166.73, 143.30, 143.18, 133.85, 133.04, 132.99, 131.37, 130.17, 130.16, 129.38, 129.37, 127.75, 127.73, 121.81 (q, *J* = 284.8 Hz), 119.46, 110.82, 105.81, 105.52, 102.64, 102.39, 67.24 (q, *J* = 34.5 Hz), 64.96, 64.93, 45.62, 45.36, 40.35, 40.24, 39.43, 39.05, 31.31, 31.19, 29.43, 28.04, 28.03, 27.24, 27.14, 18.74, 18.57.

$^{19}F\{^{1}H\}$ NMR (377 MHz, acetone-*d*6) δ -73.60 – -73.68 (m).

HRMS (ESI): *m/z* $[M+H]^+$ calcd for $C_{32}H_{34}F_6NO_4^+$, 610.2387; found, 610.2387.

1,1,1,3,3,3-Hexafluoropropan-2-yl 8-cyano-6-(4-cyanophenyl)-4-methyl-2-neopentylocta-4,5-dienoate (14)

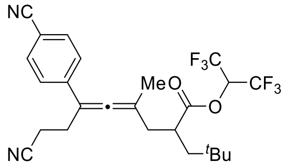

The product was prepared by **General procedure II** with 6-methylhept-6-en-4-ynenitrile (**S14**, 35.8 mg, 0.30 mmol, 1.5 equiv), 1,1,1,3,3,3-hexafluoroisopropyl acrylate (**2e**, 200 mg, 0.90 mmol, 4.5 equiv), 4-iodobenzonitrile (**3**, 45.8 mg, 0.20 mmol, 1.0 equiv) and 2-iodo-2-methylpropane (**4**, 110 mg, 0.60 mmol, 3.0 equiv). The reaction time was 2 h. Flash column chromatography (ethyl actate/hexanes, 0–10%) afforded the title compound as a colorless oil (73.1 mg, 73%).

^{1}H NMR (400 MHz, acetone-*d*6) δ 7.77–7.73 (m, 2H), 7.67–7.57 (m, 2H), 6.52–6.16 (m, 1H), 2.95–2.86 (m, 2H), 2.83–2.77 (m, 1H), 2.72 (q, *J* = 6.3 Hz, 2H), 2.65–2.37 (m, 2H), 1.95 (d, *J* = 1.5 Hz, 3H), 1.84–1.71 (m, 1H), 1.58–1.45 (m, 1H), 0.89 (s, 3.5H), 0.81 (s, 5.5H).

$^{13}C\{^{1}H\}$ NMR (100 MHz, acetone-*d*6) δ 203.94, 203.40, 173.92, 173.72, 142.48, 142.35, 133.15, 133.10, 127.65, 127.63, 121.79 (q, *J* = 282.0 Hz), 120.24, 120.20, 119.37, 119.36, 111.17, 111.15, 104.94, 104.55, 104.50, 104.09, 67.25 (sept, *J* = 34.1 Hz), 45.58, 45.46, 40.29, 40.17, 39.52, 38.97, 31.30, 31.24, 29.49, 29.41, 26.63, 26.56, 18.88, 18.54, 16.21, 16.18.

$^{19}F\{^{1}H\}$ NMR (377 MHz, acetone-*d*6) δ -73.59 – -73.72 (m).

HRMS (ESI): *m/z* $[M+H]^+$ calcd for $C_{25}H_{27}F_6N_2O_2^+$, 501.1971; found, 501.1975.

1,1,1,3,3,3-Hexafluoropropan-2-yl 9-chloro-6-(4-cyanophenyl)-4-methyl-2-neopentylnona-4,5-dienoate (15)

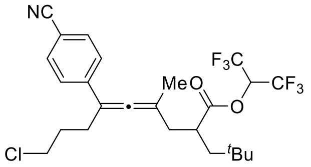

The product was prepared by **General procedure II** with 7-chloro-2-methylhept-1-en-3-yne (**S15**, 42.8 mg, 0.30 mmol, 1.5 equiv), 1,1,1,3,3,3-hexafluoroisopropyl acrylate (**2e**, 200 mg, 0.90 mmol, 4.5 equiv), 4-iodobenzonitrile (**3**, 45.8 mg, 0.20 mmol, 1.0 equiv) and 2-iodo-2-methylpropane (**4**, 110 mg, 0.60 mmol, 3.0 equiv). The reaction time was 2 h. Flash column chromatography (DCM/hexanes, 10–50%) afforded the title compound as a colorless oil (57.6 mg, 55%).

^{1}H NMR (400 MHz, acetone-*d*$_6$) δ 7.77–7.69 (m, 2H), 7.66–7.56 (m, 2H), 6.45–6.24 (m, 1H), 3.72 (td, *J* = 6.5, 1.6 Hz, 2H), 2.95–2.82 (m, 1H), 2.69–2.60 (m, 2H), 2.58–2.49 (m, 1H), 2.47–2.33 (m, 1H), 2.03–1.98 (m, 2H), 1.90 (s, 3H), 1.83–1.71 (m, 1H), 1.59–1.47 (m, 1H), 0.89 (s, 4.4H), 0.81 (s, 4.6H).

^{13}C{^{1}H} NMR (100 MHz, acetone-*d*$_6$) δ 204.14, 203.88, 173.88, 173.72, 143.16, 143.03, 133.07, 133.03, 127.74, 127.72, 121.83 (q, *J* = 285.3 Hz), 119.45, 110.88, 105.31, 105.04, 102.57, 102.37, 67.26 (sept, *J* = 33.8 Hz), 45.67, 45.29, 45.23, 45.17, 40.34, 40.25, 39.46, 39.04, 31.89, 31.86, 31.33, 31.20, 27.96, 27.86, 18.68, 18.50.

^{19}F{^{1}H} NMR (377 MHz, acetone-*d*$_6$) δ -73.58 – -73.74 (m).

HRMS (ESI): *m/z* [M+H]$^+$ calcd for $C_{25}H_{29}ClF_6NO_2^+$, 524.1786; found, 524.1784.

1,1,1,3,3,3-Hexafluoropropan-2-yl 9-chloro-6-(4-cyanophenyl)-2-neopentyl-4-phenylnona-4,5-dienoate (16)

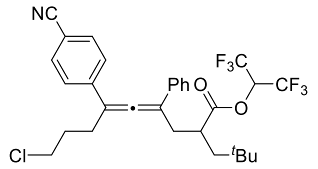

The product was prepared by **General procedure II** with (7-chlorohept-1-en-3-yn-2-yl)benzene (**S16**, 61.4 mg, 0.30 mmol, 1.5 equiv), 1,1,1,3,3,3-hexafluoroisopropyl acrylate (**2e**, 200 mg, 0.90 mmol, 4.5 equiv), 4-iodobenzonitrile (**3**, 45.8 mg, 0.20 mmol, 1.0 equiv) and 2-iodo-2-methylpropane (**4**, 110 mg, 0.60 mmol, 3.0 equiv). The reaction time was 12 h. Flash column chromatography (DCM/hexanes, 10–50%) afforded the title compound as a colorless oil (75.0 mg, 64%).

^{1}H NMR (400 MHz, acetone-*d*$_6$) δ 7.82–7.74 (m, 2H), 7.71–7.64 (m, 2H), 7.52–7.46 (m, 2H), 7.41 (tt, *J* = 6.4, 1.9 Hz, 2H), 7.35–7.27 (m, 1H), 6.49–6.25 (m, 1H), 3.80–3.66 (m, 2H), 3.23–3.11 (m, 1H), 2.97–2.70 (m, 4H), 2.18–2.07 (m, 2H), 1.95–1.72 (m, 1H), 1.70–1.57 (m, 1H), 0.82 (s, 2.9H), 0.76 (s, 6.1H).

^{13}C{^{1}H} NMR (100 MHz, acetone-*d*$_6$) δ 207.32, 207.19, 173.70, 173.53, 142.03, 141.81, 135.70, 135.63, 133.31, 133.30, 129.81, 129.77, 128.75, 127.80, 127.76, 127.04, 126.93, 121.80 (q, *J* = 281.2 Hz), 119.32, 111.54, 111.49, 109.26, 109.15, 108.80, 108.73, 67.34 (sept, *J* = 34.1 Hz), 67.31 (sept, *J* = 34.3 Hz), 45.57, 45.30, 45.25, 40.54, 40.46, 35.90, 35.58, 32.07, 31.88, 31.33, 31.23, 28.21.

^{19}F{^{1}H} NMR (377 MHz, acetone-*d*$_6$) δ -73.64 – -73.74 (m).

HRMS (ESI): *m/z* [M+H]$^+$ calcd for $C_{30}H_{31}ClF_6NO_2^+$, 586.1942; found, 586.1942.

1,1,1,3,3,3-Hexafluoropropan-2-yl 6-(4-cyanophenyl)-6-(4-methoxyphenyl)-2-neopentyl-4-(phenoxymethyl)hexa-4,5-dienoate (17)

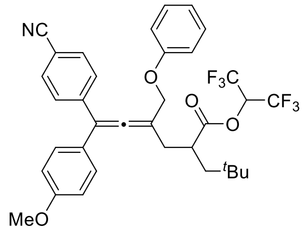

The product was prepared by **General procedure II** with 1-methoxy-4-(3-(phenoxymethyl)but-3-en-1-yn-1-yl)benzene (**S17**, 79.3 mg, 0.30 mmol, 1.5 equiv), 1,1,1,3,3,3-hexafluoroisopropyl acrylate (**2e**, 200 mg, 0.90 mmol, 4.5 equiv), 4-iodobenzonitrile (**3**, 45.8 mg, 0.20 mmol, 1.0 equiv) and 2-iodo-2-methylpropane (**4**, 110 mg, 0.60 mmol, 3.0 equiv). The reaction time was 12 h. Flash column chromatography (DCM/hexanes, 10–50%) afforded the title compound as a colorless oil (100.0 mg, 77%).

^{1}H NMR (400 MHz, acetone-d_6) δ 7.78–7.70 (m, 2H), 7.50–7.43 (m, 2H), 7.28–7.22 (m, 2H), 7.22–7.15 (m, 2H), 7.01–6.95 (m, 2H), 6.95–6.84 (m, 3H), 6.37–6.24 (m, 1H), 4.86 (s, 2H), 3.83 (d, J = 2.7 Hz, 3H), 3.10–2.96 (m, 1H), 2.77–2.69 (m, 1H), 2.60–2.43 (m, 1H), 1.80–1.65 (m, 1H), 1.55–1.43 (m, 1H), 0.75 (s, 4.8H), 0.73 (s, 4.2H).

^{13}C{^{1}H} NMR (150 MHz, acetone-d_6) δ 205.44, 205.11, 173.77, 173.71, 160.65, 160.62, 159.11, 142.86, 142.57, 133.07, 133.05, 130.69, 130.57, 130.27, 130.25, 130.00, 129.86, 128.25, 128.02, 122.09 (q), 121.92, 121.89, 119.34, 119.32, 115.93, 115.89, 115.00, 114.93, 112.50, 112.19, 111.75, 111.64, 103.48, 103.20, 68.58, 68.49, 67.77–66.73 (m), 55.69, 55.68, 45.22, 45.05, 40.48, 40.43, 35.19, 35.09, 31.16, 31.11, 29.39, 29.37.

^{19}F{^{1}H} NMR (377 MHz, acetone-d_6) δ -73.62 – -73.65 (m).

HRMS (ESI): *m/z* $[M+H]^+$ calcd for $C_{35}H_{34}F_6NO_4^+$, 646.2387; found, 646.2385.

1,1,1,3,3,3-Hexafluoropropan-2-yl 4-((*tert*-butyldimethylsilyl)oxy)-6-(4-cyanophenyl)-2-neopentyl-6-phenylhexa-4,5-dienoate (18)

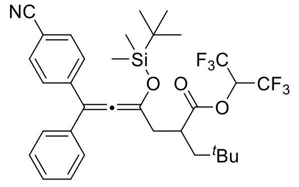

The product was prepared by **General procedure II** with tert-butyldimethyl((4-phenylbut-1-en-3-yn-2-yl)oxy)silane (**S18**, 77.5 mg, 0.30 mmol, 1.5 equiv), 1,1,1,3,3,3-hexafluoroisopropyl acrylate (**2e**, 200 mg, 0.90 mmol, 4.5 equiv), 4-iodobenzonitrile (**3**, 45.8 mg, 0.20 mmol, 1.0 equiv) and 2-iodo-2-methylpropane (**4**, 110 mg, 0.60 mmol, 3.0 equiv). The reaction time was 2 h. Flash column

chromatography (DCM/hexanes, 5–30%) afforded the title compound as a colorless oil (104.6 mg, 82%).

^{1}H NMR (400 MHz, acetone-*d*$_6$) δ 7.88–7.76 (m, 2H), 7.61–7.53 (m, 2H), 7.49–7.32 (m, 5H), 6.41–6.26 (m, 1H), 3.12–3.02 (m, 1H), 2.83–2.70 (m, 1H), 2.57–2.45 (m, 1H), 1.88–1.71 (m, 1H), 1.54–1.44 (m, 1H), 0.97–0.92 (m, 9H), 0.89–0.84 (m, 9H), 0.07–0.00 (m, 6H).

^{13}C{^{1}H} NMR (150 MHz, acetone-*d*$_6$) δ 201.26, 201.14, 173.80, 173.72, 143.38, 142.79, 137.60, 137.08, 133.21, 133.14, 130.15, 130.13, 129.53, 129.47, 129.35, 129.32, 129.07, 129.03, 127.73, 127.59, 121.80 (q, *J* = 281.9 Hz), 119.26, 119.22, 117.80, 117.65, 112.13, 112.04, 67.28 (sept, *J* = 34.1 Hz), 47.96, 47.19, 44.82, 44.75, 40.46, 40.39, 38.99, 38.95, 31.21, 29.45, 29.42, 25.89, 18.51, -4.57, -4.64, -4.95, -5.01.

^{19}F{^{1}H} NMR (377 MHz, acetone-*d*$_6$) δ -73.77, -73.79.

HRMS (ESI): *m/z* [M+H]$^+$ calcd for $C_{33}H_{40}F_6NO_3Si^+$, 640.2676; found, 640.2673.

Methyl 4-(1-(4-cyanophenyl)-5-(((1,1,1,3,3,3-hexafluoropropan-2-yl)oxy)carbonyl)-7,7-dimethylocta-1,2-dien-1-yl)benzoate (19)

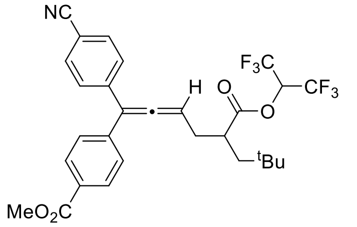

The product was prepared by **General procedure III** with methyl 4-(but-3-en-1-yn-1-yl)benzoate (**S19**, 55.9 mg, 0.30 mmol, 1.5 equiv), 1,1,1,3,3,3-hexafluoroisopropyl acrylate (**2e**, 200 mg, 0.90 mmol, 4.5 equiv), 4-iodobenzonitrile (**3**, 45.8 mg, 0.20 mmol, 1.0 equiv) and 2-iodo-2-methylpropane (**4**, 110 mg, 0.60 mmol, 3.0 equiv). Flash column chromatography (ethyl acetate/hexanes, 0–20%) afforded the title compound as a colorless oil (36.0 mg, 32%).

^{1}H NMR (400 MHz, acetone-*d*$_6$) δ 8.04 (dd, *J* = 8.5, 3.1 Hz, 2H), 7.81 (dd, *J* = 8.6, 3.3 Hz, 2H), 7.54 (dd, *J* = 8.4, 5.3 Hz, 2H), 7.47 (dd, *J* = 8.3, 6.3 Hz, 2H), 6.40 – 6.31 (m, 1H), 5.96 (t, *J* = 7.5 Hz, 1H), 3.90 (d, *J* = 1.2 Hz, 3H), 2.98 – 2.86 (m, 1H), 2.73 – 2.61 (m, 1H), 2.59 – 2.47 (m, 1H), 1.87 – 1.78 (m, 1H), 1.52 – 1.46 (m, 1H), 0.84 (d, *J* = 0.7 Hz, 9H).

^{13}C NMR (100 MHz, acetone-*d*$_6$) δ 208.29, 208.25, 173.61, 173.58, 166.83, 166.82, 142.01, 141.89, 141.33, 141.22, 133.64, 133.57, 133.32, 133.29, 132.51, 132.47, 130.59, 130.56, 130.42, 130.38, 129.99, 129.96, 129.32, 129.28, 121.78 (q, *J* = 281.0 Hz), 119.22, 119.21, 111.98, 111.94, 109.82, 109.79, 93.14, 67.24 (hept, *J* = 34.2 Hz), 52.42, 52.41, 45.22, 45.19, 41.71, 41.68, 34.27, 34.21, 31.23, 29.40.

^{19}F{^{1}H} NMR (377 MHz, acetone-*d*$_6$) δ -73.62 – -73.70 (m).

HRMS (ESI): *m/z* [M+H]$^+$ calcd for $C_{29}H_{28}F_6NO_4^+$, 568.1917; found, 568.1913.

1,1,1,3,3,3-Hexafluoropropan-2-yl 3-(1-(5-chloro-2-(4-cyanophenyl)pent-1-en-1-ylidene)-1,2,3,4-tetrahydronaphthalen-2-yl)-4,4-dimethylpentanoate (20)

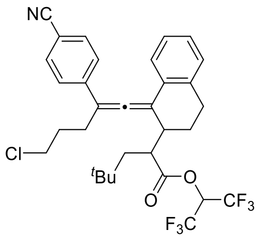

The product was prepared by **General procedure II** with 4-(5-chloropent-1-yn-1-yl)-1,2-dihydronaphthalene (**S19**, 69.2 mg, 0.30 mmol, 1.5 equiv), 1,1,1,3,3,3-hexafluoroisopropyl acrylate (**2e**, 200 mg, 0.90 mmol, 4.5 equiv), 4-iodobenzonitrile (**3**, 45.8 mg, 0.20 mmol, 1.0 equiv) and 2-iodo-2-methylpropane (**4**, 110 mg, 0.60 mmol, 3.0 equiv). The reaction time was 12 h. Flash column chromatography (DCM/hexanes, 10–50%) afforded the title compound as a pale-yellow oil (77.1 mg, 63%).

^{1}H NMR (400 MHz, acetone-d_6) δ 7.80–7.72 (m, 2H), 7.72–7.63 (m, 2H), 7.38 (ddd, J = 5.9, 4.6, 2.5 Hz, 1H), 7.24–7.10 (m, 3H), 6.54–6.34 (m, 1H), 3.86–3.63 (m, 2H), 3.15–2.74 (m, 6H), 2.28–2.08 (m, 3H), 1.98–1.50 (m, 3H), 0.87 (s, 5.9H), 0.77 (s, 3.1H).

^{13}C{^{1}H} NMR (100 MHz, acetone-d_6) δ 204.91, 173.56, 173.15, 141.97, 141.92, 137.01, 136.30, 133.38, 133.32, 131.34, 130.50, 130.28, 128.73, 128.50, 128.11, 127.83, 127.60, 127.50, 127.35, 127.23, 121.77 (q, J = 281.5 Hz), 119.33, 119.32, 111.52, 111.35, 111.06, 109.86, 109.42, 108.93, 67.23 (sept, J = 34.1 Hz), 67.38 (sept, J = 33.94 Hz), 45.46, 45.40, 45.07, 44.13, 43.85, 43.45, 43.07, 41.88, 32.50, 32.10, 31.16, 31.12, 29.43, 28.73, 28.58, 27.24, 26.51, 25.75.

^{19}F{^{1}H} NMR (377 MHz, acetone-d_6) δ -73.12 – -73.16 (m), -73.43.

HRMS (ESI): *m/z* [M+H]$^+$ calcd for $C_{32}H_{33}ClF_6NO_2^+$, 612.2099; found, 612.2097.

1,1,1,3,3,3-Hexafluoropropan-2-yl 9-(acryloyloxy)-6-(4-cyanophenyl)-4-methyl-2-neopentylnona-4,5-dienoate (21)

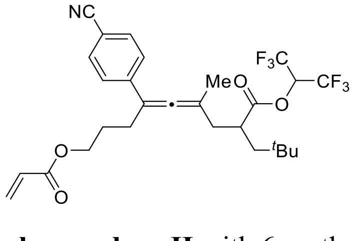

The product was prepared by **General procedure II** with 6-methylhept-6-en-4-yn-1-ol (**S20**, 37.3 mg, 0.30 mmol, 1.5 equiv), 1,1,1,3,3,3-hexafluoroisopropyl acrylate (**2e**, 200 mg, 0.90 mmol, 4.5 equiv), 4-iodobenzonitrile (**3**, 45.8 mg, 0.20 mmol, 1.0 equiv) and 2-iodo-2-methylpropane (**4**, 110 mg, 0.60 mmol, 3.0 equiv). The reaction time was 12 h. Flash column chromatography (DCM/hexanes, 10–50%) afforded the title compound as a colorless oil (56.0 mg, 50%).

^{1}**H NMR** (400 MHz, acetone-d_6) δ 7.78–7.68 (m, 2H), 7.66–7.49 (m, 2H), 6.42–6.34 (m, 1H), 6.34–6.26 (m, 1H), 6.21–6.11 (m, 1H), 5.93–5.83 (m, 1H), 4.45–3.91 (m, 2H), 2.94–2.83 (m, 1H), 2.63–2.59 (m, 1H), 2.59–2.56 (m, 1H), 2.56–2.50 (m, 1H), 2.44–2.33 (m, 1H), 1.99–1.91 (m, 2H), 1.90 (d, J = 0.9 Hz, 3H), 1.78–1.69 (m, 1H), 1.57–1.46 (m, 1H), 0.89 (s, 4.1H), 0.81 (s, 4.9H).
^{13}**C{^{1}H} NMR** (100 MHz, acetone-d_6) δ 203.20, 202.91, 172.95, 172.80, 165.45, 142.41, 142.28, 132.14, 132.10, 130.10, 128.59, 126.82, 126.81, 120.91 (q, J = 286.3 Hz), 118.56, 118.56, 109.92, 104.90, 104.58, 101.75, 101.48, 66.35 (sept, J = 68.2, 34.2 Hz), 63.52, 63.51, 44.73, 44.45, 39.44, 39.34, 38.55, 38.14, 30.42, 30.31, 27.07, 27.04, 26.19, 26.10, 17.83, 17.64.
^{19}**F{^{1}H} NMR** (377 MHz, acetone-d_6) δ -73.60 – -73.72 (m).
HRMS (ESI): *m/z* [M+H]$^+$ calcd for $C_{28}H_{32}F_6NO_4^+$, 560.2230; found, 560.2228.

Methyl 6-(4-cyanophenyl)-6-(4-methoxyphenyl)-4-methyl-2-neopentylhexa-4,5-dienoate (5ad)

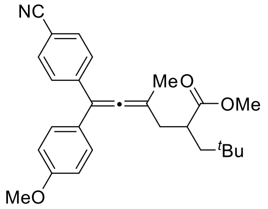

The product was prepared by **General procedure III** with 1-methoxy-4-(3-methylbut-3-en-1-yn-1-yl)benzene (**1a**, 51.7 mg, 0.30 mmol, 1.5 equiv), methyl acrylate (**2d**, 77.5 mg, 0.90 mmol, 4.5 equiv), 4-iodobenzonitrile (**3**, 45.8 mg, 0.20 mmol, 1.0 equiv) and 2-iodo-2-methylpropane (**4**, 110 mg, 0.60 mmol, 3.0 equiv). Flash column chromatography (DCM/hexanes, 10–50%) afforded the title compound as a colorless oil (44.3 mg, 53%).

***tert*-Butyl 6-(4-cyanophenyl)-6-(4-methoxyphenyl)-4-methyl-2-neopentylhexa-4,5-dienoate (5af)**

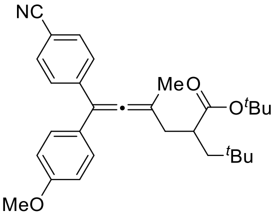

The product was prepared by **General procedure III** with 1-methoxy-4-(3-methylbut-3-en-1-yn-1-yl)benzene (**1a**, 51.7 mg, 0.30 mmol, 1.5 equiv), *tert*-butyl acrylate (**2f**, 115 mg, 0.90 mmol, 4.5 equiv), 4-iodobenzonitrile (**3**, 45.8 mg, 0.20 mmol, 1.0 equiv) and 2-iodo-2-methylpropane (**4**, 110 mg, 0.60 mmol, 3.0 equiv). Flash column chromatography (DCM/hexanes, 10–50%) afforded the title compound as a colorless oil (52.4 mg, 57%).
^{1}**H NMR** (400 MHz, acetone-d_6) δ 7.77–7.70 (m, 2H), 7.56–7.48 (m, 2H), 7.31–7.20 (m, 2H), 7.00–6.93 (m, 2H), 3.82 (d, J = 1.5 Hz, 3H), 2.60–2.42 (m, 1H), 2.31–2.19 (m, 1H), 1.92 (s, 3H), 1.67–1.57 (m, 1H), 1.39 (s, 4.1H), 1.36 (s, 4.9H), 1.27–1.21 (m, 1H), 0.79 (s, 4.9H), 0.77 (s, 4.1H).

^{13}C{^{1}H} NMR (100 MHz, acetone-d_6) δ 205.33, 205.22, 176.00, 175.94, 160.26, 160.23, 144.23, 143.91, 133.00, 132.98, 130.56, 130.54, 129.79, 129.75, 129.49, 129.23, 119.42, 119.40, 114.89, 114.84, 111.16, 111.09, 108.91, 108.81, 102.52, 102.39, 80.36, 80.34, 55.64, 55.63, 45.92, 45.72, 41.82, 41.79, 39.92, 31.22, 31.16, 29.78, 29.76, 28.12, 28.10, 18.84, 18.78.
HRMS (ESI): *m/z* $[M+Na]^+$ calcd for $C_{30}H_{37}NNaO_3^+$, 482.2666; found, 482.2666.

Ethyl 6-(4-cyanophenyl)-6-(4-methoxyphenyl)-4-methyl-2-neopentylhexa-4,5-dienoate (22)

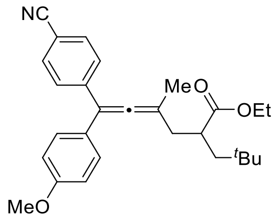

The product was prepared by **General procedure III** with 1-methoxy-4-(3-methylbut-3-en-1-yn-1-yl)benzene (**1a**, 51.7 mg, 0.30 mmol, 1.5 equiv), ethyl acrylate (90.1 mg, 0.90 mmol, 4.5 equiv), 4-iodobenzonitrile (**3**, 45.8 mg, 0.20 mmol, 1.0 equiv) and 2-iodo-2-methylpropane (**4**, 110 mg, 0.60 mmol, 3.0 equiv). Flash column chromatography (DCM/hexanes, 10–50%) afforded the title compound as a colorless oil (42.3 mg, 49%).
^{1}H NMR (400 MHz, acetone-d_6) δ 7.75 (dd, J = 8.5, 1.7 Hz, 2H), 7.49 (dd, J = 8.5, 6.9 Hz, 2H), 7.23 (dd, J = 8.8, 3.6 Hz, 2H), 6.96 (d, J = 8.8 Hz, 2H), 4.05–3.71 (m, 5H), 2.73–2.62 (m, 1H), 2.56–2.45 (m, 1H), 2.34–2.23 (m, 1H), 1.91 (d, J = 0.9 Hz, 3H), 1.73–1.61 (m, 1H), 1.37–1.29 (m, 1H), 1.18–1.01 (m, 3H), 0.81 (s, 4.9H), 0.79 (s, 4.1H).
^{13}C{^{1}H} NMR (100 MHz, acetone-d_6) δ 205.04, 204.95, 176.57, 176.52, 160.24, 144.08, 143.96, 132.98, 130.54, 130.47, 129.76, 129.66, 129.38, 129.27, 119.42, 114.85, 114.83, 111.12, 111.10, 109.01, 108.88, 102.50, 102.35, 60.77, 60.74, 55.64, 46.74, 46.56, 40.99, 40.08, 40.03, 31.24, 31.19, 29.65, 18.84, 18.75, 14.32, 14.29.
HRMS (ESI): *m/z* $[M+H]^+$ calcd for $C_{28}H_{34}NO_3^+$, 432.2533; found, 432.2532.

Methyl 6-(4-cyanophenyl)-6-(4-methoxyphenyl)-2-neopentyl-4-(phenoxymethyl)hexa-4,5-dienoate (23)

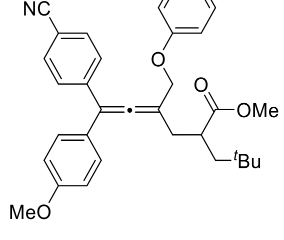

The product was prepared by **General procedure III** with 1-methoxy-4-(3-(phenoxymethyl)but-3-en-1-yn-1-yl)benzene (**S17**, 79.3 mg, 0.30 mmol, 1.5 equiv), methyl acrylate (**2d**, 77.5 mg, 0.90 mmol, 4.5

equiv), 4-iodobenzonitrile (**3**, 45.8 mg, 0.20 mmol, 1.0 equiv) and 2-iodo-2-methylpropane (**4**, 110 mg, 0.60 mmol, 3.0 equiv). Flash column chromatography (DCM/hexanes, 10–50%) afforded the title compound as a colorless oil (43.7 mg, 43%).

^{1}H NMR (600 MHz, acetone-*d*$_6$) δ 7.75–7.70 (m, 2H), 7.45–7.39 (m, 2H), 7.23–7.18 (m, 2H), 7.18–7.13 (m, 2H), 6.97–6.94 (m, 2H), 6.94–6.91 (m, 2H), 6.90–6.87 (m, 1H), 4.82 (d, *J* = 3.3 Hz, 2H), 3.82 (s, 3H), 3.43–3.33 (m, 3H), 2.82–2.74 (m, 1H), 2.65–2.57 (m, 1H), 2.47–2.41 (m, 1H), 1.77–1.62 (m, 1H), 1.45–1.34 (m, 1H), 0.79 (s, 4.6H), 0.78 (s, 4.4H).

^{13}C{^{1}H} NMR (150 MHz, acetone-*d*$_6$) δ 204.77, 204.68, 177.01, 176.97, 160.47, 159.20, 143.06, 142.99, 132.98, 132.96, 130.61, 130.57, 130.20, 129.88, 129.82, 128.44, 128.38, 121.76, 119.37, 115.93, 115.91, 114.87, 112.09, 111.99, 111.47, 111.44, 104.28, 104.16, 68.65, 68.60, 55.66, 51.80, 51.75, 47.05, 46.95, 41.01, 40.99, 35.76, 35.68, 31.21, 31.18, 29.59.

HRMS (ESI): *m/z* [M+H]$^+$ calcd for $C_{33}H_{36}NO_4^+$, 510.2639; found, 510.2643.

2,2,2-Trifluoroethyl 6-(4-cyanophenyl)-6-(4-methoxyphenyl)-4-methyl-2-neopentylhexa-4,5-dienoate (24)

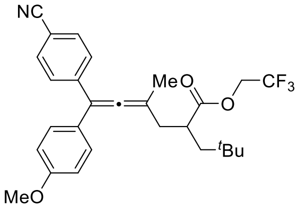

The product was prepared by **General procedure III** with 1-methoxy-4-(3-methylbut-3-en-1-yn-1-yl)benzene (**1a**, 51.7 mg, 0.30 mmol, 1.5 equiv), 2,2,2-trifluoroethyl acrylate (138.7 mg, 0.90 mmol, 4.5 equiv), 4-iodobenzonitrile (**3**, 45.8 mg, 0.20 mmol, 1.0 equiv) and 2-iodo-2-methylpropane (**4**, 110 mg, 0.60 mmol, 3.0 equiv). Flash column chromatography (DCM/hexanes, 10–50%) afforded the title compound as a pale-yellow oil (66.0 mg, 68%).

^{1}H NMR (400 MHz, acetone-*d*$_6$) δ 7.75 (dd, *J* = 8.4, 4.5 Hz, 2H), 7.49 (dd, *J* = 9.4, 8.4 Hz, 2H), 7.23 (dd, *J* = 8.9, 2.2 Hz, 2H), 6.97 (dd, *J* = 8.9, 2.6 Hz, 2H), 4.55–4.10 (m, 2H), 3.82 (d, *J* = 0.9 Hz, 3H), 2.82 (s, 1H), 2.64–2.49 (m, 1H), 2.45–2.29 (m, 1H), 1.93 (d, *J* = 1.1 Hz, 3H), 1.74–1.64 (m, 1H), 1.45–1.35 (m, 1H), 0.82 (s, 5.2H), 0.80 (s, 3.8H).

^{13}C{^{1}H} NMR (100 MHz, acetone-*d*$_6$) δ 204.90, 175.17, 175.09, 160.32, 160.31, 143.81, 143.78, 133.03, 133.01, 130.58, 130.45, 129.76, 129.61, 129.18, 124.38 (q, *J* = 278.4 Hz), 119.37, 114.89, 111.28, 111.22, 109.29, 109.21, 102.19, 102.17, 60.71 (q, *J* = 35.89 Hz), 55.64, 46.37, 46.21, 40.69, 40.61, 39.87, 39.80, 31.22, 31.16, 29.51, 29.50, 18.76, 18.72.

^{19}F{^{1}H} NMR (377 MHz, acetone-*d*$_6$) δ -74.15, -74.17.

HRMS (ESI): *m/z* [M+H]$^+$ calcd for $C_{28}H_{31}F_3NO_3^+$, 486.2251; found, 486.2252.

2-Chloroethyl 6-(4-cyanophenyl)-6-(4-methoxyphenyl)-4-methyl-2-neopentylhexa-4,5-dienoate (25)

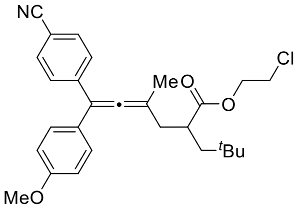

The product was prepared by **General procedure III** with 1-methoxy-4-(3-methylbut-3-en-1-yn-1-yl)benzene (**1a**, 51.7 mg, 0.30 mmol, 1.5 equiv), 2-chloroethyl acrylate (121 mg, 0.90 mmol, 4.5 equiv), 4-iodobenzonitrile (**3**, 45.8 mg, 0.20 mmol, 1.0 equiv) and 2-iodo-2-methylpropane (**4**, 110 mg, 0.60 mmol, 3.0 equiv). Flash column chromatography (DCM/hexanes, 10–50%) afforded the title compound as a colorless oil (28.1 mg, 30%).

^{1}H NMR (400 MHz, acetone-d_6) δ 7.78–7.71 (m, 2H), 7.52–7.46 (m, 2H), 7.23 (dd, J = 8.8, 4.9 Hz, 2H), 6.97 (dd, J = 8.8, 1.8 Hz, 2H), 4.18–3.92 (m, 2H), 3.82 (d, J = 1.3 Hz, 3H), 3.69–3.58 (m, 2H), 2.78–2.71 (m, 1H), 2.61–2.50 (m, 1H), 2.36–2.29 (m, 1H), 1.93 (s, 3H), 1.75–1.65 (m, 1H), 1.41–1.32 (m, 2H), 0.83 (s, 5.3H), 0.81 (s, 3.7H).

^{13}C{^{1}H} NMR (100 MHz, acetone-d_6) δ 204.95, 204.91, 176.38, 176.28, 160.29, 144.01, 143.92, 133.03, 133.01, 130.59, 130.47, 129.80, 129.65, 129.33, 129.25, 119.42, 114.88, 111.21, 111.15, 109.08, 102.45, 102.37, 65.02, 64.95, 55.64, 46.71, 46.56, 42.72, 42.68, 40.95, 40.92, 40.06, 40.03, 31.29, 31.23, 18.81, 18.79.

HRMS (ESI): *m/z* $[M+H]^+$ calcd for $C_{28}H_{33}ClNO_3^+$, 466.2143; found, 466.2142.

Benzyl 6-(4-cyanophenyl)-6-(4-methoxyphenyl)-4-methyl-2-neopentylhexa-4,5-dienoate (26)

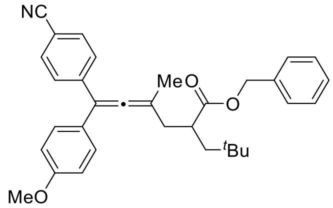

The product was prepared by **General procedure III** with 1-methoxy-4-(3-methylbut-3-en-1-yn-1-yl)benzene (**1a**, 51.7 mg, 0.30 mmol, 1.5 equiv), benzyl acrylate (146 mg, 0.90 mmol, 4.5 equiv), 4-iodobenzonitrile (**3**, 45.8 mg, 0.20 mmol, 1.0 equiv) and 2-iodo-2-methylpropane (**4**, 110 mg, 0.60 mmol, 3.0 equiv). Flash column chromatography (DCM/hexanes, 10–50%) afforded the title compound as a colorless oil (44.4 mg, 45%).

^{1}H NMR (400 MHz, acetone-d_6) δ 7.74–7.68 (m, 2H), 7.47 (d, J = 1.7 Hz, 2H), 7.36–7.25 (m, 5H), 7.25–7.19 (m, 2H), 6.94 (d, J = 8.7 Hz, 2H), 5.00–4.72 (m, 2H), 3.80 (d, J = 3.6 Hz, 3H), 2.81–2.71 (m, 1H), 2.59–2.47 (m, 1H), 2.36–2.26 (m, 1H), 1.91 (s, 3H), 1.76–1.59 (m, 1H), 1.39–1.30 (m, 1H), 0.79 (s, 5H), 0.77 (s, 4H).

^{13}C{^{1}H} NMR (100 MHz, acetone-d_6) δ 205.02, 204.97, 176.36, 176.31, 160.23, 143.98, 143.89, 137.10, 137.09, 132.97, 130.57, 130.47, 129.77, 129.64, 129.31, 129.24, 129.21, 129.20, 128.94, 128.92, 128.82, 128.80, 119.41, 114.86, 114.83, 111.13, 111.10, 109.06, 108.99, 102.46, 102.36, 66.70, 66.68, 55.63, 55.62, 46.69, 46.53, 41.08, 41.06, 40.03, 31.23, 31.18, 18.87, 18.81.
HRMS (ESI): *m/z* [M+H]$^+$ calcd for $C_{33}H_{36}NO_3^+$, 494.2690; found, 494.2685.

3-Phenoxybenzyl 6-(4-cyanophenyl)-6-(4-methoxyphenyl)-4-methyl-2-neopentylhexa-4,5-dienoate (27)

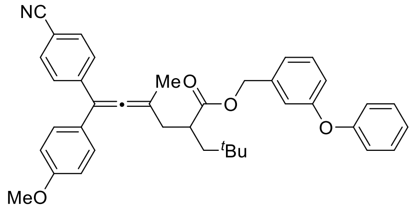

The product was prepared by **General procedure III** with 1-methoxy-4-(3-methylbut-3-en-1-yn-1-yl)benzene (**1a**, 51.7 mg, 0.30 mmol, 1.5 equiv), 3-phenoxybenzyl acrylate (229 mg, 0.90 mmol, 4.5 equiv), 4-iodobenzonitrile (**3**, 45.8 mg, 0.20 mmol, 1.0 equiv) and 2-iodo-2-methylpropane (**4**, 110 mg, 0.60 mmol, 3.0 equiv). Flash column chromatography (DCM/hexanes, 10–50%) afforded the title compound as a colorless oil (94.9 mg, 81%).
^{1}H NMR (400 MHz, acetone-d_6) δ 7.74–7.60 (m, 2H), 7.49–7.41 (m, 2H), 7.40–7.30 (m, 3H), 7.23–7.16 (m, 2H), 7.15–7.10 (m, 1H), 7.07–6.97 (m, 3H), 6.97–6.85 (m, 4H), 5.06–4.59 (m, 2H), 3.79 (d, *J* = 1.5 Hz, 3H), 2.78–2.67 (m, 1H), 2.59–2.45 (m, 1H), 2.34–2.26 (m, 1H), 1.89 (s, 3H), 1.74–1.59 (m, 1H), 1.37–1.30 (m, 1H), 0.78 (s, 5.1H), 0.76 (s, 3.9H).
^{13}C{^{1}H} NMR (100 MHz, acetone-d_6) δ 204.93, 204.90, 176.25, 176.19, 160.21, 158.33, 157.88, 143.92, 143.85, 139.24, 139.21, 132.95, 130.77, 130.74, 130.57, 130.43, 129.75, 129.59, 129.27, 129.21, 124.36, 123.57, 123.53, 119.75, 119.40, 119.01, 118.69, 118.64, 114.84, 114.82, 111.13, 111.08, 109.07, 109.02, 102.43, 102.36, 66.13, 55.62, 46.72, 46.59, 41.02, 41.00, 40.02, 31.23, 31.18, 18.85, 18.81.
HRMS (ESI): *m/z* [M+H]$^+$ calcd for $C_{39}H_{40}NO_4^+$, 586.2952; found, 586.2956.

4-Ethylphenyl 6-(4-cyanophenyl)-6-(4-methoxyphenyl)-4-methyl-2-neopentylhexa-4,5-dienoate (28)

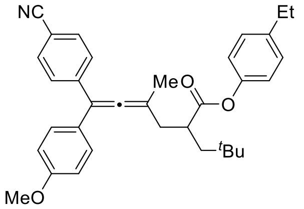

The product was prepared by **General procedure III** with 1-methoxy-4-(3-methylbut-3-en-1-yn-1-yl)benzene (**1a**, 51.7 mg, 0.30 mmol, 1.5 equiv), 4-ethylphenyl acrylate (159 mg, 0.90 mmol, 4.5 equiv),

4-iodobenzonitrile (**3**, 45.8 mg, 0.20 mmol, 1.0 equiv) and 2-iodo-2-methylpropane (**4**, 110 mg, 0.60 mmol, 3.0 equiv). Flash column chromatography (DCM/hexanes, 10–50%) afforded the title compound as a colorless oil (33.5 mg, 33%).

^{1}H NMR (400 MHz, acetone-*d*$_6$) δ 7.75–7.57 (m, 2H), 7.52–7.43 (m, 2H), 7.24 (dd, *J* = 8.9, 2.5 Hz, 2H), 7.17–7.08 (m, 2H), 6.98–6.89 (m, 2H), 6.85–6.73 (m, 2H), 3.82 (s, 3H), 3.00–2.89 (m, 1H), 2.74–2.66 (m, 1H), 2.65–2.57 (m, 2H), 2.51–2.36 (m, 1H), 2.01 (d, *J* = 3.9 Hz, 3H), 1.88–1.79 (m, 1H), 1.48–1.39 (m, 1H), 1.24–1.16 (m, 3H), 0.91 (s, 5.1H), 0.90 (s, 3.9H).

^{13}C{^{1}H} NMR (100 MHz, acetone-*d*$_6$) δ 205.15, 204.97, 175.23, 175.18, 160.29, 160.25, 149.75, 144.03, 143.71, 142.34, 142.24, 133.02, 132.94, 130.58, 130.56, 129.79, 129.78, 129.35, 129.27, 129.23, 129.13, 122.06, 122.02, 119.41, 114.91, 114.88, 111.19, 111.12, 109.23, 109.02, 102.33, 102.12, 55.63, 55.61, 46.66, 46.55, 41.08, 41.07, 40.19, 40.07, 31.42, 31.37, 29.77, 28.78, 18.88, 18.75, 16.13, 16.08.

HRMS (ESI): *m/z* [M+H]$^+$ calcd for $C_{34}H_{38}NO_3^+$, 508.2846; found, 508.2849.

Perfluorophenyl 6-(4-cyanophenyl)-6-(4-methoxyphenyl)-4-methyl-2-neopentylhexa-4,5-dienoate (29)

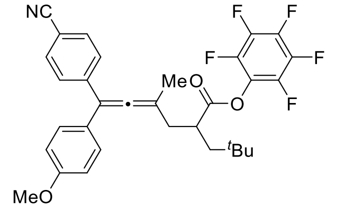

The product was prepared by **General procedure III** with 1-methoxy-4-(3-methylbut-3-en-1-yn-1-yl)benzene (**1a**, 51.7 mg, 0.30 mmol, 1.5 equiv), pentafluorophenyl acrylate (214 mg, 0.90 mmol, 4.5 equiv), 4-iodobenzonitrile (**3**, 45.8 mg, 0.20 mmol, 1.0 equiv) and 2-iodo-2-methylpropane (**4**, 110 mg, 0.60 mmol, 3.0 equiv). Flash column chromatography (DCM/hexanes, 10–50%) afforded the title compound as a yellow oil (86.4 mg, 76%).

^{1}H NMR (400 MHz, acetone-*d*$_6$) δ 7.75–7.66 (m, 2H), 7.48 (dd, *J* = 8.6, 7.0 Hz, 2H), 7.27–7.15 (m, 2H), 6.95–6.85 (m, 2H), 3.80 (d, *J* = 1.3 Hz, 3H), 3.20–3.05 (m, 1H), 2.75–2.65 (m, 1H), 2.65–2.52 (m, 1H), 2.01 (d, *J* = 1.9 Hz, 3H), 1.91–1.82 (m, 1H), 1.55–1.47 (m, 1H), 0.89 (s, 5.1H), 0.86 (s, 3.9H).

^{13}C{^{1}H} NMR (150 MHz, acetone-*d*$_6$) δ 204.38, 204.26, 172.18, 172.02, 159.40, 159.37, 143.05, 142.78, 142.47–141.50 (m), 140.72–139.94 (m), 139.30–138.27 (m), 137.24–136.58 (m), 132.08, 132.04, 129.73, 129.67, 129.62, 128.95, 128.87, 128.82, 128.39, 128.07, 125.40–124.66 (m), 118.44, 113.90, 113.79, 110.38, 110.36, 108.65, 108.59, 100.77, 100.72, 54.72, 54.69, 54.08, 45.17, 45.00, 39.65, 39.60, 39.43, 39.37, 30.37, 30.31, 28.65, 28.62, 17.92, 17.86.

^{19}F{^{1}H} NMR (377 MHz, acetone-*d*$_6$) δ -153.41 – -153.76 (m), -160.28 – -160.75 (m), -164.50 – -165.28 (m).

HRMS (ESI): *m/z* [M+H]$^+$ calcd for $C_{32}H_{29}F_5NO_3^+$, 570.2062; found, 570.2078.

4-(1-(1,3-Dioxoisoindolin-2-yl)-8-(ethylsulfonyl)-6,10,10-trimethylundeca-4,5-dien-4-yl)benzonitrile (30)

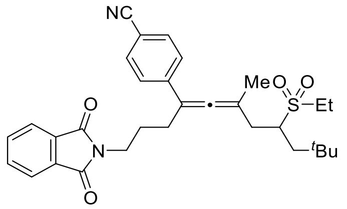

The product was prepared by **General procedure III** with **S29** (76.0 mg, 0.30 mmol, 1.5 equiv), (ethylsulfonyl)ethene (108 mg, 0.90 mmol, 4.5 equiv), 4-iodobenzonitrile (**3**, 45.8 mg, 0.20 mmol, 1.0 equiv) and 2-iodo-2-methylpropane (**4**, 110 mg, 0.60 mmol, 3.0 equiv). Flash column chromatography (ethyl acetate/hexanes, 0–40%) afforded the title compound as a pale-yellow oil (38.6 mg, 36%).

^{1}H NMR (400 MHz, acetone-d_6) δ 7.87–7.81 (m, 4H), 7.72–7.60 (m, 4H), 3.84–3.68 (m, 2H), 3.10–3.04 (m, 1H), 2.97–2.87 (m, 1H), 2.78–2.72 (m, 1H), 2.69–2.55 (m, 2H), 2.53–2.45 (m, 1H), 2.00–1.93 (m, 5H), 1.93–1.87 (m, 1H), 1.50–1.39 (m, 1H), 1.24 (t, *J* = 7.4 Hz, 1H), 1.07 (t, *J* = 7.4 Hz, 2H), 0.92 (s, 7H), 0.82 (s, 2H).

^{13}C{^{1}H} NMR (100 MHz, acetone-d_6) δ 203.93, 203.52, 168.83, 168.79, 143.51, 143.17, 134.98, 134.94, 133.21, 133.18, 132.94, 132.89, 128.07, 127.97, 123.71, 123.69, 119.48, 110.84, 107.43, 106.87, 104.64, 103.94, 57.61, 57.54, 44.77, 44.40, 42.87, 42.84, 38.21, 38.18, 36.22, 35.84, 31.65, 31.51, 28.30, 28.20, 28.03, 27.75, 19.64, 19.15, 5.92.

HRMS (ESI): *m/z* $[M+H]^+$ calcd for $C_{31}H_{37}N_2O_4S^+$, 533.2469; found, 533.2468.

1,1,1,3,3,3-Hexafluoropropan-2-yl 6-(4-cyanophenyl)-6-(4-methoxyphenyl)-4-methyl-2-neopentylhexa-4,5-dienoate (5ae)

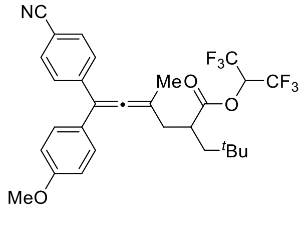

The product was prepared by **General procedure II** with 1-methoxy-4-(3-methylbut-3-en-1-yn-1-yl)benzene (**1a**, 51.7 mg, 0.30 mmol, 1.5 equiv), 1,1,1,3,3,3-hexafluoroisopropyl acrylate (**2e**, 200 mg, 0.90 mmol, 4.5 equiv), 4-bromobenzonitrile (36.4 mg, 0.20 mmol, 1.0 equiv) and 2-iodo-2-methylpropane (**4**, 110 mg, 0.60 mmol, 3.0 equiv). The reaction time was 2 h. Flash column chromatography (DCM/hexanes, 10–50%) afforded the title compound as a pale-yellow oil (72.0 mg, 65%).

1,1,1,3,3,3-Hexafluoropropan-2-yl 6-(3-cyanophenyl)-6-(4-methoxyphenyl)-4-methyl-2-neopentylhexa-4,5-dienoate (31)

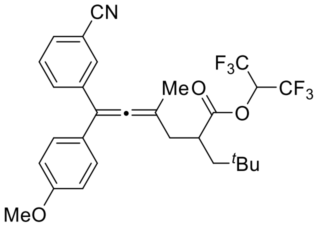

The product was prepared by **General procedure II** with 1-methoxy-4-(3-methylbut-3-en-1-yn-1-yl)benzene (**1a**, 51.7 mg, 0.30 mmol, 1.5 equiv), 1,1,1,3,3,3-hexafluoroisopropyl acrylate (**2e**, 200 mg, 0.90 mmol, 4.5 equiv), 3-iodobenzonitrile (45.8 mg, 0.20 mmol, 1.0 equiv) and 2-iodo-2-methylpropane (**4**, 110 mg, 0.60 mmol, 3.0 equiv). The reaction time was 2 h. Flash column chromatography (DCM/hexanes, 10–50%) afforded the title compound as a pale-yellow oil (78.6 mg, 71%).

^{1}H NMR (400 MHz, acetone-d_6) δ 7.75–7.54 (m, 4H), 7.29–7.15 (m, 2H), 7.03–6.92 (m, 2H), 6.33–6.18 (m, 1H), 3.82 (d, J = 2.7 Hz, 3H), 2.99–2.86 (m, 1H), 2.64–2.55 (m, 1H), 2.49–2.38 (m, 1H), 2.05 (quint, J = 2.2 Hz, 3H), 1.80–1.67 (m, 1H), 1.54–1.38 (m, 1H), 0.78 (s, 4.1H), 0.77 (s, 4.9H).

^{13}C{^{1}H} NMR (100 MHz, acetone-d_6) δ 204.01, 203.92, 173.03, 172.93, 159.49, 159.47, 139.54, 139.26, 132.66, 132.53, 131.51, 131.42, 130.67, 130.56, 129.56, 129.54, 129.45, 128.36, 128.13, 120.87 (q, J = 281.9 Hz), 118.34, 118.30, 114.08, 114.02, 112.56, 112.48, 107.97, 107.84, 100.68, 100.60, 66.36 (sept, J = 34.2 Hz), 54.76, 54.74, 44.27, 44.25, 39.40, 39.37, 38.74, 38.71, 30.17, 30.16, 28.52, 28.49, 17.83, 17.76.

^{19}F{^{1}H} NMR (377 MHz, acetone-d_6) δ -73.61 – -73.71 (m).

HRMS (ESI): *m/z* $[M+H]^+$ calcd for $C_{29}H_{30}F_6NO_3^+$, 554.2124; found, 554.2128.

Methyl 4-(5-(((1,1,1,3,3,3-hexafluoropropan-2-yl)oxy)carbonyl)-1-(4-methoxyphenyl)-3,7,7-trimethylocta-1,2-dien-1-yl)benzoate (32)

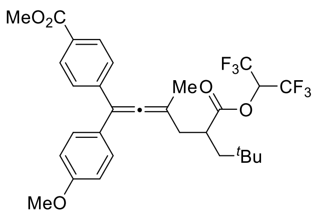

The product was prepared by **General procedure II** with 1-methoxy-4-(3-methylbut-3-en-1-yn-1-yl)benzene (**1a**, 51.7 mg, 0.30 mmol, 1.5 equiv), 1,1,1,3,3,3-hexafluoroisopropyl acrylate (**2e**, 200 mg, 0.90 mmol, 4.5 equiv), methyl 4-iodobenzoate (52.4 mg, 0.20 mmol, 1.0 equiv) and 2-iodo-2-methylpropane (**4**, 110 mg, 0.60 mmol, 3.0 equiv). The reaction time was 2 h. Flash column chromatography (DCM/hexanes, 10–50%) afforded the title compound as a pale-yellow oil (91.5 mg, 78%).

^{1}H NMR (400 MHz, acetone-d_6) δ 8.03–7.95 (m, 2H), 7.47–7.40 (m, 2H), 7.29–7.19 (m, 2H), 7.01–6.93 (m, 2H), 6.34–6.21 (m, 1H), 3.88 (d, J = 2.0 Hz, 3H), 3.82 (d, J = 2.9 Hz, 3H), 2.96–2.87 (m, 1H), 2.64–2.52 (m, 1H), 2.45–2.34 (m, 1H), 1.94 (d, J = 1.2 Hz, 3H), 1.78–1.65 (m, 1H), 1.51–1.40 (m, 1H), 0.83 (d, J = 6.0 Hz, 1.7H), 0.76 (d, J = 8.7 Hz, 7.3H).
^{13}C{^{1}H} NMR (100 MHz, acetone-d_6) δ 205.22, 205.09, 173.91, 173.88, 173.79, 172.31, 172.29, 166.98, 166.97, 160.30, 160.28, 143.70, 143.40, 130.58, 130.55, 130.53, 130.49, 130.33, 130.31, 130.28, 129.80, 129.71, 129.69, 129.42, 129.19, 129.14, 129.07, 121.78 (q, J = 281.2 Hz), 114.87, 114.81, 109.83, 109.75, 101.03, 100.94, 67.27 (sept, J = 34.1 Hz), 55.64, 52.32, 52.30, 47.57, 45.13, 45.03, 41.79, 40.33, 40.30, 40.00, 39.63, 39.58, 37.19, 31.08, 31.03, 29.41, 29.39, 18.76, 18.72, 18.15, 18.12.
^{19}F{^{1}H} NMR (377 MHz, acetone-d_6) δ -73.58 – -73.81 (m).
HRMS (ESI): m/z [M+H]$^+$ calcd for $C_{30}H_{33}F_6O_5^+$, 587.2227; found, 587.2230.

Benzyl 4-(5-(((1,1,1,3,3,3-hexafluoropropan-2-yl)oxy)carbonyl)-1-(4-methoxyphenyl)-3,7,7-trimethylocta-1,2-dien-1-yl)benzoate (33)

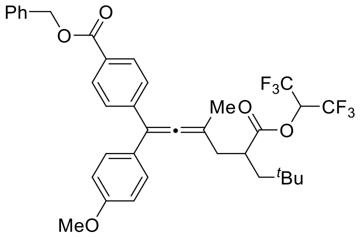

The product was prepared by **General procedure II** with 1-methoxy-4-(3-methylbut-3-en-1-yn-1-yl)benzene (**1a**, 51.7 mg, 0.30 mmol, 1.5 equiv), 1,1,1,3,3,3-hexafluoroisopropyl acrylate (**2e**, 200 mg, 0.90 mmol, 4.5 equiv), benzyl 4-iodobenzoate (67.6 mg, 0.20 mmol, 1.0 equiv), and 2-iodo-2-methylpropane (**4**, 110 mg, 0.60 mmol, 3.0 equiv). The reaction time was 2 h. Flash column chromatography (DCM/hexanes, 10–50%) afforded the title compound as a colorless oil (101 mg, 76%).
^{1}H NMR (400 MHz, acetone-d_6) δ 8.11–8.00 (m, 2H), 7.52–7.48 (m, 2H), 7.48–7.43 (m, 2H), 7.43–7.38 (m, 2H), 7.37–7.32 (m, 1H), 7.29–7.21 (m, 2H), 7.02–6.93 (m, 1H), 5.39 (d, J = 2.9 Hz, 2H), 3.81 (d, J = 3.0 Hz, 3H), 2.98–2.85 (m, 1H), 2.63–2.53 (m, 1H), 2.47–2.35 (m, 1H), 1.94 (d, J = 1.2 Hz, 3H), 1.80–1.67 (m, 1H), 1.53–1.41 (m, 1H), 0.77 (s, 4.4H), 0.76 (s, 4.6H).
^{13}C{^{1}H} NMR (100 MHz, acetone-d_6) δ 205.26, 205.11, 173.90, 166.35, 160.26, 143.86, 143.55, 137.50, 130.55, 130.46, 130.43, 130.40, 129.78, 129.69, 129.64, 129.37, 129.25, 129.13, 128.93, 128.91, 128.87, 121.78 (d, J = 282.2 Hz), 114.85, 114.79, 109.84, 109.74, 101.03, 100.92, 67.25 (sept, J = 34.2 Hz), 67.05, 55.63, 55.61, 45.12, 45.02, 40.33, 40.29, 39.62, 39.58, 31.07, 31.02, 29.45, 29.40, 18.75, 18.70.
^{19}F{^{1}H} NMR (377 MHz, acetone-d_6) δ -73.56 – -73.65 (m).
HRMS (ESI): m/z [M+H]$^+$ calcd for $C_{36}H_{37}F_6O_5^+$, 663.2540; found, 663.2541.

1,1,1,3,3,3-Hexafluoropropan-2-yl 6-(4-acetylphenyl)-6-(4-methoxyphenyl)-4-methyl-2-neopentylhexa-4,5-dienoate (34)

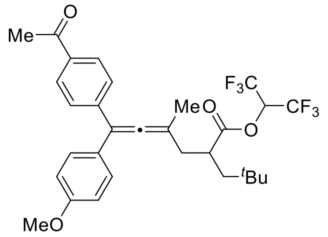

The product was prepared by **General procedure II** with 1-methoxy-4-(3-methylbut-3-en-1-yn-1-yl)benzene (**1a**, 51.7 mg, 0.30 mmol, 1.5 equiv), 1,1,1,3,3,3-hexafluoroisopropyl acrylate (**2e**, 200 mg, 0.90 mmol, 4.5 equiv), 4′-iodoacetophenone (49.2 mg, 0.20 mmol, 1.0 equiv), and 2-iodo-2-methylpropane (**4**, 110 mg, 0.60 mmol, 3.0 equiv). The reaction time was 2 h. Flash column chromatography (DCM/hexanes, 10–50%) afforded the title compound as a pale-yellow oil (63.9 mg, 56%).

^{1}H NMR (400 MHz, acetone-*d*$_6$) δ 8.03–7.92 (m, 2H), 7.49–7.40 (m, 2H), 7.28–7.16 (m, 2H), 6.99–6.89 (m, 2H), 6.34–6.17 (m, 1H), 3.82 (d, J = 2.8 Hz, 3H), 2.97–2.84 (m, 1H), 2.58 (d, J = 2.8 Hz, 4H), 2.46–2.34 (m, 1H), 1.95 (s, 3H), 1.79–1.63 (m, 1H), 1.55–1.41 (m, 1H), 0.77 (d, J = 1.4 Hz, 2.8H), 0.76 (d, J = 1.6 Hz, 6.2H).

^{13}C{^{1}H} NMR (100 MHz, acetone-*d*$_6$) δ 202.35, 202.16, 194.34, 170.97, 170.95, 157.35, 157.33, 140.60, 140.31, 134.02, 133.94, 127.62, 127.53, 126.75, 126.47, 126.29, 126.27, 126.17, 118.79 (q, J = 280.1 Hz), 111.92, 111.86, 106.96, 106.82, 98.06, 97.93, 64.33 (sept, J = 67.9, 33.9 Hz), 52.71, 52.69, 42.17, 42.08, 37.41, 37.37, 36.67, 28.14, 28.10, 26.47, 26.46, 15.83, 15.76.

^{19}F{^{1}H} NMR (377 MHz, acetone-*d*$_6$) δ -73.59 – -73.68 (m).

HRMS (ESI): *m/z* [M+H]$^+$ calcd for $C_{30}H_{33}F_6O_4^+$, 571.2278; found, 571.2273.

1,1,1,3,3,3-Hexafluoropropan-2-yl 6-(3-(dimethylcarbamoyl)phenyl)-6-(4-methoxyphenyl)-4-methyl-2-neopentylhexa-4,5-dienoate (35)

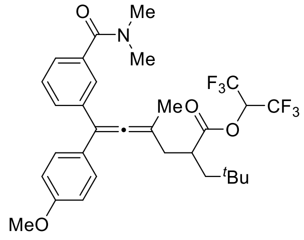

The product was prepared by **General procedure II** with 1-methoxy-4-(3-methylbut-3-en-1-yn-1-yl)benzene (**1a**, 51.7 mg, 0.30 mmol, 1.5 equiv), 1,1,1,3,3,3-hexafluoroisopropyl acrylate (**2e**, 200 mg, 0.90 mmol, 4.5 equiv), 3-iodo-*N,N*-dimethylbenzamide (55.0 mg, 0.20 mmol, 1.0 equiv) and 2-iodo-2-methylpropane (**4**, 110 mg, 0.60 mmol, 3.0 equiv). The reaction time was 2 h. Flash column

chromatography (ethyl acetate/hexanes, 0–40%) afforded the title compound as a colorless oil (102 mg, 85%).

^{1}H NMR (400 MHz, acetone-d_6) δ 7.45–7.40 (m, 1H), 7.39–7.34 (m, 2H), 7.34–7.30 (m, 1H), 7.25 (dd, *J* = 8.9, 6.3 Hz, 2H), 7.28–7.23 (m, 2H), 6.49–6.23 (m, 1H), 3.81 (d, *J* = 2.3 Hz, 3H), 3.00 (s, 6H), 2.94–2.88 (m, 1H), 2.62–2.51 (m, 1H), 2.45–2.34 (m, 1H), 1.93 (s, 3H), 1.78–1.66 (m, 1H), 1.53–1.38 (m, 1H), 0.78 (s, 4.9H), 0.77 (s, 4.1H).

^{13}C{^{1}H} NMR (100 MHz, acetone-d_6) δ 204.60, 204.44, 173.98, 173.88, 171.10, 160.18, 138.74, 138.57, 138.19, 138.15, 130.47, 130.41, 130.04, 129.96, 129.76, 129.13, 128.97, 127.81, 126.78, 126.56, 121.78 (q, *J* = 281.1 Hz), 115.48, 114.79, 114.75, 109.88, 109.66, 100.68, 100.49, 67.29 (sept, *J* = 33.9 Hz), 55.61, 45.39, 45.08, 40.34, 40.30, 39.97, 39.67, 39.48, 35.05, 31.07, 29.43, 18.90, 18.69.

^{19}F{^{1}H} NMR (377 MHz, acetone-d_6) δ -73.50 – -73.71 (m).

HRMS (ESI): *m/z* [M+H]$^+$ calcd for $C_{31}H_{36}F_6NO_4^+$, 600.2543; found, 600.2541.

1,1,1,3,3,3-Hexafluoropropan-2-yl 6-(3-cyano-4-methoxyphenyl)-6-(4-methoxyphenyl)-4-methyl-2-neopentylhexa-4,5-dienoate (36)

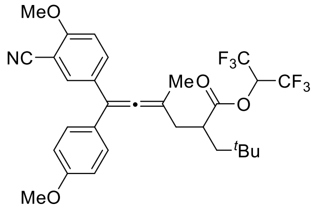

The product was prepared by **General procedure II** with 1-methoxy-4-(3-methylbut-3-en-1-yn-1-yl)benzene (**1a**, 51.7 mg, 0.30 mmol, 1.5 equiv), 1,1,1,3,3,3-hexafluoroisopropyl acrylate (**2e**, 200 mg, 0.90 mmol, 4.5 equiv), 5-iodo-2-methoxybenzonitrile (51.8 mg, 0.20 mmol, 1.0 equiv), and 2-iodo-2-methylpropane (**4**, 110 mg, 0.60 mmol, 3.0 equiv). The reaction time was 2 h. Flash column chromatography (DCM/hexanes, 10–50%) afforded the title compound as a pale-yellow oil (95.7 mg, 82%).

^{1}H NMR (400 MHz, acetone-d_6) δ 7.58–7.53 (m, 2H), 7.26–7.20 (m, 3H), 6.96 (dd, *J* = 8.8, 3.8 Hz, 2H), 6.32–6.21 (m, 1H), 3.98 (d, *J* = 2.4 Hz, 3H), 3.82 (d, *J* = 2.6 Hz, 3H), 2.98–2.86 (m, 1H), 2.62–2.52 (m, 1H), 2.46–2.33 (m, 1H), 1.93 (s, 3H), 1.77–1.64 (m, 1H), 1.49–1.41 (m, 1H), 0.79 (s, 4.5H), 0.78 (s, 4.5H).

^{13}C{^{1}H} NMR (100 MHz, acetone-d_6) δ 204.32, 204.24, 173.98, 173.86, 161.29, 161.24, 160.30, 160.28, 135.26, 135.18, 133.87, 133.81, 131.72, 131.46, 130.35, 130.26, 129.64, 129.39, 121.76 (q, *J* = 276.1 Hz), 116.72, 116.67, 114.89, 114.84, 112.88, 112.83, 108.46, 108.32, 102.43, 102.35, 101.14, 101.06, 67.23 (sept, *J* = 34.2 Hz), 56.83, 55.64, 55.62, 45.16, 45.09, 40.33, 40.28, 39.78, 39.67, 31.06, 29.42, 29.40, 18.86, 18.77.

^{19}F{^{1}H} NMR (377 MHz, acetone-d_6) δ -73.59 – -73.70 (m).

HRMS (ESI): *m/z* $[M+H]^+$ calcd for $C_{30}H_{32}F_6NO_4^+$, 584.2230; found, 584.2230.

1,1,1,3,3,3-Hexafluoropropan-2-yl 6-(4-methoxyphenyl)-4-methyl-2-neopentyl-6-(4-oxochroman-6-yl)hexa-4,5-dienoate (37)

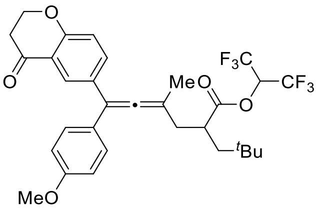

The product was prepared by **General procedure II** with 1-methoxy-4-(3-methylbut-3-en-1-yn-1-yl)benzene (**1a**, 51.7 mg, 0.30 mmol, 1.5 equiv), 1,1,1,3,3,3-hexafluoroisopropyl acrylate (**2e**, 200 mg, 0.90 mmol, 4.5 equiv), 6-iodochroman-4-one (54.8 mg, 0.20 mmol, 1.0 equiv) and 2-iodo-2-methylpropane (**4**, 110 mg, 0.60 mmol, 3.0 equiv). The reaction time was 2 h. Flash column chromatography (ethyl acetate/hexanes, 0–20%) afforded the title compound as a yellow oil (76.6 mg, 64%).

^{1}H NMR (400 MHz, acetone-d_6) δ 7.74 (dd, J = 4.6, 2.4 Hz, 1H), 7.48 (ddd, J = 8.6, 6.2, 2.4 Hz, 1H), 7.28–7.19 (m, 1H), 7.01 (dd, J = 8.6, 3.3 Hz, 1H), 6.97–6.91 (m, 2H), 6.40–6.21 (m, 1H), 4.59 (ddd, J = 6.9, 5.9, 2.0 Hz, 2H), 3.81 (d, J = 2.8 Hz, 3H), 2.96–2.86 (m, 1H), 2.86–2.74 (m, 2H), 2.59–2.51 (m, 1H), 2.43–2.34 (m, 1H), 1.92 (s, 3H), 1.78–1.67 (m, 1H), 1.51–1.41 (m, 1H), 0.78 (s, 4.6H), 0.77 (s, 4.4H).

^{13}C{^{1}H} NMR (100 MHz, acetone-d_6) δ 204.29, 191.72, 191.53, 173.97, 173.93, 162.04, 162.02, 160.21, 160.19, 136.49, 136.42, 131.67, 131.38, 130.39, 130.27, 130.09, 129.78, 126.85, 126.69, 122.06, 122.00, 121.79 (q, J = 281.3 Hz), 118.87, 118.84, 114.81, 114.77, 109.19, 109.07, 100.55, 100.52, 68.04, 67.24 (sept, J = 33.7 Hz), 55.61, 45.20, 44.96, 40.33, 40.30, 39.94, 39.79, 38.28, 38.26, 35.08, 31.07, 31.04, 29.41, 18.91, 18.85.

^{19}F{^{1}H} NMR (377 MHz, acetone-d_6) δ -73.57 – -73.69 (m).

HRMS (ESI): *m/z* $[M+H]^+$ calcd for $C_{31}H_{33}F_6O_5^+$, 599.2227; found, 599.2228.

1,1,1,3,3,3-Hexafluoropropan-2-yl 6-(4-methoxyphenyl)-4-methyl-2-neopentyl-6-(3-oxo-1,3-dihydroisobenzofuran-5-yl)hexa-4,5-dienoate (38)

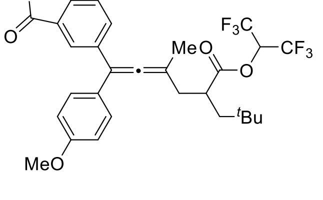

The product was prepared by **General procedure II** with 1-methoxy-4-(3-methylbut-3-en-1-yn-1-yl)benzene (**1a**, 51.7 mg, 0.30 mmol, 1.5 equiv), 1,1,1,3,3,3-hexafluoroisopropyl acrylate (**2e**, 200 mg,

0.90 mmol, 4.5 equiv), 6-iodoisobenzofuran-1(3*H*)-one (52.0 mg, 0.20 mmol, 1.0 equiv), and 2-iodo-2-methylpropane (**4**, 110 mg, 0.60 mmol, 3.0 equiv). The reaction time was 2 h. Flash column chromatography (ethyl acetate/hexanes, 0–20%) afforded the title compound as a colorless oil (93.5 mg, 80%).

^{1}H NMR (400 MHz, acetone-*d*$_6$) δ 7.78–7.62 (m, 3H), 7.26 (dd, *J* = 8.9, 8.2 Hz, 2H), 6.97 (dd, *J* = 8.9, 4.4 Hz, 2H), 6.40–6.20 (m, 1H), 5.41 (d, *J* = 1.1 Hz, 2H), 3.82 (d, *J* = 3.0 Hz, 3H), 3.02–2.88 (m, 1H), 2.65–2.55 (m, 1H), 2.50–2.34 (m, 1H), 1.96 (s, 3H), 1.81–1.64 (m, 1H), 1.55–1.41 (m, 1H), 0.78 (s, 4.5H), 0.77 (s, 4.5H).

^{13}C{^{1}H} NMR (100 MHz, acetone-*d*$_6$) δ 204.90, 204.74, 174.00, 173.85, 171.15, 170.99, 160.33, 160.31, 147.13, 147.03, 140.09, 139.80, 134.94, 134.76, 130.48, 130.36, 129.72, 129.47, 126.99, 126.94, 125.02, 124.93, 123.68, 123.61, 121.74 (q, *J* = 281.7 Hz), 114.93, 114.87, 109.31, 109.05, 101.17, 101.01, 70.48, 70.43, 67.25 (sept, *J* = 34.1 Hz), 55.64, 55.63, 45.35, 45.11, 40.32, 40.25, 39.87, 39.65, 31.08, 31.06, 29.40, 18.80, 18.60.

^{19}F{^{1}H} NMR (377 MHz, acetone-*d*$_6$) δ -73.56 – -73.68 (m).

HRMS (ESI): *m/z* [M+H]$^+$ calcd for $C_{30}H_{31}F_6O_5^+$, 585.2070; found, 585.2068.

1,1,1,3,3,3-Hexafluoropropan-2-yl 6-(4-methoxyphenyl)-4-methyl-2-neopentyl-6-(3-oxo-2,3-dihydro-1H-inden-5-yl)hexa-4,5-dienoate (39)

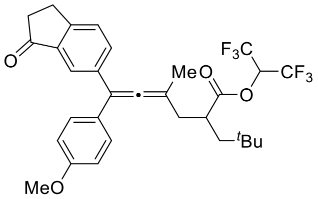

The product was prepared by **General procedure II** with 1-methoxy-4-(3-methylbut-3-en-1-yn-1-yl)benzene (**1a**, 51.7 mg, 0.30 mmol, 1.5 equiv), 1,1,1,3,3,3-hexafluoroisopropyl acrylate (**2e**, 200 mg, 0.90 mmol, 4.5 equiv), 6-iodo-1-indanone (51.6 mg, 0.20 mmol, 1.0 equiv) and 2-iodo-2-methylpropane (**4**, 110 mg, 0.60 mmol, 3.0 equiv). The reaction time was 2 h. Flash column chromatography (ethyl acetate/hexanes, 0–20%) afforded the title compound as a pale-yellow oil (67.6 mg, 58%).

^{1}H NMR (400 MHz, acetone-*d*$_6$) δ 7.63–7.51 (m, 3H), 7.24 (dd, *J* = 8.9, 6.9 Hz, 2H), 7.00–6.91 (m, 2H), 6.45–6.21 (m, 1H), 3.82 (d, *J* = 3.0 Hz, 3H), 3.21–3.11 (m, 2H), 3.00–2.88 (m, 1H), 2.68–2.62 (m, 2H), 2.60–2.53 (m, 1H), 2.45–2.35 (m, 1H), 1.94 (s, 3H), 1.78–1.66 (m, 1H), 1.54–1.39 (m, 1H), 0.78 (s, 4.8H), 0.76 (s, 4.2H).

^{13}C{^{1}H} NMR (100 MHz, acetone-*d*$_6$) δ 206.10, 204.71, 204.62, 174.01, 173.91, 160.23, 160.22, 155.18, 155.12, 138.30, 138.25, 138.12, 137.80, 135.39, 135.23, 130.46, 130.34, 130.00, 129.72, 127.75, 127.68, 123.18, 123.11, 121.78 (q, *J* = 283.3 Hz), 114.85, 114.80, 109.54, 109.31, 100.69, 100.58, 67.26 (sept,

J = 34.2 Hz), 55.62, 45.34, 45.00, 40.33, 40.27, 39.97, 39.74, 36.95, 36.94, 31.08, 31.04, 29.41, 29.39, 26.10, 18.83, 18.67.

^{19}F{^{1}H} NMR (377 MHz, acetone-d_6) δ -73.54–73.67 (m).

HRMS (ESI): *m/z* [M+H]$^+$ calcd for $C_{31}H_{33}F_6O_4^+$, 583.2278; found, 583.2279.

1,1,1,3,3,3-Hexafluoropropan-2-yl 6-(4-methoxyphenyl)-4-methyl-2-neopentyl-6-(4-sulfamoylphenyl)hexa-4,5-dienoate (40)

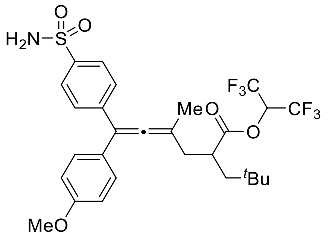

The product was prepared by **General procedure II** with 1-methoxy-4-(3-methylbut-3-en-1-yn-1-yl)benzene (**1a**, 51.7 mg, 0.30 mmol, 1.5 equiv), 1,1,1,3,3,3-hexafluoroisopropyl acrylate (**2e**, 200 mg, 0.90 mmol, 4.5 equiv), 4-iodo-benzenesulfonamide (56.6 mg, 0.20 mmol, 1.0 equiv) and 2-iodo-2-methylpropane (**4**, 110 mg, 0.60 mmol, 3.0 equiv). The reaction time was 2 h. Flash column chromatography (ethyl acetate/hexanes, 0–40%) afforded the title compound as a colorless oil (107 mg, 88%).

^{1}H NMR (400 MHz, acetone-d_6) δ 7.97–7.80 (m, 2H), 7.57–7.40 (m, 2H), 7.31–7.17 (m, 2H), 7.02–6.88 (m, 2H), 6.58 (d, J = 4.0 Hz, 2H), 6.37–6.13 (m, 1H), 3.82 (d, J = 2.8 Hz, 3H), 2.97–2.89 (m, 1H), 2.66–2.52 (m, 1H), 2.48–2.32 (m, 1H), 1.94 (s, 3H), 1.79–1.63 (m, 1H), 1.53–1.39 (m, 1H), 0.78 (s, 4.1H), 0.77 (s, 4.9H).

^{13}C{^{1}H} NMR (100 MHz, acetone-d_6) δ 205.25, 204.97, 173.89, 173.86, 160.28, 160.26, 143.75, 143.67, 142.59, 142.32, 130.50, 130.42, 129.57, 129.39, 129.30, 127.08, 127.05, 121.77 (q, J = 284.6 Hz), 114.87, 114.81, 109.53, 109.33, 101.18, 100.99, 67.23 (sept, J = 34.1 Hz), 55.64, 55.62, 45.15, 44.98, 40.31, 40.25, 39.57, 31.06, 31.01, 29.42, 29.38, 18.78, 18.66.

^{19}F{^{1}H} NMR (377 MHz, acetone-d_6) δ -73.56 – -73.66 (m).

HRMS (ESI): *m/z* [M+H]$^+$ calcd for $C_{28}H_{32}F_6NO_5S^+$, 608.1900; found, 608.1908.

1,1,1,3,3,3-Hexafluoropropan-2-yl 6-(benzo[*d*]thiazol-6-yl)-6-(4-methoxyphenyl)-4-methyl-2-neopentylhexa-4,5-dienoate (41)

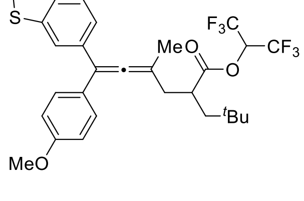

The product was prepared by **General procedure II** with 1-methoxy-4-(3-methylbut-3-en-1-yn-1-yl)benzene (**1a**, 51.7 mg, 0.30 mmol, 1.5 equiv), 1,1,1,3,3,3-hexafluoroisopropyl acrylate (**2e**, 200 mg, 0.90 mmol, 4.5 equiv), 6-iodobenzo[*d*]thiazole (52.2 mg, 0.20 mmol, 1.0 equiv) and 2-iodo-2-methylpropane (**4**, 110 mg, 0.60 mmol, 3.0 equiv). The reaction time was 2 h. Flash column chromatography (ethyl acetate/hexanes, 0–20%) afforded the title compound as a pale-yellow oil (93.7 mg, 80%).

^{1}H NMR (400 MHz, acetone-d_6) δ 9.22 (d, J = 3.8 Hz, 1H), 8.10–7.98 (m, 2H), 7.52 (dt, J = 8.5, 1.7 Hz, 1H), 7.34–7.25 (m, 2H), 7.03–6.94 (m, 2H), 6.33–6.22 (m, 1H), 3.82 (d, J = 2.9 Hz, 3H), 2.99–2.86 (m, 1H), 2.65–2.52 (m, 1H), 2.48–2.35 (m, 1H), 1.96 (s, 3H), 1.81–1.67 (m, 1H), 1.55–1.42 (m, 1H), 0.74 (s, 4.7H), 0.74 (s, 4.3H).

^{13}C{^{1}H} NMR (100 MHz, acetone-d_6) δ 204.97, 204.96, 173.99, 173.93, 160.22, 160.19, 155.66, 153.77, 153.74, 136.49, 136.18, 135.25, 135.21, 130.55, 130.51, 130.45, 130.22, 129.94, 127.73, 127.65, 123.84, 123.81, 122.39, 122.29, 121.76 (q, J = 280.3 Hz), 114.81, 114.75, 109.85, 100.48, 67.23 (sept, J = 34.2 Hz), 55.62, 55.61, 45.01, 44.97, 40.32, 40.30, 39.81, 39.78, 31.01, 30.42, 29.39, 18.84, 18.81.

^{19}F{^{1}H} NMR (377 MHz, acetone-d_6) δ -73.57 – -73.67 (m).

HRMS (ESI): *m/z* [M+H]$^+$ calcd for $C_{29}H_{30}F_6NO_3S^+$, 586.1845; found, 586.1847.

1,1,1,3,3,3-Hexafluoropropan-2-yl 6-(6-fluoropyridin-3-yl)-6-(4-methoxyphenyl)-4-methyl-2-neopentylhexa-4,5-dienoate (42)

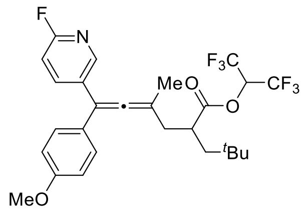

The product was prepared by **General procedure II** with 1-methoxy-4-(3-methylbut-3-en-1-yn-1-yl)benzene (**1a**, 51.7 mg, 0.30 mmol, 1.5 equiv), 1,1,1,3,3,3-hexafluoroisopropyl acrylate (**2e**, 200 mg, 0.90 mmol, 4.5 equiv), 2-fluoro-5-iodopyridine (44.6 mg, 0.20 mmol, 1.0 equiv) and 2-iodo-2-methylpropane (**4**, 110 mg, 0.60 mmol, 3.0 equiv). The reaction time was 2 h. Flash column chromatography (DCM/hexanes, 10–50%) afforded the title compound as a colorless oil (89.8 mg, 82%).

^{1}H NMR (400 MHz, acetone-d_6) δ 8.14 (dd, J = 8.8, 2.4 Hz, 1H), 7.87–7.74 (m, 1H), 7.30–7.20 (m, 2H), 7.09 (dtd, J = 8.5, 3.2, 0.7 Hz, 1H), 6.99–6.93 (m, 2H), 6.33–6.19 (m, 1H), 3.82 (d, J = 2.3 Hz, 3H), 2.97–2.88 (m, 1H), 2.63–2.53 (m, 1H), 2.50–2.37 (m, 1H), 1.95 (s, 3H), 1.79–1.66 (m, 1H), 1.51–1.40 (m, 1H), 0.79 (s, 4.1H), 0.78 (s, 4.9H).

^{13}C{^{1}H} NMR (100 MHz, acetone-d_6) δ 204.62, 204.40, 173.91, 173.79, 164.89, 164.86, 162.54, 162.51, 160.38, 160.36, 147.98, 147.93, 147.83, 147.77, 142.13, 142.08, 142.05, 142.00, 132.88, 132.83, 132.66,

132.61, 130.13, 130.03, 129.31, 129.09, 121.76 (q, J = 280.3 Hz), 114.97, 114.93, 110.07, 110.04, 109.69, 109.66, 106.41, 106.20, 101.54, 101.32, 67.25 (sept, J = 34.1 Hz), 55.65, 55.65, 45.26, 45.22, 40.37, 40.27, 39.72, 39.53, 31.09, 29.42, 29.40, 18.91, 18.71.

^{19}F{^{1}H} NMR (377 MHz, acetone-d_6) δ -72.14 (d, J = 37.2 Hz), -73.60 – -73.72 (m).

HRMS (ESI): *m/z* [M+H]$^+$ calcd for $C_{27}H_{29}F_7NO_3^+$, 548.2030; found, 548.2034.

1,1,1,3,3,3-Hexafluoropropan-2-yl 6-(4-methoxyphenyl)-4-methyl-2-neopentyl-6-(quinolin-6-yl)hexa-4,5-dienoate (43)

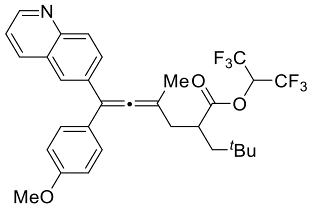

The product was prepared by **General procedure II** with 1-methoxy-4-(3-methylbut-3-en-1-yn-1-yl)benzene (**1a**, 51.7 mg, 0.30 mmol, 1.5 equiv), 1,1,1,3,3,3-hexafluoroisopropyl acrylate (**2e**, 200 mg, 0.90 mmol, 4.5 equiv), 6-iodoquinoline (51.0 mg, 0.20 mmol, 1.0 equiv) and 2-iodo-2-methylpropane (**4**, 110 mg, 0.60 mmol, 3.0 equiv). The reaction time was 2 h. Flash column chromatography (ethyl acetate/hexanes, 0–40%) afforded the title compound as a colorless oil (90.4 mg, 78%).

^{1}H NMR (400 MHz, acetone-d_6) δ 8.87 (td, J = 3.9, 1.7 Hz, 1H), 8.29–8.19 (m, 1H), 8.10–8.00 (m, 1H), 7.82–7.66 (m, 2H), 7.53–7.44 (m, 1H), 7.38–7.27 (m, 2H), 7.11–6.92 (m, 2H), 6.35–6.22 (m, 1H), 3.83 (d, J = 3.6 Hz, 3H), 3.01–2.91 (m, 1H), 2.67–2.56 (m, 1H), 2.48–2.38 (m, 1H), 1.98 (d, J = 0.7 Hz, 3H), 1.79–1.68 (m, 1H), 1.55–1.42 (m, 1H), 0.75 (s, 4.3H), 0.74 (s, 4.7H).

^{13}C{^{1}H} NMR (100 MHz, acetone-d_6) δ 205.29, 205.28, 173.98, 173.92, 160.26, 160.24, 151.21, 151.19, 148.76, 148.74, 136.85, 136.56, 136.55, 130.92, 130.83, 130.65, 130.56, 130.28, 130.26, 130.08, 129.80, 129.18, 129.15, 127.57, 127.43, 122.46, 122.44, 121.81 (q, J = 281.6 Hz), 114.85, 114.79, 109.93, 100.70, 67.24 (sept, J = 34.1 Hz), 55.64, 55.62, 45.04, 45.03, 40.33, 39.78, 39.74, 31.04, 31.02, 29.38, 18.85, 18.83.

^{19}F{^{1}H} NMR (377 MHz, acetone-d_6) δ -73.57 – -73.67 (m).

HRMS (ESI): *m/z* [M+H]$^+$ calcd for $C_{31}H_{32}F_6NO_3^+$, 580.2281; found, 580.2283.

(3*S*,8*S*,9*S*,10*R*,13*S*,14*S*,17*S*)-17-Acetyl-10,13-dimethyl-2,3,4,7,8,9,10,11,12,13,14,15,16,17-tetradecahydro-1*H*-cyclopenta[*a*]phenanthren-3-yl 4-(5-(((1,1,1,3,3,3-hexafluoropropan-2-yl)oxy)carbonyl)-1-(4-methoxyphenyl)-3,7,7-trimethylocta-1,2-dien-1-yl)benzoate (44)

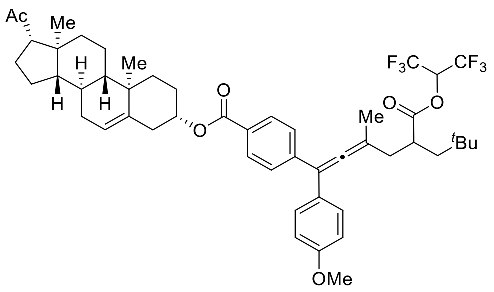

The product was prepared by **General procedure II** with 1-methoxy-4-(3-methylbut-3-en-1-yn-1-yl)benzene (**1a**, 51.7 mg, 0.30 mmol, 1.5 equiv), 1,1,1,3,3,3-hexafluoroisopropyl acrylate (**2e**, 200 mg, 0.90 mmol, 4.5 equiv), (3*S*,8*S*,9*S*,10*R*,13*S*,14*S*,17*S*)-17-acetyl-10,13-dimethyl-2,3,4,7,8,9,10,11,12,13,14,15,16,17-tetradecahydro-1*H*-cyclopenta[*a*]phenanthren-3-yl 4-iodobenzoate (**S43**, 109 mg, 0.20 mmol, 1.0 equiv) and 2-iodo-2-methylpropane (**4**, 110 mg, 0.60 mmol, 3.0 equiv). The reaction time was 2 h. Flash column chromatography (ethyl acetate/hexanes, 0–10%) afforded the title compound as a white solid (117 mg, 64%).

^{1}H NMR (400 MHz, acetone-d_6) δ 8.09–7.96 (m, 2H), 7.50–7.38 (m, 2H), 7.33–7.20 (m, 2H), 7.06–6.89 (m, 2H), 6.38–6.14 (m, 1H), 5.48–5.39 (m, 1H), 4.92–4.69 (m, 1H), 3.82 (d, J = 2.8 Hz, 3H), 2.99–2.87 (m, 1H), 2.62–2.52 (m, 2H), 2.52–2.45 (m, 2H), 2.45–2.36 (m, 1H), 2.22–2.10 (m, 2H), 2.07 (s, 3H), 2.00 (d, J = 4.6 Hz, 2H), 1.96–1.93 (m, 3H), 1.85–1.58 (m, 7H), 1.57–1.44 (m, 4H), 1.33–1.17 (m, 4H), 1.10 (s, 3H), 0.78 (s, 4.6H), 0.77 (s, 4.4H), 0.63 (s, 3H).

^{13}C{^{1}H} NMR (100 MHz, acetone-d_6) δ 208.14, 205.25, 205.08, 173.86, 173.84, 165.83, 165.81, 160.24, 160.22, 143.54, 143.25, 140.65, 130.52, 130.44, 130.33, 130.31, 130.29, 130.24, 129.62, 129.35, 129.11, 129.02, 123.16, 121.74 (d, J = 282.1 Hz), 114.82, 114.76, 109.82, 109.70, 100.93, 100.81, 75.08, 68.40–66.34 (m), 63.94, 57.48, 55.63, 55.61, 50.88, 45.12, 44.99, 44.32, 40.31, 40.25, 39.64, 39.61, 39.41, 38.94, 37.86, 37.42, 32.64, 32.52, 31.49, 31.06, 31.01, 29.42, 29.41, 28.58, 25.10, 23.41, 21.80, 19.71, 18.76, 18.69, 13.49.

^{19}F{^{1}H} NMR (377 MHz, acetone-d_6) δ -73.51 – -73.60 (m).

HRMS (ESI): *m/z* [M+H]$^+$ calcd for $C_{50}H_{61}F_6O_6^+$, 871.4367; found, 871.4376.

1,1,1,3,3,3-Hexafluoropropan-2-yl 6-(4-cyanophenyl)-2-(2,2-dimethyl-3-phenylpropyl)-6-(4-methoxyphenyl)-4-methylhexa-4,5-dienoate (45)

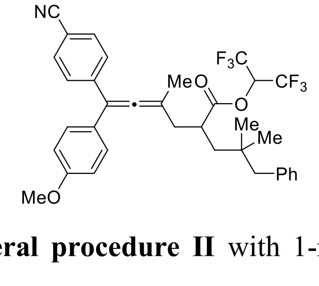

The product was prepared by **General procedure II** with 1-methoxy-4-(3-methylbut-3-en-1-yn-1-yl)benzene (**1a**, 51.7 mg, 0.30 mmol, 1.5 equiv), 1,1,1,3,3,3-hexafluoroisopropyl acrylate (**2e**, 200 mg,

0.90 mmol, 4.5 equiv), 4-iodobenzonitrile (**3**, 45.8 mg, 0.20 mmol, 1.0 equiv) and (2-iodo-2-methylpropyl)benzene (156 mg, 0.60 mmol, 3.0 equiv). The reaction time was 12 h. Flash column chromatography (DCM/hexanes, 10–50%) afforded the title compound as a colorless oil (69.3 mg, 55%).

^{1}H NMR (400 MHz, acetone-d_6) δ 7.83–7.73 (m, 2H), 7.55 (dd, *J* = 9.5, 8.4 Hz, 2H), 7.35–7.15 (m, 5H), 7.08–6.93 (m, 4H), 6.39–6.22 (m, 1H), 3.85 (d, *J* = 1.1 Hz, 3H), 3.16–2.97 (m, 1H), 2.72–2.59 (m, 1H), 2.53–2.43 (m, 1H), 2.42–2.28 (m, 2H), 2.00 (d, *J* = 2.2 Hz, 3H), 1.90–1.80 (m, 1H), 1.58–1.51 (m, 1H), 0.81–0.70 (m, 6H).

^{13}C{^{1}H} NMR (100 MHz, acetone-d_6) δ 205.58, 205.23, 173.89, 173.80, 160.42, 160.38, 143.82, 143.53, 139.14, 133.10, 133.06, 131.37, 130.68, 130.51, 129.88, 129.70, 129.25, 128.95, 128.48, 126.82, 126.81, 121.75 (q, *J* = 281.8 Hz), 119.40, 119.36, 115.01, 114.92, 111.44, 111.30, 109.65, 109.35, 101.66, 101.34, 67.26 (sept, *J* = 34.0 Hz), 55.64, 49.25, 49.22, 44.45, 44.27, 40.00, 39.87, 39.64, 39.56, 34.94, 34.84, 26.21, 26.14, 26.09, 18.85, 18.64.

^{19}F{^{1}H} NMR (377 MHz, acetone-d_6) δ -73.51 – -73.66 (m).

HRMS (ESI): *m/z* [M+H]$^+$ calcd for $C_{35}H_{34}F_6NO_3^+$, 630.2437; found, 630.2433.

1,1,1,3,3,3-Hexafluoropropan-2-yl 6-(4-cyanophenyl)-2-(2,2-dimethyl-4-phenylbutyl)-6-(4-methoxyphenyl)-4-methylhexa-4,5-dienoate (46)

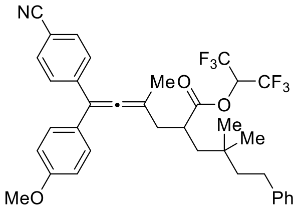

The product was prepared by **General procedure II** with 1-methoxy-4-(3-methylbut-3-en-1-yn-1-yl)benzene (**1a**, 51.7 mg, 0.30 mmol, 1.5 equiv), 1,1,1,3,3,3-hexafluoroisopropyl acrylate (**2e**, 200 mg, 0.90 mmol, 4.5 equiv), 4-iodobenzonitrile (**3**, 45.8 mg, 0.20 mmol, 1.0 equiv) and (3-iodo-3-methylbutyl)benzene (**S45**, 164 mg, 0.60 mmol, 3.0 equiv). The reaction time was 12 h. Flash column chromatography (DCM/hexanes, 10–50%) afforded the title compound as a colorless oil (97.6 mg, 76%).

^{1}H NMR (400 MHz, acetone-d_6) δ 7.76–7.69 (m, 2H), 7.52–7.45 (m, 2H), 7.30–7.19 (m, 4H), 7.17–7.14 (m, 1H), 7.13–7.09 (m, 2H), 6.98–6.90 (m, 2H), 6.37–6.22 (m, 1H), 3.82–3.71 (m, 3H), 3.07–2.89 (m, 1H), 2.69–2.54 (m, 1H), 2.51–2.47 (m, 1H), 2.45–2.35 (m, 2H), 1.96 (d, *J* = 2.8 Hz, 3H), 1.88–1.75 (m, 1H), 1.60–1.48 (m, 1H), 1.42–1.23 (m, 2H), 0.84 (dd, *J* = 7.6, 4.3 Hz, 6H).

^{13}C{^{1}H} NMR (100 MHz, acetone-d_6) δ 205.52, 205.14, 173.92, 173.83, 160.34, 143.76, 143.73, 143.50, 133.05, 133.02, 130.63, 130.46, 129.87, 129.66, 129.13, 129.11, 129.07, 129.05, 128.90, 126.38, 126.37, 121.74 (q, *J* = 282.0 Hz), 119.39, 119.34, 114.95, 114.88, 111.43, 111.28, 109.64, 109.31, 101.64,

101.30, 67.27 (sept, J = 34.1 Hz), 55.61, 55.57, 45.72, 45.65, 43.44, 43.17, 39.95, 39.83, 39.61, 39.54, 33.83, 33.74, 31.09, 26.87, 26.81, 26.55, 26.53, 18.85, 18.62.

^{19}F{^{1}H} NMR (377 MHz, acetone-d_6) δ -73.50 – -73.64 (m).

HRMS (ESI): *m/z* [M+H]$^+$ calcd for $C_{36}H_{36}F_6NO_3^+$, 644.2594; found, 644.2592.

9-(4-Cyanophenyl)-5-(((1,1,1,3,3,3-hexafluoropropan-2-yl)oxy)carbonyl)-9-(4-methoxyphenyl)-3,3,7-trimethylnona-7,8-dien-1-yl 4-methylbenzoate (47)

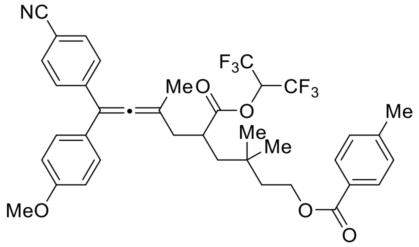

The product was prepared by **General procedure II** with 1-methoxy-4-(3-methylbut-3-en-1-yn-1-yl)benzene (**1a**, 51.7 mg, 0.30 mmol, 1.5 equiv), 1,1,1,3,3,3-hexafluoroisopropyl acrylate (**2e**, 200 mg, 0.90 mmol, 4.5 equiv), 4-iodobenzonitrile (**3**, 45.8 mg, 0.20 mmol, 1.0 equiv), 3-iodo-3-methylbutyl 4-methylbenzoate (**S46**, 199 mg, 0.60 mmol, 3.0 equiv). The reaction time was 12 h. Flash column chromatography (DCM/hexanes, 10–50%) afforded the title compound as a white solid (60.3 mg, 43%).

^{1}H NMR (400 MHz, acetone-d_6) δ 7.92–7.83 (m, 2H), 7.78–7.70 (m, 1H), 7.54–7.47 (m, 2H), 7.34–7.28 (m, 2H), 7.27–7.21 (m, 2H), 7.02–6.89 (m, 2H), 6.39–6.23 (m, 1H), 4.30–4.14 (m, 2H), 3.79 (d, J = 2.7 Hz, 3H), 3.16–2.93 (m, 1H), 2.67–2.55 (m, 1H), 2.53–2.42 (m, 1H), 2.40 (s, 3H), 1.96 (d, J = 3.0 Hz, 3H), 1.90–1.80 (m, 1H), 1.65–1.57 (m, 1H), 1.57–1.47 (m, 2H), 0.86 (dd, J = 7.5, 3.6 Hz, 6H).

^{13}C{^{1}H} NMR ^{13}C NMR (100 MHz, acetone-d_6) δ 205.56, 205.23, 173.87, 173.80, 166.62, 160.41, 160.38, 144.44, 143.76, 143.50, 133.10, 133.05, 130.64, 130.50, 130.18, 129.98, 129.87, 129.69, 129.15, 128.91, 128.69, 128.67, 121.76 (q, J = 283.0 Hz), 119.39, 119.36, 114.98, 114.91, 111.45, 111.31, 109.68, 109.37, 101.57, 101.28, 68.15–66.15 (m), 62.10, 62.08, 55.62, 44.00, 43.83, 41.01, 40.98, 39.86, 39.75, 39.64, 39.56, 33.18, 33.10, 32.65, 27.02, 26.93, 26.78, 26.76, 21.55, 18.81, 18.59.

^{19}F{^{1}H} NMR (377 MHz, acetone-d_6) δ -73.52 – -73.66 (m).

HRMS (ESI): *m/z* [M+H]$^+$ calcd for $C_{38}H_{38}F_6NO_5^+$, 702.2649; found, 702.2652.

1,1,1,3,3,3-Hexafluoropropan-2-yl 2-(4-(4-cyanophenyl)-4-(4-methoxyphenyl)-2-methylbuta-2,3-dien-1-yl)-4-ethyl-4,8-dimethylnonanoate (48)

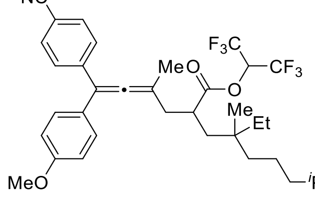

The product was prepared by **General procedure II** with 1-methoxy-4-(3-methylbut-3-en-1-yn-1-yl)benzene (**1a**, 51.7 mg, 0.30 mmol, 1.5 equiv), 1,1,1,3,3,3-hexafluoroisopropyl acrylate (**2e**, 200 mg, 0.90 mmol, 4.5 equiv), 4-iodobenzonitrile (**3**, 45.8 mg, 0.20 mmol, 1.0 equiv), 6-iodo-2,6-dimethyloctane (**S47**, 161 mg, 0.60 mmol, 3.0 equiv). The reaction time was 12 h. Flash column chromatography (DCM/hexanes, 10–50%) afforded the title compound as a colorless oil (26.1 mg, 20%).

^{1}H NMR (400 MHz, acetone-d_6) δ 7.79–7.69 (m, 2H), 7.56–7.42 (m, 2H), 7.27–7.18 (m, 2H), 7.01–6.92 (m, 2H), 6.37–6.20 (m, 1H), 3.83 (d, J = 4.1 Hz, 3H), 2.97–2.85 (m, 1H), 2.63–2.50 (m, 1H), 2.50–2.39 (m, 1H), 1.94 (d, J = 1.3 Hz, 3H), 1.79–1.69 (m, 1H), 1.47–1.40 (m, 1H), 1.18–0.98 (m, 8H), 0.84 (d, J = 6.6 Hz, 6H), 0.75–0.63 (m, 6H).

^{13}C{^{1}H} NMR (150 MHz, acetone-d_6) δ 205.61, 205.36, 205.34, 173.97, 173.88, 160.37, 160.34, 143.83, 143.63, 143.60, 133.07, 133.04, 130.63, 130.61, 130.46, 130.44, 129.90, 129.88, 129.70, 129.68, 129.13, 128.99, 128.97, 121.78 (q, J = 283.0 Hz), 119.41, 119.38, 114.93, 114.88, 111.38, 111.24, 109.36, 109.16, 109.14, 101.49, 101.24, 67.93–66.50 (m), 55.65, 41.30, 41.25, 41.15, 41.11, 40.54, 40.51, 39.82, 39.80, 39.77, 39.71, 39.67, 39.56, 39.53, 39.47, 39.44, 39.40, 39.36, 36.05, 36.03, 35.96, 31.94, 31.92, 31.81, 31.79, 28.54, 28.52, 24.26, 22.96, 22.94, 22.91, 21.74, 18.65, 18.63, 18.51, 8.15, 8.12.

^{19}F{^{1}H} NMR (377 MHz, acetone-d_6) δ -73.53 – -73.68 (m).

HRMS (ESI): m/z [M+H]$^+$ calcd for $C_{35}H_{42}F_6NO_3^+$, 638.3063; found, 638.3068.

1,1,1,3,3,3-Hexafluoropropan-2-yl 6-(4-cyanophenyl)-2-isobutyl-6-(4-methoxyphenyl)-4-methylhexa-4,5-dienoate (49)

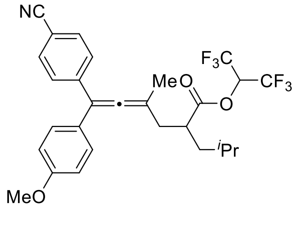

The product was prepared by **General procedure II** with 1-methoxy-4-(3-methylbut-3-en-1-yn-1-yl)benzene (**1a**, 51.7 mg, 0.30 mmol, 1.5 equiv), 1,1,1,3,3,3-hexafluoroisopropyl acrylate (**2e**, 200 mg, 0.90 mmol, 4.5 equiv), 4-iodobenzonitrile (**3**, 45.8 mg, 0.20 mmol, 1.0 equiv) and 2-iodopropane (102 mg, 0.60 mmol, 3.0 equiv). The reaction time was 12 h. Flash column chromatography (DCM/hexanes, 10–50%) afforded the title compound as a colorless oil (37.2 mg, 34%).

^{1}H NMR (400 MHz, acetone-d_6) δ 7.82–7.71 (m, 2H), 7.56–7.45 (m, 2H), 7.26–7.18 (m, 2H), 7.01–6.92 (m, 2H), 6.34–6.21 (m, 1H), 3.82 (d, J = 1.0 Hz, 3H), 3.07–2.92 (m, 1H), 2.67–2.55 (m, 1H), 2.51–2.38 (m, 1H), 1.94 (s, 3H), 1.63–1.53 (m, 1H), 1.53–1.44 (m, 1H), 1.44–1.34 (m, 1H), 0.82–0.71 (m, 6H).

$^{13}C\{^1H\}$ NMR (100 MHz, acetone-d_6) δ 204.87, 204.69, 172.92, 172.85, 160.35, 143.75, 143.63, 133.03, 130.49, 129.74, 129.72, 129.09, 128.99, 121.71 (q, J = 284.0 Hz), 119.41, 114.91, 114.88, 111.30, 111.26, 109.86, 109.66, 102.11, 101.91, 67.08 (sept, J = 34.1 Hz), 55.64, 42.42, 42.35, 41.50, 41.46, 37.54, 37.40, 26.78, 26.75, 23.16, 23.12, 21.71, 21.64, 19.05, 18.86.

$^{19}F\{^1H\}$ NMR (377 MHz, acetone-d_6) δ -73.86 – -73.88 (m).

HRMS (ESI): *m/z* $[M+H]^+$ calcd for $C_{28}H_{28}F_6NO_3^+$, 540.1968; found, 540.1970.

5. Mechanistic Studies

5.1. Control Experiments

Scheme S5. Control Experiments

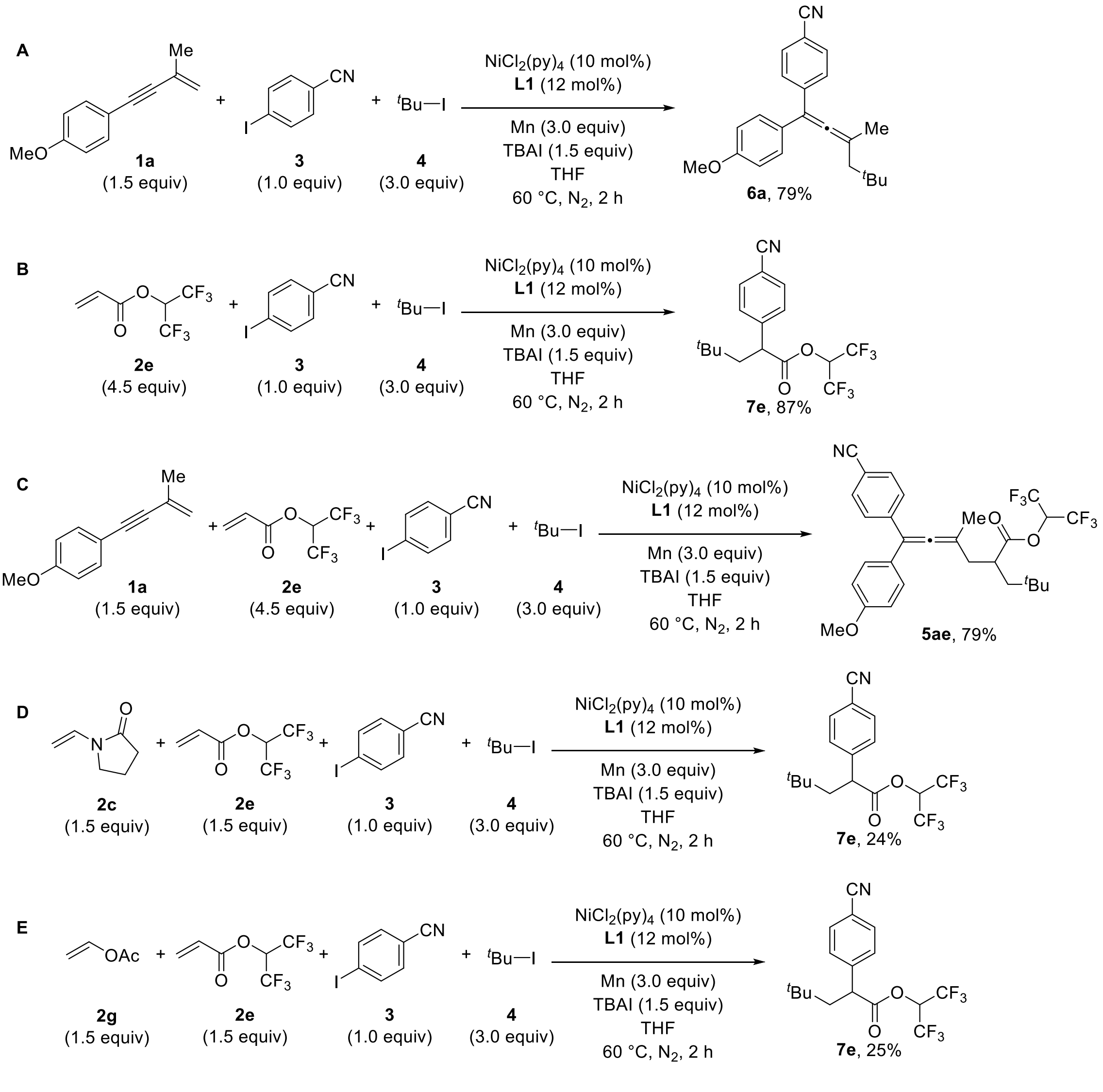

Experimental Procedure for Scheme S5: The reactions in the absence of one of the conjunctive reagents (control experiments) were conducted following **General procedure II**. The reaction using **1a** without **2e** afforded the 1,3-enyne dicarbofunctionalization product **6a** in a yield of 79%. The reaction using **2e** without **1a** gave the acrylate dicarbofunctionalization product **7e** in a yield of 87%. The

reaction using both **1a** and **2e** afforded the twofold conjunctive coupling product **5ae** in 79% yield. In comparison, the reactions with two electronically differentiated alkenes (**2c**+**2e** or **2g**+**2e**) did not produce twofold conjunctive coupling but acrylate dicarbofunctionalization in a low yield (**7e**, 24% and 25%, respectively, yields were determined by ^{1}H NMR spectroscopy using dibromomethane as an internal standard).

The reaction mixtures from **Schemes S5D** and **S5E** were monitored by TLC. No noticeable product peak with conventional R_f value was observed, except the peak of **7e**. However, we observed a peak with an extremely low R_f value by TLC. We suspect that it might be polymerized products with several alkene molecules incorporated.

It is plausible that **int2_HFIP** reacted with alkene forming a new radical intermediate, which reduced the formation of **7e**. But further polymerization was not suppressed, because the capture of the subsequent radical intermediate by nickel is slower than polymerization, resulting in a lower amount of **7e** and no noticeable product of interest.

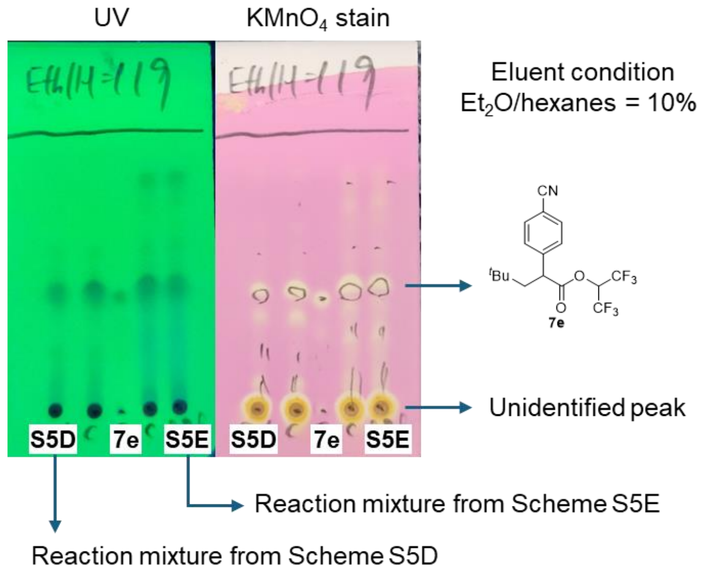

Figure S1. TLC analysis of reaction mixture from Schemes S5D and S5E

4-(1-(4-Methoxyphenyl)-3,5,5-trimethylhexa-1,2-dien-1-yl)benzonitrile (6a)

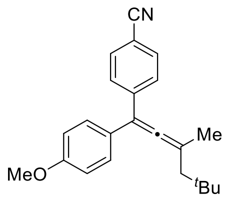

The product was prepared by **Experimental Procedure for Scheme S5A** with 1-methoxy-4-(3-methylbut-3-en-1-yn-1-yl)benzene (**1a**, 51.7 mg, 0.30 mmol, 1.5 equiv). Flash column chromatography (diethyl ether/hexanes, 0–15%) afforded the title compound as a pale-yellow oil (52.2 mg, 79%).

1,1,1,3,3,3-Hexafluoropropan-2-yl 2-(4-cyanophenyl)-4,4-dimethylpentanoate (7e)

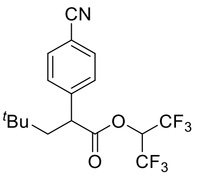

The product was prepared by **Experimental Procedure for Scheme S5B** with methyl acrylate (**2e**, 200 mg, 0.90 mmol, 4.5 equiv). Flash column chromatography (diethyl ether/hexanes, 0–15%) afforded the title compound as a colorless oil (66.6 mg, 87%).

^{1}H NMR (400 MHz, acetone-d_6) δ 7.83–7.78 (m, 2H), 7.68–7.62 (m, 2H), 6.37 (quint, J = 6.2 Hz, 1H), 4.18 (dd, J = 8.4, 4.3 Hz, 1H), 2.37 (dd, J = 14.2, 8.4 Hz, 1H), 1.76 (dd, J = 14.2, 4.3 Hz, 1H), 0.93 (s, 9H).

^{13}C{^{1}H} NMR (100 MHz, acetone-d_6) δ 171.64, 145.37, 133.54, 129.91, 121.60 (q, J = 264.1 Hz), 118.98, 112.47, 68.61–66.17 (m), 47.97, 47.19, 31.70, 29.51.

^{19}F{^{1}H} NMR (377 MHz, acetone-d_6) δ -73.80 (q, J = 8.6 Hz), -74.10 (q, J = 8.5 Hz).

HRMS (ESI): m/z $[M-H]^-$ calcd for $C_{17}H_{16}F_6NO_2^-$, 380.1091; found, 380.1089.

5.2. Effects of Substituents in the Conjunctive Platforms

Scheme S6. Effect of Substituents in 1,3-Enynes

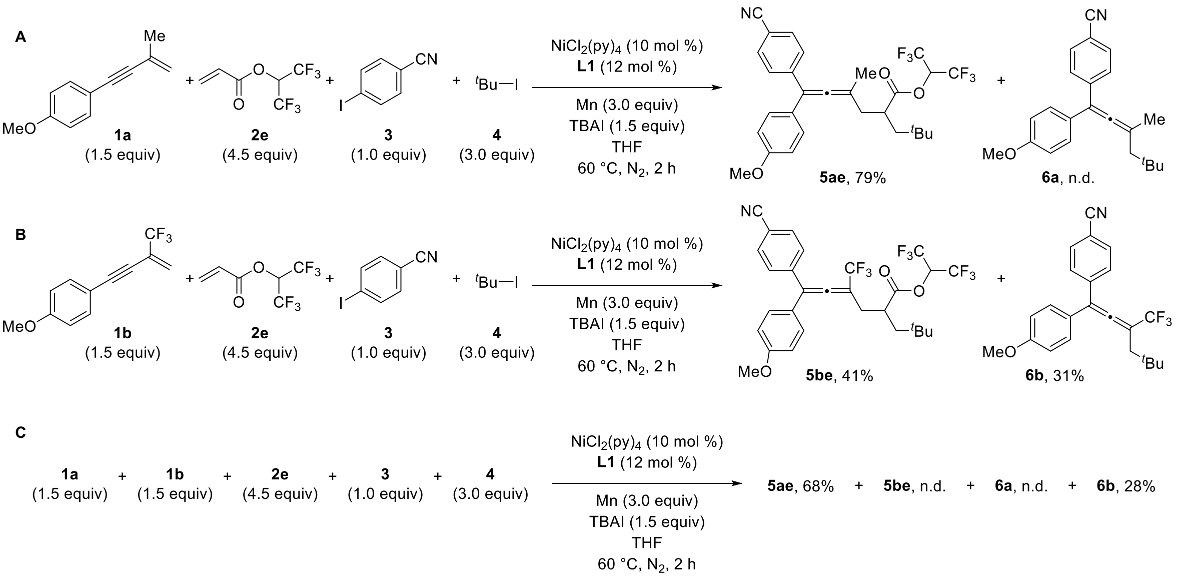

Experimental Procedure for Scheme S6A and S6B: In an N_2-filled glovebox, a flame-dried 4 mL screw-cap vial containing a magnetic stir bar was charged with $NiCl_2(py)_4$ (8.9 mg, 0.020 mmol, 10

mol%), 2,2′-bipyridine (**L1**, 3.8 mg, 0.024 mmol, 12 mol%), Mn (33 mg, 0.60 mmol, 3.0 equiv), TBAI (111 mg, 0.30 mmol, 1.5 equiv), 1,3-enyne (**1a**, 51.7 mg, 0.30 mmol, 1.5 equiv, for **Scheme S6A**; **1b**, 67.9 mg, 0.30 mmol, 1.5 equiv, for **Scheme S6B**), 1,1,1,3,3,3-hexafluoroisopropyl acrylate (**2e**, 200 mg, 0.90 mmol, 4.5 equiv), 4-iodobenzonitrile (**3**, 45.8 mg, 0.20 mmol, 1.0 equiv), 2-iodo-2-methylpropane (**4**, 110 mg, 0.60 mmol, 3.0 equiv) and anhydrous THF (1 mL). The vial was then capped and removed from the glovebox, and the mixture was stirred at 60 °C at a stirring speed of 1500 rpm. After 2 h, the mixture was concentrated *in vacuo*. The residue was purified by flash column chromatography to afford the product.

1,1,1,3,3,3-Hexafluoropropan-2-yl 6-(4-cyanophenyl)-6-(4-methoxyphenyl)-2-neopentyl-4-(trifluoromethyl)hexa-4,5-dienoate (5be)

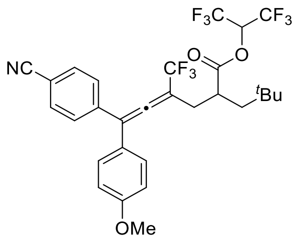

The product was prepared by **Experimental Procedure for Scheme S6B**. Flash column chromatography (DCM/hexanes, 10–50%) afforded the title compound as a colorless oil (49.8 mg, 41%).

^{1}H NMR (400 MHz, acetone-*d*$_6$) δ 7.87 (dd, *J* = 8.3, 4.0 Hz, 2H), 7.56 (dd, *J* = 8.3, 3.6 Hz, 2H), 7.35–7.17 (m, 2H), 7.05 (dd, *J* = 8.8, 3.6 Hz, 2H), 6.36–6.21 (m, 1H), 3.85 (d, *J* = 1.7 Hz, 3H), 2.94 (q, *J* = 7.9 Hz, 1H), 2.89–2.79 (m, 1H), 2.73–2.61 (m, 1H), 1.81–1.68 (m, 1H), 1.51–1.38 (m, 1H), 0.77 (s, 4.3H), 0.76 (s, 4.7H).

^{13}C{^{1}H} NMR (150 MHz, acetone-*d*$_6$) δ 205.82, 205.45, 172.90, 161.52, 140.40, 140.19, 133.56, 133.53, 130.76, 130.70, 130.27, 130.20, 125.87, 125.68, 125.27, 125.23, 123.45, 123.41, 121.69 (q, *J* = 282.2 Hz), 119.00, 117.83, 117.52, 115.43, 115.40, 113.17, 113.11, 100.31, 100.08, 100.04, 99.82, 67.98–66.61 (m), 55.79, 45.12, 45.01, 40.52, 40.50, 32.30, 32.11, 31.20, 31.16, 29.23.

^{19}F{^{1}H} NMR (377 MHz, acetone-*d*$_6$) δ -63.90 (d, *J* = 78.3 Hz), -73.65 – -73.80 (m).

HRMS (ESI): *m/z* $[M+H]^+$ calcd for $C_{29}H_{27}F_9NO_3^+$, 608.1842; found, 608.1841.

4-(1-(4-Methoxyphenyl)-5,5-dimethyl-3-(trifluoromethyl)hexa-1,2-dien-1-yl)benzonitrile (6b)

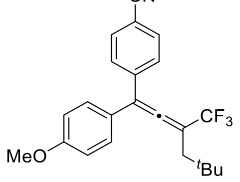

The product was prepared by **Experimental Procedure for Scheme S6B**. Flash column chromatography (DCM/hexanes, 10–50%) afforded the title compound as a colorless oil (23.9 mg, 31%).

^{1}H NMR (400 MHz, acetone-d_6) δ 7.90–7.80 (m, 2H), 7.60–7.51 (m, 2H), 7.32–7.23 (m, 2H), 7.07–6.99 (m, 2H), 3.85 (s, 3H), 2.44–2.28 (m, 2H), 0.93 (s, 9H).

^{13}C{^{1}H} NMR (100 MHz, acetone-d_6) δ 205.78, 161.22, 140.96, 133.55, 130.55, 130.00, 126.37, 126.22, 115.39, 112.81, 101.11–99.90 (m), 55.72, 40.61, 32.32, 29.55.

^{19}F{^{1}H} NMR (377 MHz, acetone-d_6) δ -63.65.

HRMS (ESI): *m/z* $[M+H]^+$ calcd for $C_{23}H_{23}F_3NO^+$, 386.1726; found, 386.1725.

Experimental Procedure for Scheme S6C: In an N_2-filled glovebox, a flame-dried 4 mL screw-cap vial containing a magnetic stir bar was charged with $NiCl_2(py)_4$ (8.9 mg, 0.020 mmol, 10 mol%), 2,2′-bipyridine (**L1**, 3.8 mg, 0.024 mmol, 12 mol%), Mn (33 mg, 0.60 mmol, 3.0 equiv), TBAI (111 mg, 0.30 mmol, 1.5 equiv), 1,3-enyne **1a** (51.7 mg, 0.60 mmol, 1.5 equiv), 1,3-enyne **1b** (67.9 mg, 0.30 mmol, 1.5 equiv), 1,1,1,3,3,3-hexafluoroisopropyl acrylate (**2e**, 200 mg, 0.90 mmol, 4.5 equiv), 4-iodobenzonitrile (**3**, 45.8 mg, 0.20 mmol, 1.0 equiv), 2-iodo-2-methylpropane (**4**, 110 mg, 0.60 mmol, 3.0 equiv) and anhydrous THF (1 mL). The vial was then capped and removed from the glovebox, and the mixture was stirred at 60 °C at a stirring speed of 1500 rpm. After 2 h, the mixture was diluted with ethyl acetate and filtered through a pad of Celite. The filtrate was concentrated *in vacuo*. Yields were determined by ^{1}H NMR spectroscopy using dibromomethane as internal standard.

Scheme S7. Effect of Substituents in Alkenes

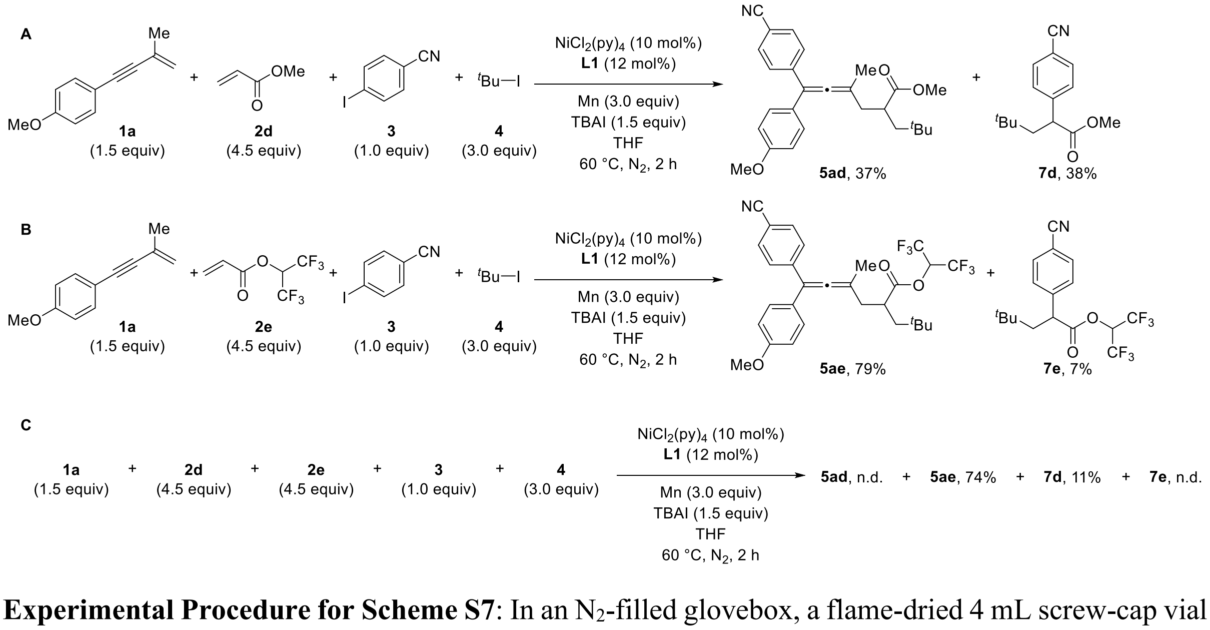

Experimental Procedure for Scheme S7: In an N_2-filled glovebox, a flame-dried 4 mL screw-cap vial containing a magnetic stir bar was charged with $NiCl_2(py)_4$ (8.9 mg, 0.020 mmol, 10 mol%), 2,2′-

bipyridine (**L1**, 3.8 mg, 0.024 mmol, 12 mol%), Mn (33 mg, 0.60 mmol, 3.0 equiv), TBAI (111 mg, 0.30 mmol, 1.5 equiv), 1-methoxy-4-(3-methylbut-3-en-1-yn-1-yl)benzene (**1a**, 51.7 mg, 0.30 mmol, 1.5 equiv), acrylate **2a** (77.5 mg, 0.90 mmol, 4.5 equiv, for **Scheme S7A** and **Scheme S7C**), acrylate **2e** (200 mg, 0.90 mmol, 4.5 equiv, for **Scheme S7B** and **Scheme S7C**), 4-iodobenzonitrile (**3**, 45.8 mg, 0.20 mmol, 1.0 equiv), 2-iodo-2-methylpropane (**4**, 110 mg, 0.60 mmol, 3.0 equiv) and anhydrous THF (1 mL). The vial was then capped and removed from the glovebox, and the mixture was stirred at 60 °C at a stirring speed of 1500 rpm. After 2 h, the mixture was diluted with ethyl acetate and filtered through a pad of Celite. The filtrate was concentrated *in vacuo*. Yields were determined by ^{1}H NMR spectroscopy using dibromomethane as internal standard.

5.3. Mechanistic Investigation of Radical Intermediates

Scheme S8. Radical Trap Experiments

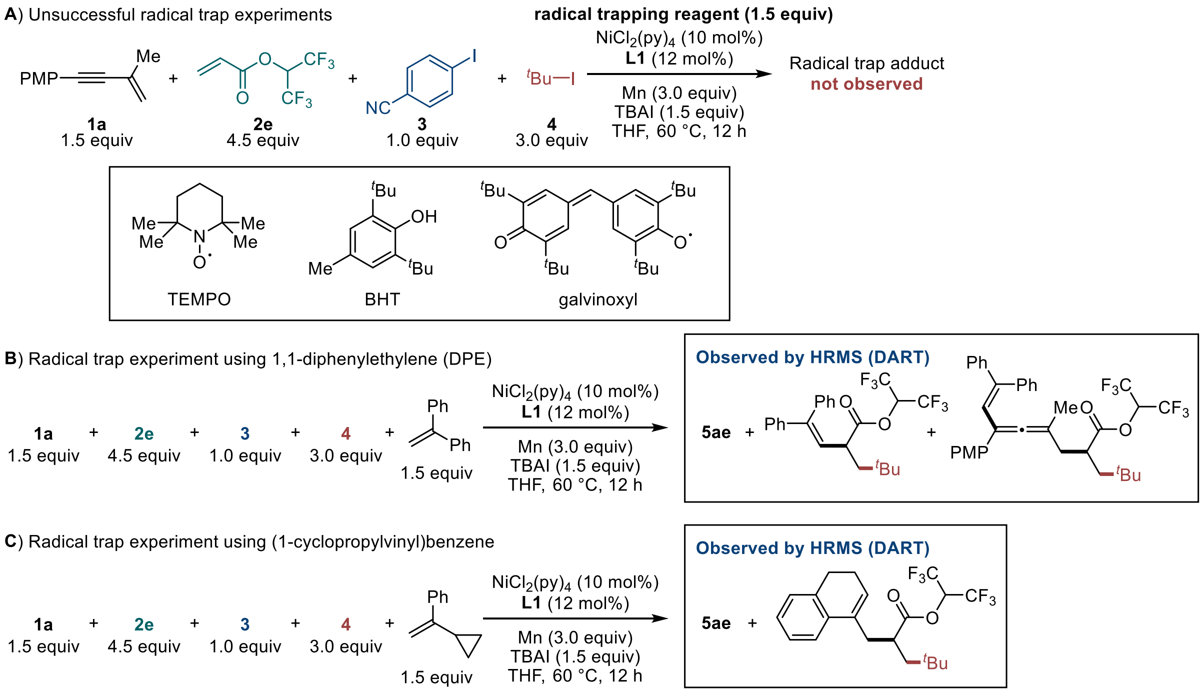

Experimental Procedure for Scheme S8: In an N_2-filled glovebox, a flame-dried 4 mL screw-cap vial containing a magnetic stir bar was charged with $NiCl_2(py)_4$ (8.9 mg, 0.020 mmol, 10 mol%), 2,2′-bipyridine (**L1**, 3.8 mg, 0.024 mmol, 12 mol%), Mn (33 mg, 0.60 mmol, 3.0 equiv), TBAI (111 mg, 0.30 mmol, 1.5 equiv), 1-methoxy-4-(3-methylbut-3-en-1-yn-1-yl)benzene (**1a**, 51.7 mg, 0.30 mmol, 1.5 equiv), 1,1,1,3,3,3-hexafluoroisopropyl acrylate (**2e**, 200 mg, 0.90 mmol, 4.5 equiv), 4-iodobenzonitrile (**3**, 45.8 mg, 0.20 mmol, 1.0 equiv), 2-iodo-2-methylpropane (**4**, 110 mg, 0.60 mmol, 3.0 equiv), the radical trap reagent (0.30 mmol, 1.5 equiv), and anhydrous THF (1 mL). The vial was then capped and removed from the glovebox, and the mixture was stirred at at 60 °C at a stirring speed of 1500 rpm. After 12 h, the mixture was diluted with ethyl acetate and filtered through a pad of Celite.

The filtrate was concentrated *in vacuo*. The resulting mixture was analyzed by high-resolution mass spectrometry (HRMS) using DART ionization.

Adducts of radical intermediates were not observed by mass spectrometry on the reaction mixture when TEMPO, BHT, or galvinoxyl were used, as illustrated in Scheme S8A. By contrast, adducts were observed when 1,1-diphenylethylene (DPE) or (1-cyclopropylvinyl)benzene were used (Schemes S8B and S8C). The high-resolution mass spectrum of a sample of the reaction mixture from **Scheme S8B** showed peaks having the masses expected for the desired product **5ae**, the DPE adduct of the α-carbonyl radical intermediate, and the DPE adduct of the corresponding propargyl/allenyl radical intermediate.
HRMS (DART): *m/z* $[M+H]^+$ calcd for $C_{29}H_{30}F_6NO_3^+$, 554.2124; found, 554.2126.
HRMS (DART): *m/z* $[M+H]^+$ calcd for $C_{24}H_{25}F_6O_2^+$, 459.1753; found, 459.1745.
HRMS (DART): *m/z* $[M+H]^+$ calcd for $C_{36}H_{37}F_6O_3^+$, 631.2641; found, 631.2662.

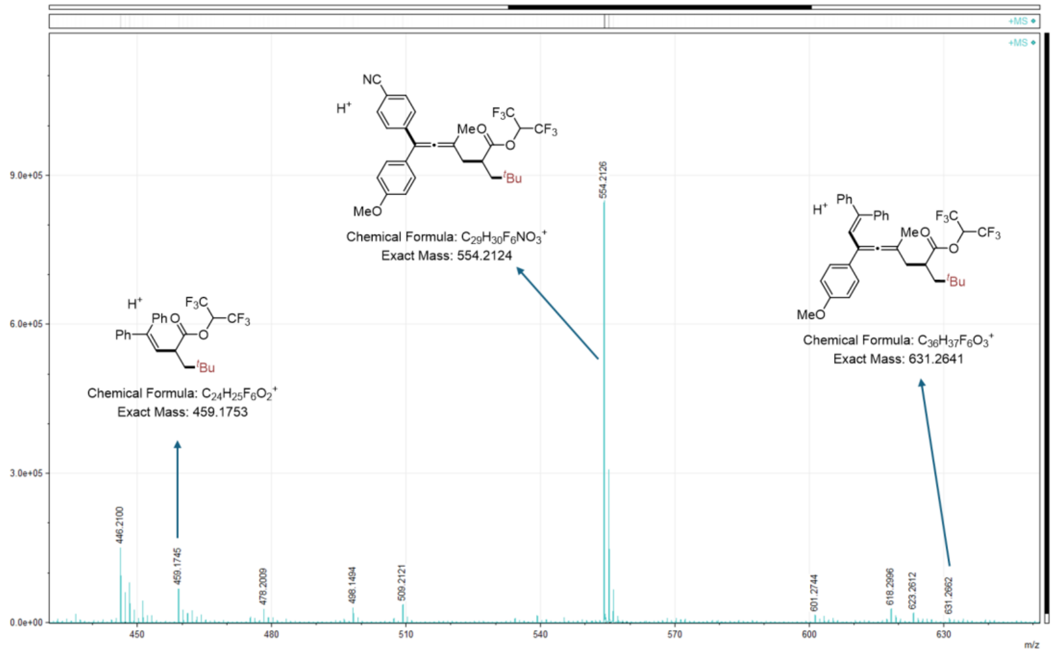

Figure S2. HRMS (DART) of Radical Trap Experiment in Scheme S8B

The high-resolution mass spectrum of a sample of the reaction mixture from **Scheme S8C** showed peaks having the masses expected for the desired product **5ae** and an adduct of the α-carbonyl radical intermediate.
HRMS (DART): *m/z* $[M+H]^+$ calcd for $C_{29}H_{30}F_6NO_3^+$, 554.2124; found, 554.2126.
HRMS (DART): *m/z* $[M+H]^+$ calcd for $C_{21}H_{25}F_6O_2^+$, 423.1753; found, 423.1731.

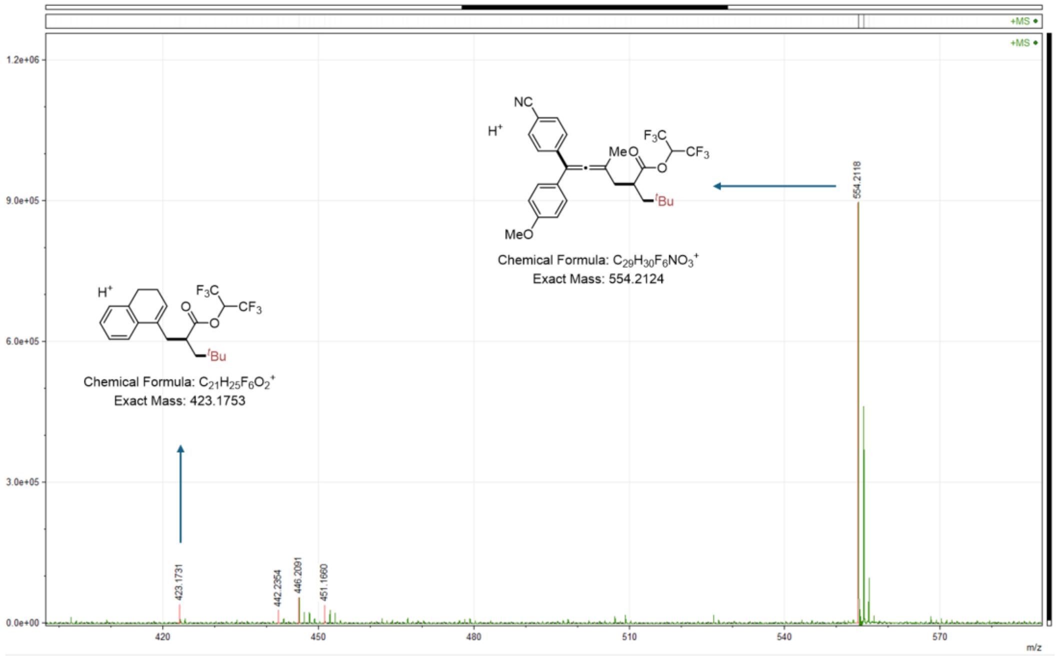

Figure S3. HRMS (DART) of Radical Trap Experiment in Scheme S8C

5.4. Mechanistic Investigation on the Rate-Limiting Step

Scheme S9. Effect of the Stirring Rate on the Yield

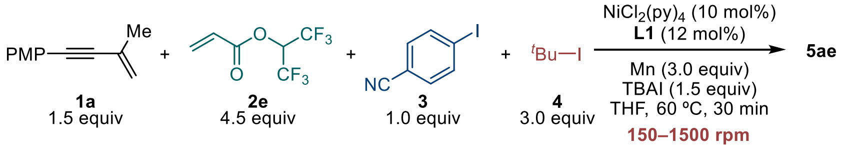

Stirring rate	Yield[a]
150 rpm	13%
300 rpm	38%
600 rpm	57%
1500 rpm	74%

Experimental Procedure for Scheme S9: In an N_2-filled glovebox, a flame-dried 4 mL screw-cap vial containing a magnetic stir bar was charged with $NiCl_2(py)_4$ (8.9 mg, 0.020 mmol, 10 mol%), 2,2´-bipyridine (**L1**, 3.8 mg, 0.024 mmol, 12 mol%), Mn (33 mg, 0.60 mmol, 3.0 equiv), TBAI (111 mg, 0.30 mmol, 1.5 equiv), 1-methoxy-4-(3-methylbut-3-en-1-yn-1-yl)benzene (**1a**, 51.7 mg, 0.30 mmol, 1.5 equiv), 1,1,1,3,3,3-hexafluoroisopropyl acrylate (**2e**, 200 mg, 0.90 mmol, 4.5 equiv), 4-iodobenzonitrile (**3**, 45.8 mg, 0.20 mmol, 1.0 equiv), 2-iodo-2-methylpropane (**4**, 110 mg, 0.60 mmol, 3.0 equiv) and anhydrous THF (1 mL). The vial was then capped and removed from the glovebox, and the mixture was stirred at 60 °C at the given stirring speed (Scheme S9). After 30 min, the mixture was diluted with ethyl acetate and filtered through a pad of Celite. The filtrate was concentrated *in vacuo*. The yield was determined by ^{1}H NMR spectroscopy using dibromomethane as internal standard.

Standard reactions were carried out with various stirring rates (150–1500 rpm) at a shorter reaction time, and the yields were determined. As the stirring rate increased, the product yield increased. This result implies that this XEC using a metallic reducing agent is a mass-transfer-limited reaction. These results, combined with the literature precedent on the Ni-catalyzed reductive dicarbofunctionalization of

alkenes with metallic Zn[14], imply that the rate-limiting step in our system is the heterogeneous reduction of Ni species.

6. Nickel-Catalyzed Five-Component Threefold Conjunctive Coupling

Scheme S10. Nickel-Catalyzed Five-Component Threefold Conjunctive Coupling

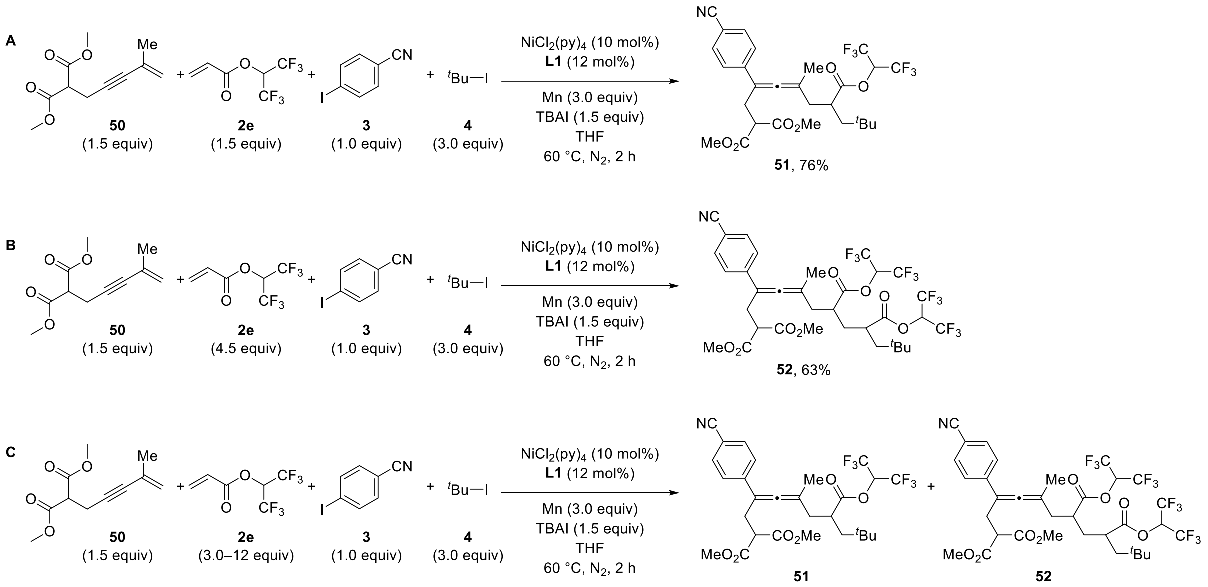

2e	51	52
3.0 equiv	56%	27%
6.0 equiv	31%	63%
12 equiv	15%	82%

7,9-Bis(1,1,1,3,3,3-hexafluoropropan-2-yl) 1,1-dimethyl 3-(4-cyanophenyl)-5,11,11-trimethyldodeca-3,4-diene-1,1,7,9-tetracarboxylate (51)

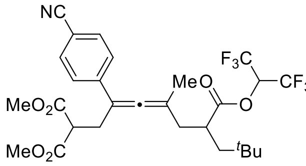

The product was prepared by modified **General procedure II** with dimethyl 2-(4-methylpent-4-en-2-yn-1-yl)malonate (**50**, 63.1 mg, 0.30 mmol, 1.5 equiv), 1,1,1,3,3,3-hexafluoroisopropyl acrylate (**2e**, 66.6 mg, 0.30 mmol, 1.5 equiv), 4-iodobenzonitrile (**3**, 45.8 mg, 0.20 mmol, 1.0 equiv) and 2-iodo-2-methylpropane (**4**, 110 mg, 0.60 mmol, 3.0 equiv) (**Scheme S10A**). The reaction time was 2 h. Flash column chromatography (ethyl acetate/hexanes, 0–30%) afforded the title compound as a colorless oil (89.9 mg, 76%).

^{1}H NMR (400 MHz, acetone-*d*$_6$) δ 7.75 (dt, *J* = 6.7, 2.6 Hz, 2H), 7.64–7.56 (m, 2H), 6.44–6.21 (m, 1H), 3.77–3.69 (m, 6H), 3.73–3.64 (m, 1H), 3.09–3.01 (m, 2H), 2.91–2.82 (m, 1H), 2.59–2.45 (m, 1H), 2.38–2.29 (m, 1H), 1.85 (d, *J* = 1.5 Hz, 3H), 1.79–1.69 (m, 1H), 1.54–1.43 (m, 1H), 0.89 (s, 3.8H), 0.77 (s, 5.2H).

$^{13}C\{^{1}H\}$ NMR (100 MHz, acetone-d_6) δ 203.60, 202.91, 173.93, 173.67, 169.80, 169.63, 142.72, 142.57, 133.10, 133.06, 127.67, 121.79 (q, J = 282.3 Hz), 119.38, 119.36, 111.16, 111.12, 104.72, 104.33, 104.21, 103.78, 67.24 (sept, J = 34.2 Hz), 52.88, 52.86, 51.03, 51.02, 45.26, 45.12, 40.13, 40.09, 39.39, 38.94, 31.24, 31.15, 29.39, 18.82, 18.43.

$^{19}F\{^{1}H\}$ NMR (377 MHz, acetone-d_6) δ -73.57, -73.61 – -73.77 (m).

HRMS (ESI): *m/z* $[M+Na]^+$ calcd for $C_{28}H_{31}F_6NNaO_6^+$, 614.1948; found, 614.1949.

7,9-Bis(1,1,1,3,3,3-hexafluoropropan-2-yl) 1,1-dimethyl 3-(4-cyanophenyl)-5,11,11-trimethyldodeca-3,4-diene-1,1,7,9-tetracarboxylate (52)

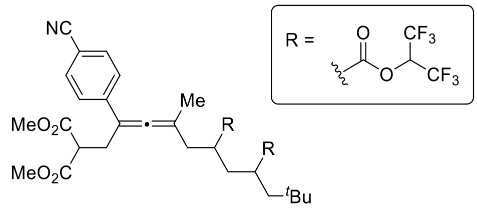

The product was prepared by **General procedure II** with dimethyl 2-(4-methylpent-4-en-2-yn-1-yl)malonate (**50**, 63.1 mg, 0.30 mmol, 1.5 equiv), 1,1,1,3,3,3-hexafluoroisopropyl acrylate (**2e**, 200 mg, 0.90 mmol, 4.5 equiv), 4-iodobenzonitrile (**3**, 45.8 mg, 0.20 mmol, 1.0 equiv) and 2-iodo-2-methylpropane (**4**, 110 mg, 0.60 mmol, 3.0 equiv) (**Scheme S10B**). The reaction time was 2 h. Flash column chromatography (ethyl acetate/hexanes, 0–30%) afforded the title compound as a colorless oil (102 mg, 63%).

^{1}H NMR (400 MHz, acetone-d_6) δ 7.80–7.64 (m, 2H), 7.57 (dd, J = 8.6, 1.9 Hz, 2H), 6.45–6.12 (m, 2H), 3.72–3.67 (m, 3H), 3.57–3.49 (m, 3H), 3.31–3.10 (m, 2H), 2.88–2.81 (m, 1H), 2.72–2.65 (m, 1H), 2.64–2.55 (m, 1H), 2.54–2.42 (m, 1H), 2.40–2.32 (m, 2H), 2.32–2.16 (m,1H), 1.88 (d, J = 3.4 Hz, 3H), 1.76–1.62 (m, 1H), 1.48–1.33 (m, 1H), 0.83 (s, 4.3H), 0.74 (s, 4.7H).

$^{13}C\{^{1}H\}$ NMR (100 MHz, acetone-d_6) δ 204.89, 173.83, 173.64, 171.24, 171.16, 171.10, 170.48, 143.74, 143.36, 132.99, 128.45, 128.43, 128.32, 121.71 (q, J = 284.5 Hz), 119.34, 119.30, 111.30, 111.24, 102.49, 101.62, 101.50, 100.95, 100.90, 67.22 (sept, J = 34.3 Hz), 57.00, 56.89, 53.11, 52.92, 52.85, 45.35, 44.93, 40.16, 40.07, 39.54, 39.01, 33.98, 33.82, 31.12, 29.43, 29.38, 29.17, 29.14, 27.61, 18.90, 18.28.

$^{19}F\{^{1}H\}$ NMR (377 MHz, acetone-d_6) δ -73.61, -73.66 – -73.81 (m), -74.00, -74.02.

HRMS (ESI): *m/z* $[M+H]^+$ calcd for $C_{34}H_{36}F_{12}NO_8^+$, 814.2244; found, 814.2249.

Experimental Procedure for Scheme S10C: In an N_2-filled glovebox, a flame-dried 4 mL screw-cap vial containing a magnetic stir bar was charged with $NiCl_2(py)_4$ (8.9 mg, 0.020 mmol, 10 mol%), 2,2′-bipyridine (**L1**, 3.8 mg, 0.024 mmol, 12 mol%), Mn (33 mg, 0.60 mmol, 3.0 equiv), TBAI (111 mg, 0.30 mmol, 1.5 equiv), dimethyl 2-(4-methylpent-4-en-2-yn-1-yl)malonate (**50**, 63.1 mg, 0.30 mmol,

1.5 equiv), 1,1,1,3,3,3-hexafluoroisopropyl acrylate (**2e**, 0.600–2.40 mmol, 3.0–12.0 equiv), 4-iodobenzonitrile (**3**, 45.8 mg, 0.20 mmol, 1.0 equiv), 2-iodo-2-methylpropane (**4**, 110 mg, 0.60 mmol, 3.0 equiv) and anhydrous THF (1 mL). The vial was then capped and removed from the glovebox, and the mixture was stirred at 60°C at a stirring speed of 1500 rpm. After 2 h, the mixture was diluted with ethyl acetate and filtered through a pad of Celite. The filtrate was concentrated *in vacuo*. Yields of the products were determined by ^{1}H NMR using dibromomethane as internal standard.

7. Computational Studies

7.1. Computational Details

All calculations were performed using the density functional theory[15] (DFT) as implemented in the Gaussian 16[16] suite of programs. Geometry optimizations were performed using the uM06 functional[17] and a mixed basis set of LANL2DZ[18–20] for Ni and I, and 6-31G**[21] for all other atoms. Vibrational frequency calculations were carried out at the same level of theory as that used for geometry optimizations, wherein thermochemistry correction energy (G – E) was acquired. Transition states were realized by the presence of single imaginary frequency and confirmed by intrinsic reaction coordinate calculations (IRC). Single point energies of optimized structures were calculated with the uM06 functional[17] and a mixed basis set of LANL2DZ for Ni and I, and 6-311+G** for all other atoms. Solvation effects were incorporated using the SMD model[22] based on the gas-phase optimized geometries and carried out at the same level as single-point calculations. Implicit THF solvent was modelled with the SMD continuum solvation.[22] Final solution phase Gibbs free energies were calculated as follows:

$$G_{sol} = E_{sol} + (G - E) \quad (1)$$

$$\Delta G(sol) = \Sigma G(sol) \text{ for products} - \Sigma G(sol) \text{ for reactants} \quad (2)$$

7.2. Summarized Energy Components of All DFT-Optimized Structures

Table S8. Summarized Energy Components of DFT-Optimized Structures

**DFT-optimized Structures**	**E(sol)** (SCF/TZ) [eV] uM06/6-311+G**, LANL2DZ(Ni & I)/ SMD(THF)	**G–E** (Thermochemistry correction energy) [eV] uM06/6-31G**, LANL2DZ(Ni & I)	**G(sol)** [eV]
**L_2Ni^IIm**	-18393.380471	3.168821	-18390.211649
**$L_2Ni^{II}ArI$**	-27202.298294	5.402442	-27196.895852
**1a**	-14677.460410	4.426260	-14673.034150
**int1**	-4291.341336	2.347745	-4288.993591
**TS1_enyne**	-18968.925975	7.352383	-18961.573592
**int2_enyne**	-18970.370899	7.543788	-18962.827111
**2d**	-8336.772944	1.768632	-8335.004312
**TS1_Me**	-12628.281846	4.727735	-12623.554111
**int2_Me**	-12629.422479	4.872309	-12624.550170
**TS2_Me_a**	-27306.933370	9.873818	-27297.059552
**int3_Me_a**	-27308.240514	10.076516	-27298.163998
**TS2_Me_b**	-39832.070737	11.015690	-39821.055047
**int3_Me_b**	-39832.247775	10.987826	-39821.259949
**TS3_Me_b**	-39832.005026	10.983798	-39821.021228
**7d**	-21440.288087	7.168434	-21433.119653
**2e**	-26678.840767	1.770646	-26677.070122
**TS1_HFIP**	-30970.452378	4.747246	-30965.705132
**int2_HFIP**	-30971.550505	4.887194	-30966.663311
**TS2_HFIP_a**	-45649.145728	9.932595	-45639.213133
**int3_HFIP_a**	-45650.381419	10.163157	-45640.218262
**TS3_HFIP_a**	-72852.873836	16.386512	-72836.487323
**int4_HFIP_a**	-2678.976131	0.598886	-1680707.302749
**TS4_HFIP_a**	-72852.949149	16.309722	-72836.639428
**5ae**	-72852.873836	16.386512	-72836.487323
**TS2_HFIP_b**	-58174.224199	11.176292	-58163.047907
**int3_HFIP_b**	-58174.432532	11.204347	-58163.228185
**TS3_HFIP_b**	-58174.154215	11.100699	-58163.053516
**7e**	-39782.376414	7.202666	-39775.173748

Cartesian Coordinates of Optimized Geometries

==

L_2Ni^II

==

Charge: 0, Spin: 2

Cartesian coordinates:

ATOM	X	Y	Z
C	-0.401096000	3.191894000	0.982128000
C	0.981062000	3.710732000	0.956099000
C	-1.497865000	3.925219000	1.427259000
C	-2.755701000	3.337741000	1.409884000
C	-2.888101000	2.033086000	0.948789000
C	-1.749137000	1.364970000	0.520952000
N	-0.536668000	1.925951000	0.535739000
N	1.894781000	2.837727000	0.492452000
C	3.178340000	3.195073000	0.424394000
C	3.619172000	4.451929000	0.818379000
C	2.683232000	5.360985000	1.298121000
C	1.346755000	4.989190000	1.369272000
Ni	1.163842000	1.043499000	-0.053774000
I	2.434128000	-0.882774000	-0.951228000
H	-1.374544000	4.943528000	1.784617000
H	-3.623439000	3.894433000	1.753684000
H	-3.853339000	1.537293000	0.919118000
H	-1.791665000	0.342518000	0.150032000
H	3.856754000	2.434817000	0.039291000
H	4.672067000	4.706798000	0.748617000
H	2.989982000	6.354164000	1.615641000
H	0.603427000	5.688212000	1.741853000

==

$L_2Ni^{II}ArI$

==

Charge: 0, Spin: 1

Cartesian coordinates:

ATOM	X	Y	Z
C	-0.516410000	3.186275000	0.383689000
C	0.830778000	2.963571000	0.932488000
C	-1.601569000	3.588429000	1.157246000
C	-2.836775000	3.774747000	0.553370000
C	-2.954207000	3.553505000	-0.811266000
C	-1.830834000	3.153982000	-1.521539000
N	-0.636085000	2.972302000	-0.945723000
N	1.754146000	2.581302000	0.026558000
C	3.005624000	2.358128000	0.436877000
C	3.397627000	2.502604000	1.761695000
C	2.451099000	2.894570000	2.697284000
C	1.148783000	3.128835000	2.277602000
Ni	1.028122000	2.410790000	-1.843574000
C	0.214681000	2.290729000	-3.520406000
I	3.109410000	1.701958000	-3.022593000
C	0.085764000	3.428688000	-4.325802000
C	-0.734817000	3.418180000	-5.446003000
C	-1.434505000	2.253508000	-5.788973000
C	-1.282390000	1.098685000	-5.009841000
C	-0.459075000	1.121060000	-3.891966000
C	-2.299738000	2.244113000	-6.926049000
N	-3.014355000	2.240283000	-7.845164000
H	-1.485134000	3.755979000	2.223433000
H	-3.693612000	4.088679000	1.143434000
H	-3.898222000	3.685585000	-1.330406000
H	-1.877954000	2.970033000	-2.591987000
H	3.704126000	2.052831000	-0.342008000
H	4.427994000	2.308451000	2.042712000
H	2.719143000	3.017943000	3.743310000
H	0.392453000	3.436238000	2.993171000
H	0.632577000	4.336631000	-4.073542000
H	-0.842859000	4.306348000	-6.065178000
H	-1.814272000	0.192216000	-5.291476000
H	-0.340730000	0.215059000	-3.298519000

==

1a

==

Charge: 0, Spin: 1

Cartesian coordinates:

ATOM	X	Y	Z
C	0.247039000	0.046003000	-7.491324000
C	-0.618748000	0.942464000	-6.861983000
C	0.976222000	-0.870475000	-6.725024000
C	-0.747354000	0.915412000	-5.478783000
C	0.841988000	-0.890071000	-5.351428000
C	-0.024932000	0.005161000	-4.698707000
H	-1.194782000	1.662143000	-7.435999000
H	1.642448000	-1.556479000	-7.241689000
H	-1.420706000	1.612354000	-4.985263000
H	1.409203000	-1.602552000	-4.757198000
C	-0.162783000	-0.014619000	-3.283995000
C	-0.295420000	-0.014335000	-2.075599000
C	-0.403382000	-0.067459000	-0.655780000
C	-1.216010000	0.763803000	0.013538000
H	-1.819294000	1.505188000	-0.502396000
C	0.442267000	-1.102481000	0.031309000
H	0.193519000	-2.108563000	-0.326704000
H	1.505299000	-0.942445000	-0.184569000
H	0.298108000	-1.072502000	1.115803000
H	-1.291813000	0.715869000	1.097273000
O	0.449110000	-0.014987000	-8.828516000
C	-0.259862000	0.885511000	-9.643597000
H	-0.014312000	1.930198000	-9.404474000
H	0.040708000	0.676531000	-10.672212000

H	-1.346766000	0.746444000	-9.553383000

==

int1

==

Charge: 0, Spin: 2

Cartesian coordinates:

ATOM	X	Y	Z
C	-0.161299000	0.001987000	-0.113123000
C	0.387095000	1.384564000	-0.145691000
H	-0.402303000	2.132195000	-0.299211000
H	0.892486000	1.657079000	0.799468000
H	1.134324000	1.507567000	-0.940805000
C	-1.456186000	-0.243023000	0.577114000
H	-1.914782000	-1.188513000	0.258813000
H	-1.339938000	-0.308852000	1.674891000
H	-2.175538000	0.565677000	0.391992000
C	0.758589000	-1.143408000	-0.347903000
H	1.343041000	-1.403147000	0.554277000
H	0.212605000	-2.052360000	-0.633174000
H	1.490814000	-0.922538000	-1.135763000

==

TS1_enyne

==

Charge: 0, Spin: 2

Imaginary frequency: –300.5614 cm^{-1}

Cartesian coordinates:

ATOM	X	Y	Z
C	0.104659000	-1.790639000	0.728870000
C	0.192905000	-3.148439000	0.121045000
H	-0.350594000	-3.896864000	0.713904000
H	-0.215395000	-3.152998000	-0.898559000
H	1.240033000	-3.492255000	0.049334000
C	0.536337000	-0.646611000	-0.124523000
H	0.032693000	-0.663801000	-1.100915000
H	0.324055000	0.319834000	0.351700000
H	1.622917000	-0.677699000	-0.319614000
C	0.324241000	-1.676348000	2.199603000
H	-0.031349000	-0.712457000	2.589033000
H	-0.183864000	-2.478459000	2.751347000
H	1.396532000	-1.744048000	2.455907000
C	-2.590567000	2.671875000	-5.796255000
C	-2.133751000	3.056230000	-4.534321000
C	-3.082479000	1.376310000	-5.991515000
C	-2.172538000	2.147564000	-3.483927000
C	-3.117413000	0.479600000	-4.942767000
C	-2.662018000	0.847133000	-3.662240000
H	-1.747612000	4.056028000	-4.358863000
H	-3.431712000	1.103975000	-6.984224000
H	-1.816622000	2.444031000	-2.499817000
H	-3.500218000	-0.526510000	-5.096908000
C	-2.694975000	-0.072770000	-2.582363000
C	-2.712650000	-0.846748000	-1.639799000
C	-2.745940000	-1.778694000	-0.576196000
C	-2.285129000	-1.440961000	0.662064000
H	-2.052590000	-0.407367000	0.907123000
C	-3.248001000	-3.159666000	-0.894198000
H	-4.287373000	-3.129085000	-1.244607000
H	-2.658867000	-3.627112000	-1.693791000
H	-3.201577000	-3.806225000	-0.011272000
H	-2.394475000	-2.135513000	1.493684000
O	-2.598629000	3.474544000	-6.887807000
C	-2.114900000	4.786357000	-6.742056000
H	-1.059195000	4.799070000	-6.434590000
H	-2.205362000	5.262128000	-7.720696000
H	-2.703883000	5.358426000	-6.010607000

==

int2_enyne

==

Charge: 0, Spin: 2

Cartesian coordinates:

ATOM	X	Y	Z
C	-0.120914000	-1.635550000	0.974804000
C	0.834392000	-0.725397000	0.486894000
C	1.636944000	0.108124000	0.054276000
C	2.503514000	1.046962000	-0.436734000
C	3.959003000	1.002317000	-0.091402000
C	2.015849000	2.119062000	-1.371164000
C	0.246255000	-2.724115000	1.796478000
C	-0.703089000	-3.604927000	2.269131000
C	-2.054457000	-3.433591000	1.941386000
C	-2.441209000	-2.362912000	1.131173000
C	-1.482038000	-1.479221000	0.657334000
O	-2.904714000	-4.353518000	2.456836000
C	-4.272513000	-4.225129000	2.157989000
C	1.265727000	3.306949000	-0.722419000
C	2.122970000	3.946373000	0.365948000
C	-0.060849000	2.847450000	-0.121866000
C	0.987835000	4.334239000	-1.817492000
H	4.183162000	0.208033000	0.626315000
H	4.569139000	0.832781000	-0.991530000
H	4.298142000	1.958383000	0.333860000
H	2.884376000	2.524991000	-1.915192000
H	1.347281000	1.676969000	-2.127188000
H	1.294344000	-2.859676000	2.052436000
H	-0.432226000	-4.446571000	2.901557000
H	-3.483158000	-2.209211000	0.866070000
H	-1.777952000	-0.641235000	0.029368000
H	-4.779672000	-5.051728000	2.659717000
H	-4.682813000	-3.275249000	2.530122000
H	-4.460871000	-4.293457000	1.076823000
H	3.087206000	4.288408000	-0.035735000

H	1.613737000	4.818152000	0.797417000
H	2.322244000	3.238862000	1.181422000
H	-0.688705000	2.358688000	-0.880144000
H	-0.617729000	3.707426000	0.273636000
H	0.088496000	2.134320000	0.696930000
H	1.921078000	4.706655000	-2.260904000
H	0.381541000	3.899510000	-2.623490000
H	0.440476000	5.196332000	-1.414260000

===

2d

===

Charge: 0, Spin: 1

Cartesian coordinates:

ATOM	X	Y	Z
C	1.661065000	1.831742000	-4.176315000
H	2.009924000	2.808469000	-3.854591000
H	0.772696000	1.421011000	-3.701094000
C	2.285000000	1.136279000	-5.123310000
H	3.175972000	1.497895000	-5.631226000
C	1.769953000	-0.189630000	-5.525758000
O	0.795546000	-0.739685000	-5.065557000
O	2.538866000	-0.722829000	-6.492861000
C	2.120901000	-2.004537000	-6.947478000
H	1.106337000	-1.959249000	-7.355783000
H	2.830198000	-2.299624000	-7.721758000
H	2.128803000	-2.728601000	-6.126524000

===

TS1_Me

===

Charge: 0, Spin: 2

Imaginary frequency: –264.95 cm^{-1}

Cartesian coordinates:

ATOM	X	Y	Z
C	-2.226698000	0.053543000	-3.720736000
C	-1.632137000	-1.049449000	-2.966276000
O	-1.196244000	-0.987923000	-1.833371000
O	-1.619421000	-2.187427000	-3.695683000
C	-1.049919000	-3.308736000	-3.036995000
C	-2.294533000	1.286878000	-3.165077000
C	-4.303087000	1.492793000	-1.924561000
C	-3.970768000	2.724586000	-1.151059000
C	-5.294082000	1.631432000	-3.031349000
C	-4.294461000	0.198612000	-1.185141000
H	-2.627170000	-0.167920000	-4.707341000
H	-1.602548000	-3.547930000	-2.122227000
H	-1.770403000	1.472330000	-2.229328000
H	-2.618902000	2.150346000	-3.740570000
H	-4.826576000	3.042277000	-0.530537000
H	-3.126412000	2.562058000	-0.469457000
H	-3.728882000	3.569132000	-1.810422000
H	-6.323645000	1.670710000	-2.635518000
H	-5.248638000	0.776275000	-3.718457000
H	-5.139307000	2.551681000	-3.609564000
H	-3.367109000	0.058004000	-0.616035000
H	-5.136556000	0.149839000	-0.473483000
H	-4.400095000	-0.652053000	-1.870513000
H	-1.107641000	-4.139711000	-3.741969000
H	-0.007485000	-3.114695000	-2.763641000

===

int2_Me

===

Charge: 0, Spin: 2

Cartesian coordinates:

ATOM	X	Y	Z
C	-2.593294000	-0.188934000	-3.496192000
C	-2.760631000	-0.300563000	-4.931396000
O	-2.895048000	0.636051000	-5.698762000
O	-2.753865000	-1.591922000	-5.334822000
C	-2.917870000	-1.775030000	-6.733345000
C	-2.564518000	1.135269000	-2.840931000
C	-3.879458000	1.522275000	-2.112869000
C	-3.698719000	2.922368000	-1.535321000
C	-5.038348000	1.523355000	-3.105291000
C	-4.166637000	0.538835000	-0.982199000
H	-2.509472000	-1.111089000	-2.923217000
H	-2.113019000	-1.282638000	-7.289412000
H	-1.751881000	1.164275000	-2.097077000
H	-2.354722000	1.898257000	-3.603246000
H	-4.604146000	3.242873000	-1.003629000
H	-2.862590000	2.955032000	-0.824041000
H	-3.497520000	3.654286000	-2.328240000
H	-5.960353000	1.868617000	-2.619546000
H	-5.226023000	0.516054000	-3.500455000
H	-4.830542000	2.180663000	-3.958922000
H	-3.334871000	0.501580000	-0.265533000
H	-5.068493000	0.837626000	-0.432125000
H	-4.334740000	-0.476890000	-1.362657000
H	-2.893158000	-2.852145000	-6.905599000
H	-3.871404000	-1.357215000	-7.073459000

===

TS2_Me_a

===

Charge: 0, Spin: 2

Imaginary frequency: –405.6291 cm^{-1}

Cartesian coordinates:

ATOM	X	Y	Z
C	-2.478625000	3.362272000	-5.278805000
C	-2.155972000	3.163304000	-3.840481000
C	-3.156534000	2.287263000	-3.060410000
C	-2.697734000	2.244920000	-1.604877000

C	-4.554840000	2.893696000	-3.140769000
C	-3.162237000	0.863896000	-3.614750000
C	1.404290000	0.440896000	-2.710801000
C	0.921611000	0.895341000	-3.963666000
C	0.466861000	1.345133000	-5.003729000
C	-0.124919000	1.837017000	-6.185003000
C	-0.591393000	0.829501000	-7.195115000
C	1.512952000	1.331526000	-1.633419000
C	1.964775000	0.907765000	-0.390673000
C	2.321757000	-0.430102000	-0.206924000
C	2.219213000	-1.331286000	-1.274059000
C	1.766590000	-0.903680000	-2.505265000
O	2.774016000	-0.949314000	0.956882000
C	2.892914000	-0.086807000	2.062516000
C	-0.407747000	3.171642000	-6.318742000
H	-2.872121000	2.537268000	-5.871932000
H	-1.165800000	2.690115000	-3.731958000
H	-2.090621000	4.154629000	-3.368694000
H	-3.374035000	1.628641000	-0.997784000
H	-1.688466000	1.816161000	-1.523295000
H	-2.675847000	3.252147000	-1.167958000
H	-5.267890000	2.321075000	-2.532838000
H	-4.929013000	2.903822000	-4.173376000
H	-4.552499000	3.930853000	-2.778690000
H	-2.151160000	0.432548000	-3.594271000
H	-3.818931000	0.218806000	-3.015956000
H	-3.526195000	0.824949000	-4.649957000
H	0.229014000	0.178844000	-7.519716000
H	-1.361662000	0.176245000	-6.761917000
H	-1.015019000	1.323204000	-8.075651000
H	1.232822000	2.372611000	-1.782574000
H	2.034476000	1.623694000	0.422671000
H	2.503488000	-2.365857000	-1.100498000
H	1.685520000	-1.605273000	-3.331982000
H	3.267001000	-0.692781000	2.889939000
H	3.603183000	0.728886000	1.866112000
H	1.922853000	0.346040000	2.345362000
H	0.003638000	3.902532000	-5.625988000
H	-0.802642000	3.548301000	-7.260060000
C	-2.796494000	4.696133000	-5.760829000
O	-2.655189000	5.732400000	-5.137600000
O	-3.249056000	4.668481000	-7.041187000
C	-3.564290000	5.942004000	-7.582408000
H	-2.688456000	6.599756000	-7.579277000
H	-3.900848000	5.765174000	-8.605539000
H	-4.356023000	6.429409000	-7.003462000

==

int3_Me_a

==

Charge: 0, Spin: 2

Cartesian coordinates:

ATOM	X	Y	Z
C	-1.246113000	0.848161000	-10.771635000
C	-1.776383000	0.347305000	-9.448015000
O	-2.547930000	-0.575236000	-9.330245000
O	-1.305110000	1.052616000	-8.402524000
C	-1.714024000	0.571646000	-7.125969000
C	-2.356964000	1.516685000	-11.591710000
C	-2.988817000	2.819591000	-11.072155000
C	-3.858814000	3.373906000	-12.201549000
C	-3.884745000	2.579843000	-9.855216000
C	-1.913502000	3.846814000	-10.723296000
C	2.209559000	0.568284000	-7.727700000
C	1.655791000	-0.066424000	-8.852728000
C	1.111068000	-0.564736000	-9.844597000
C	0.460058000	-1.071561000	-10.931452000
C	0.868779000	-2.359122000	-11.570009000
C	1.686881000	1.798861000	-7.285449000
C	2.212571000	2.448053000	-6.177369000
C	3.283329000	1.877514000	-5.483501000
C	3.816506000	0.654209000	-5.910919000
C	3.291764000	0.010598000	-7.011507000
O	3.872042000	2.424870000	-4.393368000
C	3.379140000	3.656592000	-3.927898000
C	-0.700417000	-0.348944000	-11.552796000
H	-2.804195000	0.614039000	-7.025931000
H	-1.929970000	1.726412000	-12.585847000
H	-3.156157000	0.773873000	-11.750213000
H	-4.362151000	4.298157000	-11.889536000
H	-3.258131000	3.602549000	-13.091984000
H	-4.633526000	2.652315000	-12.493691000
H	-4.432694000	3.497988000	-9.605200000
H	-3.309797000	2.297037000	-8.966617000
H	-4.621177000	1.789913000	-10.052267000
H	-1.235084000	4.014823000	-11.571340000
H	-2.372937000	4.810591000	-10.467267000
H	-1.311244000	3.529342000	-9.862474000
H	1.752509000	-2.789122000	-11.091178000
H	0.054264000	-3.096699000	-11.518278000
H	1.089864000	-2.216007000	-12.638419000
H	0.847969000	2.230564000	-7.829540000
H	1.785854000	3.396888000	-5.865204000
H	4.649192000	0.235295000	-5.351616000
H	3.708263000	-0.938348000	-7.340831000
H	3.981667000	3.924070000	-3.057522000
H	3.476154000	4.446391000	-4.686721000
H	2.324751000	3.584310000	-3.624213000
H	-0.417312000	-0.014468000	-12.566384000
H	-1.523787000	-1.068418000	-11.698605000
H	-1.233028000	1.217876000	-6.389381000
H	-1.386824000	-0.464471000	-6.990557000
H	-0.432613000	1.560296000	-10.577305000

==

TS2_Me_b

==

Charge: 0, Spin: 2

Imaginary frequency: –71.3073 cm^{-1}

Cartesian coordinates:

ATOM	X	Y	Z
C	0.036472000	2.152118000	0.647997000
C	1.388144000	2.293660000	0.080639000
C	-0.229902000	2.256696000	2.011342000
C	-1.529983000	2.080119000	2.462122000
C	-2.530211000	1.803794000	1.540427000
C	-2.188652000	1.717133000	0.197774000
N	-0.938676000	1.886658000	-0.241610000
N	1.489573000	2.067127000	-1.245883000
C	2.693901000	2.124626000	-1.823897000
C	3.850247000	2.430528000	-1.118609000
C	3.749453000	2.694979000	0.239700000
C	2.503880000	2.621254000	0.847240000
Ni	-0.283479000	1.685424000	-2.171208000
C	0.503984000	1.857056000	-3.880033000
I	-2.538791000	2.061252000	-3.311082000
C	0.596380000	3.173366000	-4.351762000
C	1.402249000	3.483536000	-5.438085000
C	2.118929000	2.470194000	-6.087664000
C	2.017292000	1.152572000	-5.630889000
C	1.216844000	0.850496000	-4.532944000
C	2.959017000	2.784084000	-7.200941000
N	3.650893000	3.042388000	-8.100541000
H	0.567144000	2.464557000	2.718394000
H	-1.755842000	2.155313000	3.522602000
H	-3.561087000	1.655755000	1.846242000
H	-2.933149000	1.515029000	-0.572872000
H	2.724518000	1.914274000	-2.890408000
H	4.803125000	2.462600000	-1.637037000
H	4.629105000	2.951479000	0.824145000
H	2.403939000	2.819982000	1.909754000
H	0.033336000	3.969751000	-3.868231000
H	1.476831000	4.507732000	-5.796825000
H	2.581894000	0.367804000	-6.129658000
H	1.211094000	-0.169010000	-4.159057000
C	-0.437065000	-0.578858000	-1.950953000
C	0.875350000	-0.867703000	-1.387000000
O	1.867072000	-1.223729000	-1.998401000
O	0.895192000	-0.686125000	-0.033626000
C	-0.850127000	-1.342136000	-3.172108000
C	-1.775418000	-2.545772000	-2.868221000
C	-1.998841000	-3.293934000	-4.180532000
C	-1.113669000	-3.483824000	-1.861035000
C	-3.127331000	-2.074013000	-2.336570000
H	-1.370649000	-0.689143000	-3.888156000
H	0.046863000	-1.730332000	-3.674222000
H	-2.666271000	-4.153189000	-4.031738000
H	-2.454875000	-2.639393000	-4.934726000
H	-1.051401000	-3.670627000	-4.587995000
H	-1.724825000	-4.383576000	-1.709924000
H	-0.984105000	-3.003424000	-0.881481000
H	-0.122683000	-3.802568000	-2.212692000
H	-3.610302000	-1.387243000	-3.044194000
H	-3.796256000	-2.930916000	-2.179833000
H	-3.037247000	-1.551854000	-1.374486000
H	-1.212158000	-0.435201000	-1.198329000
C	2.144403000	-0.941495000	0.585080000
H	2.009786000	-0.715662000	1.646506000
H	2.441574000	-1.987825000	0.457517000
H	2.936519000	-0.310003000	0.163540000

==

int3_Me_b

==

Charge: 0, Spin: 2

Cartesian coordinates:

ATOM	X	Y	Z
C	0.037074000	2.033031000	0.777541000
C	1.408251000	2.221020000	0.270979000
C	-0.303761000	2.058282000	2.128836000
C	-1.635287000	1.917706000	2.490810000
C	-2.597689000	1.756810000	1.500203000
C	-2.179353000	1.732123000	0.178282000
N	-0.898949000	1.862394000	-0.168045000
N	1.548050000	2.121153000	-1.063964000
C	2.733346000	2.370508000	-1.620144000
C	3.858601000	2.685706000	-0.869215000
C	3.733153000	2.756291000	0.511690000
C	2.491275000	2.531596000	1.089869000
Ni	-0.234790000	1.758857000	-2.099905000
C	0.204421000	1.554178000	-3.965710000
I	-0.715639000	4.336036000	-2.348764000
C	-0.751414000	1.926548000	-4.916228000
C	-0.494165000	1.773504000	-6.271149000
C	0.733257000	1.251480000	-6.697479000
C	1.685810000	0.868666000	-5.749781000
C	1.413057000	1.010088000	-4.393139000
C	1.005756000	1.098564000	-8.092562000
N	1.228328000	0.971314000	-9.227807000
H	0.458807000	2.186124000	2.891130000
H	-1.920079000	1.936357000	3.539395000
H	-3.650398000	1.651270000	1.741939000
H	-2.885385000	1.606083000	-0.643072000
H	2.769983000	2.324187000	-2.706239000
H	4.804696000	2.876971000	-1.365332000
H	4.589454000	3.001016000	1.134553000
H	2.366143000	2.614241000	2.165308000
H	-1.701505000	2.357867000	-4.607560000

H	-1.235655000	2.070730000	-7.009227000
H	2.627771000	0.436436000	-6.079736000
H	2.127299000	0.608283000	-3.678430000
C	-0.472858000	-0.284961000	-2.058742000
C	0.831036000	-0.760214000	-1.559373000
O	1.775938000	-1.139127000	-2.221997000
O	0.890846000	-0.714578000	-0.198080000
C	-1.009815000	-1.013456000	-3.270055000
C	-1.760481000	-2.325666000	-2.949801000
C	-2.129630000	-2.975781000	-4.282099000
C	-0.883961000	-3.289603000	-2.152382000
C	-3.046340000	-2.036924000	-2.175027000
H	-1.708170000	-0.366905000	-3.823078000
H	-0.182609000	-1.233203000	-3.959773000
H	-2.693345000	-3.904576000	-4.122909000
H	-2.750280000	-2.305664000	-4.891694000
H	-1.231319000	-3.221555000	-4.862822000
H	-1.397868000	-4.250432000	-2.015786000
H	-0.651071000	-2.899732000	-1.152016000
H	0.064772000	-3.481491000	-2.669802000
H	-3.690674000	-1.339701000	-2.728712000
H	-3.616127000	-2.961782000	-2.014902000
H	-2.849182000	-1.604447000	-1.184633000
H	-1.197388000	-0.283402000	-1.239162000
C	2.141178000	-1.093174000	0.355488000
H	2.042013000	-0.977816000	1.437881000
H	2.383373000	-2.131430000	0.105528000
H	2.949365000	-0.452912000	-0.019779000

==

TS3_Me_b

==

Charge: 0, Spin: 2

Imaginary frequency: –228.6737 cm^{-1}

Cartesian coordinates:

ATOM	X	Y	Z
C	0.095375000	2.141324000	0.818219000
C	1.472946000	2.240712000	0.296647000
C	-0.225181000	2.208808000	2.173176000
C	-1.558299000	2.136146000	2.553258000
C	-2.538209000	2.005454000	1.576773000
C	-2.135095000	1.944854000	0.249303000
N	-0.856902000	2.000854000	-0.115218000
N	1.602694000	2.107298000	-1.037356000
C	2.803303000	2.257085000	-1.598263000
C	3.950296000	2.503978000	-0.856658000
C	3.832798000	2.613273000	0.522758000
C	2.579542000	2.490368000	1.106220000
Ni	-0.154823000	1.905827000	-2.094119000
C	0.123812000	1.267896000	-3.905143000
I	-0.606179000	4.461798000	-2.494479000
C	-0.895867000	1.648762000	-4.792739000
C	-0.645277000	1.748864000	-6.148300000
C	0.627279000	1.440233000	-6.653064000
C	1.631236000	1.018607000	-5.777653000
C	1.375425000	0.917704000	-4.415601000
C	0.888473000	1.531402000	-8.054293000
N	1.102388000	1.598409000	-9.196555000
H	0.549966000	2.319001000	2.925786000
H	-1.828901000	2.187241000	3.604647000
H	-3.591901000	1.954528000	1.832486000
H	-2.859460000	1.857186000	-0.561271000
H	2.832636000	2.182839000	-2.683508000
H	4.907152000	2.613527000	-1.356841000
H	4.705994000	2.807581000	1.139980000
H	2.464767000	2.602513000	2.180101000
H	-1.883302000	1.909165000	-4.413907000
H	-1.426433000	2.078011000	-6.829092000
H	2.609008000	0.752971000	-6.172815000
H	2.137295000	0.504752000	-3.760124000
C	-0.477582000	-0.157158000	-2.442337000
C	0.690841000	-0.653197000	-1.660099000
O	1.744486000	-1.033069000	-2.125032000
O	0.449514000	-0.662329000	-0.326062000
C	-0.917542000	-1.141029000	-3.522056000
C	-1.584610000	-2.438976000	-3.011599000
C	-2.050081000	-3.200748000	-4.253421000
C	-0.616204000	-3.330239000	-2.233514000
C	-2.800552000	-2.128243000	-2.139271000
H	-1.644177000	-0.642515000	-4.177939000
H	-0.057108000	-1.404773000	-4.153737000
H	-2.529562000	-4.147320000	-3.972518000
H	-2.775747000	-2.614724000	-4.832450000
H	-1.204363000	-3.434829000	-4.912966000
H	-1.089626000	-4.296762000	-2.015945000
H	-0.331871000	-2.889890000	-1.269689000
H	0.300240000	-3.521604000	-2.805634000
H	-3.495472000	-1.448781000	-2.652608000
H	-3.348566000	-3.050323000	-1.905884000
H	-2.516218000	-1.670260000	-1.181835000
H	-1.330961000	0.026963000	-1.774516000
C	1.530228000	-1.136376000	0.467594000
H	1.214009000	-1.023310000	1.506950000
H	1.741843000	-2.188365000	0.245666000
H	2.440391000	-0.554219000	0.279862000

==

7d

==

Charge: 0, Spin: 1

Cartesian coordinates:

ATOM	X	Y	Z
C	1.197897000	-2.168222000	-0.428648000
C	1.407604000	-3.306488000	-1.213190000

C	1.976266000	-4.445077000	-0.663781000
C	2.348690000	-4.460085000	0.685080000
C	2.143503000	-3.325161000	1.476193000
C	1.571620000	-2.192768000	0.916360000
C	2.935945000	-5.633576000	1.255064000
N	3.413788000	-6.586982000	1.719737000
H	1.106039000	-3.295799000	-2.258324000
H	2.136260000	-5.331664000	-1.272017000
H	2.432024000	-3.342153000	2.523990000
H	1.411365000	-1.308909000	1.531707000
C	0.631928000	-0.896790000	-1.026491000
C	-0.621986000	-1.216575000	-1.812508000
O	-0.641291000	-1.746215000	-2.899690000
O	-1.726883000	-0.851884000	-1.146613000
C	-2.949503000	-1.092968000	-1.840776000
C	1.680297000	-0.216738000	-1.921415000
C	1.440461000	1.260051000	-2.276894000
C	2.561440000	1.682544000	-3.226295000
C	0.097134000	1.469933000	-2.975740000
C	1.503183000	2.132608000	-1.023875000
H	0.349578000	-0.230922000	-0.200717000
H	-3.062080000	-2.156408000	-2.070689000
H	2.653547000	-0.292138000	-1.412578000
H	1.763791000	-0.807311000	-2.846383000
H	2.463579000	2.741301000	-3.498578000
H	3.547503000	1.544932000	-2.763194000
H	2.538301000	1.093300000	-4.152168000
H	0.003234000	2.512529000	-3.306146000
H	-0.749777000	1.269846000	-2.305099000
H	-0.003189000	0.823560000	-3.856827000
H	2.455327000	1.996756000	-0.493419000
H	1.418933000	3.193711000	-1.291862000
H	0.688249000	1.912862000	-0.322252000
H	-2.969287000	-0.528828000	-2.779646000
H	-3.746581000	-0.757405000	-1.176809000

==

2e

==

Charge: 0, Spin: 1

Cartesian coordinates:

ATOM	X	Y	Z
C	0.320661000	0.491433000	-3.661907000
H	0.288185000	1.335174000	-2.979506000
H	-0.421336000	-0.294653000	-3.539925000
C	1.233905000	0.403431000	-4.626448000
H	1.997280000	1.158719000	-4.793486000
C	1.231683000	-0.761074000	-5.525250000
O	0.471239000	-1.694746000	-5.498317000
O	2.243462000	-0.645249000	-6.440033000
C	2.362159000	-1.696177000	-7.366441000
H	1.501931000	-2.374803000	-7.298504000
C	2.382276000	-1.076903000	-8.753317000
F	3.378008000	-0.210395000	-8.898346000
F	2.507944000	-2.024825000	-9.679545000
F	1.235147000	-0.432579000	-8.960016000
C	3.613632000	-2.488497000	-7.023299000
F	4.712436000	-1.747784000	-7.119794000
F	3.517228000	-2.930744000	-5.770575000
F	3.737557000	-3.536486000	-7.833790000

==

TS1_HFIP

==

Charge: 0, Spin: 2

Imaginary frequency: –181.7957 cm^{-1}

Cartesian coordinates:

ATOM	X	Y	Z
C	0.403716000	1.115735000	-4.074209000
H	0.505896000	1.964905000	-3.403966000
H	-0.450502000	0.457545000	-3.928308000
C	1.419330000	0.719998000	-4.874388000
H	2.331711000	1.297253000	-4.999635000
C	1.266486000	-0.482604000	-5.676341000
O	0.339932000	-1.260393000	-5.667509000
O	2.353731000	-0.641082000	-6.508730000
C	2.290609000	-1.725228000	-7.394756000
H	1.483607000	-2.414768000	-7.112984000
C	1.979887000	-1.191231000	-8.784932000
F	2.958702000	-0.425806000	-9.258006000
F	1.779267000	-2.192968000	-9.637985000
F	0.867770000	-0.455482000	-8.734014000
C	3.613862000	-2.464595000	-7.301071000
F	4.651552000	-1.665270000	-7.520642000
F	3.740270000	-2.982708000	-6.080747000
F	3.653515000	-3.458630000	-8.187775000
C	-1.076427000	2.507235000	-5.406742000
C	-0.250901000	3.746473000	-5.488254000
H	0.733412000	3.545461000	-5.930860000
H	-0.739441000	4.501028000	-6.128365000
H	-0.104654000	4.210498000	-4.504347000
C	-2.250050000	2.523130000	-4.487275000
H	-3.049259000	3.174533000	-4.881112000
H	-2.686878000	1.524186000	-4.364840000
H	-1.987630000	2.914312000	-3.495096000
C	-1.170218000	1.674552000	-6.638294000
H	-1.832600000	2.151732000	-7.380615000
H	-0.191360000	1.550745000	-7.120351000
H	-1.576126000	0.676810000	-6.430618000

==

int2_HFIP

==

Charge: 0, Spin: 2

Cartesian coordinates:

ATOM	X	Y	Z
C	-2.238079000	0.013854000	-3.271254000
C	-1.831557000	-1.149273000	-2.522214000
O	-1.607830000	-1.197362000	-1.331048000
O	-1.712831000	-2.244973000	-3.347784000
C	-1.309013000	-3.439092000	-2.731507000
C	-2.413562000	1.318569000	-2.603312000
C	-1.236969000	2.310018000	-2.822662000
C	-1.553434000	3.582365000	-2.043516000
C	0.059501000	1.697483000	-2.301037000
C	-1.094988000	2.641908000	-4.305168000
H	-2.380118000	-0.103875000	-4.344107000
H	-3.330193000	1.802180000	-2.976261000
H	-2.530632000	1.153950000	-1.523608000
H	-0.750782000	4.320594000	-2.167633000
H	-2.488164000	4.041245000	-2.391929000
H	-1.656497000	3.375260000	-0.970585000
H	0.882438000	2.419503000	-2.378717000
H	0.344612000	0.807163000	-2.877729000
H	-0.035469000	1.397703000	-1.249712000
H	-2.026443000	3.057912000	-4.712381000
H	-0.303003000	3.386513000	-4.456357000
H	-0.828677000	1.756484000	-4.896563000
H	-1.018463000	-3.261692000	-1.687434000
C	-2.495311000	-4.390035000	-2.722460000
F	-2.937663000	-4.655120000	-3.947033000
F	-2.171404000	-5.538423000	-2.132720000
F	-3.489203000	-3.827775000	-2.035112000
C	-0.090035000	-3.949071000	-3.481355000
F	-0.334724000	-4.142755000	-4.772505000
F	0.888510000	-3.050040000	-3.374991000
F	0.333183000	-5.096392000	-2.955281000

==

TS2_HFIP_a

==

Charge: 0, Spin: 2

Imaginary frequency: –386.4533 cm^{-1}

Cartesian coordinates:

ATOM	X	Y	Z
C	-2.527009000	3.379840000	-5.187686000
C	-2.166874000	3.219210000	-3.754265000
C	-3.152882000	2.367430000	-2.928446000
C	-2.646573000	2.342815000	-1.488428000
C	-4.545916000	2.989210000	-2.973244000
C	-3.192526000	0.936535000	-3.461716000
C	1.389395000	0.479957000	-2.762273000
C	0.898552000	0.980335000	-3.993438000
C	0.437115000	1.455766000	-5.019313000
C	-0.168553000	1.969848000	-6.182518000
C	-0.626937000	0.983344000	-7.216168000
C	1.526636000	1.331567000	-1.656852000
C	1.984386000	0.856930000	-0.435165000
C	2.319001000	-0.493160000	-0.302093000
C	2.188593000	-1.355357000	-1.398501000
C	1.729698000	-0.877584000	-2.608151000
O	2.773668000	-1.059900000	0.836766000
C	2.921263000	-0.239751000	1.971658000
C	-0.473538000	3.303848000	-6.281017000
H	-2.889396000	2.528394000	-5.761781000
H	-1.177772000	2.739699000	-3.666354000
H	-2.081856000	4.219779000	-3.306013000
H	-3.314691000	1.751318000	-0.848876000
H	-1.643932000	1.895363000	-1.431215000
H	-2.591511000	3.356937000	-1.070914000
H	-5.245684000	2.430604000	-2.337896000
H	-4.952783000	2.991992000	-3.993511000
H	-4.522667000	4.029664000	-2.621748000
H	-2.183982000	0.499454000	-3.488147000
H	-3.819238000	0.303656000	-2.819756000
H	-3.612377000	0.883614000	-4.474740000
H	0.203994000	0.363874000	-7.572951000
H	-1.372472000	0.298014000	-6.789468000
H	-1.077273000	1.493132000	-8.073788000
H	1.264616000	2.382197000	-1.766948000
H	2.076927000	1.542361000	0.401723000
H	2.456187000	-2.400025000	-1.263691000
H	1.626216000	-1.548233000	-3.457656000
H	3.290552000	-0.882906000	2.772579000
H	3.646728000	0.567195000	1.796774000
H	1.963036000	0.201906000	2.279529000
H	-0.064957000	4.024813000	-5.576025000
H	-0.873850000	3.696468000	-7.213717000
C	-2.903633000	4.677781000	-5.687176000
O	-2.815095000	5.744453000	-5.116551000
O	-3.360309000	4.578700000	-6.993666000
C	-3.638948000	5.788949000	-7.638314000
H	-3.650456000	6.621996000	-6.921964000
C	-2.521719000	6.049749000	-8.636533000
F	-1.371282000	6.184564000	-7.970308000
F	-2.372950000	5.044225000	-9.495631000
F	-2.740720000	7.166065000	-9.325337000
F	-5.097695000	4.678751000	-9.136523000
C	-5.020855000	5.674142000	-8.258209000
F	-5.913914000	5.448982000	-7.296660000
F	-5.349066000	6.806746000	-8.878753000

==

int3_HFIP_a

==

Charge: 0, Spin: 2

Cartesian coordinates:

ATOM	X	Y	Z
C	-1.453101000	0.757093000	-10.554635000

C	-2.030342000	0.136826000	-9.309738000
O	-2.828339000	-0.762219000	-9.277955000
O	-1.553511000	0.742988000	-8.174746000
C	-2.041430000	0.249886000	-6.953107000
C	-2.522815000	1.523583000	-11.345358000
C	-3.242231000	2.710562000	-10.679111000
C	-3.927297000	3.499488000	-11.796375000
C	-4.321649000	2.239374000	-9.704174000
C	-2.256426000	3.629039000	-9.957214000
C	2.259309000	0.783032000	-7.878737000
C	1.653534000	0.079331000	-8.933031000
C	1.053525000	-0.485676000	-9.855193000
C	0.331638000	-1.056589000	-10.862349000
C	0.735354000	-2.342242000	-11.506747000
C	1.624090000	1.920313000	-7.347451000
C	2.175087000	2.625411000	-6.288870000
C	3.391891000	2.209108000	-5.741060000
C	4.040102000	1.080260000	-6.260260000
C	3.484721000	0.376738000	-7.307880000
O	4.020246000	2.823300000	-4.711224000
C	3.407636000	3.957529000	-4.148738000
C	-0.879050000	-0.364932000	-11.422802000
H	-2.013820000	1.897887000	-12.247576000
H	-3.279244000	0.802183000	-11.695703000
H	-4.511805000	4.332089000	-11.384521000
H	-3.192182000	3.918240000	-12.496395000
H	-4.612428000	2.859972000	-12.368973000
H	-4.875554000	3.100820000	-9.308605000
H	-3.911244000	1.705995000	-8.841847000
H	-5.039325000	1.573971000	-10.201464000
H	-1.437966000	3.936068000	-10.623147000
H	-2.767121000	4.539113000	-9.616909000
H	-1.816471000	3.151324000	-9.071607000
H	1.662051000	-2.736504000	-11.081662000
H	-0.050148000	-3.103062000	-11.388385000
H	0.880505000	-2.212355000	-12.589954000
H	0.665193000	2.224832000	-7.763223000
H	1.645164000	3.487616000	-5.894363000
H	4.983183000	0.779893000	-5.810891000
H	3.986495000	-0.502481000	-7.704685000
H	4.064475000	4.299955000	-3.346609000
H	3.292323000	4.764676000	-4.886382000
H	2.420961000	3.718950000	-3.726606000
H	-0.634273000	0.050744000	-12.416258000
H	-1.672101000	-1.109256000	-11.601829000
H	-2.996937000	-0.272492000	-7.096701000
C	-1.059632000	-0.763039000	-6.368807000
F	-1.641840000	-1.427410000	-5.370133000
F	-0.708064000	-1.634896000	-7.305887000
F	0.039801000	-0.182973000	-5.897198000
C	-2.276695000	1.457559000	-6.057985000
F	-2.541384000	1.068463000	-4.813495000
F	-1.230978000	2.278900000	-6.030971000
F	-3.322565000	2.152077000	-6.511020000
H	-0.643775000	1.437807000	-10.260708000

===

TS3_HFIP_a

===

Charge: 0, Spin: 2

Imaginary frequency: –134.1591 cm^{-1}

Cartesian coordinates:

ATOM	X	Y	Z
C	-5.209566000	-1.364748000	-6.665905000
C	-6.670524000	-1.695606000	-6.563091000
O	-7.374390000	-2.150234000	-7.424494000
O	-7.165179000	-1.308816000	-5.336154000
C	-8.562748000	-1.257699000	-5.218827000
C	-4.359768000	-1.900474000	-5.514730000
C	-4.284956000	-3.423697000	-5.309000000
C	-3.237640000	-3.677740000	-4.223275000
C	-5.613875000	-4.010791000	-4.834694000
C	-3.852311000	-4.123096000	-6.596210000
C	-1.202446000	0.613737000	-4.141549000
C	-2.226042000	1.190267000	-4.975037000
C	-2.957909000	0.951563000	-5.979051000
C	-3.809359000	0.792684000	-7.005978000
C	-3.514290000	1.330619000	-8.369220000
C	-1.488551000	-0.528108000	-3.371712000
C	-0.539703000	-1.082928000	-2.533293000
C	0.729066000	-0.502393000	-2.430092000
C	1.034585000	0.630494000	-3.190170000
C	0.073438000	1.178326000	-4.031252000
O	1.585254000	-1.101012000	-1.567328000
C	2.862007000	-0.532745000	-1.410040000
C	-5.164014000	0.184098000	-6.770772000
C	-9.003473000	-2.303252000	-4.208685000
F	-8.781639000	-3.515758000	-4.710876000
F	-10.299761000	-2.182981000	-3.942343000
F	-8.330991000	-2.211730000	-3.057619000
C	-8.922240000	0.175280000	-4.856769000
F	-10.239911000	0.340466000	-4.840261000
F	-8.397856000	0.990516000	-5.766190000
F	-8.440429000	0.520275000	-3.660345000
H	-4.873317000	-1.793041000	-7.620240000
H	-9.042683000	-1.502557000	-6.176150000
H	-3.336979000	-1.533880000	-5.687337000
H	-4.698952000	-1.429569000	-4.577788000
H	-3.138767000	-4.751920000	-4.020508000
H	-2.250265000	-3.297283000	-4.518340000
H	-3.522553000	-3.186052000	-3.280345000
H	-5.485544000	-5.069512000	-4.574237000
H	-6.393254000	-3.970226000	-5.605051000

H	-5.988408000	-3.487291000	-3.944015000
H	-2.918070000	-3.696828000	-6.986450000
H	-3.682864000	-5.192160000	-6.414272000
H	-4.617815000	-4.044193000	-7.379357000
H	-2.464730000	1.615700000	-8.483529000
H	-3.768639000	0.598161000	-9.148038000
H	-4.131353000	2.224230000	-8.551728000
H	-2.479518000	-0.974995000	-3.443244000
H	-0.755376000	-1.971566000	-1.943326000
H	2.014301000	1.095928000	-3.130429000
H	0.305020000	2.071807000	-4.610212000
H	3.387669000	-1.145253000	-0.674865000
H	3.429972000	-0.538069000	-2.351096000
H	2.801674000	0.501435000	-1.038906000
H	-5.597480000	0.606442000	-5.854904000
H	-5.831511000	0.495138000	-7.588745000
C	-2.175924000	2.168233000	-1.469933000
C	-3.371416000	1.333998000	-1.687426000
C	-3.743891000	0.292413000	-0.838436000
N	-4.089026000	1.634015000	-2.787628000
N	-1.922975000	3.078860000	-2.431902000
C	-1.345643000	2.035522000	-0.358858000
C	-4.868757000	-0.460790000	-1.142543000
H	-3.148220000	0.053783000	0.037505000
C	-5.613963000	-0.136910000	-2.269676000
H	-5.162080000	-1.288411000	-0.501788000
C	-5.191893000	0.928571000	-3.053838000
H	-6.505048000	-0.688833000	-2.556039000
H	-5.756721000	1.254032000	-3.926642000
Ni	-3.203626000	2.959855000	-4.050899000
C	-0.853218000	3.868480000	-2.310597000
C	0.010747000	3.803280000	-1.225689000
H	-0.691449000	4.578115000	-3.120542000
C	-0.240857000	2.865068000	-0.234186000
H	0.862182000	4.474755000	-1.172261000
H	0.413164000	2.777765000	0.629670000
H	-1.558515000	1.292843000	0.403847000
C	-2.281221000	4.273258000	-5.065096000
I	-5.359580000	3.773058000	-5.230883000
C	-2.383880000	5.592044000	-4.595028000
C	-1.401444000	4.014957000	-6.121070000
C	-1.602279000	6.609128000	-5.123463000
H	-3.091391000	5.828087000	-3.800307000
C	-0.700414000	6.329375000	-6.159288000
H	-1.687846000	7.626792000	-4.748243000
C	-0.613270000	5.026737000	-6.659801000
C	0.124596000	7.364587000	-6.697333000
H	0.079229000	4.813095000	-7.471620000
H	-1.315405000	3.009923000	-6.527448000
N	0.805450000	8.204759000	-7.128431000

==

int4_HFIP_a

==

Charge: 0, Spin: 2

Cartesian coordinates:

ATOM	X	Y	Z
C	-5.493445000	1.783466000	-1.966303000
C	-4.278250000	1.748328000	-1.130400000
C	-6.703846000	1.196806000	-1.598961000
C	-7.780518000	1.269682000	-2.470458000
C	-7.633281000	1.939864000	-3.679051000
C	-6.403826000	2.515471000	-3.966038000
N	-5.362224000	2.423015000	-3.138477000
N	-3.223540000	2.432010000	-1.609599000
C	-2.066327000	2.390529000	-0.949109000
C	-1.904188000	1.690707000	0.239097000
C	-2.994061000	1.000918000	0.752534000
C	-4.194855000	1.025291000	0.058418000
Ni	-3.537009000	3.241059000	-3.538803000
I	-4.571382000	5.614805000	-3.181903000
H	-6.809030000	0.691438000	-0.643843000
H	-8.728275000	0.808125000	-2.208802000
H	-8.449987000	2.009365000	-4.390910000
H	-6.231092000	3.065912000	-4.889887000
H	-1.235264000	2.924731000	-1.404073000
H	-0.939810000	1.683709000	0.737101000
H	-2.908930000	0.437524000	1.677958000
H	-5.052230000	0.473600000	0.431602000
C	-1.899713000	4.066492000	-4.144118000
C	-1.056604000	4.711387000	-3.233941000
C	-1.553486000	4.075171000	-5.497498000
C	0.137249000	5.285869000	-3.649105000
H	-1.350433000	4.813838000	-2.190725000
C	0.494716000	5.253829000	-5.002059000
H	0.791098000	5.781554000	-2.934818000
C	-0.371645000	4.663132000	-5.929351000
C	1.730889000	5.826878000	-5.433193000
H	-0.103029000	4.657941000	-6.983476000
H	-2.203684000	3.597666000	-6.228688000
N	2.743153000	6.283966000	-5.781496000
C	-5.344350000	-1.306727000	-6.599418000
C	-6.693998000	-1.920827000	-6.817410000
O	-7.176822000	-2.249805000	-7.868543000
O	-7.388740000	-1.991312000	-5.632757000
C	-8.743950000	-2.338612000	-5.731619000
C	-4.492405000	-1.985084000	-5.527636000
C	-4.060549000	-3.442566000	-5.753018000
C	-3.069411000	-3.790224000	-4.641819000
C	-5.244472000	-4.405403000	-5.667399000
C	-3.371513000	-3.600950000	-7.107013000
C	-1.800361000	0.783486000	-3.720620000
C	-2.837480000	1.584428000	-4.374493000

C	-3.558976000	1.299636000	-5.406528000
C	-4.353675000	1.031543000	-6.432742000
C	-4.058288000	1.484054000	-7.833509000
C	-2.170894000	-0.379291000	-3.024369000
C	-1.233638000	-1.146058000	-2.355104000
C	0.114320000	-0.771907000	-2.366613000
C	0.503719000	0.376258000	-3.058518000
C	-0.449964000	1.143044000	-3.722588000
O	0.958196000	-1.581770000	-1.681798000
C	2.328404000	-1.263454000	-1.696089000
C	-5.583950000	0.182327000	-6.237851000
C	-8.986765000	-3.532108000	-4.821803000
F	-8.432106000	-4.614761000	-5.363717000
F	-10.292595000	-3.750592000	-4.680193000
F	-8.454303000	-3.356840000	-3.616520000
C	-9.562928000	-1.107359000	-5.378459000
F	-10.847948000	-1.295857000	-5.652102000
F	-9.125004000	-0.071023000	-6.097232000
F	-9.453062000	-0.783474000	-4.087983000
H	-4.843942000	-1.359477000	-7.575740000
H	-8.997758000	-2.630785000	-6.759572000
H	-3.583480000	-1.371968000	-5.424359000
H	-5.021100000	-1.913130000	-4.562115000
H	-2.719509000	-4.825867000	-4.739883000
H	-2.192063000	-3.129561000	-4.666598000
H	-3.536529000	-3.687281000	-3.652036000
H	-4.892019000	-5.443748000	-5.719446000
H	-5.954962000	-4.272694000	-6.493344000
H	-5.793882000	-4.282852000	-4.724436000
H	-2.547147000	-2.883597000	-7.219974000
H	-2.953108000	-4.610529000	-7.207928000
H	-4.070482000	-3.454675000	-7.941140000
H	-3.094103000	1.996086000	-7.899398000
H	-4.039543000	0.636153000	-8.532746000
H	-4.837474000	2.169321000	-8.195903000
H	-3.221583000	-0.673381000	-3.017791000
H	-1.514981000	-2.049472000	-1.818471000
H	1.545377000	0.682011000	-3.094248000
H	-0.137354000	2.037571000	-4.257768000
H	2.832930000	-2.032792000	-1.108158000
H	2.736167000	-1.267969000	-2.716904000
H	2.523522000	-0.281044000	-1.242067000
H	-5.923931000	0.221127000	-5.193825000
H	-6.402615000	0.580455000	-6.860918000

==

TS4_HFIP_a

==

Charge: 0, Spin: 2

Imaginary frequency: –182.3965 cm^{-1}

Cartesian coordinates:

ATOM	X	Y	Z
C	-5.346039000	1.281033000	-1.968648000
C	-4.164908000	1.331647000	-1.083320000
C	-6.408229000	0.391612000	-1.815670000
C	-7.500044000	0.499149000	-2.668652000
C	-7.519428000	1.505752000	-3.627221000
C	-6.407064000	2.330539000	-3.736602000
N	-5.343647000	2.196439000	-2.948592000
N	-3.213243000	2.210498000	-1.445996000
C	-2.139067000	2.365845000	-0.674507000
C	-1.932944000	1.624904000	0.481434000
C	-2.900199000	0.699094000	0.851610000
C	-4.037278000	0.558957000	0.068783000
Ni	-3.528061000	3.134085000	-3.288511000
I	-4.406158000	5.483897000	-2.552150000
H	-6.388724000	-0.374044000	-1.045254000
H	-8.332041000	-0.193824000	-2.582434000
H	-8.365636000	1.635218000	-4.294086000
H	-6.346181000	3.120744000	-4.485570000
H	-1.425140000	3.121066000	-0.997084000
H	-1.037222000	1.778984000	1.074509000
H	-2.777780000	0.099849000	1.749952000
H	-4.819273000	-0.137742000	0.356164000
C	-1.996396000	3.796455000	-4.292421000
C	-0.786926000	4.094023000	-3.665191000
C	-2.270210000	4.362281000	-5.544499000
C	0.120509000	4.965976000	-4.254124000
H	-0.536331000	3.648203000	-2.705028000
C	-0.168026000	5.544325000	-5.493178000
H	1.055357000	5.209771000	-3.754513000
C	-1.376598000	5.234815000	-6.136698000
C	0.769494000	6.429005000	-6.107921000
H	-1.599210000	5.685700000	-7.100871000
H	-3.203365000	4.120689000	-6.054911000
N	1.539032000	7.142070000	-6.612442000
C	-5.173928000	-1.168722000	-6.555753000
C	-6.539493000	-1.712093000	-6.859063000
O	-6.924220000	-2.126640000	-7.920353000
O	-7.368792000	-1.619638000	-5.761792000
C	-8.708108000	-1.978559000	-5.982217000
C	-4.497673000	-1.784330000	-5.331330000
C	-4.158450000	-3.284258000	-5.371341000
C	-3.317277000	-3.590572000	-4.132277000
C	-5.411990000	-4.156346000	-5.332923000
C	-3.344074000	-3.620182000	-6.619788000
C	-1.632219000	0.827630000	-3.686212000
C	-2.604816000	1.717219000	-4.351219000
C	-3.314734000	1.395233000	-5.392954000
C	-4.011127000	1.104292000	-6.476316000
C	-3.509332000	1.439628000	-7.853745000
C	-2.093685000	-0.266581000	-2.936452000
C	-1.221022000	-1.101524000	-2.263117000

C	0.157918000	-0.874361000	-2.329282000
C	0.640634000	0.185037000	-3.097002000
C	-0.252134000	1.019629000	-3.766284000
O	0.933624000	-1.735545000	-1.628153000
C	2.328402000	-1.562320000	-1.692672000
C	-5.323507000	0.364464000	-6.394661000
C	-9.092816000	-3.015231000	-4.937173000
F	-8.531332000	-4.182284000	-5.244033000
F	-10.413135000	-3.178919000	-4.904455000
F	-8.679942000	-2.672082000	-3.718046000
C	-9.568901000	-0.721829000	-5.947666000
F	-10.753165000	-0.948988000	-6.501744000
F	-8.963690000	0.251405000	-6.625769000
F	-9.776017000	-0.284448000	-4.699403000
H	-4.582588000	-1.372832000	-7.458717000
H	-8.826329000	-2.430323000	-6.976344000
H	-3.557444000	-1.229732000	-5.189717000
H	-5.115434000	-1.569979000	-4.440602000
H	-3.055070000	-4.655601000	-4.092658000
H	-2.385358000	-3.009432000	-4.128451000
H	-3.868032000	-3.347537000	-3.211893000
H	-5.133022000	-5.215907000	-5.264461000
H	-6.024988000	-4.051341000	-6.237068000
H	-6.040051000	-3.917905000	-4.464529000
H	-2.464123000	-2.968407000	-6.707777000
H	-2.989083000	-4.657765000	-6.576657000
H	-3.939332000	-3.519320000	-7.536839000
H	-2.528527000	1.922466000	-7.819200000
H	-3.425701000	0.537270000	-8.475447000
H	-4.209310000	2.112215000	-8.368914000
H	-3.165977000	-0.457976000	-2.888693000
H	-1.580186000	-1.947132000	-1.680032000
H	1.707029000	0.367986000	-3.191838000
H	0.137574000	1.835378000	-4.369904000
H	2.768596000	-2.346349000	-1.073407000
H	2.703410000	-1.665456000	-2.720856000
H	2.635159000	-0.581857000	-1.300664000
H	-5.819105000	0.543903000	-5.433934000
H	-5.992064000	0.744289000	-7.183164000

==

5ae

==

Charge: 0, Spin: 1

Cartesian coordinates:

ATOM	X	Y	Z
C	2.407865000	-1.939972000	-0.987108000
C	1.796913000	-2.536240000	0.115680000
C	3.804701000	-1.838702000	-1.002322000
C	2.550490000	-3.018560000	1.177210000
H	0.714107000	-2.618871000	0.139818000
C	3.942412000	-2.906463000	1.153136000
H	2.064295000	-3.478964000	2.033423000
C	4.568739000	-2.310829000	0.051853000
C	4.725493000	-3.395592000	2.245875000
H	5.652008000	-2.222936000	0.033727000
H	4.310861000	-1.374334000	-1.847226000
N	5.364277000	-3.793609000	3.132828000
C	1.930049000	1.047335000	-1.307968000
C	0.493169000	1.302394000	-0.916801000
O	-0.344197000	1.790154000	-1.629942000
O	0.250214000	0.939334000	0.380310000
C	-1.075703000	1.078680000	0.832441000
C	2.596897000	2.372727000	-1.722404000
C	3.278561000	3.183851000	-0.610899000
C	3.812460000	4.467423000	-1.245770000
C	2.293967000	3.549542000	0.496838000
C	4.453410000	2.400425000	-0.024916000
C	-2.439443000	-1.274773000	-1.462666000
C	-1.045533000	-1.408823000	-1.728999000
C	0.153661000	-1.445667000	-1.903332000
C	1.595366000	-1.405292000	-2.167266000
C	1.885450000	-2.265015000	-3.411794000
C	-3.135578000	-2.244514000	-0.734946000
C	-4.477309000	-2.079015000	-0.415661000
C	-5.144790000	-0.921240000	-0.821613000
C	-4.459601000	0.057916000	-1.551131000
C	-3.127361000	-0.117062000	-1.872202000
O	-6.443076000	-0.654539000	-0.558715000
C	-7.173642000	-1.597441000	0.188785000
C	1.989374000	0.057519000	-2.478247000
C	-1.396579000	-0.096149000	1.751202000
F	-2.715193000	-0.178133000	1.919550000
F	-0.825423000	0.032268000	2.946772000
F	-0.976893000	-1.241801000	1.221619000
C	-1.226642000	2.416761000	1.549850000
F	-2.405436000	2.476398000	2.168088000
F	-1.161560000	3.417974000	0.675857000
F	-0.266840000	2.600105000	2.452130000
H	2.450084000	0.611044000	-0.445349000
H	-1.786243000	1.054544000	-0.005253000
H	3.362755000	2.147292000	-2.480777000
H	1.844720000	2.995797000	-2.229997000
H	2.996464000	5.069180000	-1.666923000
H	4.333966000	5.082696000	-0.501543000
H	4.521895000	4.247072000	-2.054793000
H	2.771187000	4.214255000	1.228407000
H	1.938008000	2.668723000	1.044327000
H	1.418541000	4.075734000	0.091837000
H	4.133764000	1.483525000	0.487124000
H	5.169008000	2.116851000	-0.809578000
H	4.991187000	3.009544000	0.712865000
H	1.589454000	-3.304185000	-3.235525000

H	1.321219000	-1.886012000	-4.271285000
H	2.953388000	-2.247292000	-3.659183000
H	-2.607637000	-3.136145000	-0.403522000
H	-4.987799000	-2.848955000	0.154758000
H	-5.004345000	0.948643000	-1.852983000
H	-2.586869000	0.649256000	-2.424657000
H	-7.227336000	-2.568087000	-0.324346000
H	-6.740620000	-1.745147000	1.188185000
H	-8.182696000	-1.194612000	0.294047000
H	3.018966000	0.059034000	-2.861478000
H	1.351373000	0.427161000	-3.294149000

==

TS2_HFIP_b

==

Charge: 0, Spin: 2
Imaginary frequency: –71.4649 cm^{-1}
Cartesian coordinates:

ATOM	X	Y	Z
C	0.171080000	2.219013000	0.666769000
C	1.472223000	2.540543000	0.061416000
C	-0.032564000	2.152991000	2.042657000
C	-1.290722000	1.820134000	2.521748000
C	-2.313137000	1.574557000	1.615649000
C	-2.031477000	1.654708000	0.259185000
N	-0.817146000	1.957671000	-0.209179000
N	1.573010000	2.294859000	-1.263109000
C	2.740950000	2.529697000	-1.873142000
C	3.846796000	3.037838000	-1.206323000
C	3.735443000	3.329464000	0.145317000
C	2.532493000	3.075924000	0.786911000
Ni	-0.181650000	1.845837000	-2.141147000
C	0.589296000	1.905846000	-3.861620000
I	-2.456082000	2.149535000	-3.226157000
C	0.601523000	3.125444000	-4.550851000
C	1.391249000	3.297009000	-5.679688000
C	2.175525000	2.237729000	-6.153645000
C	2.160264000	1.013105000	-5.478166000
C	1.376930000	0.853647000	-4.339492000
C	2.996563000	2.411414000	-7.310989000
N	3.672437000	2.556045000	-8.247415000
H	0.788624000	2.322376000	2.731693000
H	-1.465822000	1.745634000	3.591701000
H	-3.314455000	1.315205000	1.944657000
H	-2.796031000	1.475455000	-0.497542000
H	2.782106000	2.305714000	-2.935710000
H	4.769477000	3.208142000	-1.751829000
H	4.572987000	3.749148000	0.695976000
H	2.415162000	3.296403000	1.843205000
H	-0.012090000	3.955154000	-4.205995000
H	1.401866000	4.248456000	-6.206907000
H	2.777550000	0.193610000	-5.839955000
H	1.436686000	-0.090482000	-3.801945000
C	-0.472942000	-0.598021000	-1.909096000
C	0.766935000	-0.995831000	-1.287539000
O	1.720602000	-1.533128000	-1.811174000
O	0.755847000	-0.746089000	0.088439000
C	1.689954000	-1.475149000	0.831712000
C	-0.871149000	-1.288185000	-3.170865000
C	-1.825246000	-2.487505000	-2.930468000
C	-2.053468000	-3.162105000	-4.280860000
C	-1.194074000	-3.490067000	-1.966426000
C	-3.168953000	-2.010352000	-2.383207000
H	-1.372742000	-0.586901000	-3.852301000
H	0.024636000	-1.663187000	-3.682890000
H	-2.738428000	-4.014119000	-4.179108000
H	-2.491820000	-2.461195000	-5.002969000
H	-1.110997000	-3.536382000	-4.701442000
H	-1.833630000	-4.375934000	-1.859586000
H	-1.056887000	-3.063981000	-0.962147000
H	-0.212265000	-3.822969000	-2.329026000
H	-3.628715000	-1.272419000	-3.053581000
H	-3.860464000	-2.857178000	-2.281263000
H	-3.074182000	-1.548730000	-1.391008000
H	2.102777000	-2.304948000	0.241110000
F	0.013797000	-2.905733000	1.591180000
C	0.939698000	-2.051766000	2.022759000
F	1.771550000	-2.705454000	2.830915000
F	0.327531000	-1.105655000	2.733343000
C	2.870613000	-0.604137000	1.233748000
F	3.401214000	-0.033074000	0.157441000
F	2.509791000	0.368308000	2.081680000
F	3.810728000	-1.333055000	1.827723000
H	-1.263076000	-0.328347000	-1.214205000

==

int3_HFIP_b

==

Charge: 0, Spin: 2
Cartesian coordinates:

ATOM	X	Y	Z
C	0.037074000	2.033031000	0.777541000
C	1.408251000	2.221020000	0.270979000
C	-0.303761000	2.058282000	2.128836000
C	-1.635287000	1.917706000	2.490810000
C	-2.597689000	1.756810000	1.500203000
C	-2.179353000	1.732123000	0.178282000
N	-0.898949000	1.862394000	-0.168045000
N	1.548050000	2.121153000	-1.063964000
C	2.733346000	2.370508000	-1.620144000
C	3.858601000	2.685706000	-0.869215000
C	3.733153000	2.756291000	0.511690000
C	2.491275000	2.531596000	1.089869000
Ni	-0.234790000	1.758857000	-2.099905000

C	0.204421000	1.554178000	-3.965710000
I	-0.715639000	4.336036000	-2.348764000
C	-0.751414000	1.926548000	-4.916228000
C	-0.494165000	1.773504000	-6.271149000
C	0.733257000	1.251480000	-6.697479000
C	1.685810000	0.868666000	-5.749781000
C	1.413057000	1.010088000	-4.393139000
C	1.005756000	1.098564000	-8.092562000
N	1.228328000	0.971314000	-9.227807000
H	0.458807000	2.186124000	2.891130000
H	-1.920079000	1.936357000	3.539395000
H	-3.650398000	1.651270000	1.741939000
H	-2.885385000	1.606083000	-0.643072000
H	2.769983000	2.324187000	-2.706239000
H	4.804696000	2.876971000	-1.365332000
H	4.589454000	3.001016000	1.134553000
H	2.366143000	2.614241000	2.165308000
H	-1.701505000	2.357867000	-4.607560000
H	-1.235655000	2.070730000	-7.009227000
H	2.627771000	0.436436000	-6.079736000
H	2.127299000	0.608283000	-3.678430000
C	-0.472858000	-0.284961000	-2.058742000
C	0.831036000	-0.760214000	-1.559373000
O	1.775938000	-1.139127000	-2.221997000
O	0.890846000	-0.714578000	-0.198080000
C	-1.009815000	-1.013456000	-3.270055000
C	-1.760481000	-2.325666000	-2.949801000
C	-2.129630000	-2.975781000	-4.282099000
C	-0.883961000	-3.289603000	-2.152382000
C	-3.046340000	-2.036924000	-2.175027000
H	-1.708170000	-0.366905000	-3.823078000
H	-0.182609000	-1.233203000	-3.959773000
H	-2.693345000	-3.904576000	-4.122909000
H	-2.750280000	-2.305664000	-4.891694000
H	-1.231319000	-3.221555000	-4.862822000
H	-1.397868000	-4.250432000	-2.015786000
H	-0.651071000	-2.899732000	-1.152016000
H	0.064772000	-3.481491000	-2.669802000
H	-3.690674000	-1.339701000	-2.728712000
H	-3.616127000	-2.961782000	-2.014902000
H	-2.849182000	-1.604447000	-1.184633000
H	-1.197388000	-0.283402000	-1.239162000
C	2.141178000	-1.093174000	0.355488000
H	2.042013000	-0.977816000	1.437881000
H	2.383373000	-2.131430000	0.105528000
H	2.949365000	-0.452912000	-0.019779000

===

TS3_HFIP_b

===

Charge: 0, Spin: 2

Imaginary frequency: –247.2025 cm^{-1}

Cartesian coordinates:

ATOM	X	Y	Z
C	0.210962000	2.144238000	0.868352000
C	1.427268000	2.637722000	0.192913000
C	0.042046000	2.146255000	2.252684000
C	-1.166510000	1.720640000	2.783573000
C	-2.180216000	1.312510000	1.924893000
C	-1.929893000	1.334541000	0.559801000
N	-0.764815000	1.726553000	0.049052000
N	1.462383000	2.481106000	-1.143758000
C	2.475315000	3.006590000	-1.837469000
C	3.532162000	3.670589000	-1.233557000
C	3.525635000	3.801274000	0.149545000
C	2.457216000	3.289639000	0.869057000
Ni	-0.237091000	1.722912000	-2.004254000
C	0.363548000	1.045214000	-3.738684000
I	-1.578776000	3.919178000	-2.517116000
C	-0.420343000	1.396126000	-4.848239000
C	0.145290000	1.487282000	-6.107288000
C	1.506344000	1.203968000	-6.292460000
C	2.286124000	0.828505000	-5.196150000
C	1.713129000	0.736403000	-3.933623000
C	2.091681000	1.289371000	-7.593032000
N	2.571857000	1.356492000	-8.651016000
H	0.845444000	2.463429000	2.910827000
H	-1.316167000	1.711115000	3.859943000
H	-3.144852000	0.985160000	2.299795000
H	-2.694587000	1.043743000	-0.162337000
H	2.425430000	2.883183000	-2.917975000
H	4.333797000	4.079310000	-1.840430000
H	4.335687000	4.312683000	0.662714000
H	2.418344000	3.408764000	1.947405000
H	-1.470001000	1.650398000	-4.718119000
H	-0.459364000	1.793810000	-6.957182000
H	3.338045000	0.593063000	-5.338885000
H	2.337571000	0.415703000	-3.103212000
C	-0.477598000	-0.340082000	-2.396676000
C	0.672978000	-1.046750000	-1.793323000
O	1.462906000	-1.763351000	-2.355062000
O	0.757733000	-0.802418000	-0.434004000
C	1.755050000	-1.507852000	0.250575000
C	-1.180620000	-1.146291000	-3.477743000
C	-2.087611000	-2.287034000	-2.965710000
C	-2.711766000	-2.933914000	-4.202432000
C	-1.311284000	-3.352245000	-2.193339000
C	-3.207131000	-1.736723000	-2.080564000
H	-1.822881000	-0.470671000	-4.058279000
H	-0.432939000	-1.550495000	-4.175467000
H	-3.370746000	-3.763769000	-3.916985000
H	-3.310212000	-2.210027000	-4.771004000
H	-1.939079000	-3.332701000	-4.872233000

H	-1.976267000	-4.186444000	-1.934208000
H	-0.903162000	-2.968671000	-1.248195000
H	-0.478728000	-3.751030000	-2.785843000
H	-3.717848000	-0.888399000	-2.558657000
H	-3.957479000	-2.513850000	-1.887030000
H	-2.835159000	-1.406296000	-1.100992000
H	2.144398000	-2.330804000	-0.364254000
F	0.207967000	-3.014318000	1.121751000
C	1.094425000	-2.090307000	1.489946000
F	1.999595000	-2.664628000	2.279104000
F	0.450689000	-1.162853000	2.193555000
C	2.926302000	-0.584184000	0.566194000
F	3.247459000	0.127748000	-0.514122000
F	2.636000000	0.273051000	1.549106000
F	3.990608000	-1.288318000	0.936167000
H	-1.206119000	-0.125713000	-1.600053000
H	2.646657000	1.966476000	-0.503408000
H	1.676906000	3.244251000	-1.258787000
H	0.879006000	1.983363000	-0.319698000
O	-1.713952000	-0.863522000	-1.174875000
C	-2.942793000	-1.208951000	-1.769718000
H	-2.820328000	-1.387219000	-2.845653000
C	-3.421744000	-2.503065000	-1.128957000
F	-2.470215000	-3.427001000	-1.262180000
F	-3.675837000	-2.350362000	0.165381000
F	-4.524171000	-2.940719000	-1.729953000
C	-3.876999000	-0.027525000	-1.577434000
F	-3.379614000	1.027615000	-2.224500000
F	-5.077774000	-0.303721000	-2.078807000
F	-4.015559000	0.298255000	-0.298781000

==

7e

==

Charge: 0, Spin: 1

Cartesian coordinates:

ATOM	X	Y	Z
C	1.112789000	-2.077381000	-0.489463000
C	1.179703000	-3.265380000	-1.221714000
C	1.664616000	-4.425473000	-0.636647000
C	2.095221000	-4.409003000	0.693882000
C	2.034603000	-3.221926000	1.431819000
C	1.544273000	-2.069318000	0.838341000
C	2.596668000	-5.603729000	1.300976000
N	3.006077000	-6.573641000	1.795415000
H	0.842379000	-3.281090000	-2.256204000
H	1.713026000	-5.351622000	-1.203259000
H	2.368535000	-3.215492000	2.466034000
H	1.495253000	-1.144478000	1.411154000
C	0.639265000	-0.783397000	-1.124786000
C	-0.599155000	-1.076732000	-1.930233000
O	-0.637574000	-1.488858000	-3.060293000
C	1.739053000	-0.153966000	-1.988018000
C	1.580258000	1.343515000	-2.300484000
C	2.716362000	1.731901000	-3.246009000
C	0.245353000	1.644963000	-2.980709000
C	1.700811000	2.172921000	-1.022239000
H	0.351972000	-0.100840000	-0.314263000
H	2.697739000	-0.297509000	-1.467167000
H	1.803219000	-0.726451000	-2.925697000
H	2.673984000	2.801459000	-3.487955000
H	3.696065000	1.528909000	-2.793601000
H	2.656509000	1.171122000	-4.187692000
H	0.191558000	2.707791000	-3.249132000
H	-0.615710000	1.442084000	-2.327752000
H	0.121835000	1.057798000	-3.899151000

8. References

[1] R. L. Jezorek, N. Zhang, P. Leowanawat, M. H. Bunner, N. Gutsche, A. K. R. Pesti, J. T. Olsen, V. Percec, *Org. Lett.* **2014**, *16*, 6326–6329.

[2] J. H. Jeon, G. H. Kim, H. S. Lee, D. H. Kim, S. Lee, W. Choe, B. Jung, J.-U. Rohde, S. Y. Hong, *ACS Catal.* **2024**, *14*, 8996–9007.

[3] X. Shi, Q. Wang, Z. Tang, H. Huang, T. Cao, H. Cao, X. Liu, *Org. Lett.* **2024**, *26*, 1255–1260.

[4] H.-H. Deng, S.-Y. Tian, J.-H. Han, X.-Y. Liu, W. Rao, S.-S. Shen, D. Sheng, Z.-Y. Yang, S.-Y. Wang, *J. Org. Chem.* **2024**, *89*, 8804–8814.

[5] H. Hou, B. Zhou, J. Wang, D. Zhao, D. Sun, X. Chen, Y. Han, C. Yan, Y. Shi, S. Zhu, *Org. Lett.* **2021**, *23*, 2981–2987.

[6] H. Shen, H. Xiao, L. Zhu, C. Li, *Synlett* **2020**, *31*, 41–44.

[7] K.-F. Zhang, K.-J. Bian, C. Li, J. Sheng, Y. Li, X.-S. Wang, *Angew. Chem. Int. Ed.* **2019**, *58*, 5069–5074.

[8] Y. Sun, N. Zhang, J. Ren, H. Huang, X. Luan, Z. Zuo, *Org. Lett.* **2023**, *26*, 35–40.

[9] Y.-L. Shao, X.-H. Zhang, J.-S. Han, P. Zhong, *Org. Lett.* **2012**, *14*, 5242–5245.

[10] M. Berthet, A. Beauseigneur, C. Moine, C. Taillier, M. Othman, V. Dalla, *Chem. Eur. J.* **2018**, *24*, 1278–1282.

[11] Z.-X. Wang, K. Livingstone, C. Hümpel, C. G. Daniliuc, C. Mück-Lichtenfeld, R. Gilmour, *Nat. Chem.* **2023**, *15*, 1515–1522.

[12] V. G. Landge, A. J. Grant, Y. Fu, A. M. Rabon, J. L. Payton, M. C. Young, *J. Am. Chem. Soc.* **2021**, *143*, 10352–10360.

[13] V. R. Yatham, Y. Shen, R. Martin, *Angew. Chem. Int. Ed.* **2017**, *56*, 10915–10919.

[14] Q. Lin, T. Diao, *J. Am. Chem. Soc.* **2019**, *141*, 17937–17948.

[15] R. G. Parr, W. Yang, *Density Functional Theory of Atoms and Molecules*. Oxford University Press: New York, **1989**

[16] M. J. Frisch, G. W. Trucks, H. B. Schlegel, G. E. Scuseria, M. A. Robb, J. R. Cheeseman, G. Scalmani, V. Barone, G. A. Petersson, H. Nakatsuji, X. Li, M. Caricato, A. V. Marenich, J. Bloino, B. G. Janesko, R. Gomperts, B. Mennucci, H. P. Hratchian, J. V. Ortiz, A. F. Izmaylov, J. L. Sonnenberg, D. Williams-Young, F. Ding, F. Lipparini, F. Egidi, J. Goings, B. Peng, A. Petrone, T. Henderson, D. Ranasinghe, V. G. Zakrzewski, J. Gao, N. Rega, G. Zheng, W. Liang, M. Hada, M. Ehara, K. Toyota, R. Fukuda, J. Hasegawa, M. Ishida, T. Nakajima, Y. Honda, O. Kitao, H. Nakai, T. Vreven, K. Throssell, J. A. Montgomery, Jr., J. E. Peralta, F. Ogliaro, M. J. Bearpark, J. J. Heyd, E. N. Brothers, K. N. Kudin, V. N. Staroverov, T. A. Keith, R. Kobayashi, J. Normand, K. Raghavachari, A. P. Rendell, J. C. Burant, S. S. Iyengar, J. Tomasi, M. Cossi, J. M. Millam, M. Klene, C. Adamo, R. Cammi, J. W. Ochterski, R. L. Martin, K. Morokuma,

O. Farkas, J. B. Foresman, D. J. Fox, Gaussian 16, revision C.01, Gaussian, Inc.: Wallingford, CT, **2016**.

[17] Y. Zhao, D. G. Truhlar, *Theor. Chem. Acc.* **2008**, *120*, 215–241.

[18] W. R. Wadt, P. J. Hay, *J. Chem. Phys.* **1985**, *82*, 284–298.

[19] P. J. Hay, W. R. Wadt, *J. Chem. Phys.* **1985**, *82*, 270–283.

[20] P. J. Hay, W. R. Wadt, *J. Chem. Phys.* **1985**, *82*, 299–310.

[21] R. Ditchfield, W. J. Hehre, J. A. Pople, *J. Chem. Phys.* **1971**, *54*, 724–728.

[22] A. V. Marenich, C. J. Cramer, D. G. Truhlar, *J. Phys. Chem. B.***2009**, *113*, 6378–6396.

9. NMR Spectra

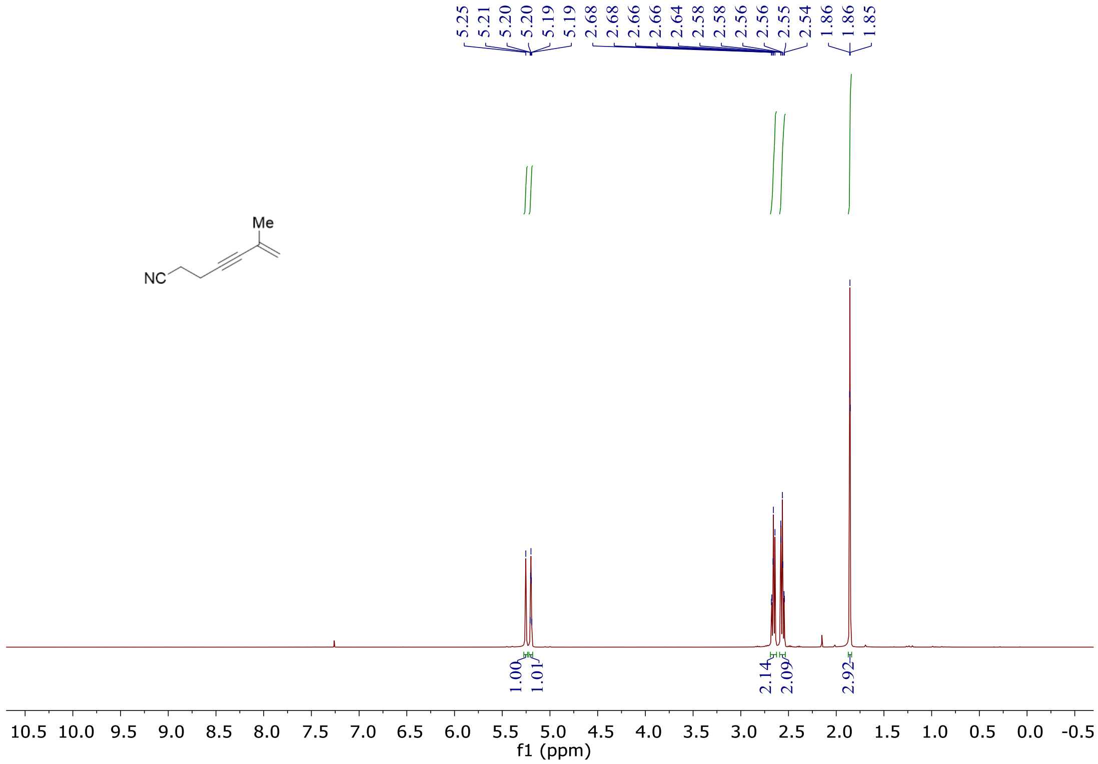

^{1}H NMR (400 MHz, $CDCl_3$) of **S14**

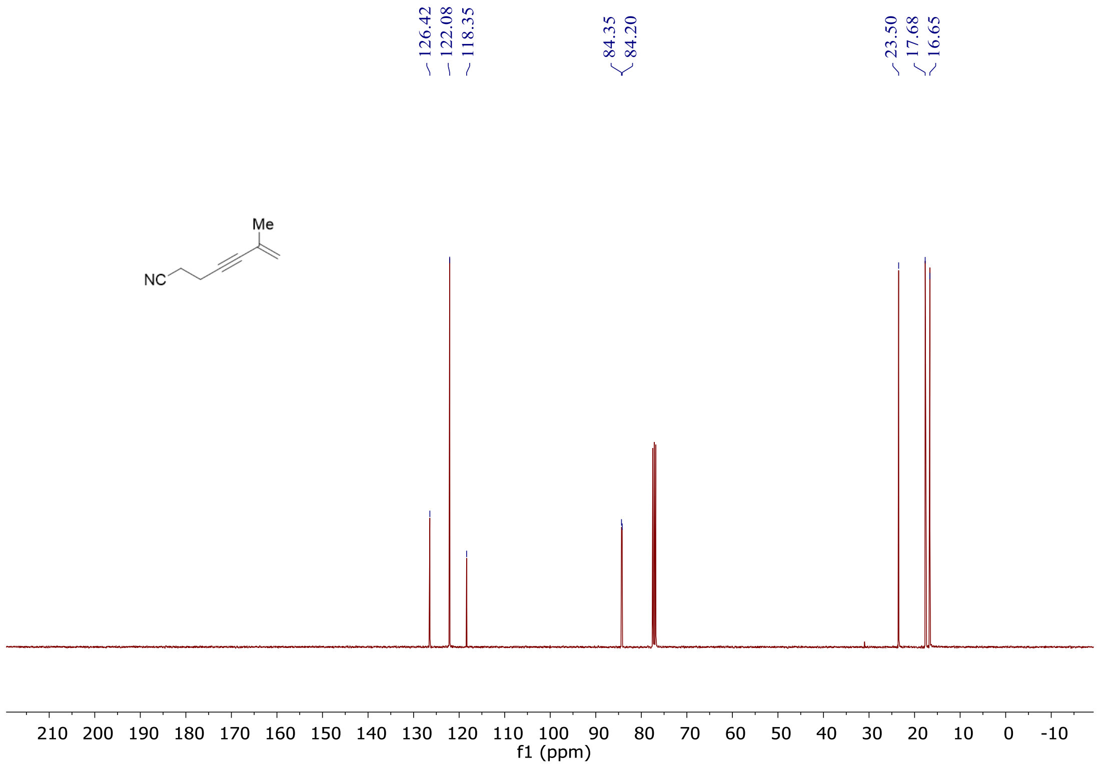

^{13}C{^{1}H} NMR (100 MHz, $CDCl_3$) of **S14**

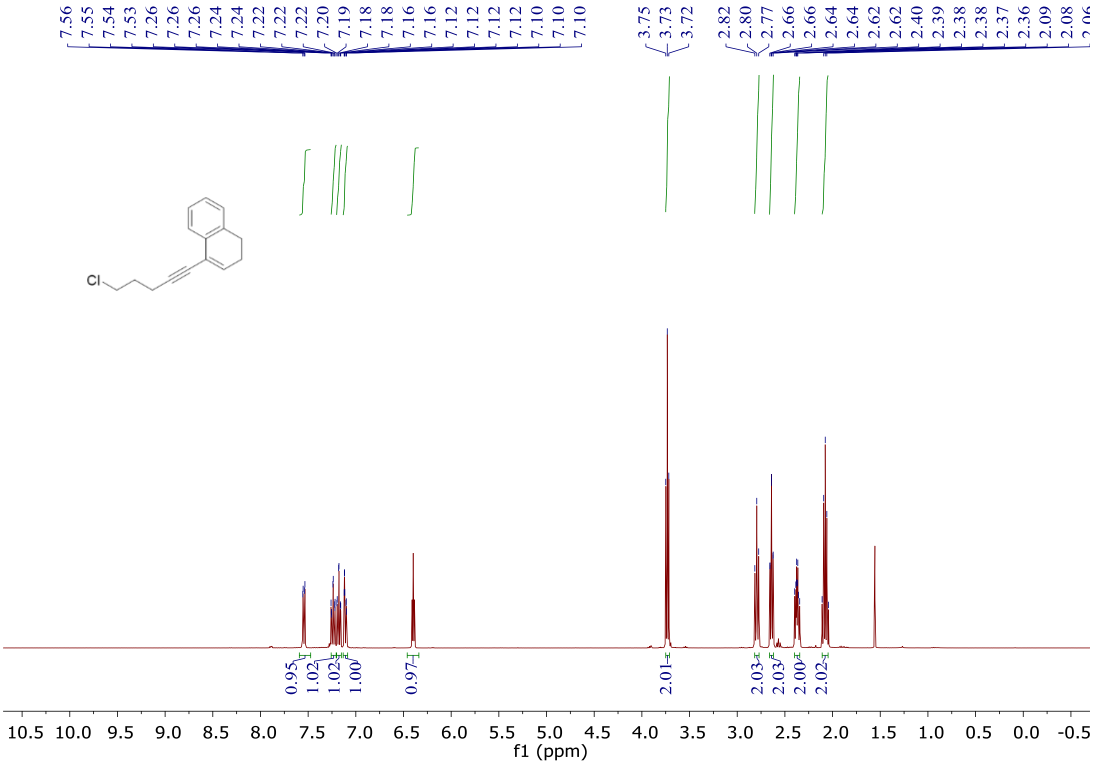

^{1}**H NMR** (400 MHz, $CDCl_3$) of **S19**

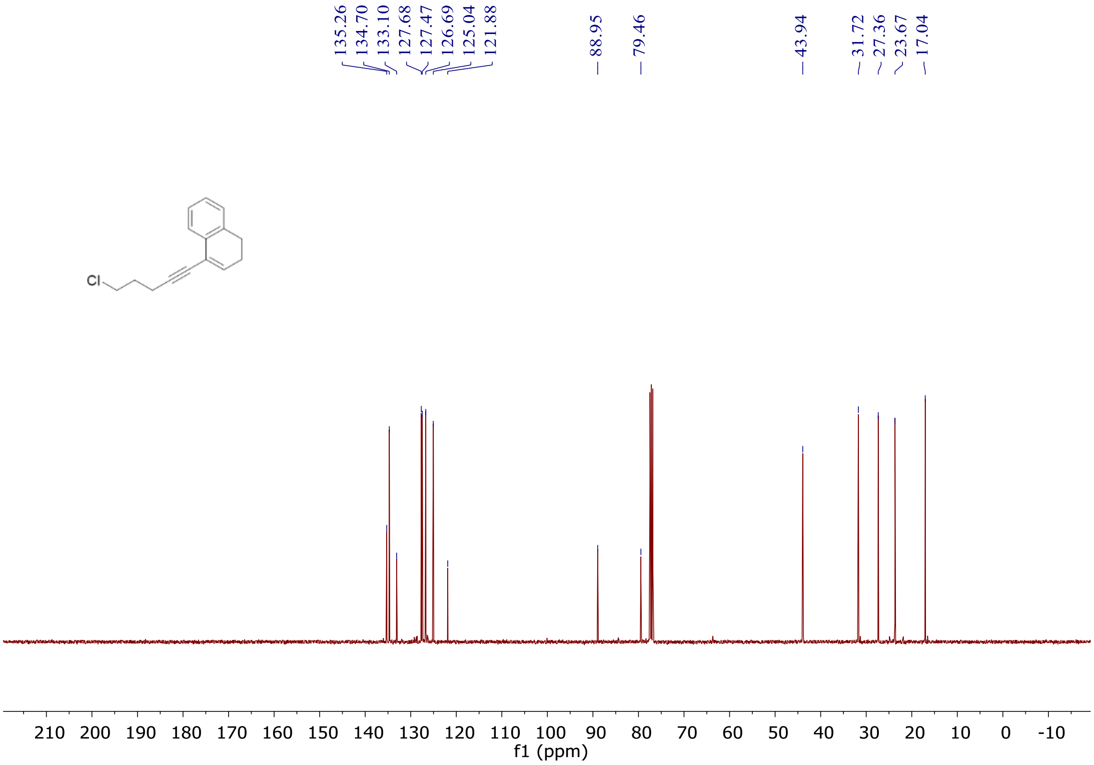

^{13}**C{^{1}H} NMR** (100 MHz, $CDCl_3$) of **S19**

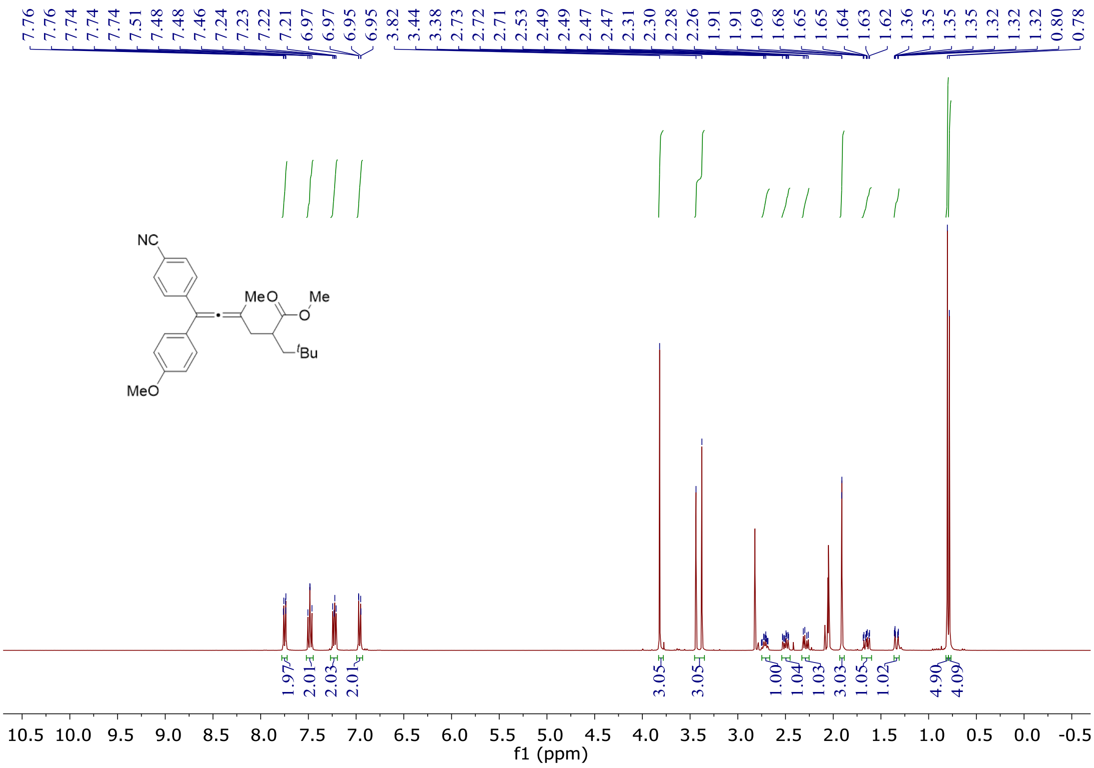

^{1}H NMR (400 MHz, acetone-d_6) of **5ad**

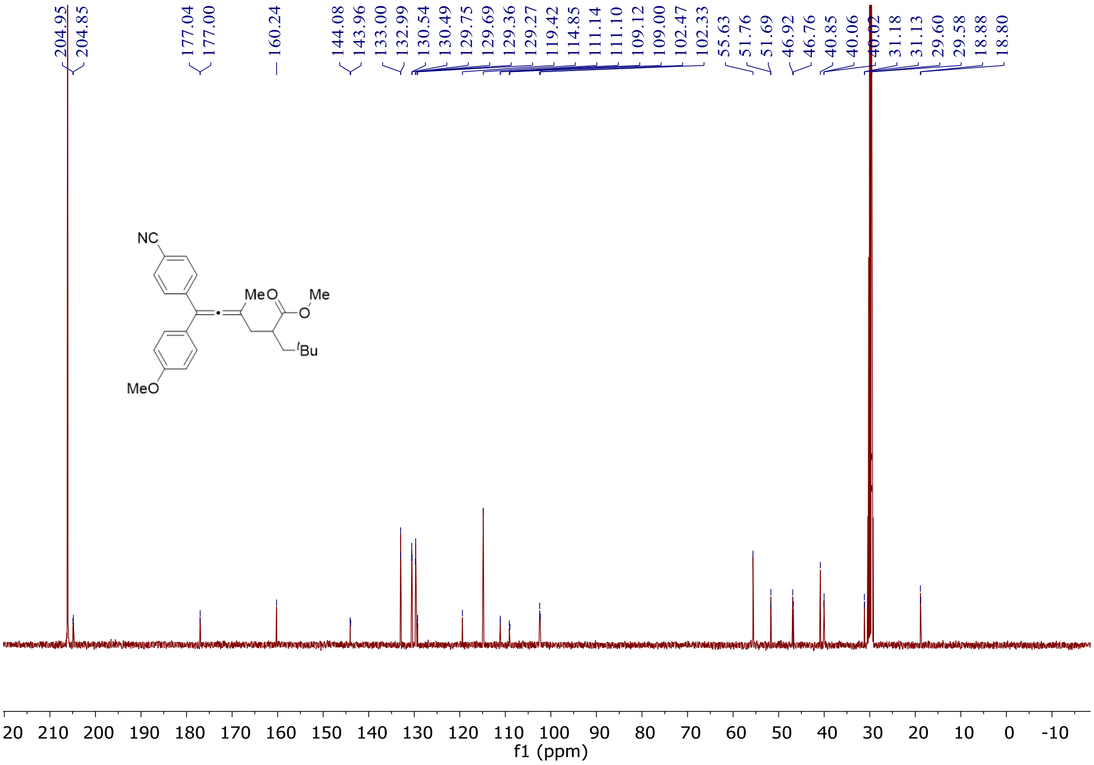

^{13}C{^{1}H} NMR (100 MHz, acetone-d_6) of **5ad**

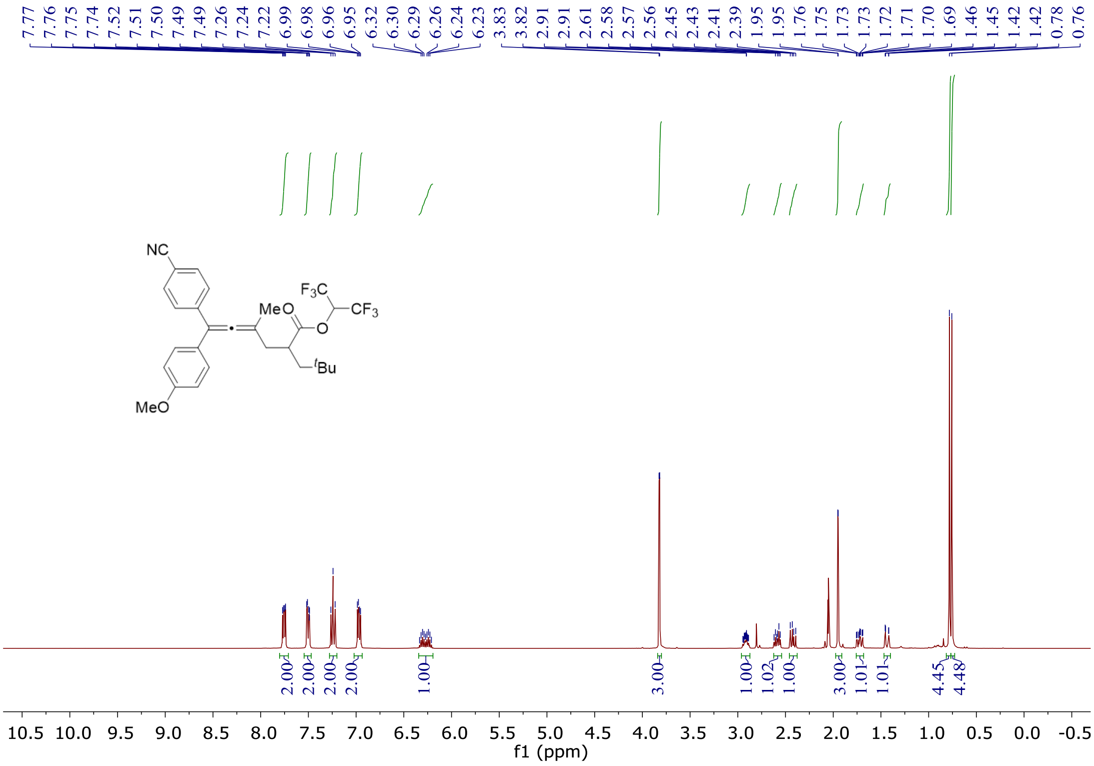

^{1}H NMR (400 MHz, acetone-d_6) of **5ae**

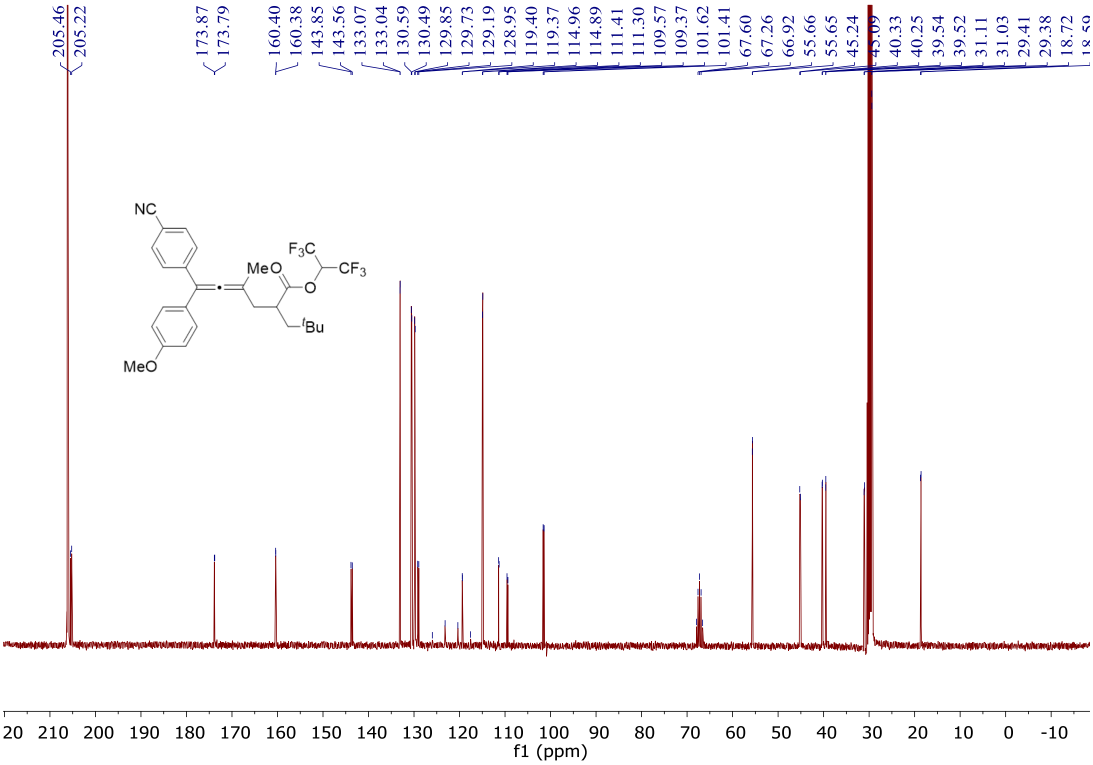

^{13}C{^{1}H} NMR (100 MHz, acetone-d_6) of **5ae**

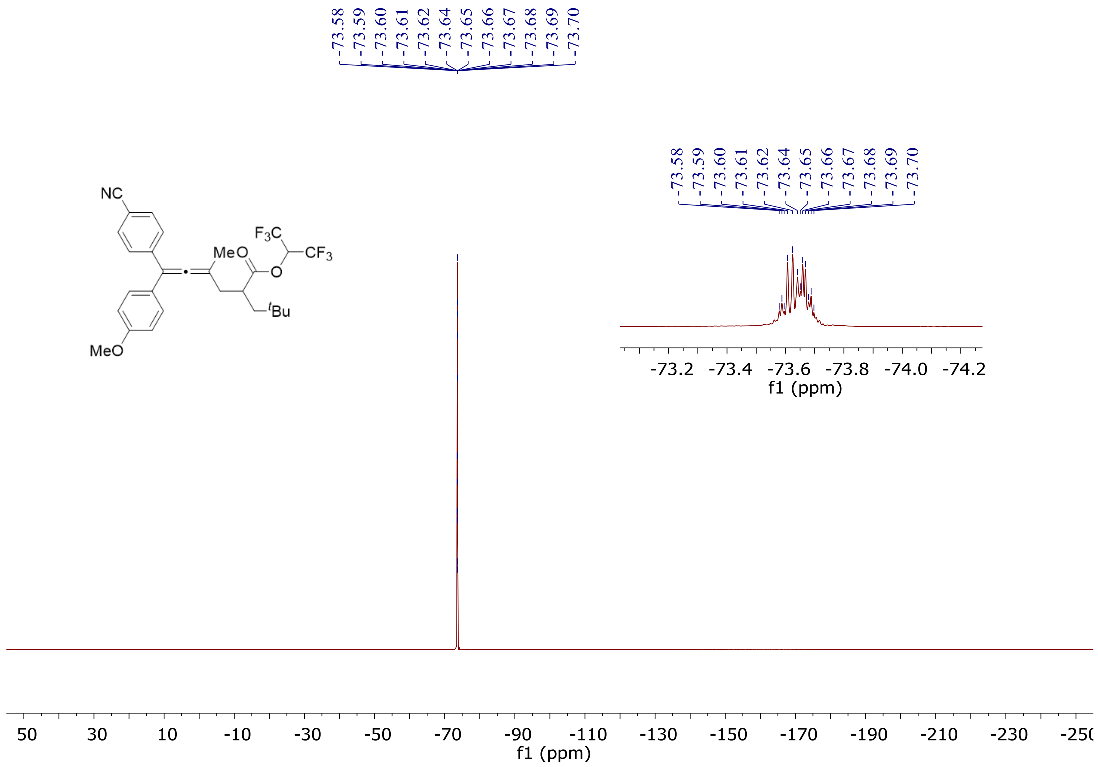

^{19}**F{^{1}H} NMR** (377 MHz, acetone-d_6) of **5ae**

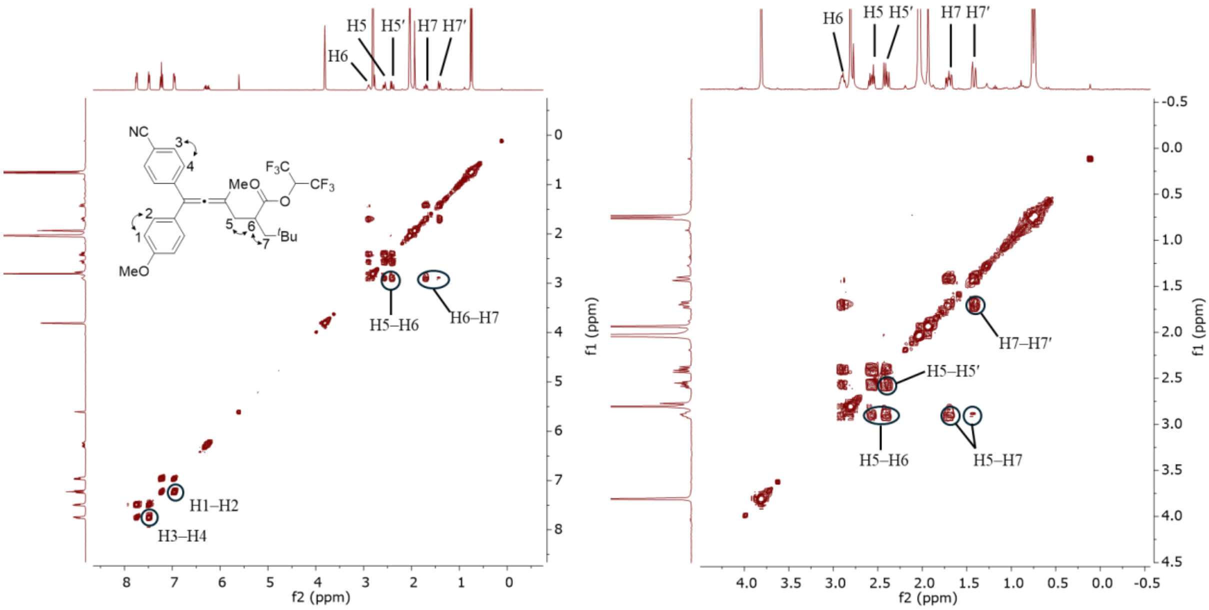

COSY NMR (400 MHz, acetone-d_6) of **5ae**

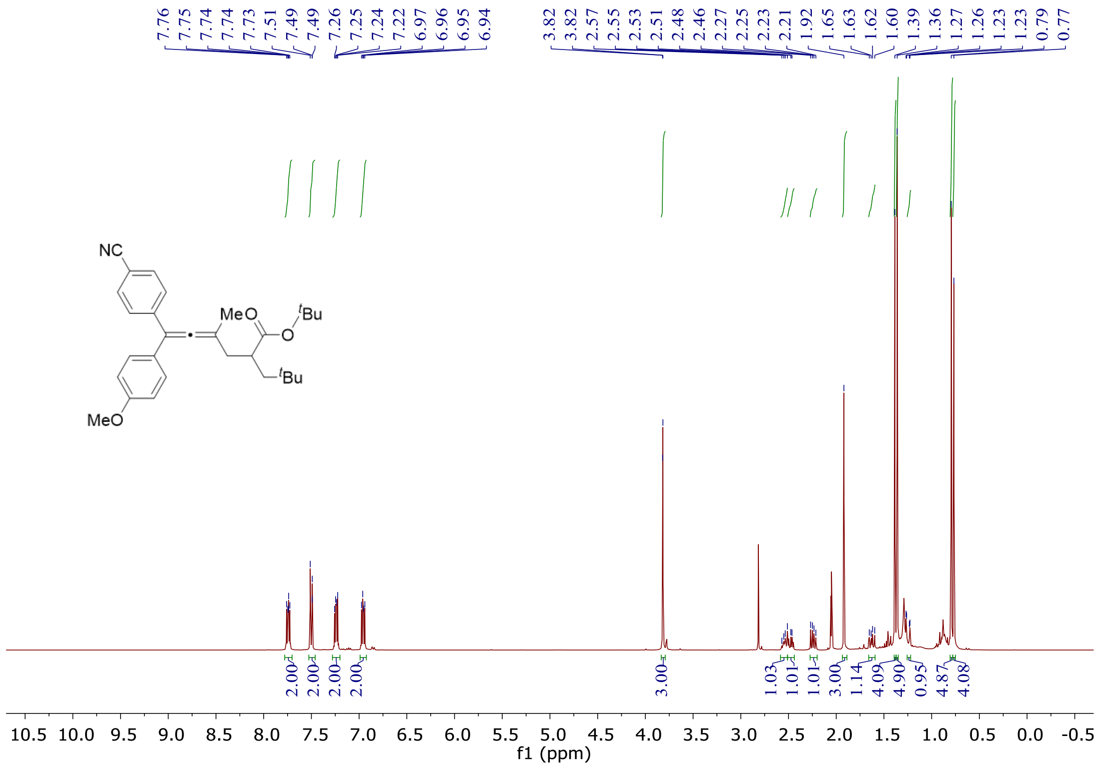

^{1}H NMR (400 MHz, acetone-d_6) of **5af**

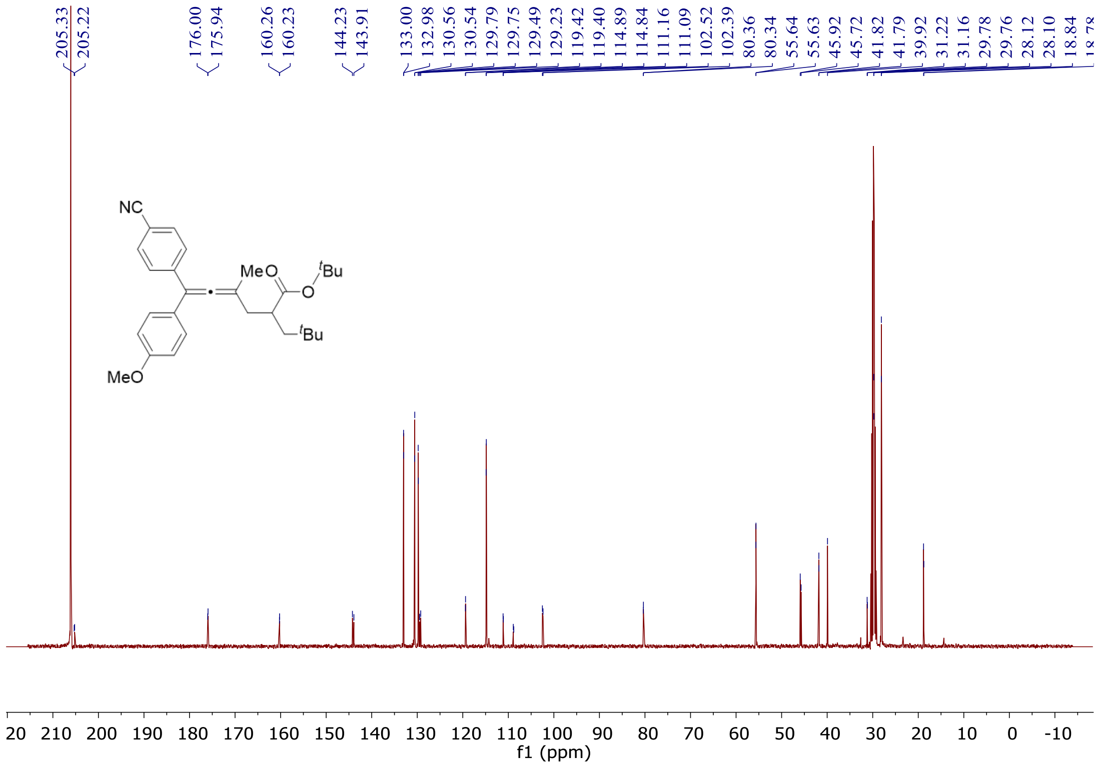

^{13}C{^{1}H} NMR (100 MHz, acetone-d_6) of **5af**

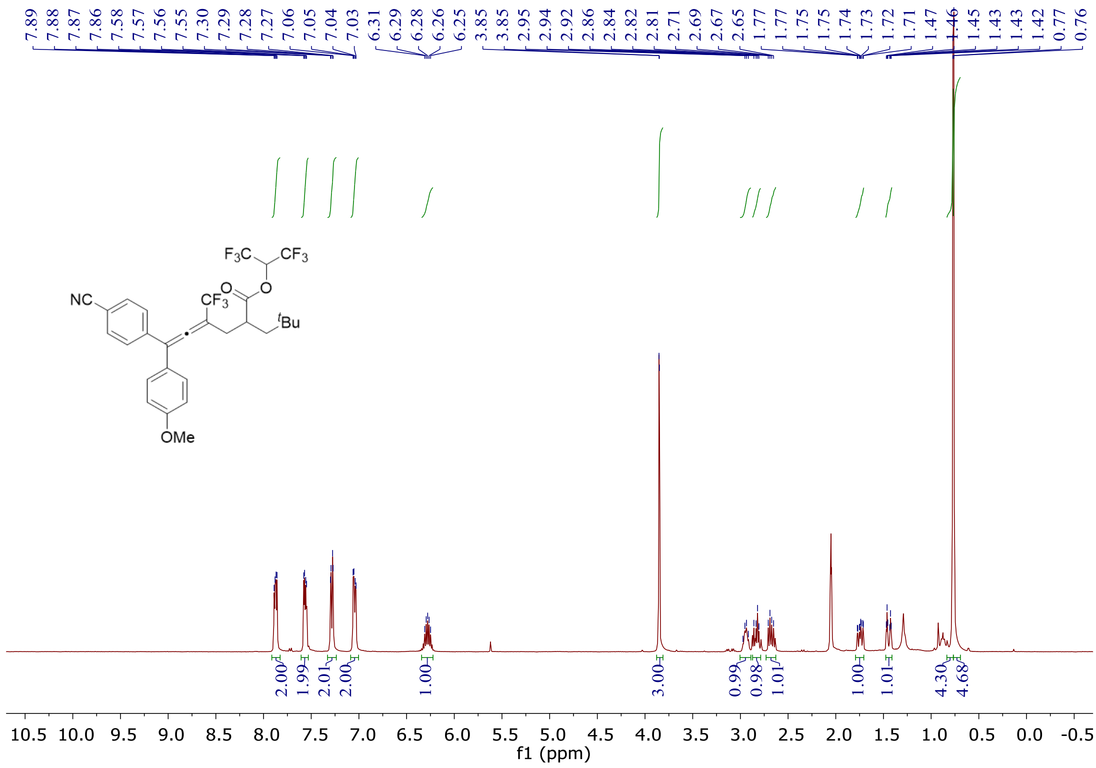

^{1}H NMR (400 MHz, acetone-d_6) of **5be**

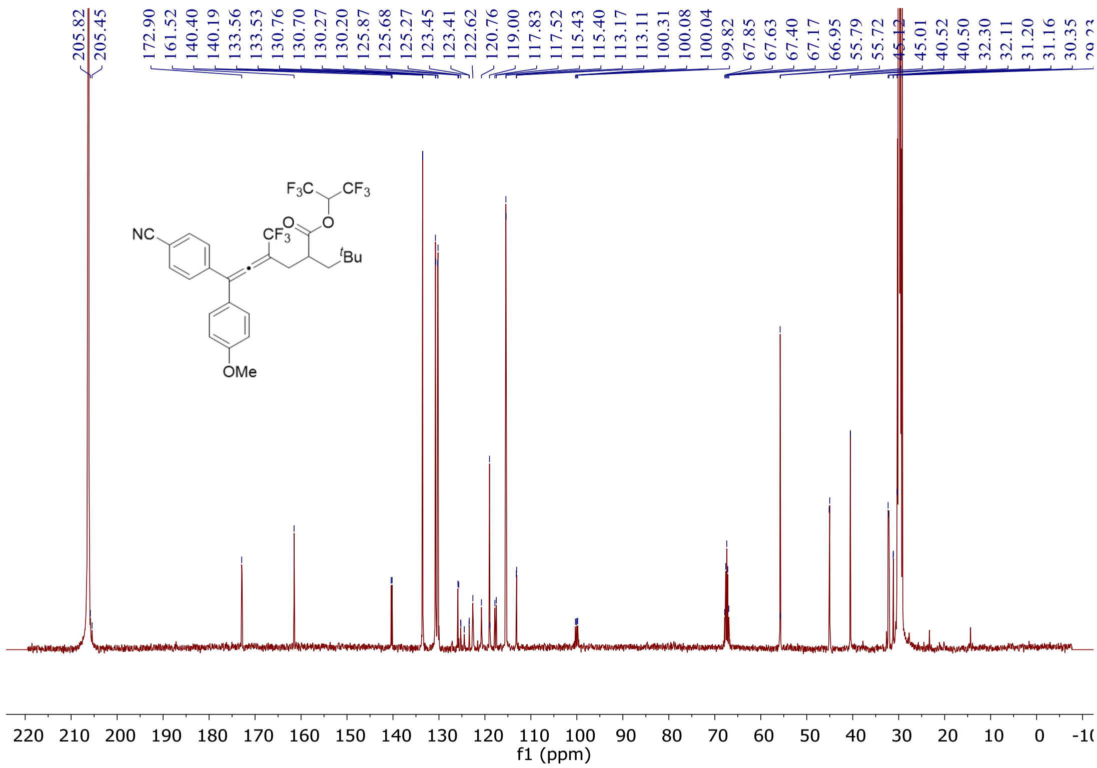

^{13}C{^{1}H} NMR (100 MHz, acetone-d_6) of **5be**

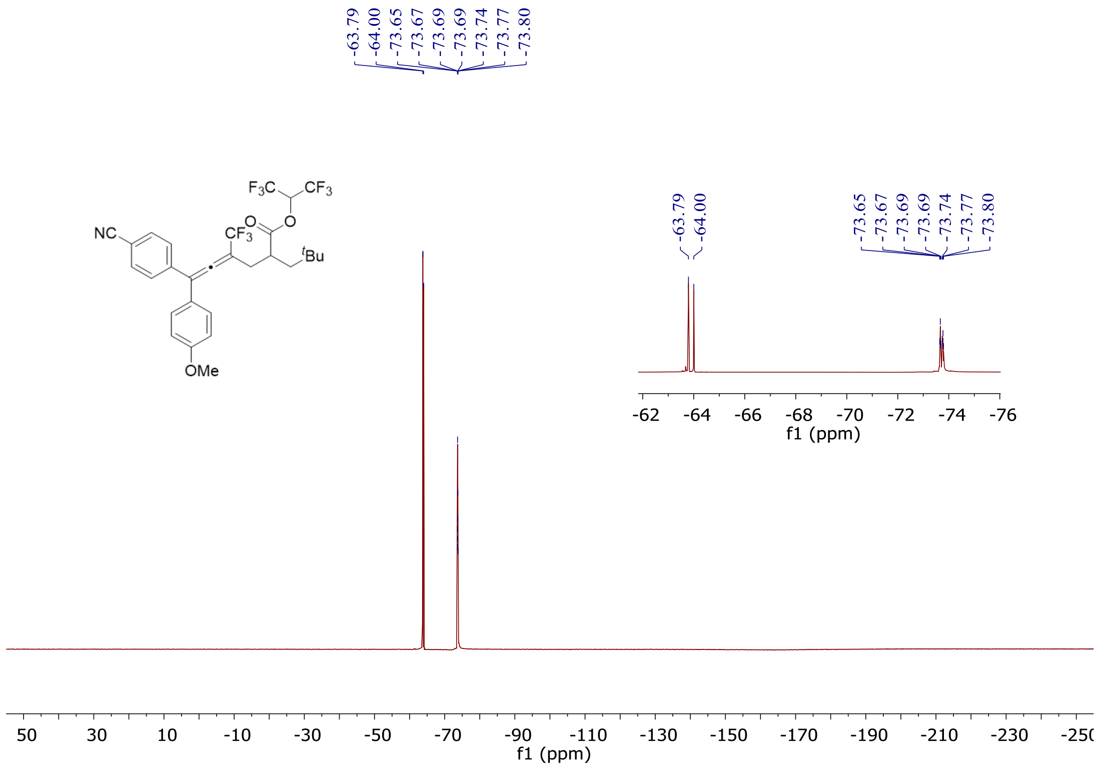

^{19}F{^{1}H} NMR (377 MHz, acetone-d_6) of **5be**

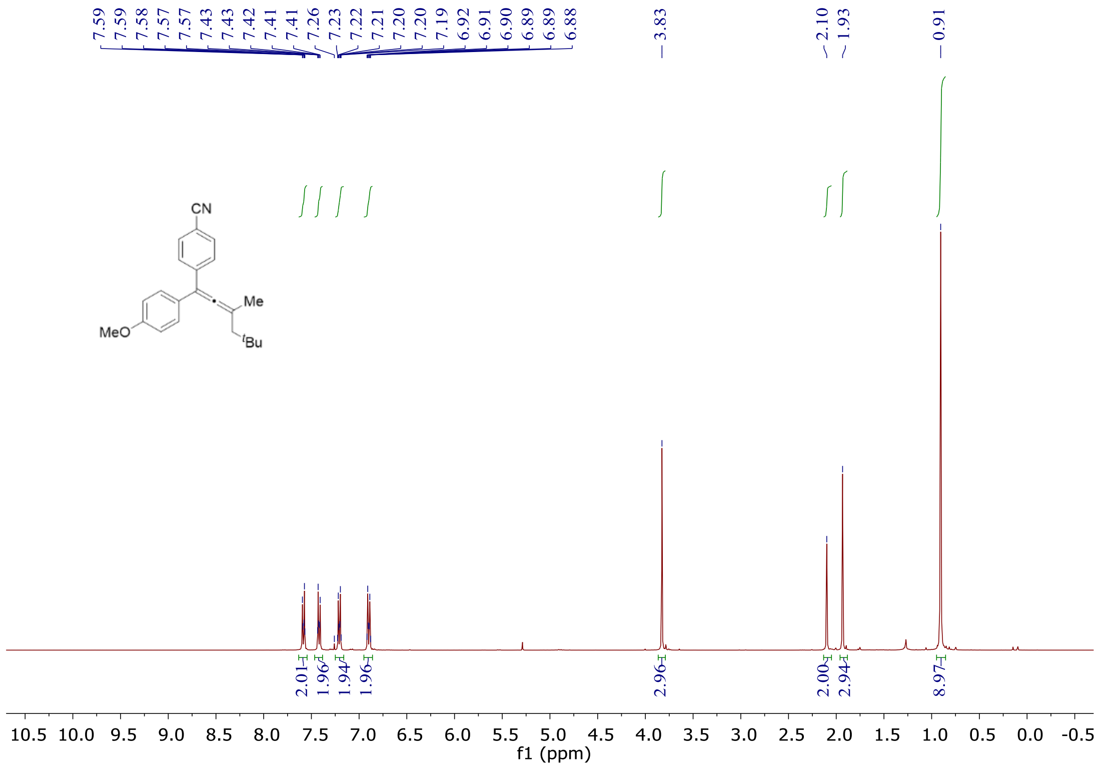

^{1}H NMR (400 MHz, $CDCl_3$) of **6a**

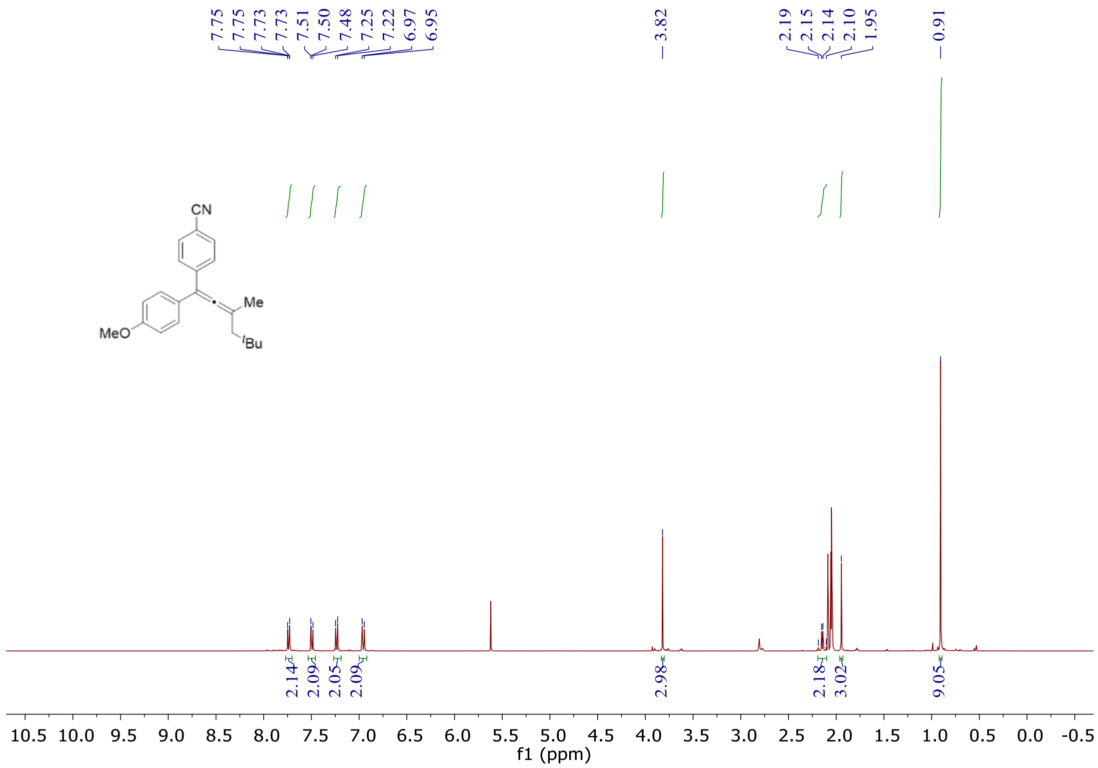

^{1}**H NMR** (400 MHz, acetone-d_6) of **6a**

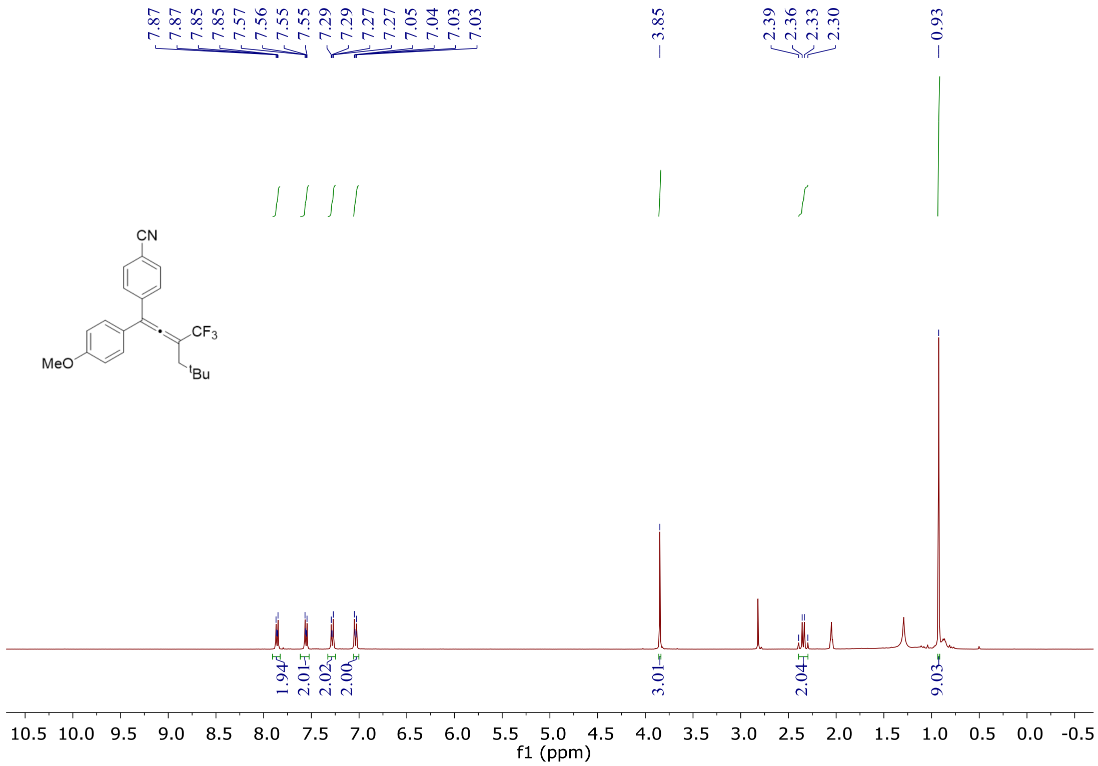

^{1}**H NMR** (400 MHz, acetone-d_6) of **6b**

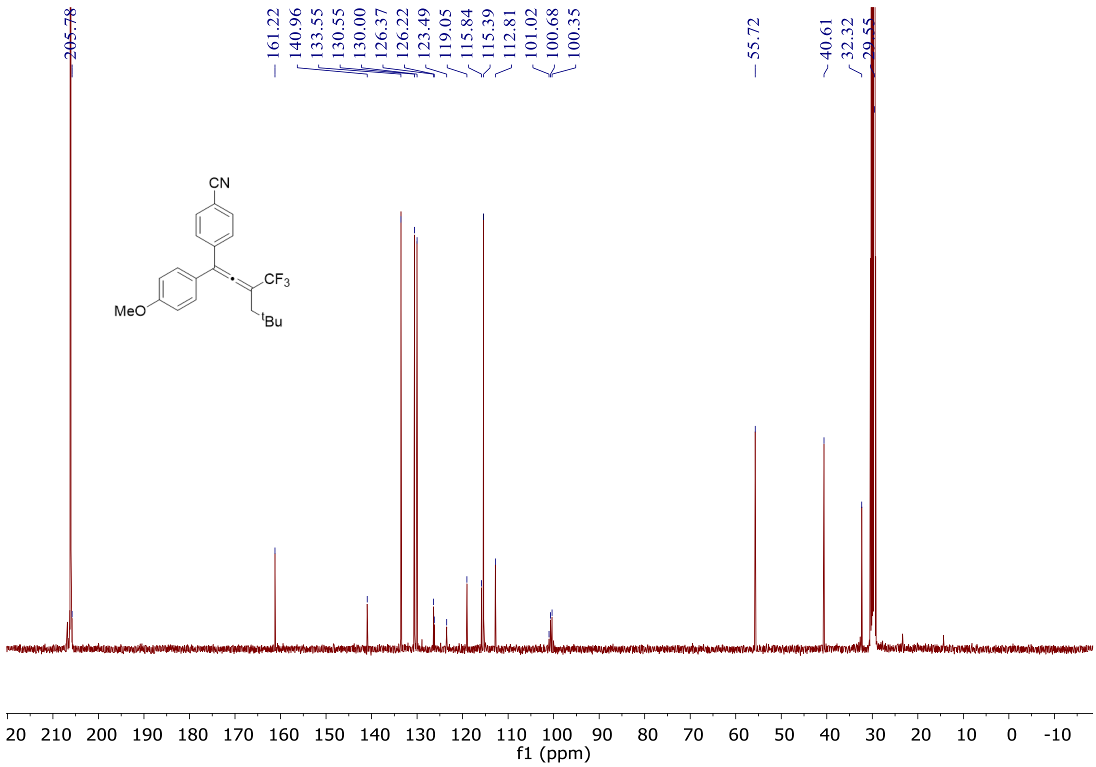

$^{13}C\{^1H\}$ NMR (100 MHz, acetone-d_6) of **6b**

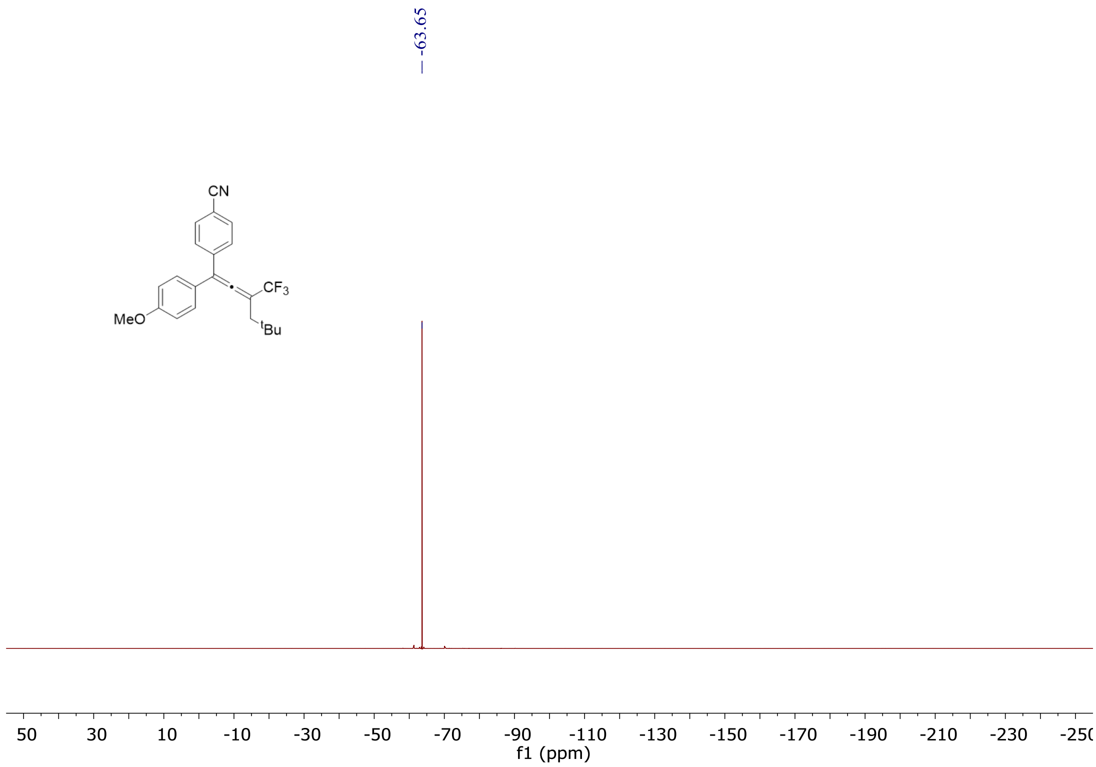

$^{19}F\{^1H\}$ NMR (377 MHz, acetone-d_6) of **6b**

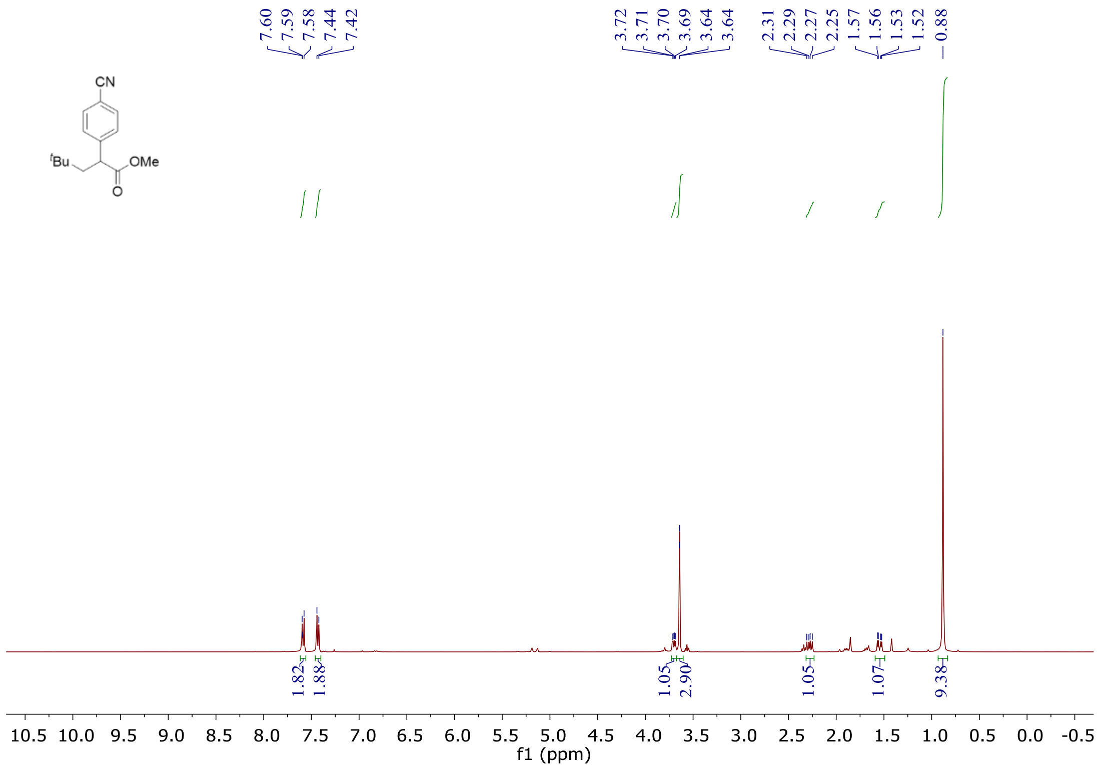

^{1}H NMR (400 MHz, $CDCl_3$) of **7d**

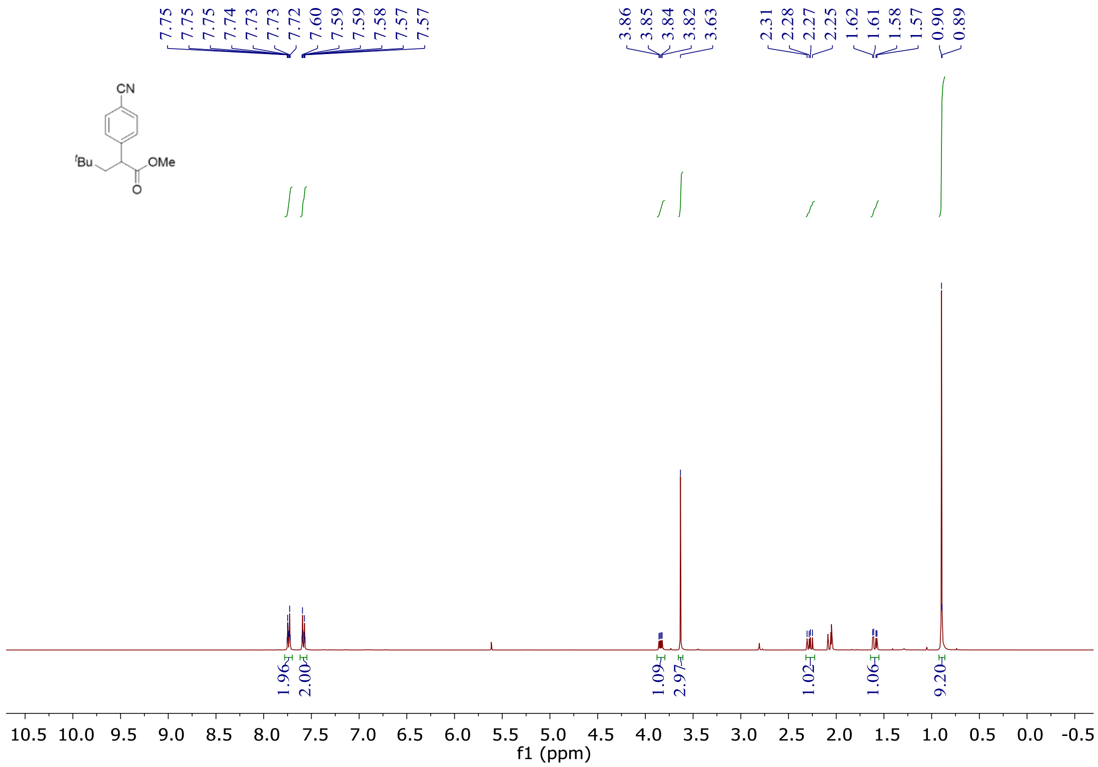

^{1}H NMR (400 MHz, acetone-d_6) of **7d**

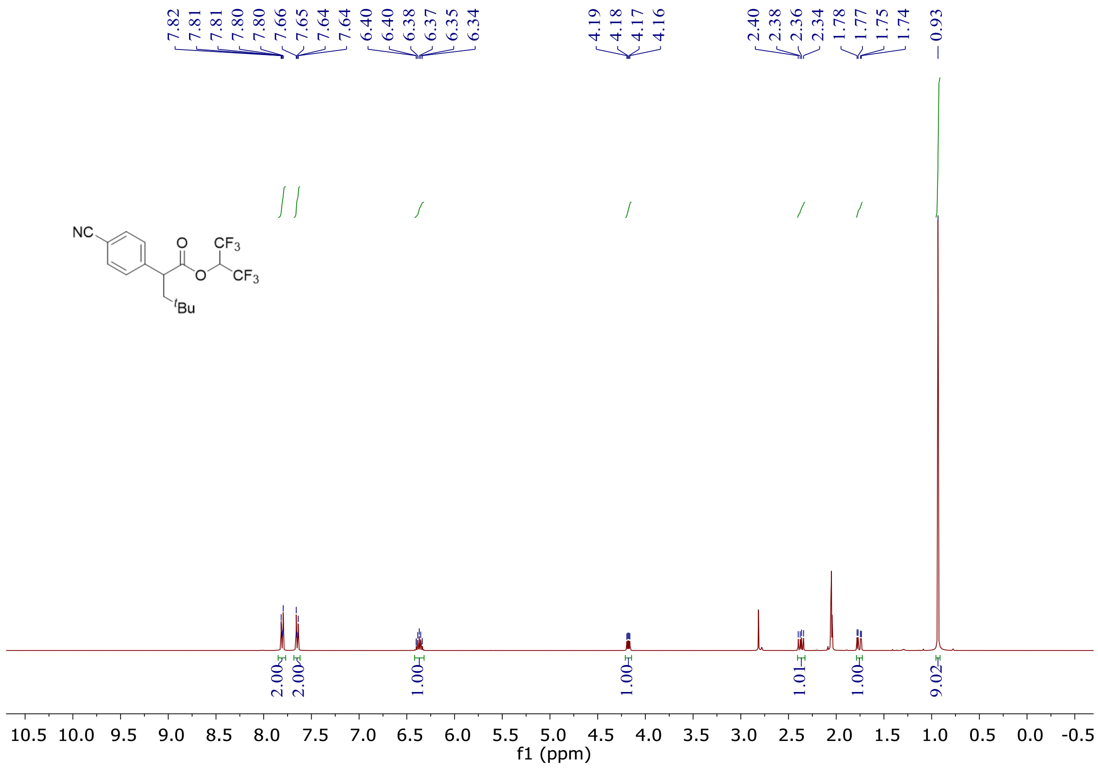

^{1}H NMR (400 MHz, acetone-d_6) of **7e**

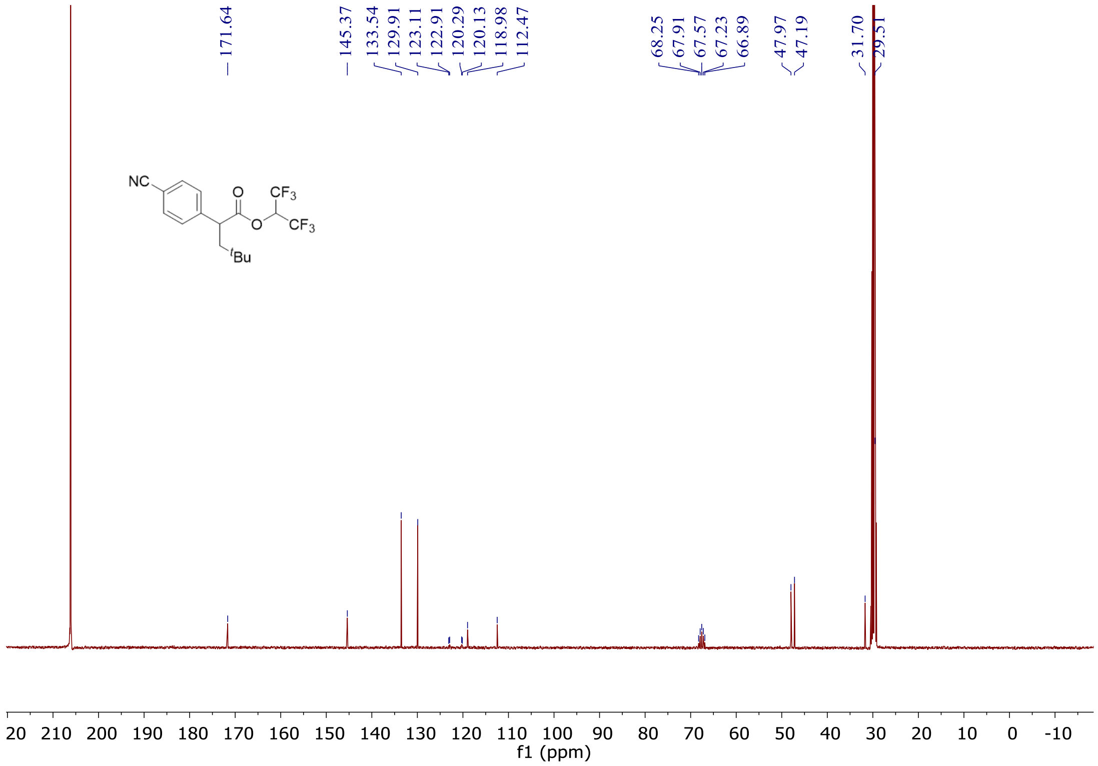

^{13}C{^{1}H} NMR (100 MHz, acetone-d_6) of **7e**

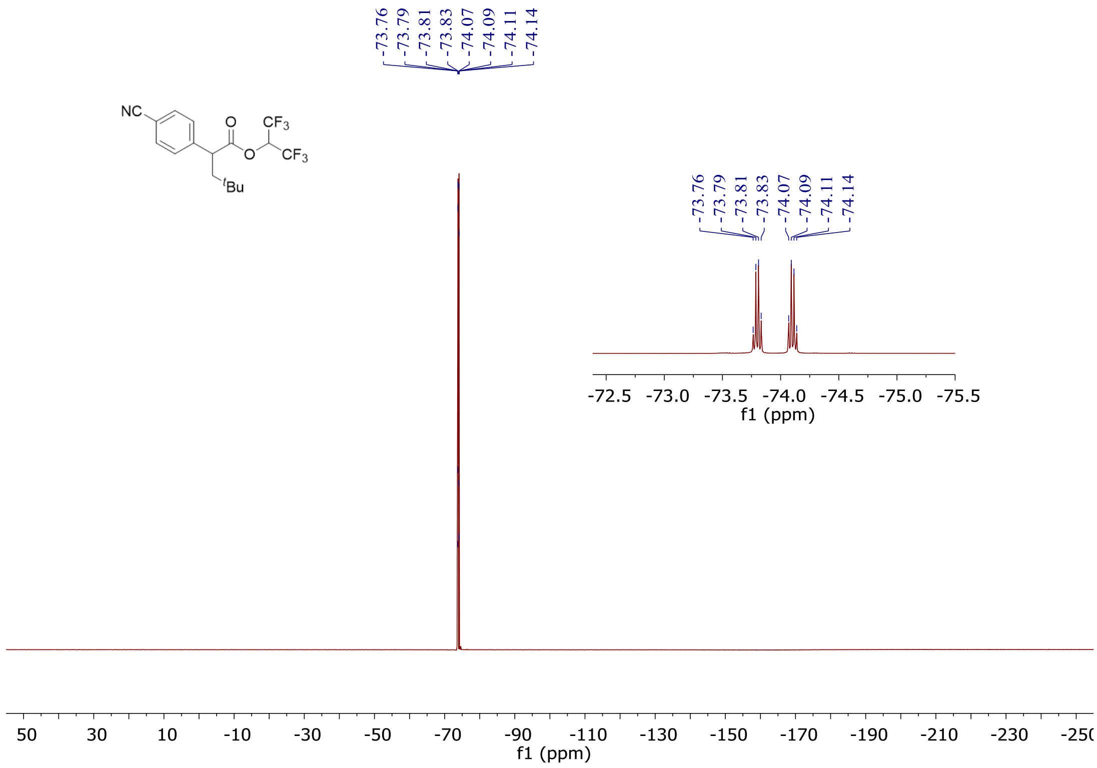

^{19}F{^{1}H} NMR (377 MHz, acetone-d_6) of **7e**

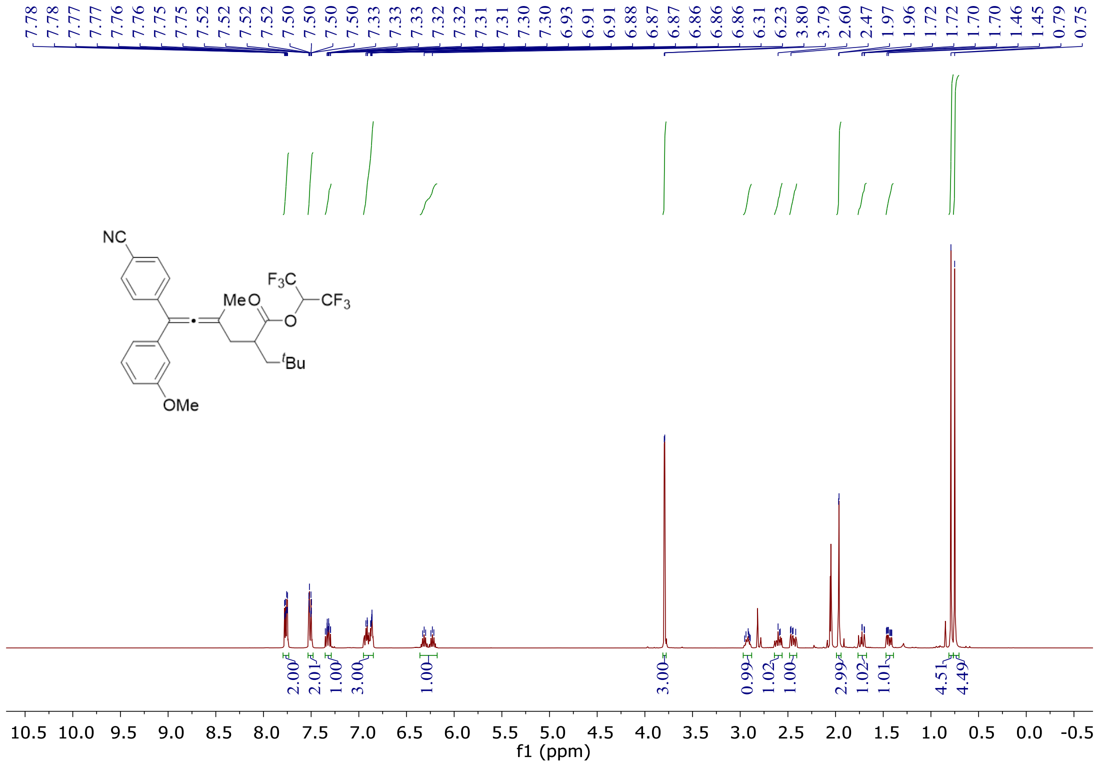

^{1}H NMR (400 MHz, acetone-d_6) of **8**

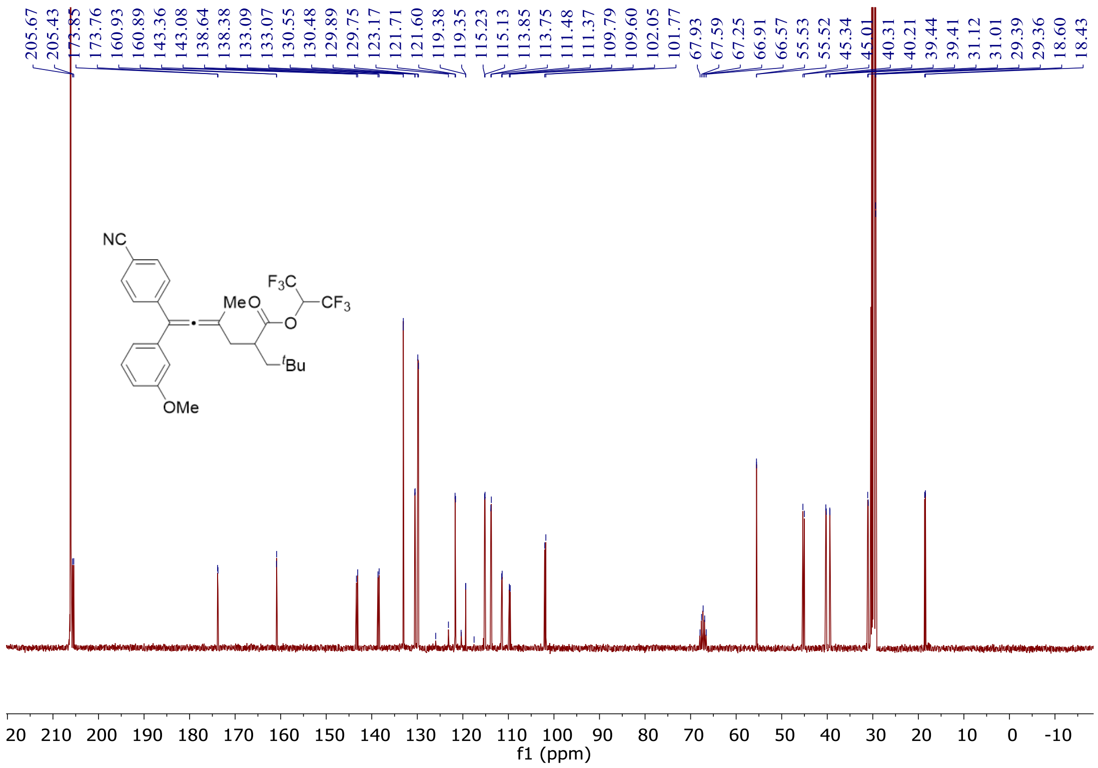

$^{13}C\{^1H\}$ **NMR** (100 MHz, acetone-d_6) of **8**

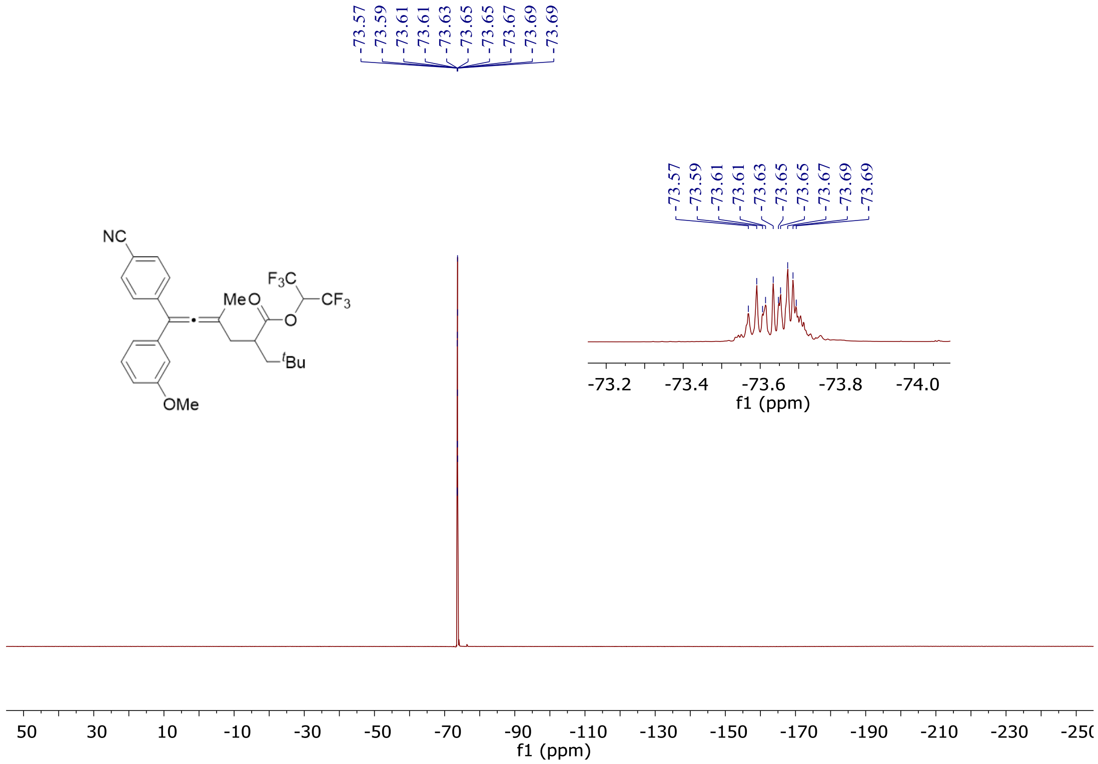

$^{19}F\{^1H\}$ **NMR** (377 MHz, acetone-d_6) of **8**

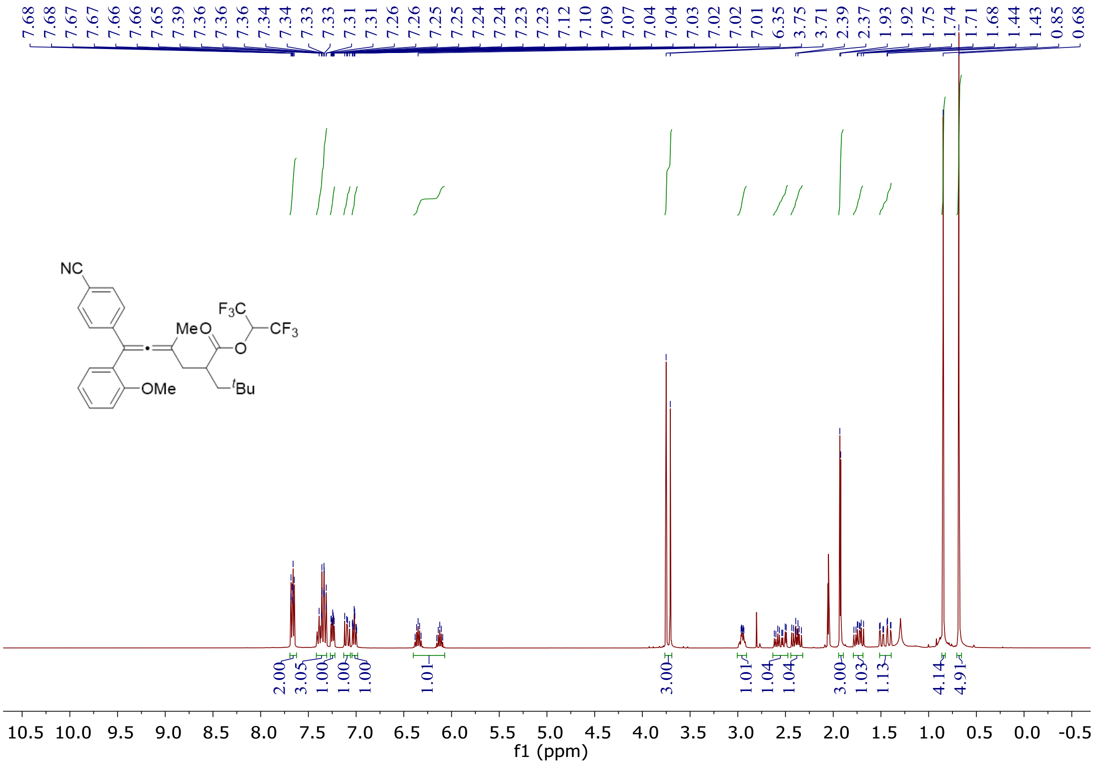

^{1}H NMR (400 MHz, acetone-d_6) of **9**

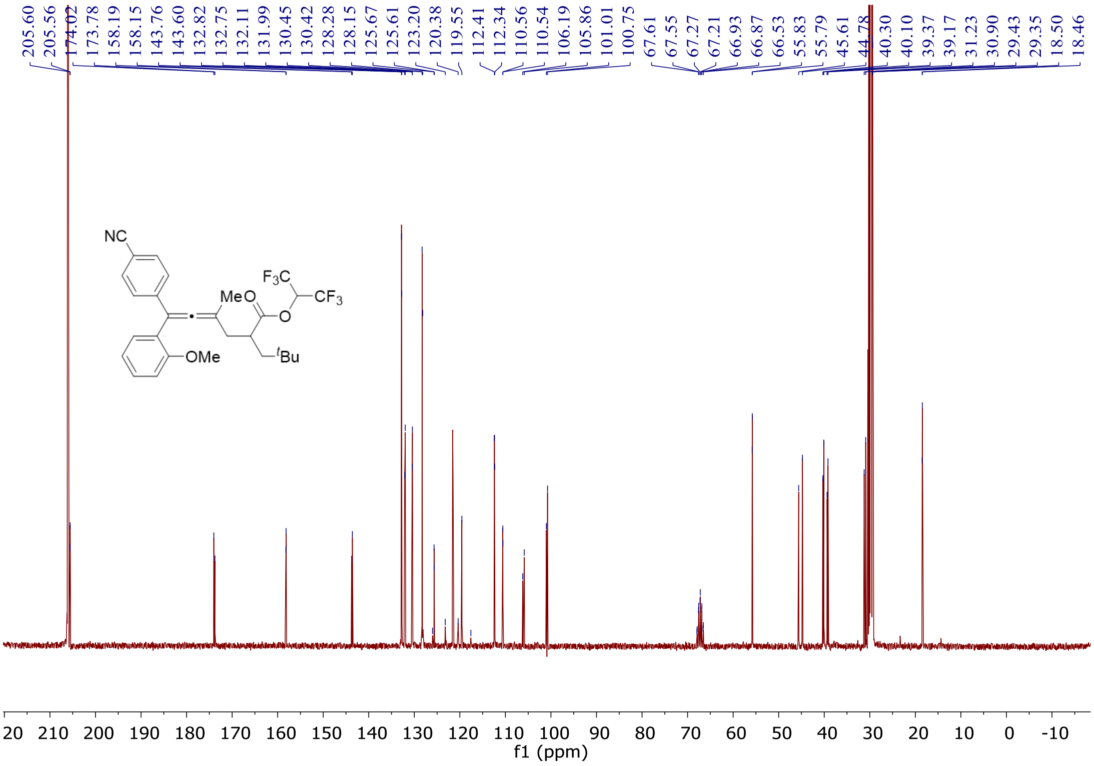

^{13}C{^{1}H} NMR (100 MHz, acetone-d_6) of **9**

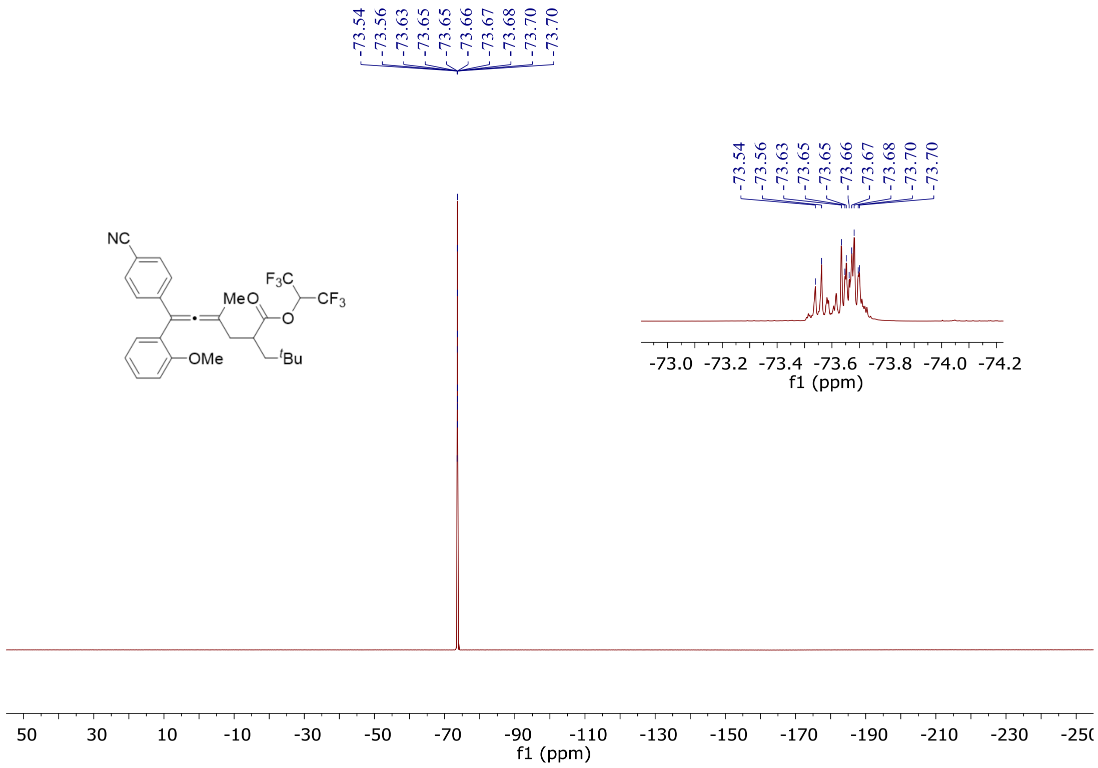

$^{19}F\{^{1}H\}$ NMR (377 MHz, acetone-d_6) of **9**

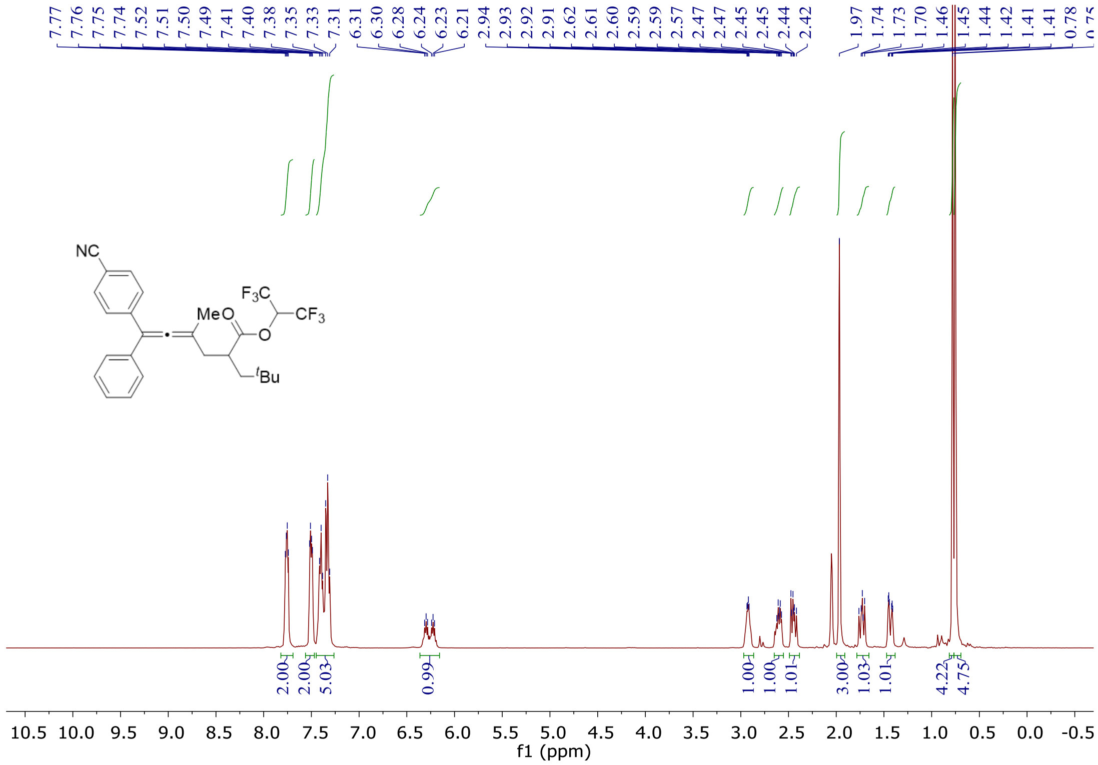

^{1}H NMR (400 MHz, acetone-d_6) of **10**

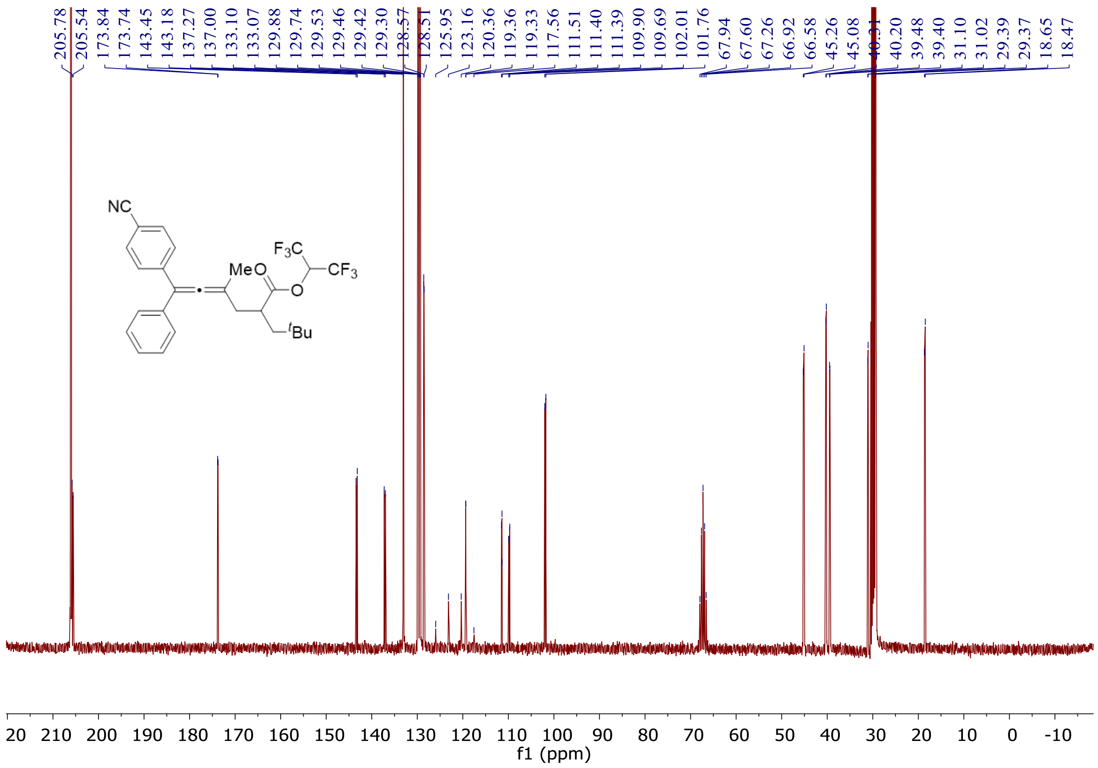

$^{13}C\{^1H\}$ NMR (100 MHz, acetone-d_6) of **10**

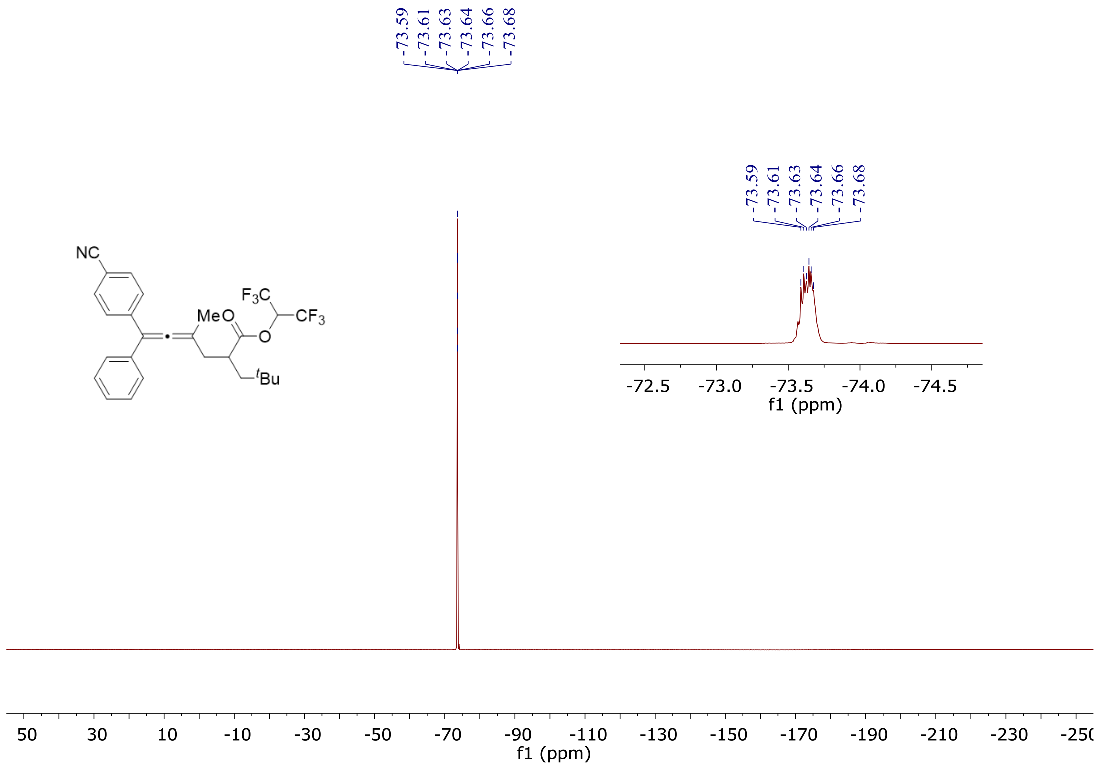

$^{19}F\{^1H\}$ NMR (377 MHz, acetone-d_6) of **10**

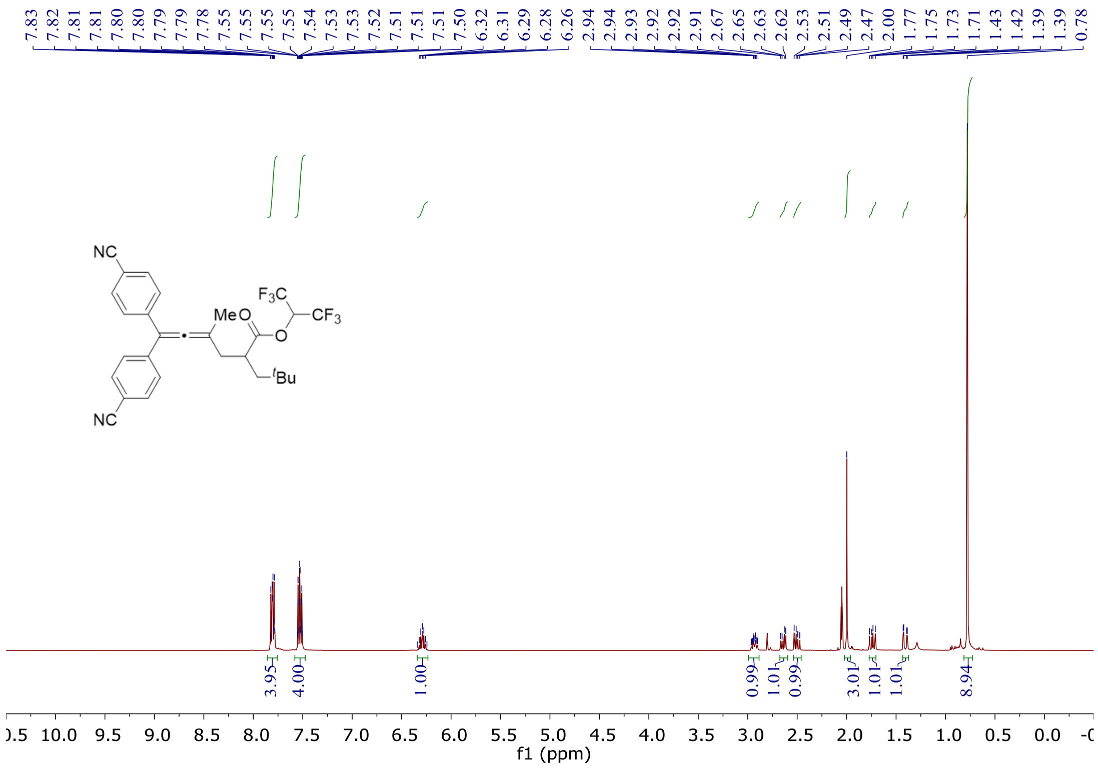

^{1}H **NMR** (400 MHz, acetone-d_6) of **11**

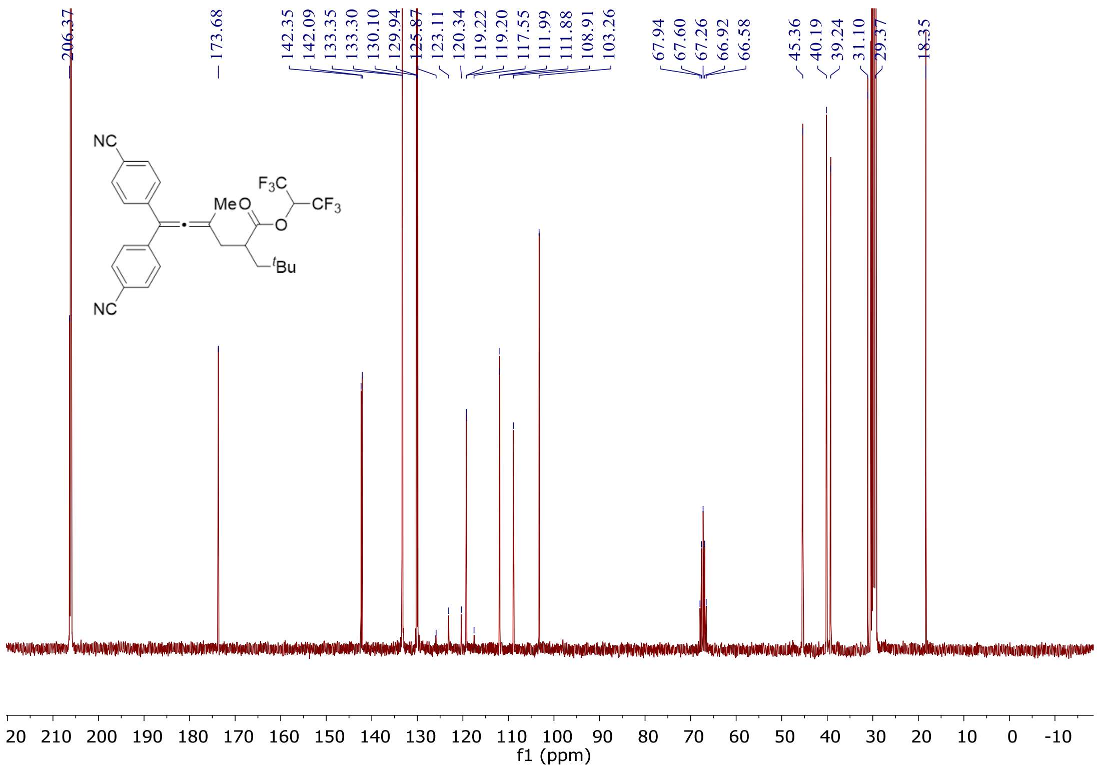

$^{13}C\{^{1}H\}$ **NMR** (100 MHz, acetone-d_6) of **11**

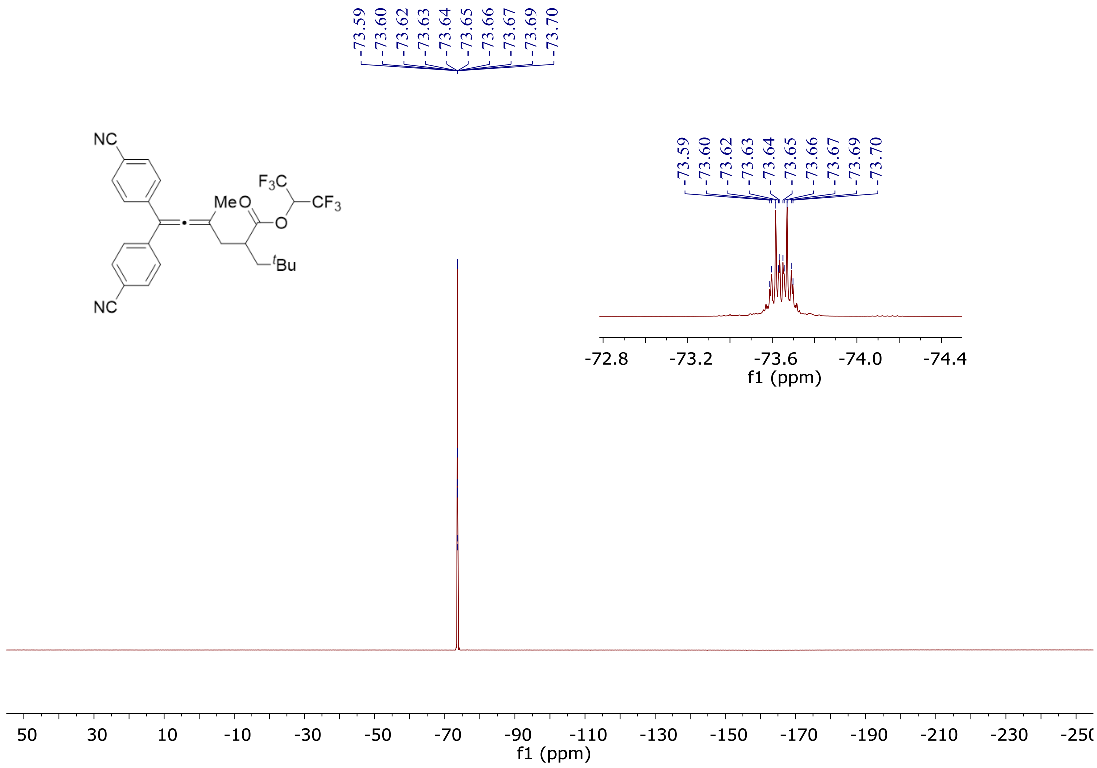

^{19}F{^{1}H} NMR (377 MHz, acetone-d_6) of **11**

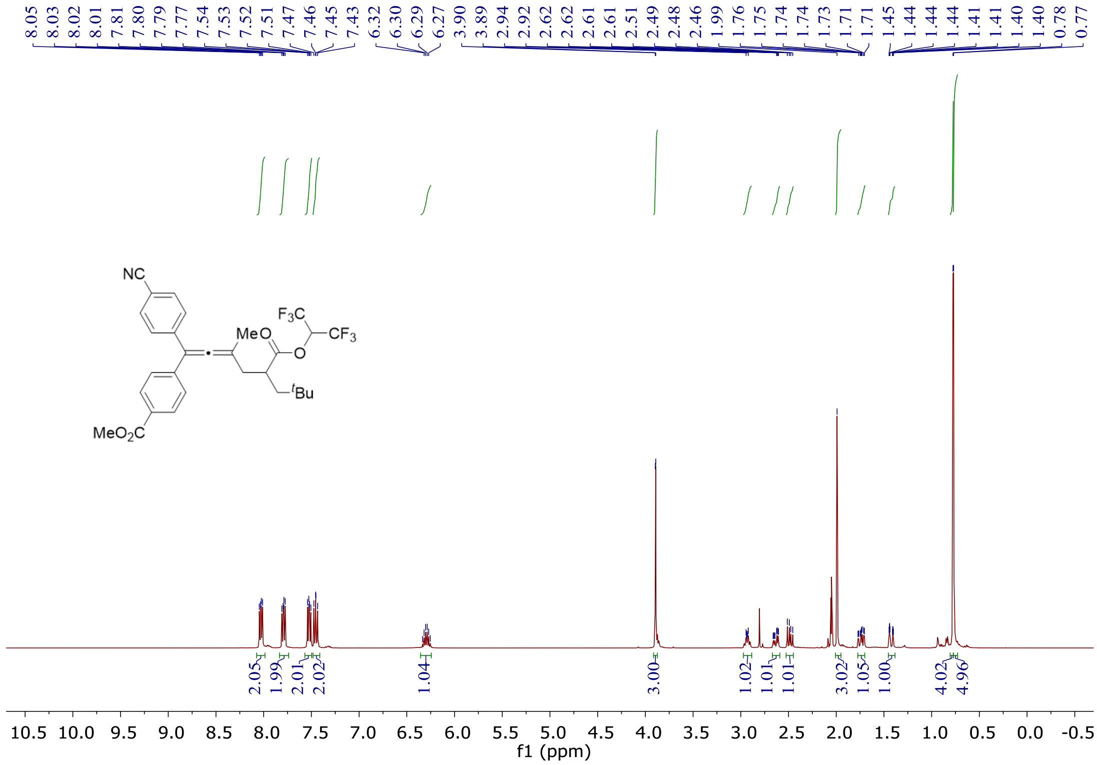

^{1}H NMR (400 MHz, acetone-d_6) of **12**

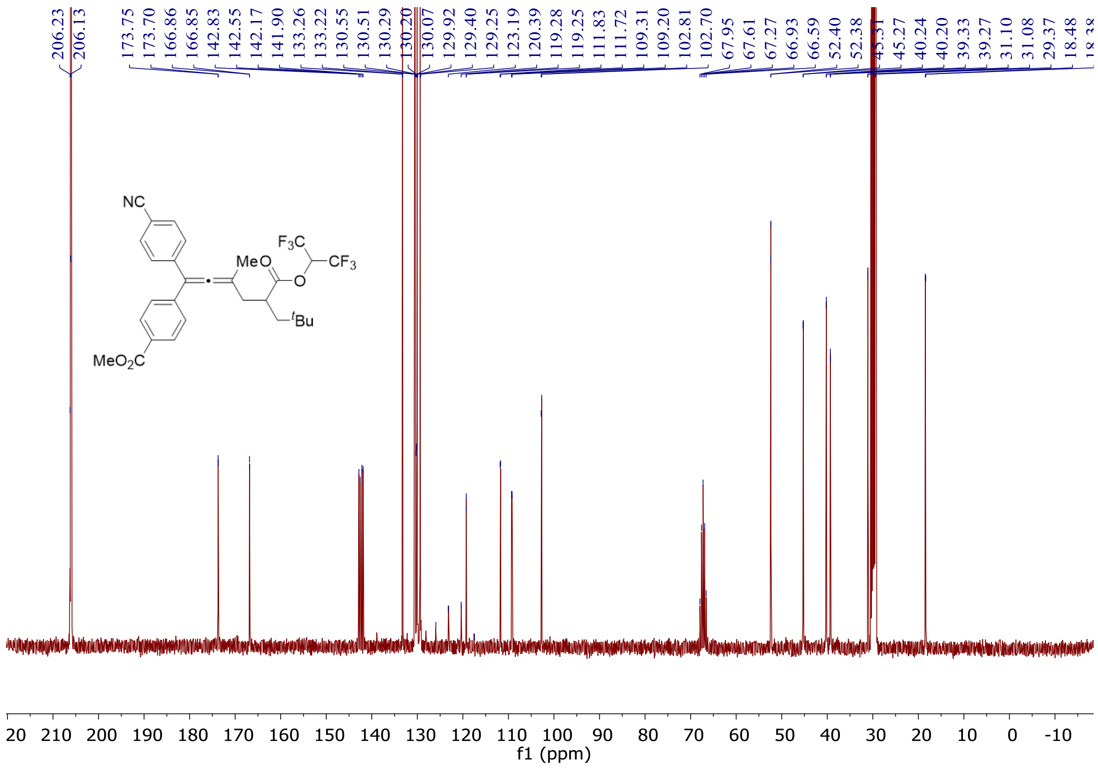

^{13}C{^{1}H} **NMR** (100 MHz, acetone-d_6) of **12**

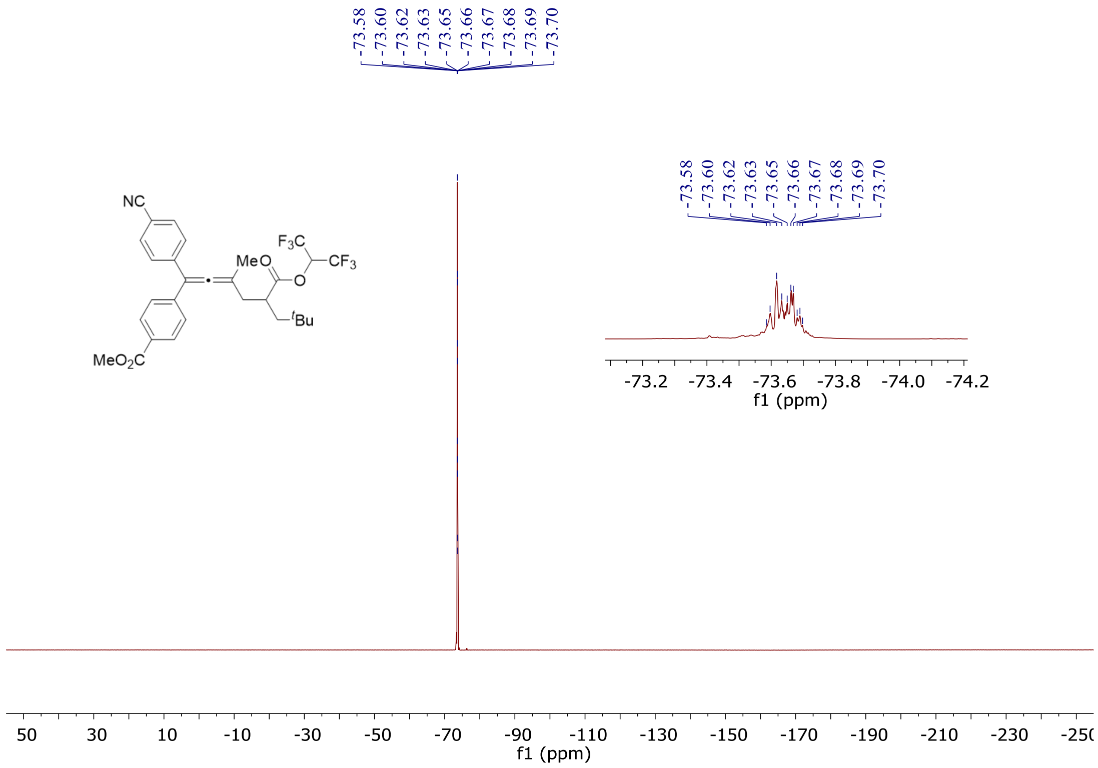

^{19}F{^{1}H} **NMR** (377 MHz, acetone-d_6) of **12**

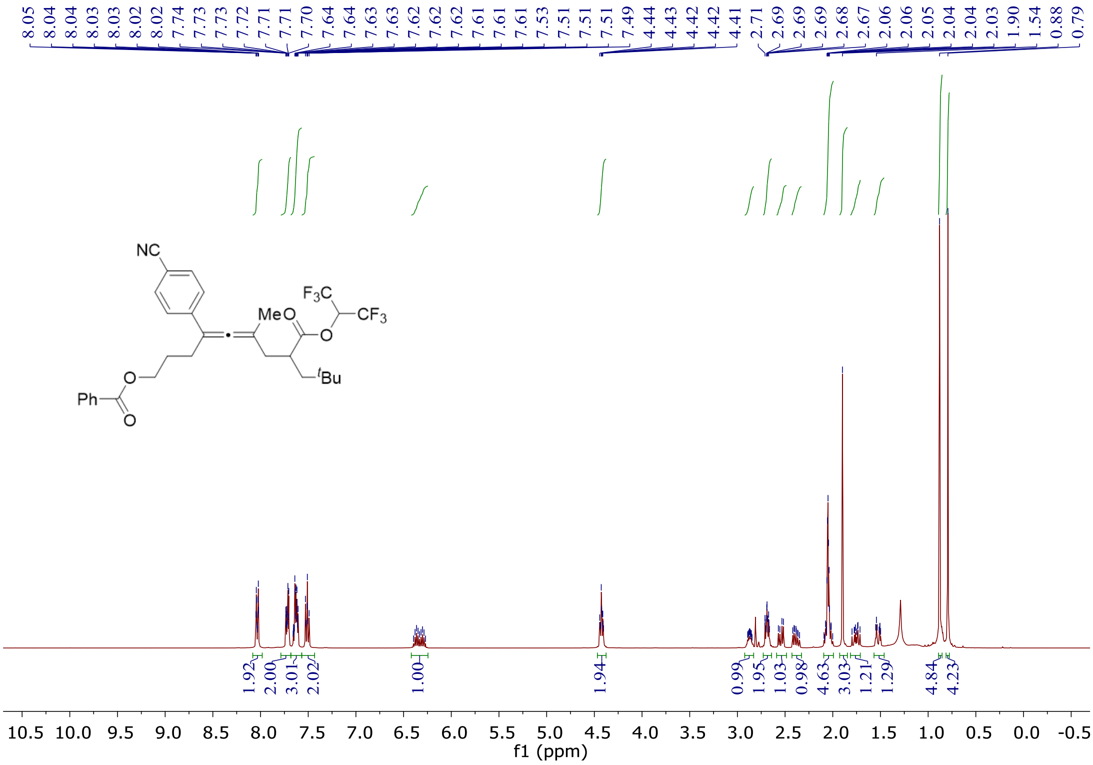

^{1}H NMR (400 MHz, acetone-d_6) of **13**

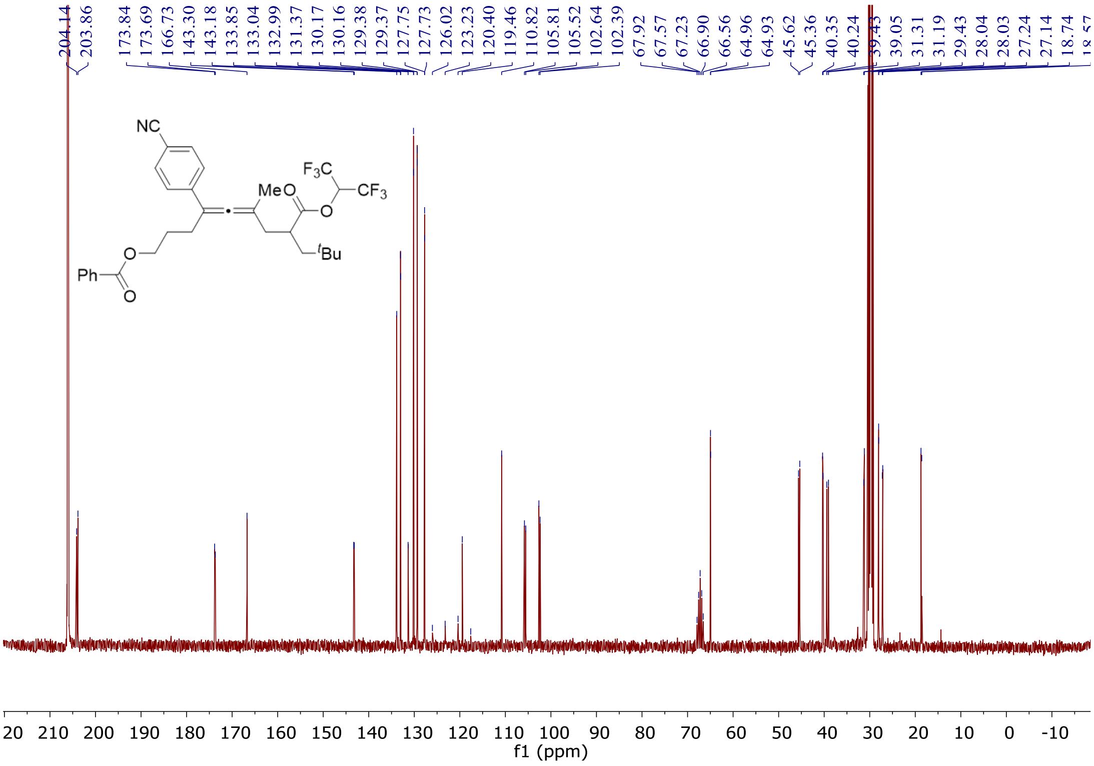

^{13}C{^{1}H} NMR (100 MHz, acetone-d_6) of **13**

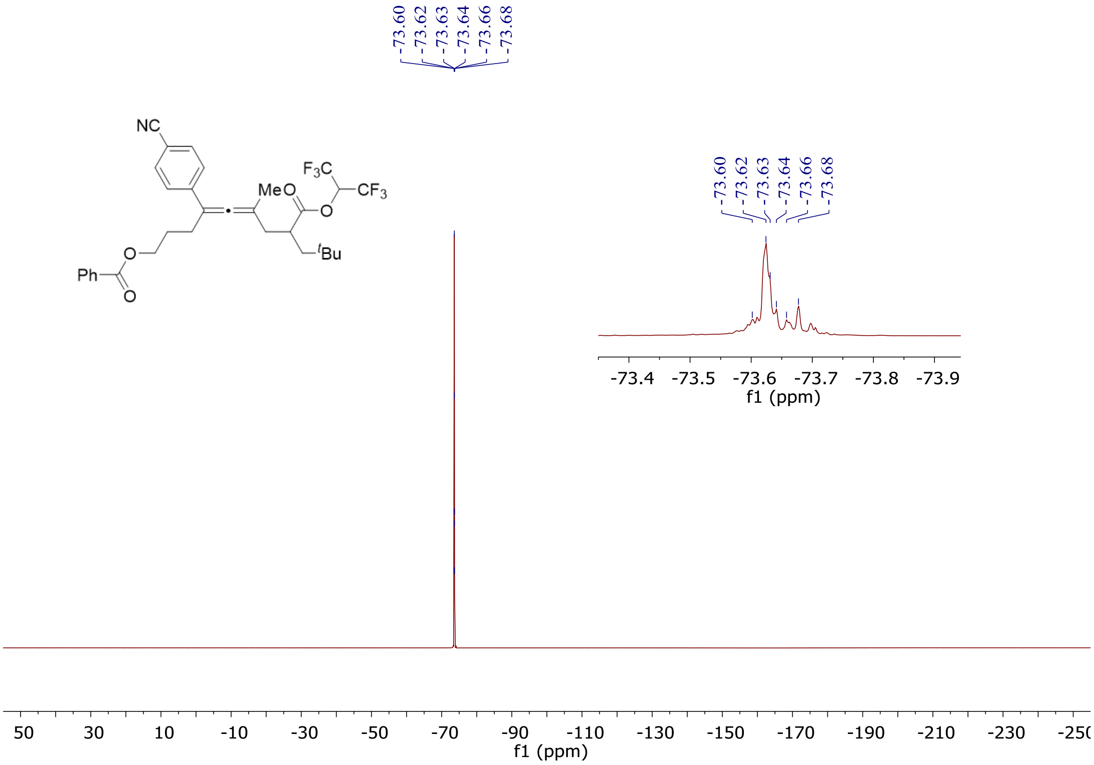

^{19}F{^{1}H} **NMR** (377 MHz, acetone-d_6) of **13**

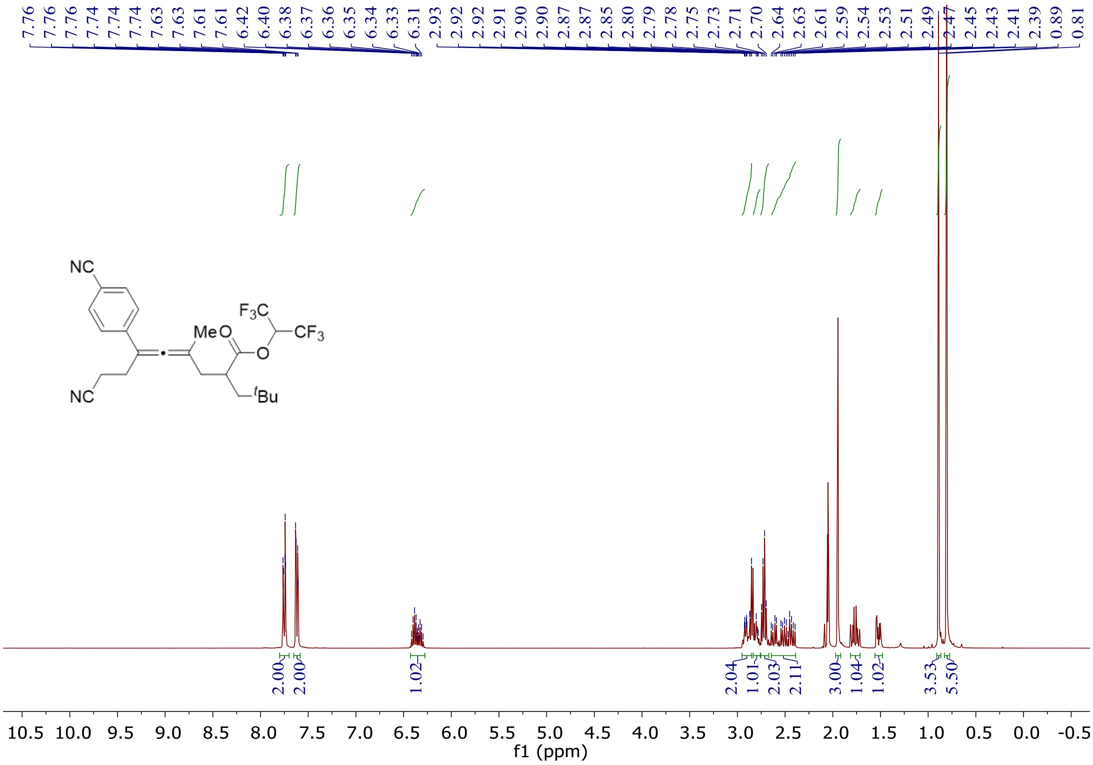

^{1}**H NMR** (400 MHz, acetone-d_6) of **14**

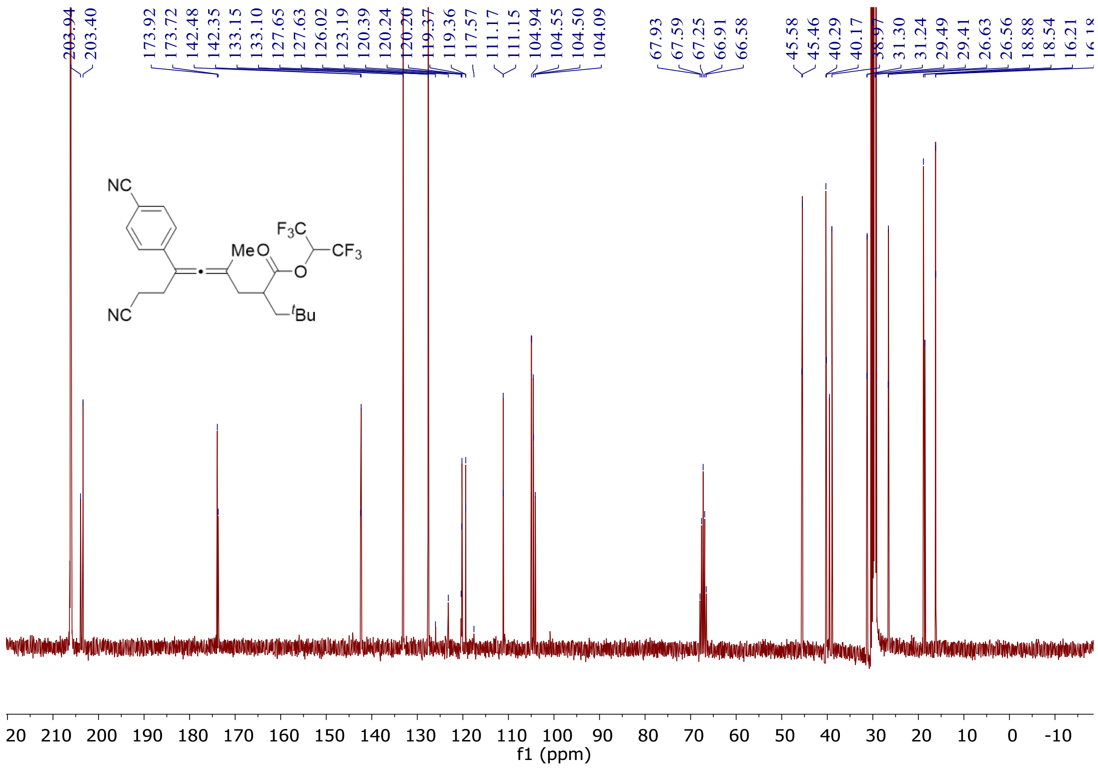

$^{13}C\{^{1}H\}$ NMR (100 MHz, acetone-d_6) of **14**

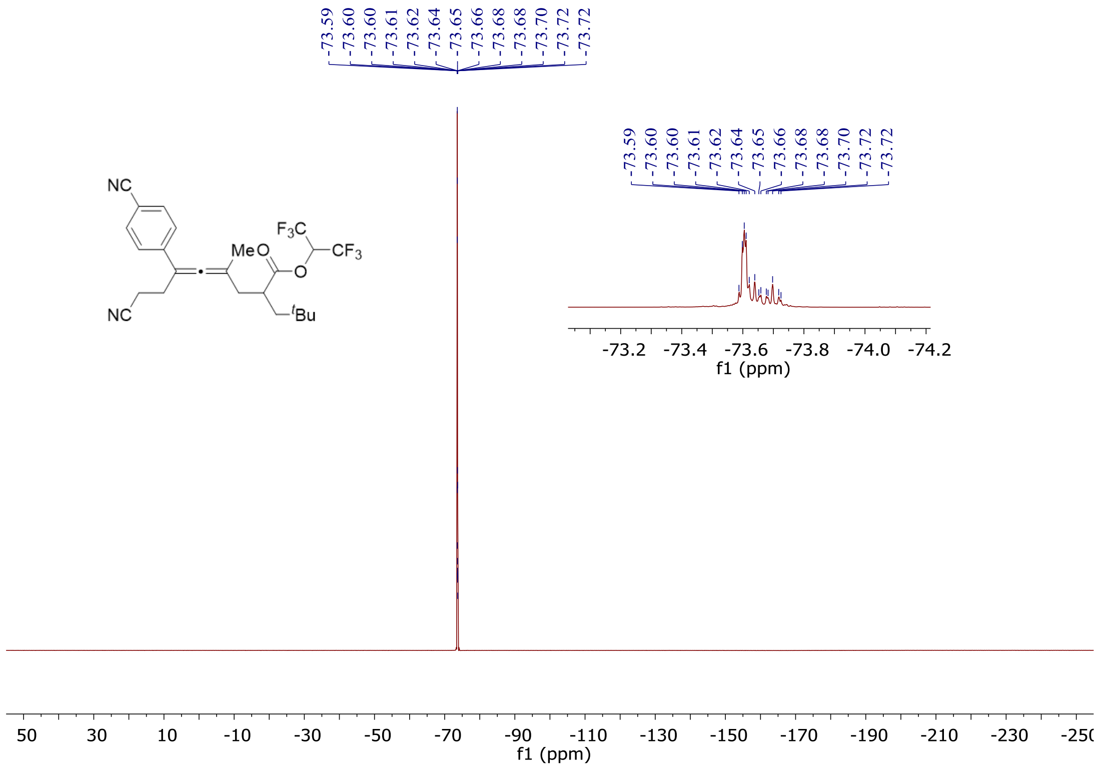

$^{19}F\{^{1}H\}$ NMR (377 MHz, acetone-d_6) of **14**

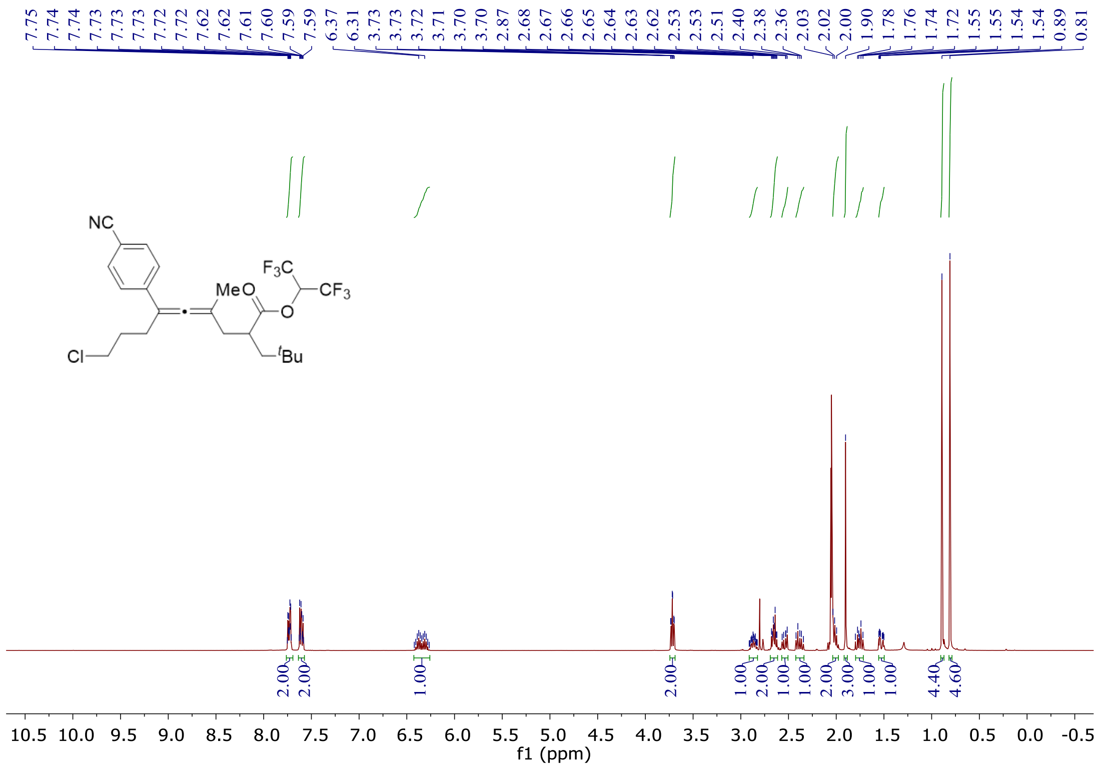

^{1}H NMR (400 MHz, acetone-d_6) of **15**

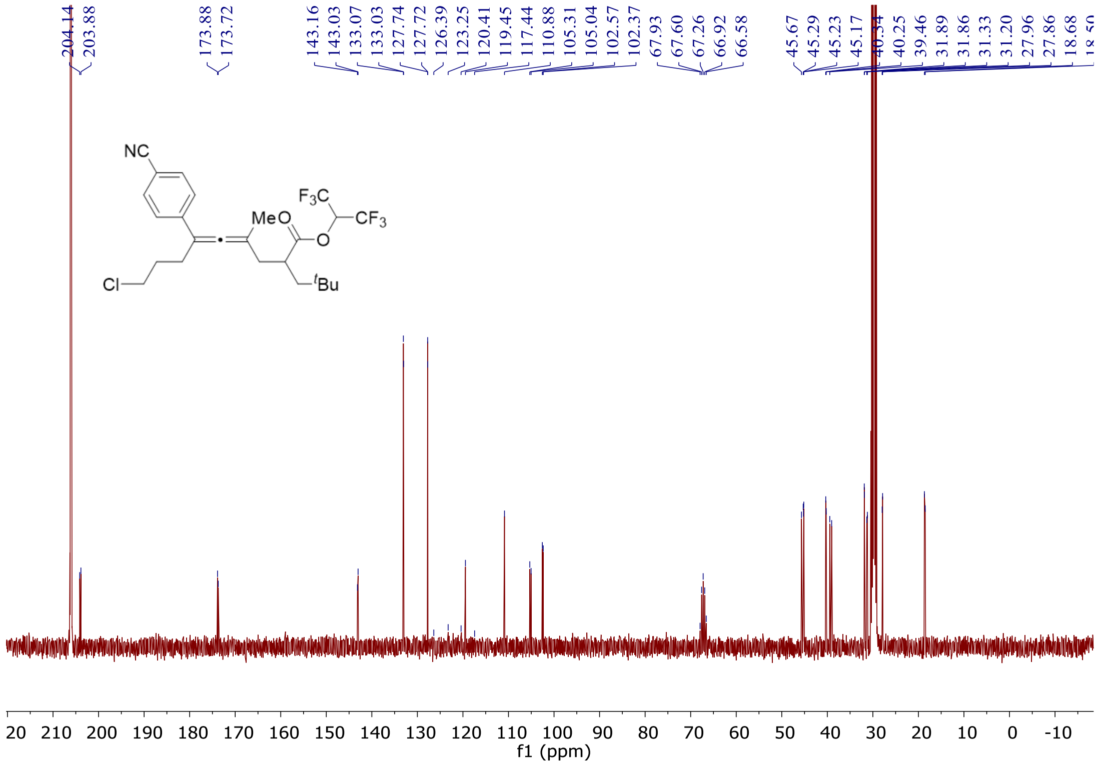

^{13}C{^{1}H} NMR (100 MHz, acetone-d_6) of **15**

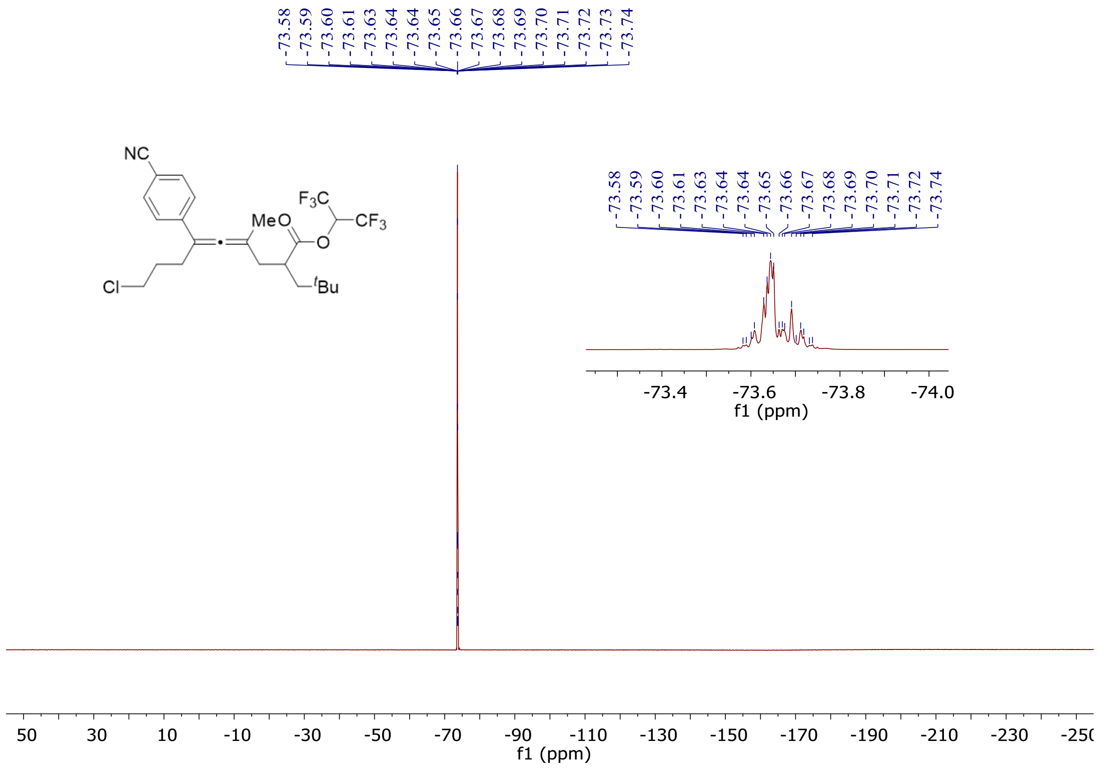

^{19}F{^{1}H} NMR (377 MHz, acetone-d_6) of **15**

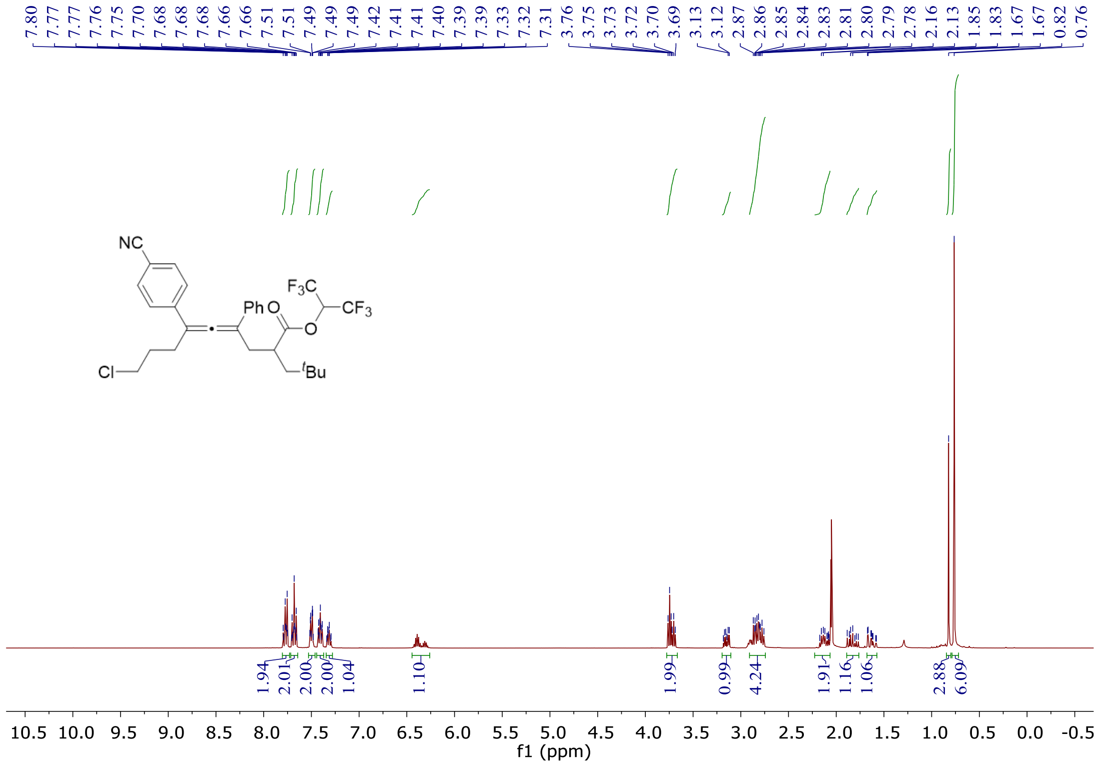

^{1}H NMR (400 MHz, acetone-d_6) of **16**

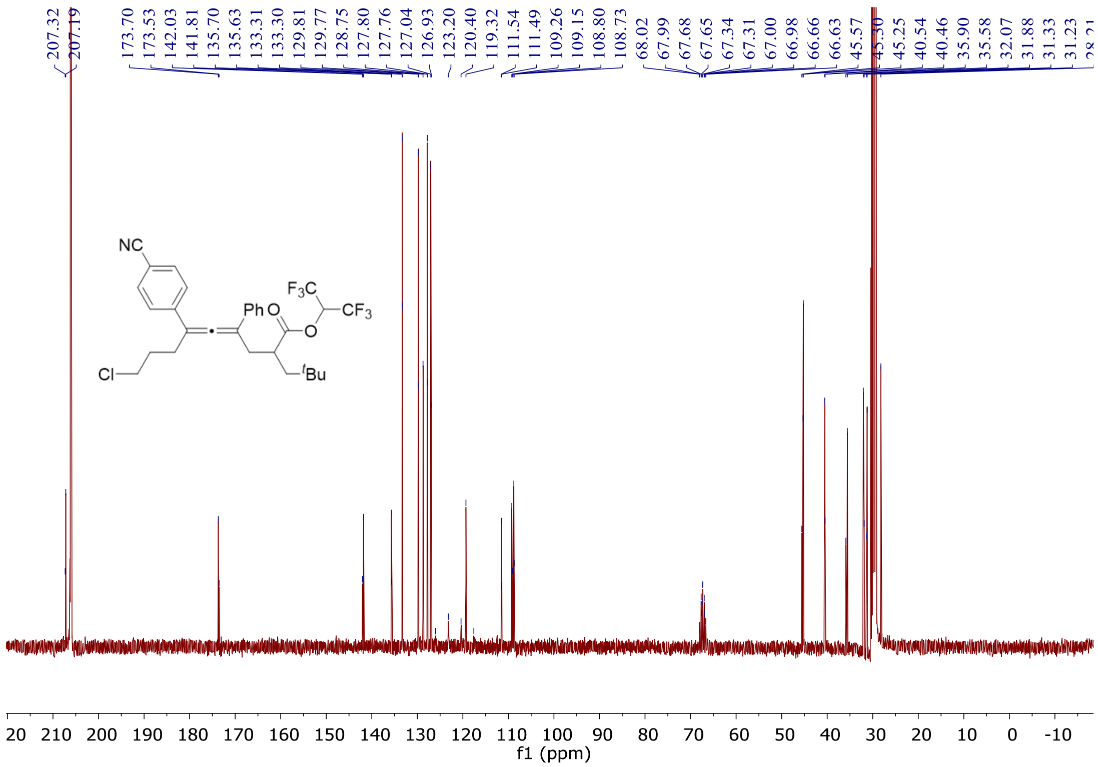

^{13}C{^{1}H} NMR (100 MHz, acetone-d_6) of **16**

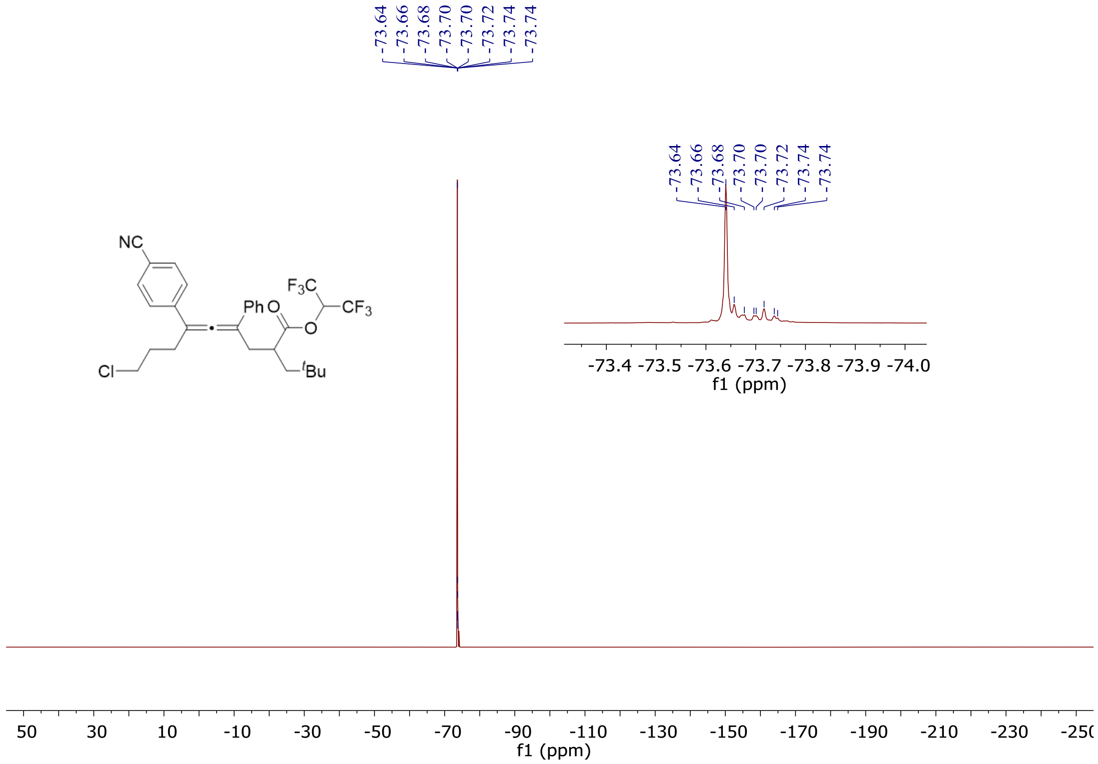

^{19}F{^{1}H} NMR (377 MHz, acetone-d_6) of **16**

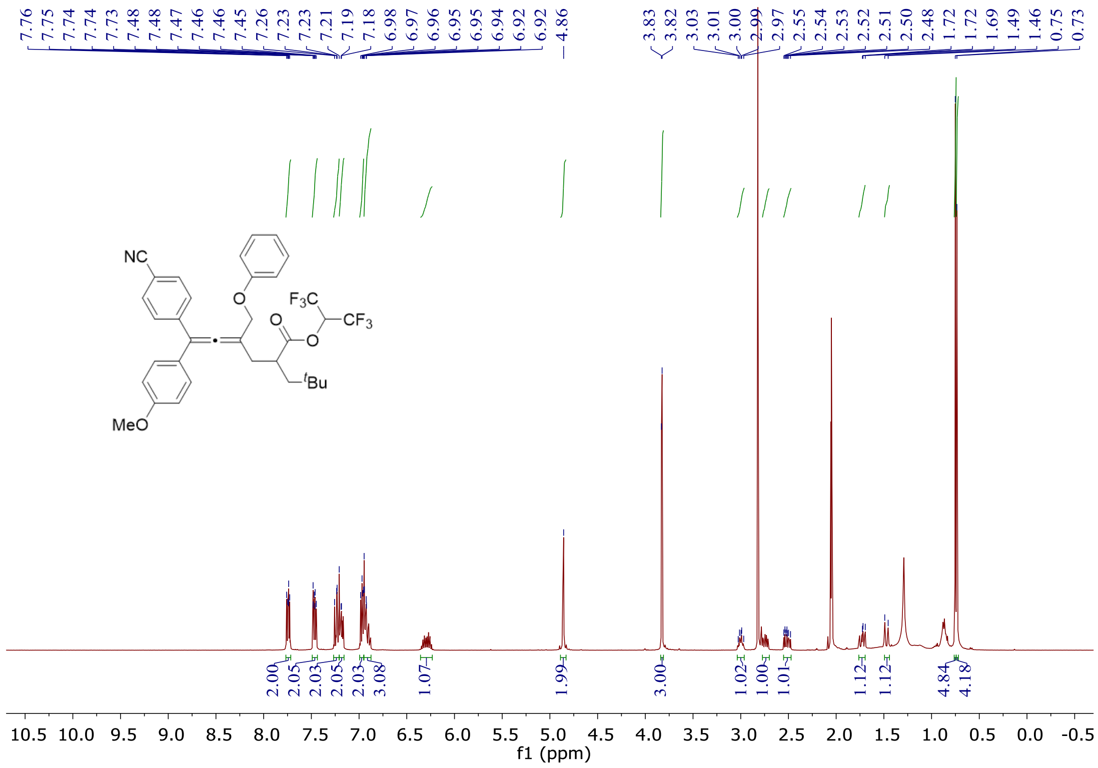

^{1}H NMR (400 MHz, acetone-d_6) of **17**

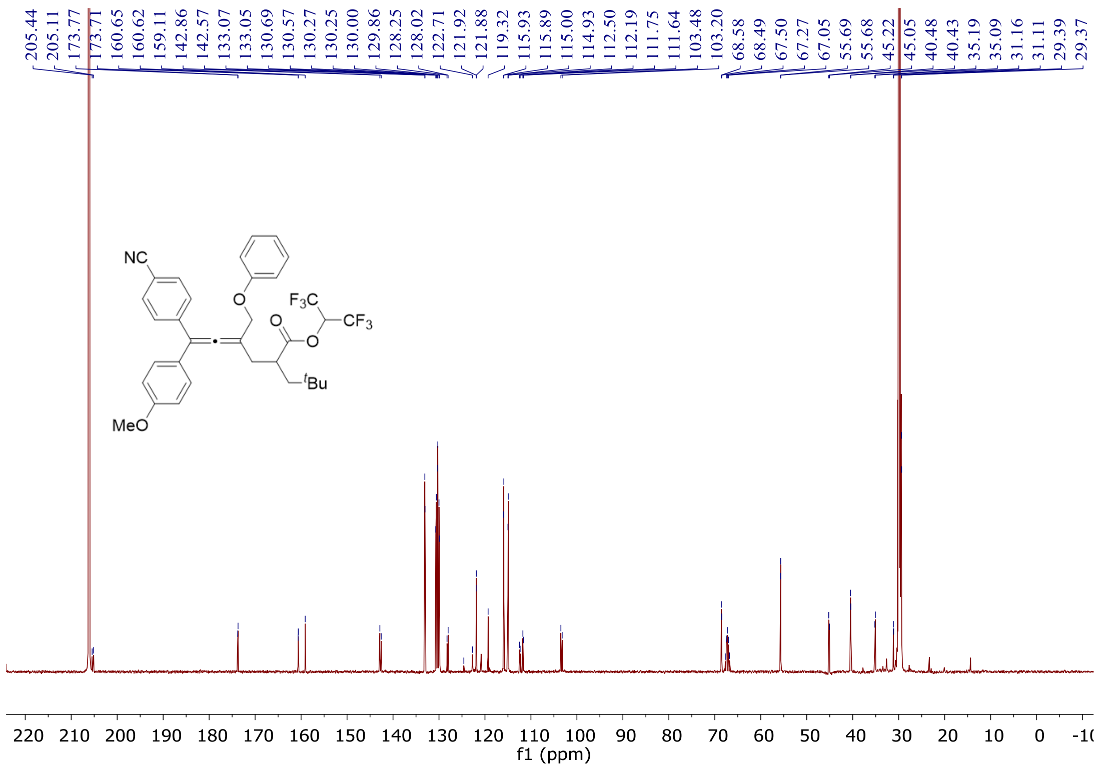

^{13}C{^{1}H} NMR (100 MHz, acetone-d_6) of **17**

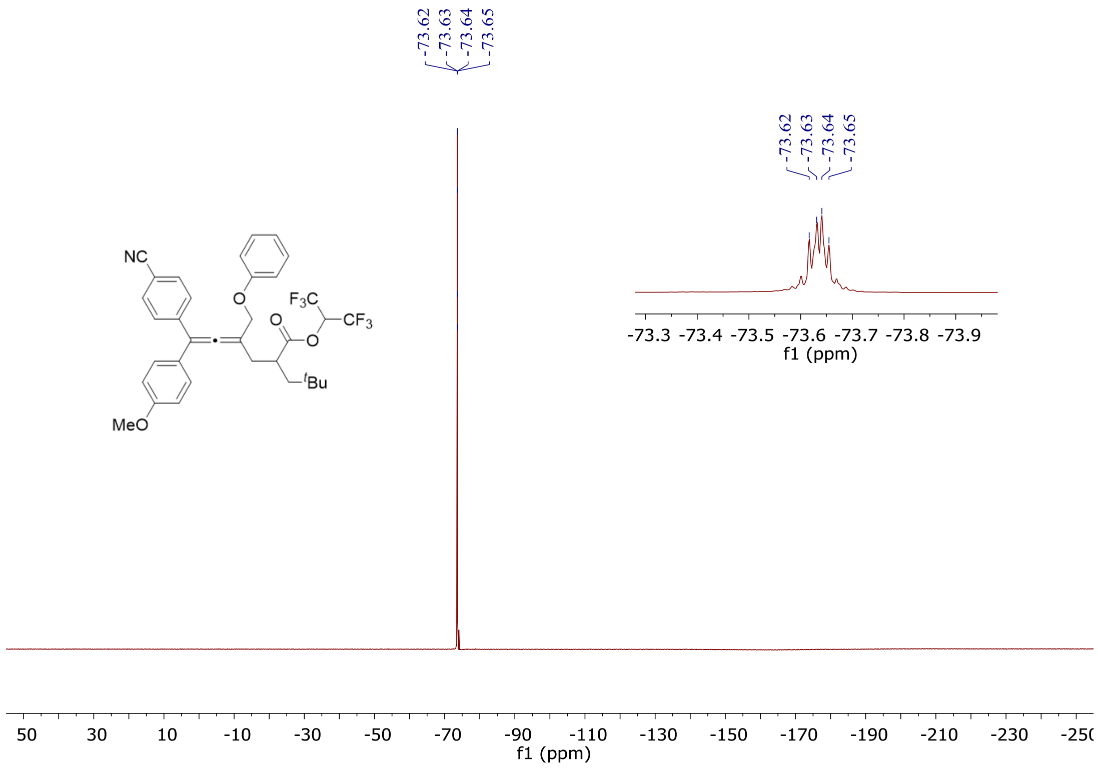

^{19}F{^{1}H} NMR (377 MHz, acetone-d_6) of **17**

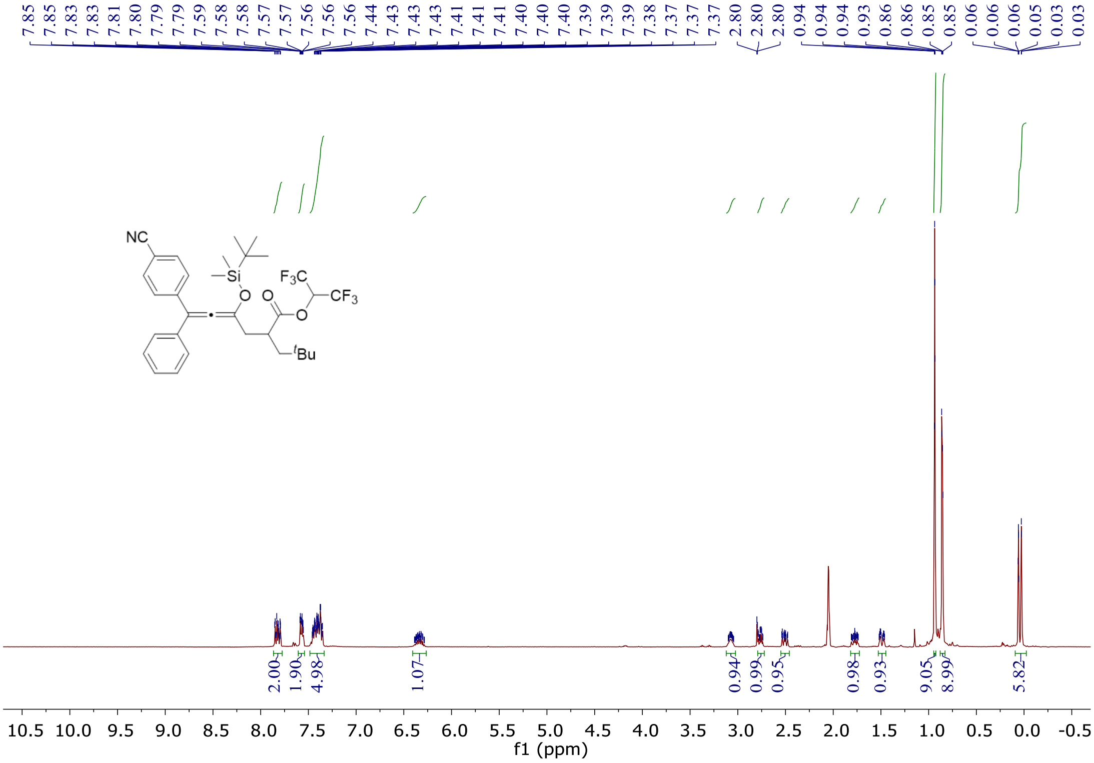

^{1}H NMR (400 MHz, acetone-d_6) of **18**

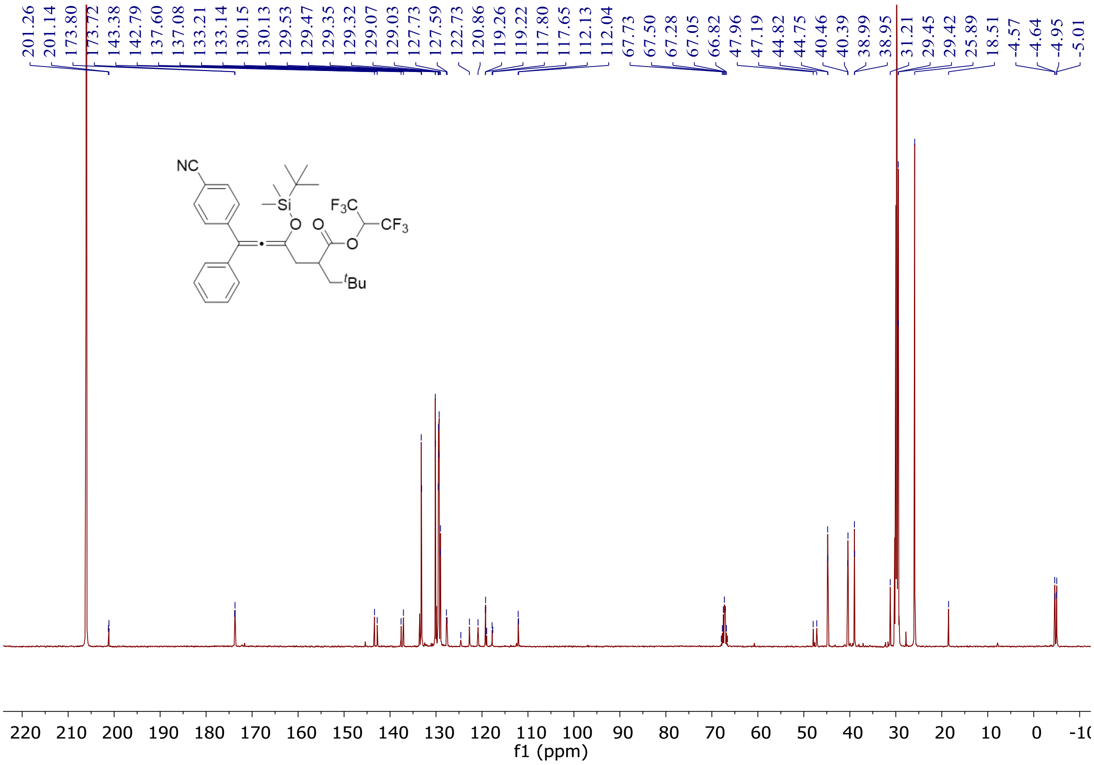

^{13}C{^{1}H} NMR (100 MHz, acetone-d_6) of **18**

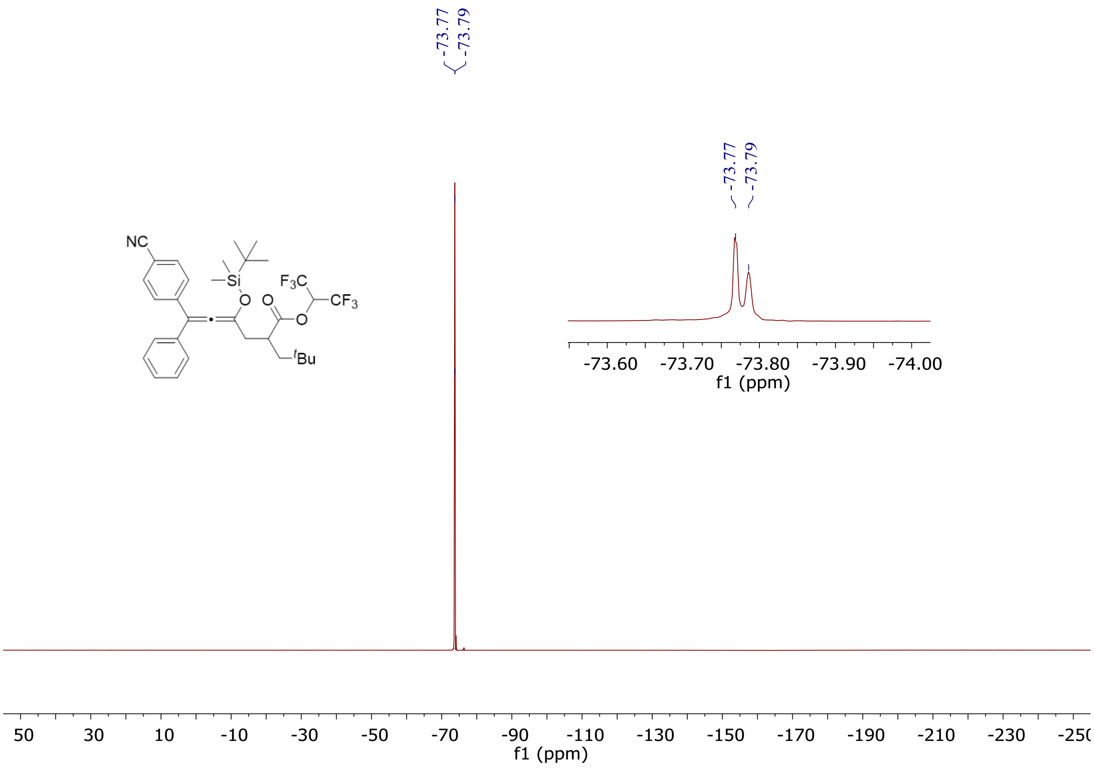

^{19}**F{^{1}H} NMR** (377 MHz, acetone-d_6) of **18**

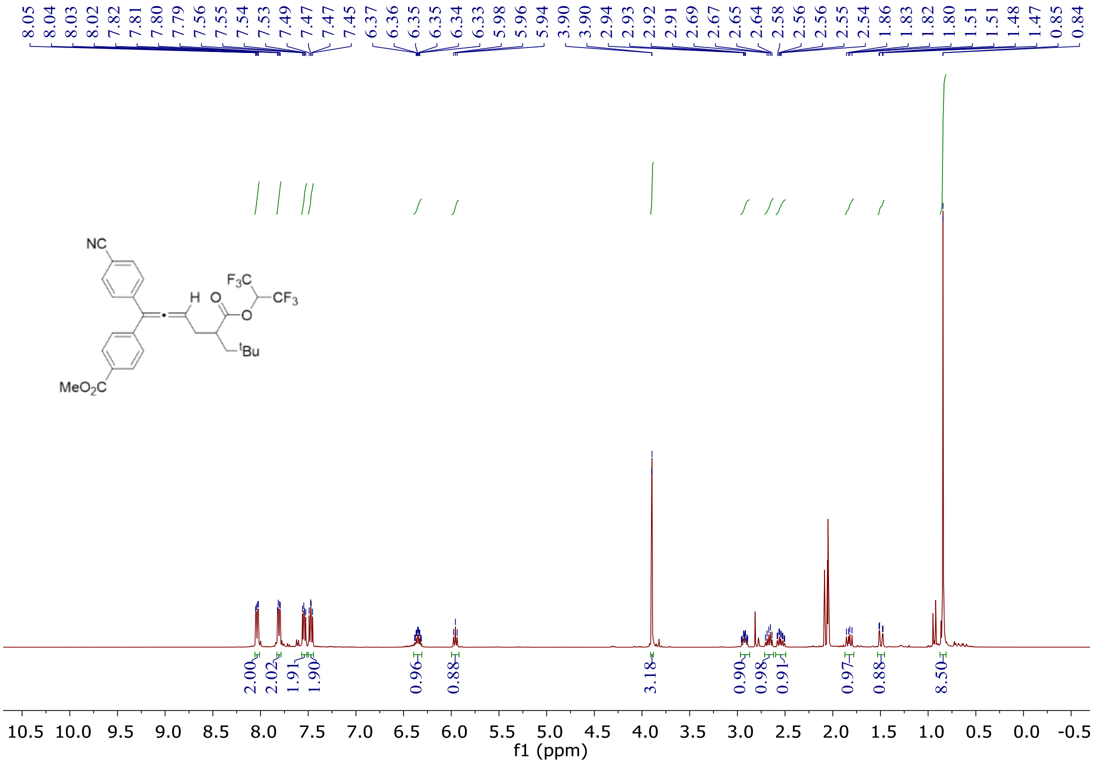

^{1}**H NMR** (400 MHz, acetone-d_6) of **19**

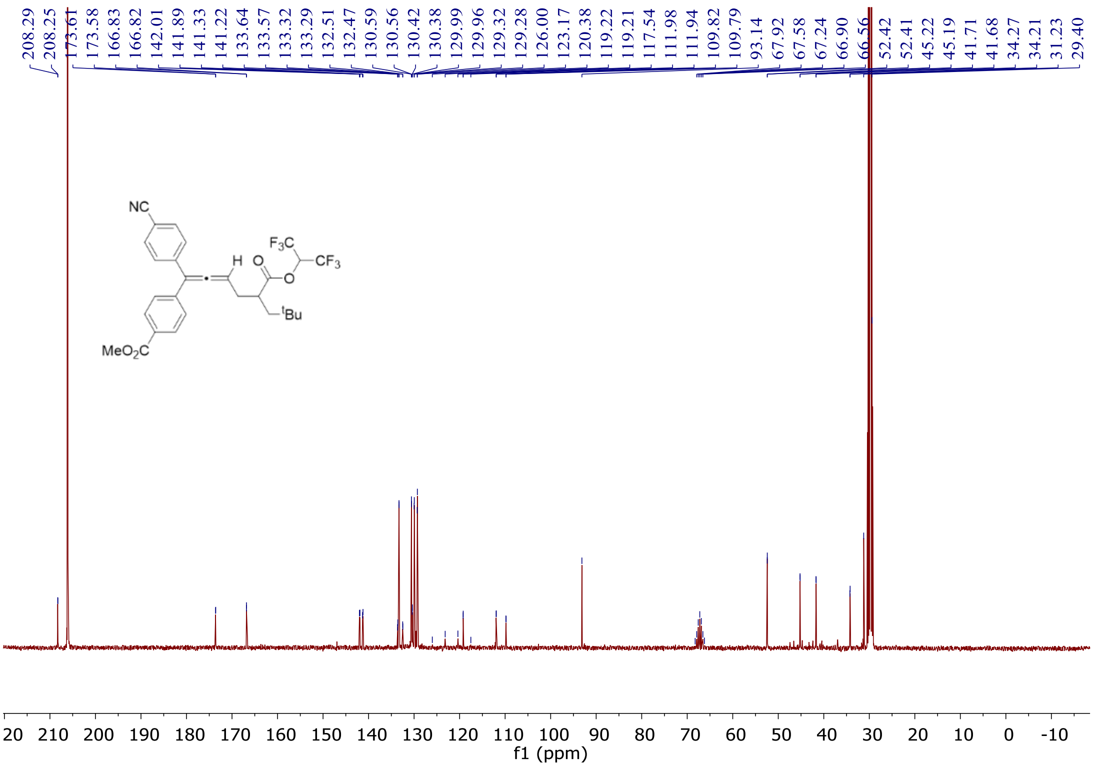

$^{13}C\{^{1}H\}$ NMR (100 MHz, acetone-d_6) of **19**

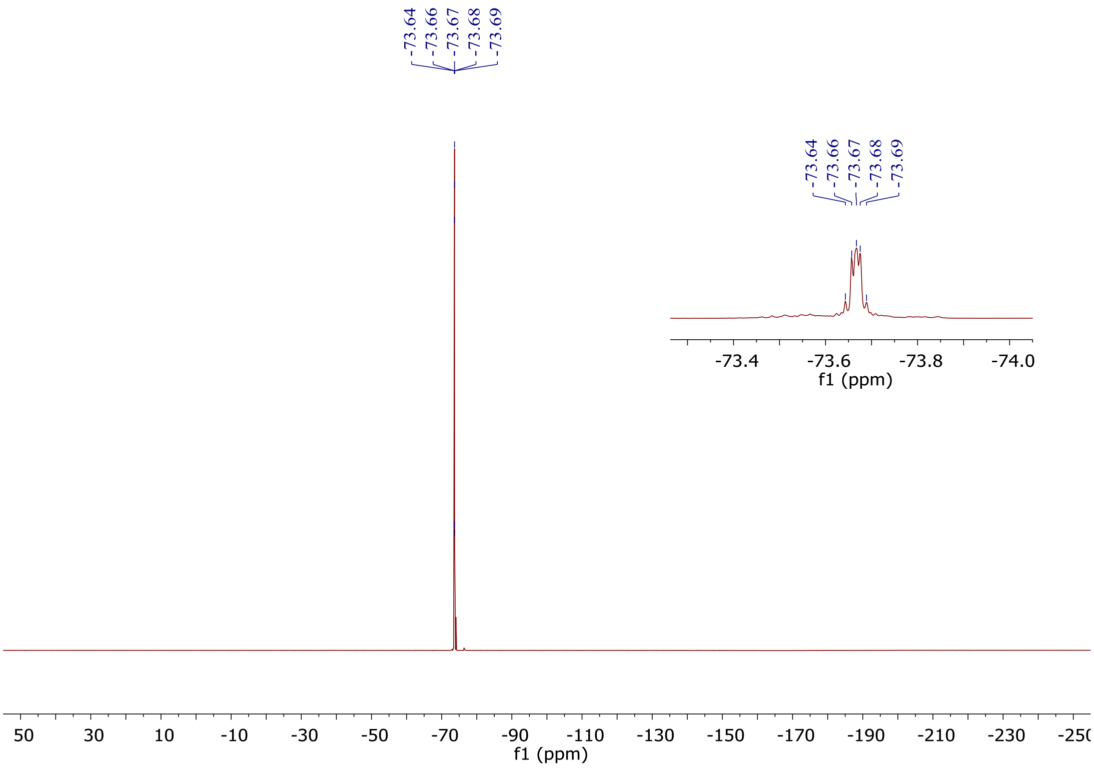

$^{19}F\{^{1}H\}$ NMR (377 MHz, acetone-d_6) of **19**

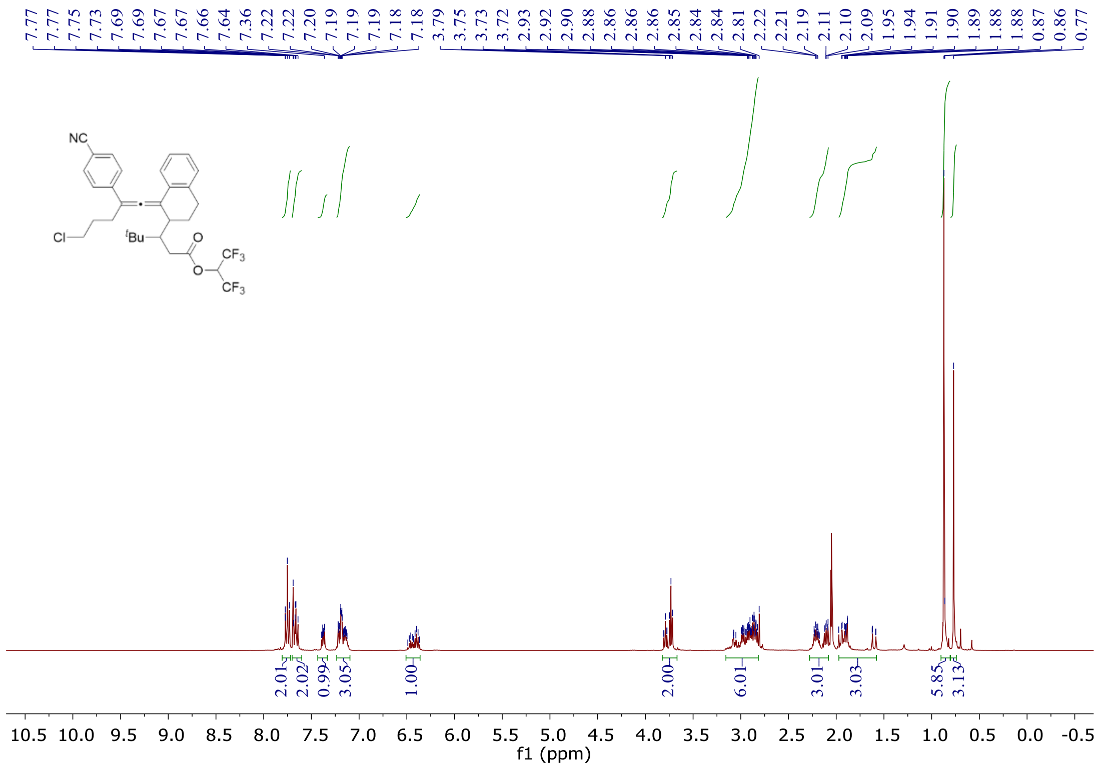

^{1}**H NMR** (400 MHz, acetone-d_6) of **20**

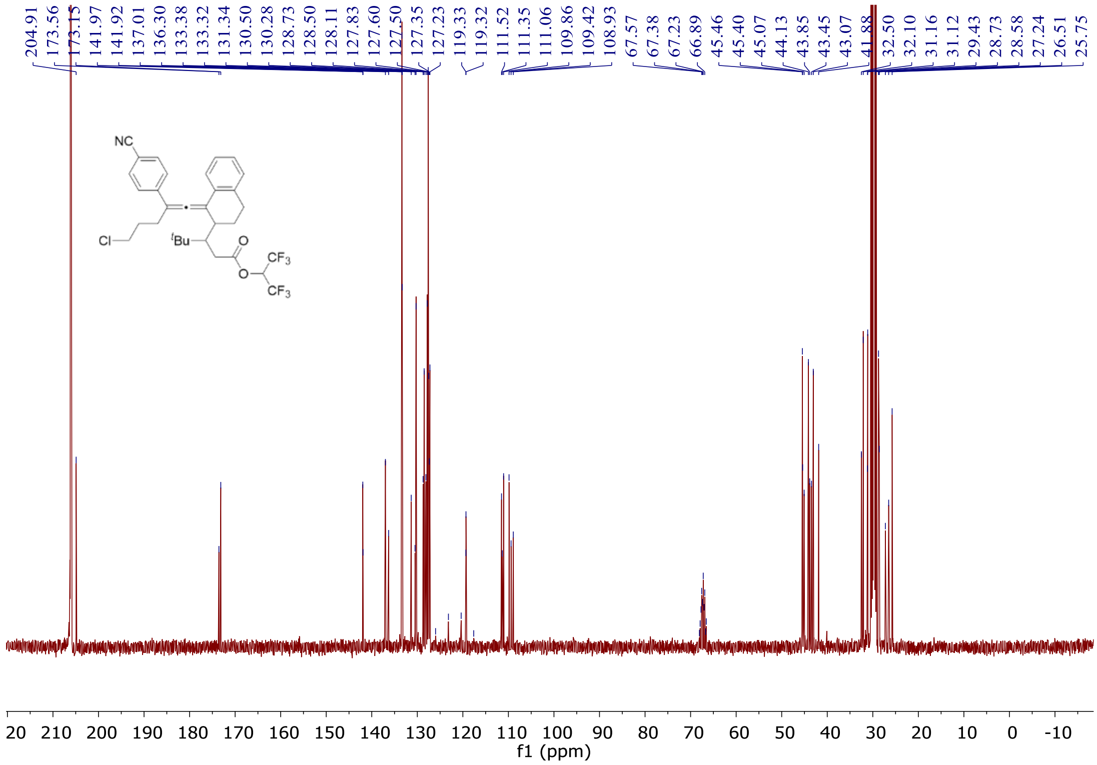

^{13}**C{^{1}H} NMR** (100 MHz, acetone-d_6) of **20**

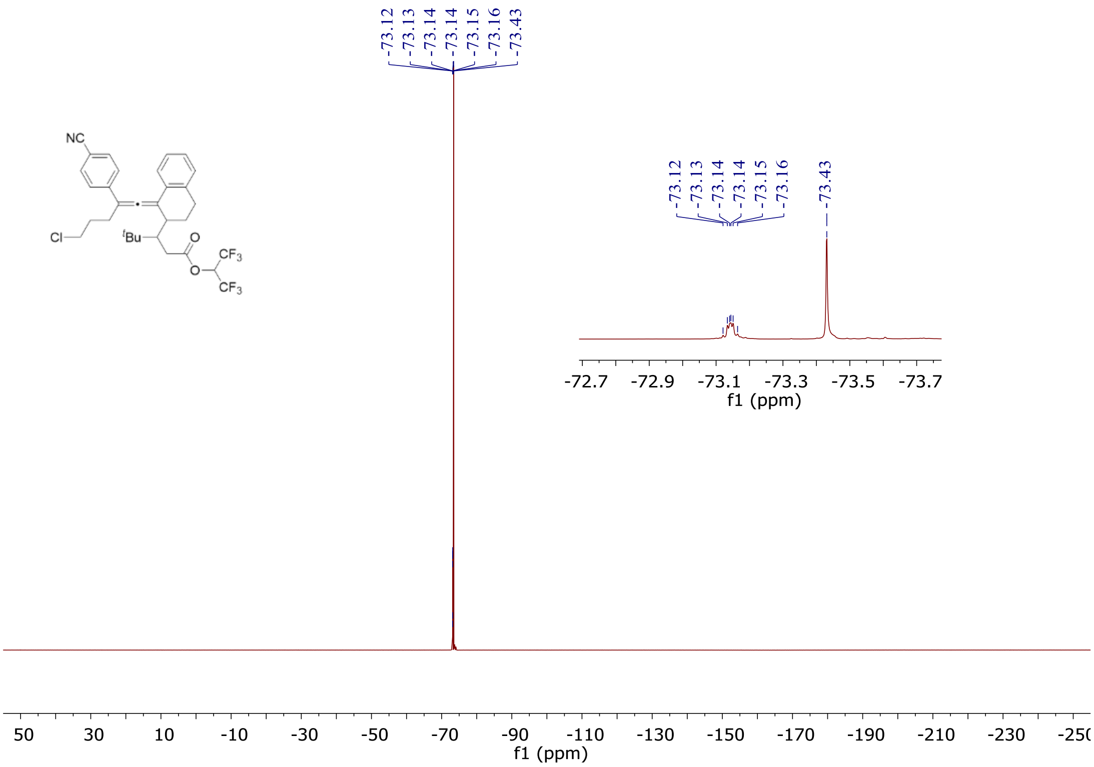

$^{19}F\{^{1}H\}$ **NMR** (377 MHz, acetone-d_6) of **20**

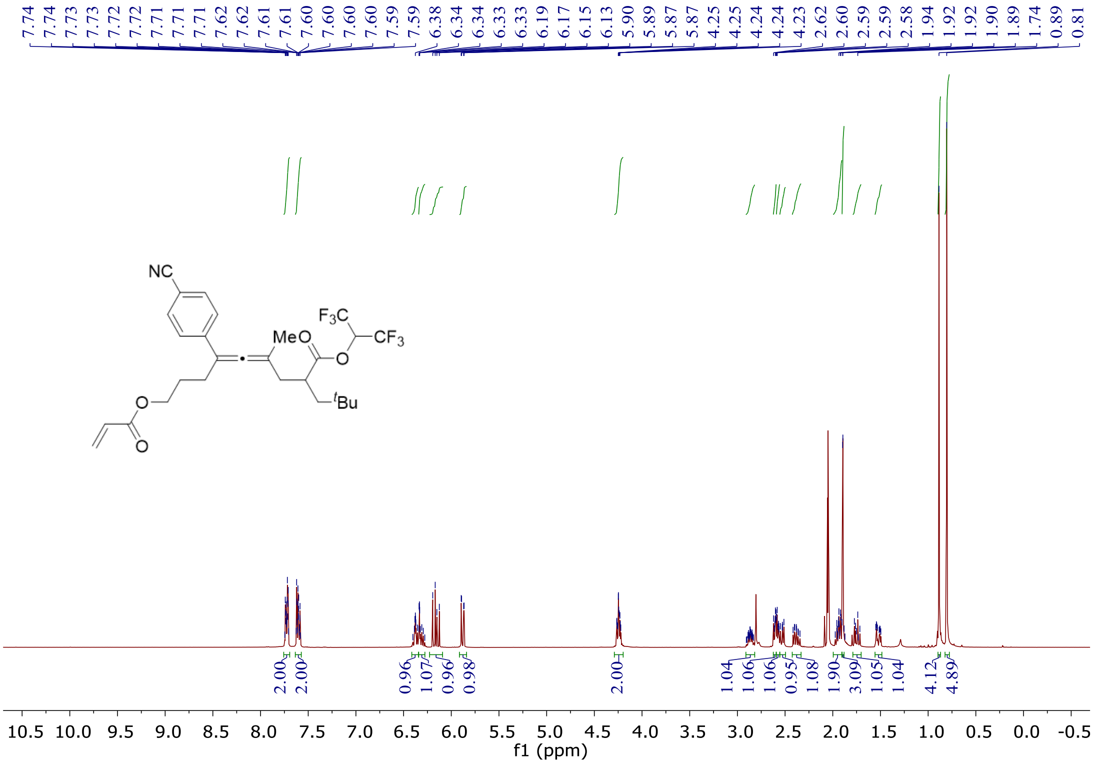

^{1}H **NMR** (400 MHz, acetone-d_6) of **21**

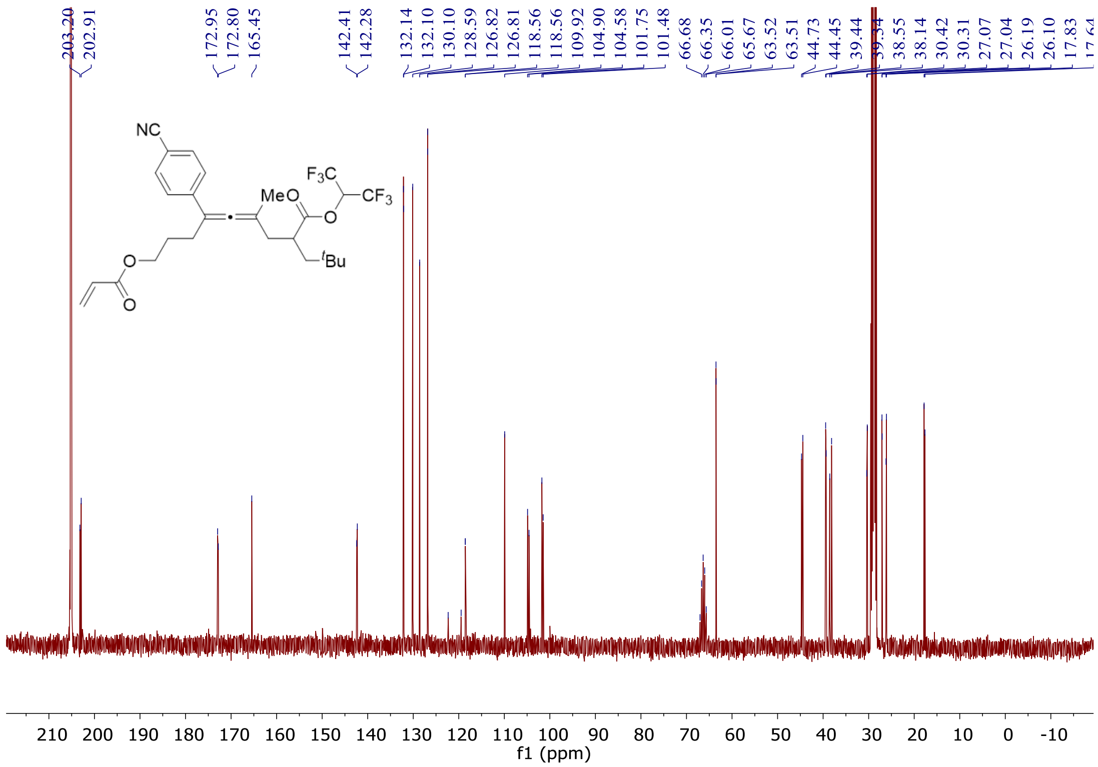

^{13}C{^{1}H} NMR (100 MHz, acetone-*d*$_6$) of **21**

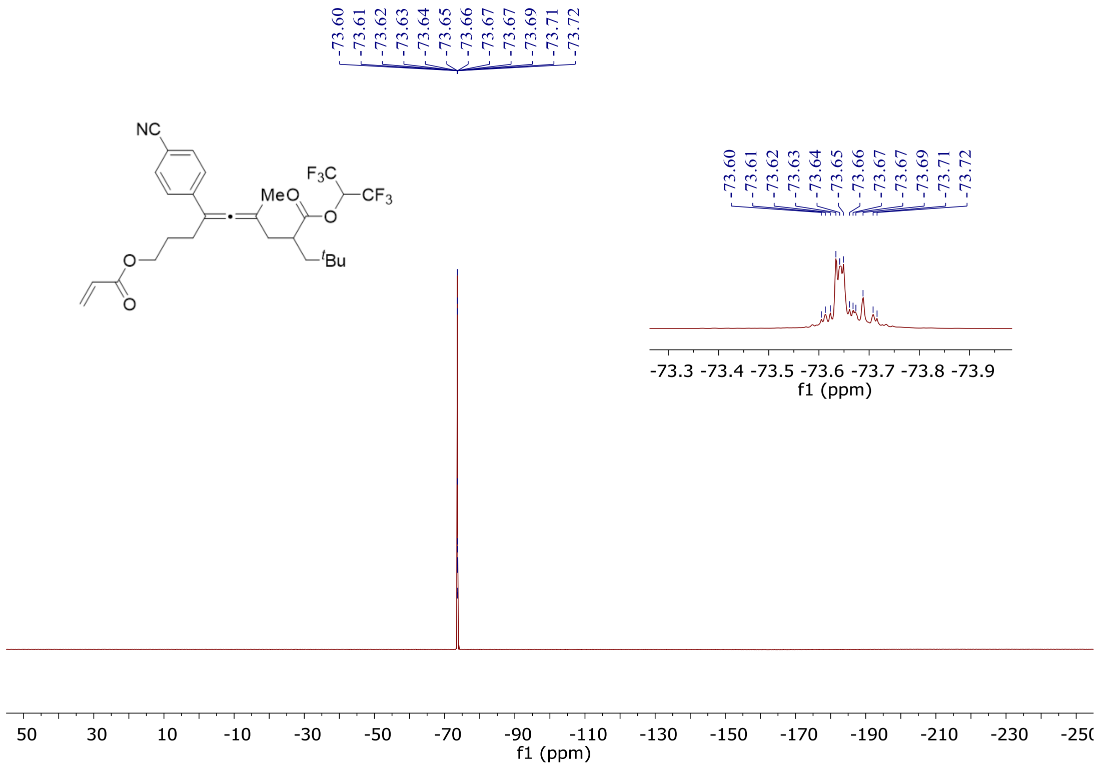

^{19}F{^{1}H} NMR (377 MHz, acetone-*d*$_6$) of **21**

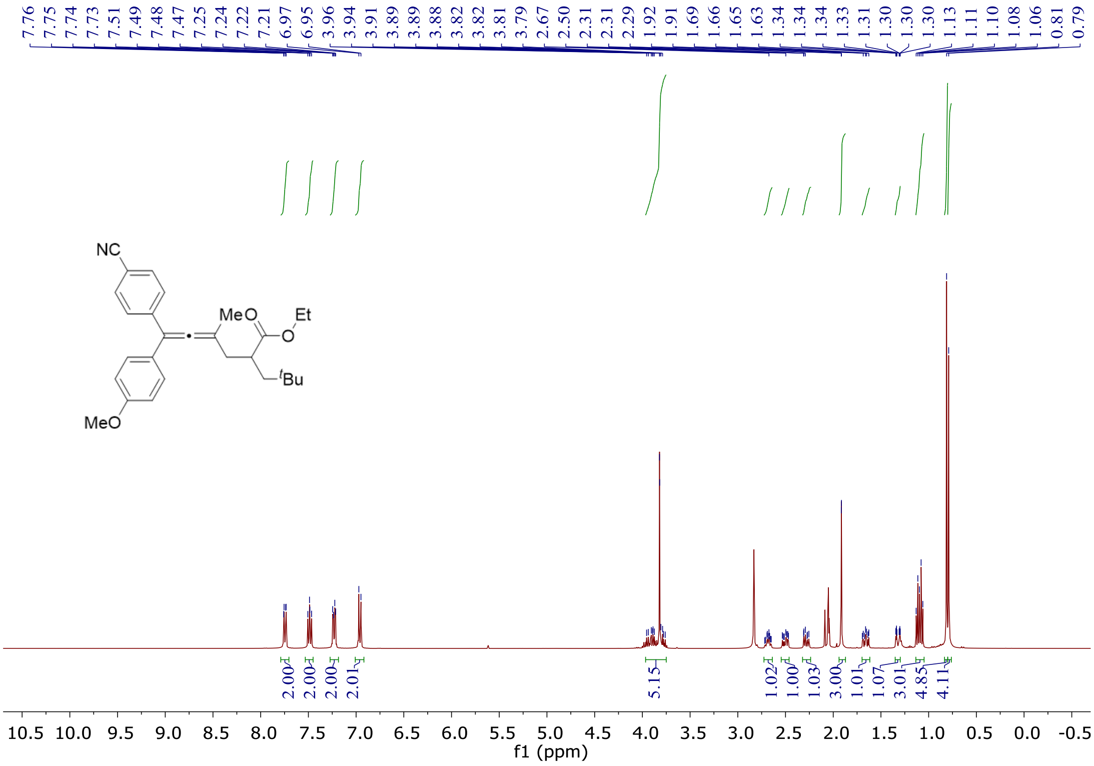

^{1}**H NMR** (400 MHz, acetone-d_6) of **22**

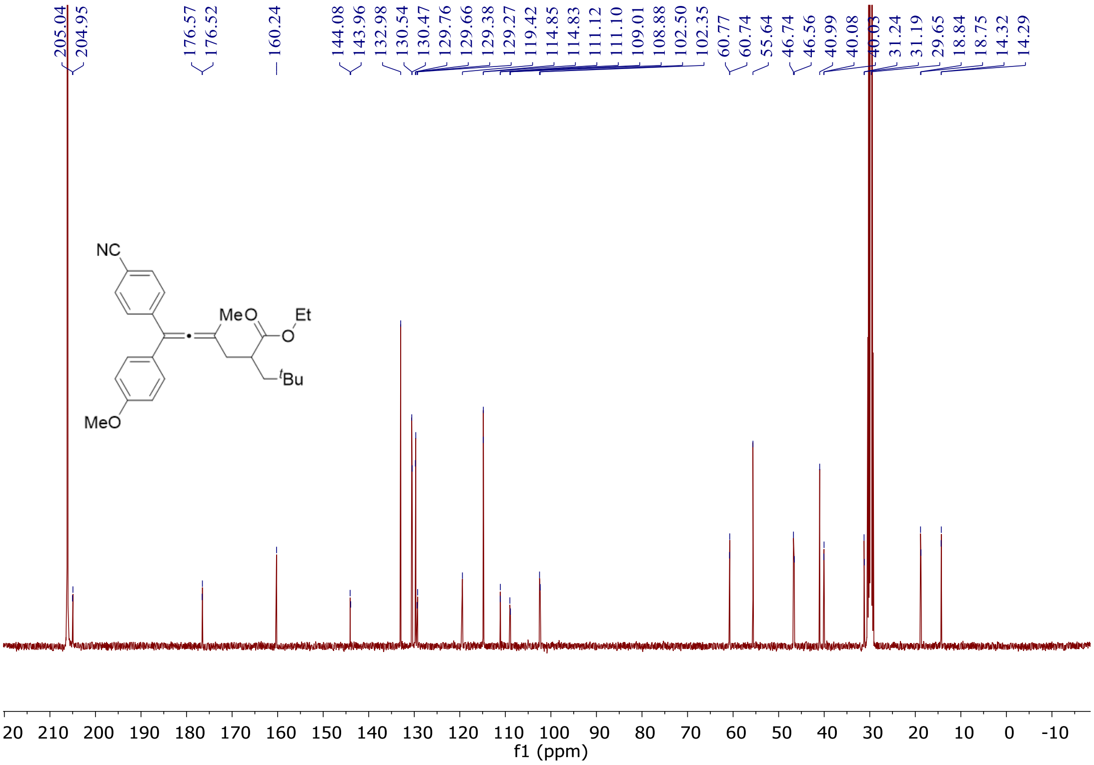

^{13}**C{^{1}H} NMR** (100 MHz, acetone-d_6) of **22**

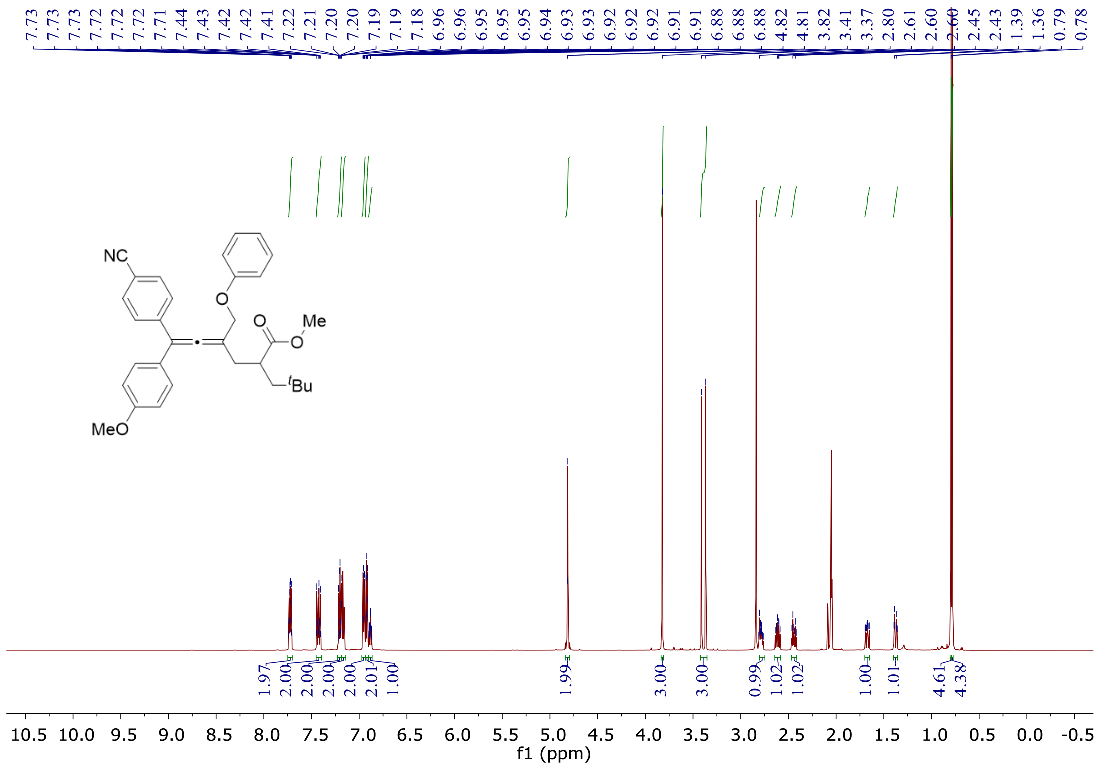

^{1}**H NMR** (400 MHz, acetone-d_6) of **23**

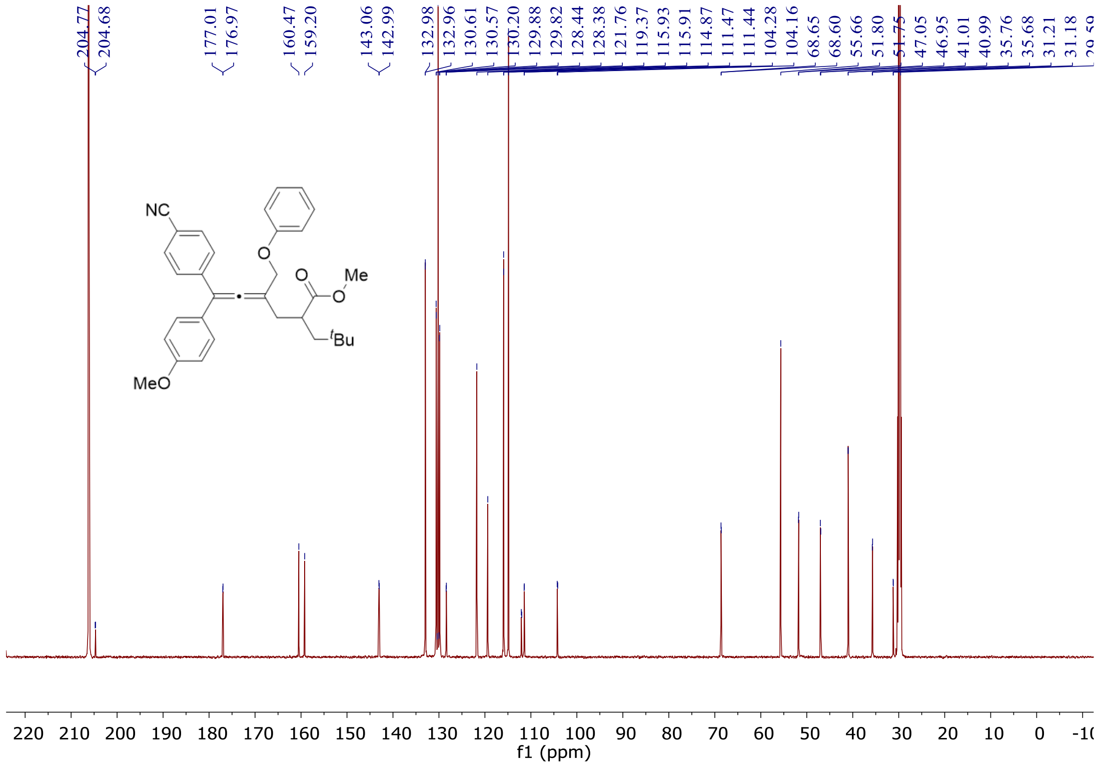

^{13}**C{^{1}H} NMR** (100 MHz, acetone-d_6) of **23**

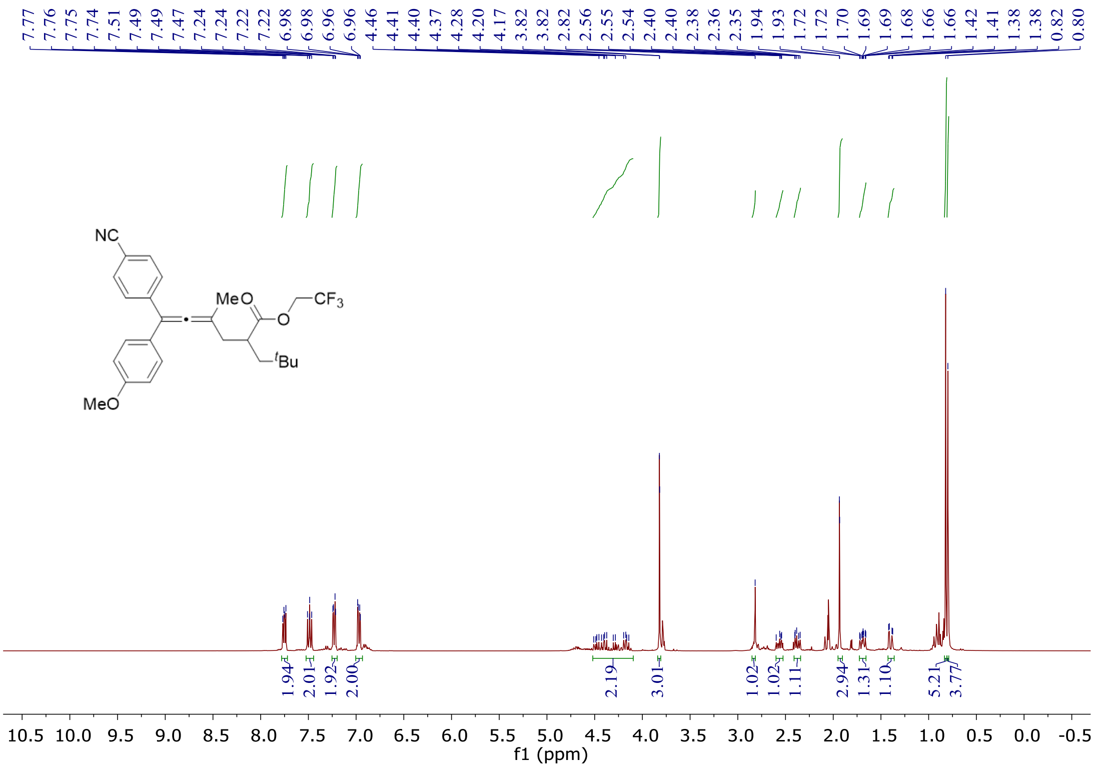

^{1}H NMR (400 MHz, acetone-d_6) of **24**

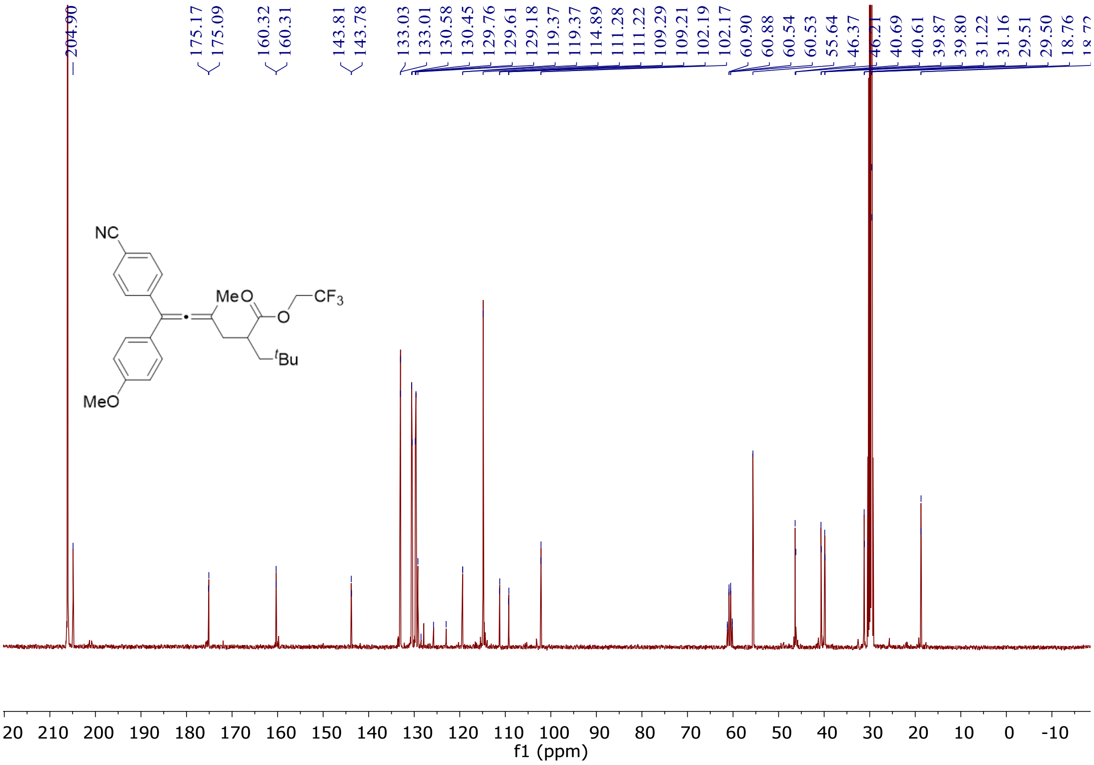

^{13}C{^{1}H} NMR (100 MHz, acetone-d_6) of **24**

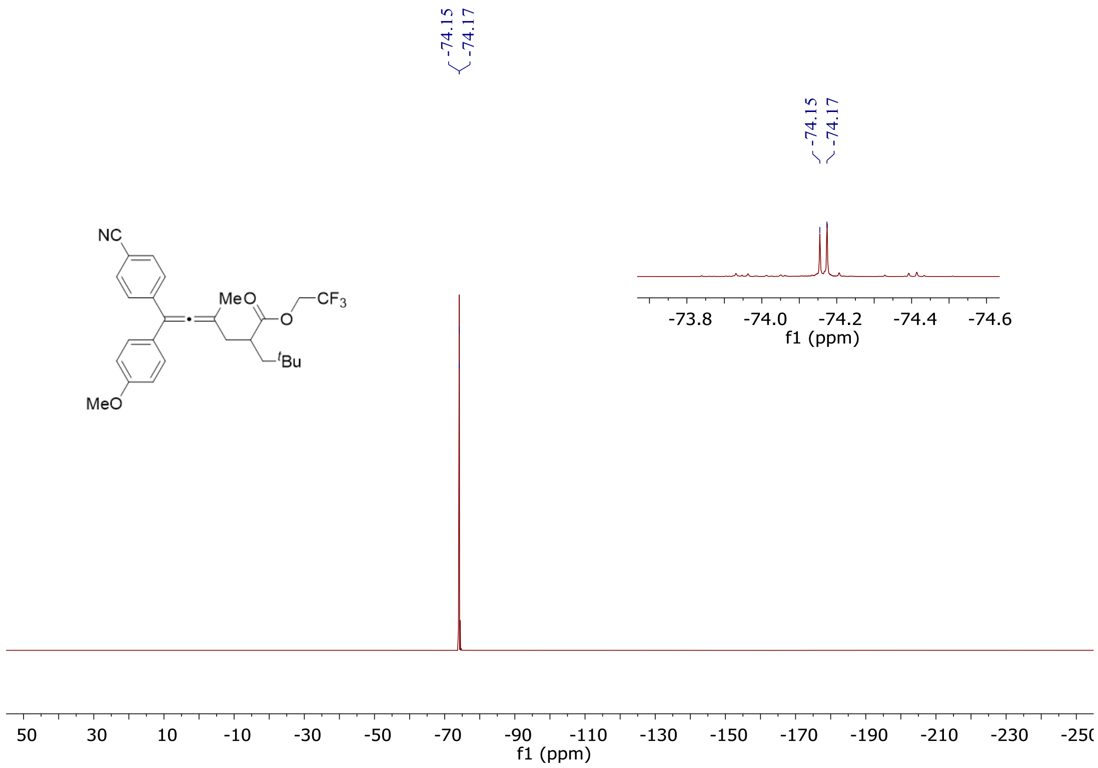

$^{19}F\{^{1}H\}$ **NMR** (377 MHz, acetone-d_6) of **24**

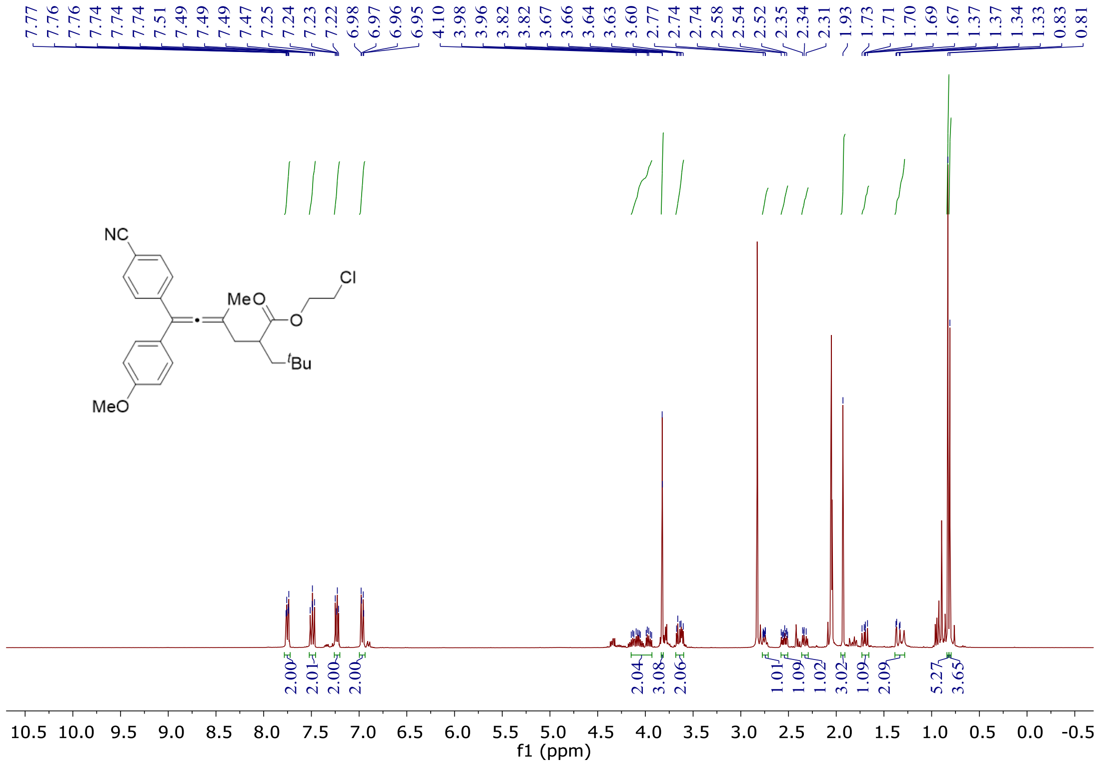

^{1}H **NMR** (400 MHz, acetone-d_6) of **25**

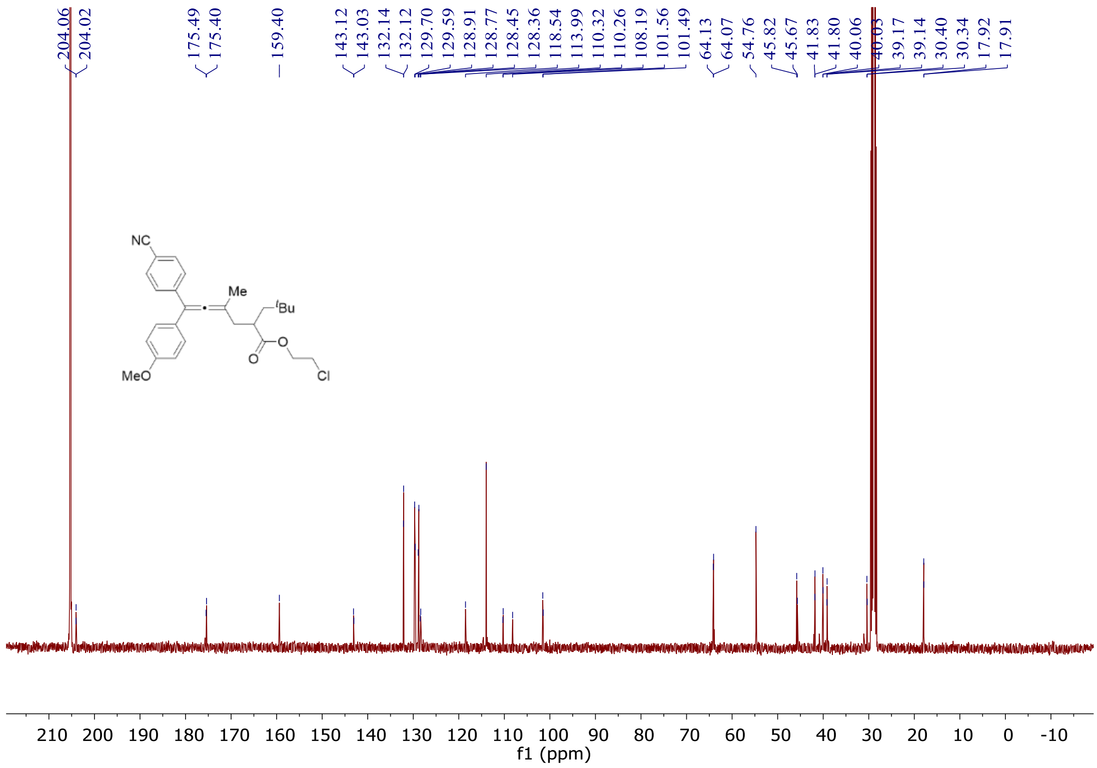

^{13}C{^{1}H} NMR (100 MHz, acetone-d_6) of **25**

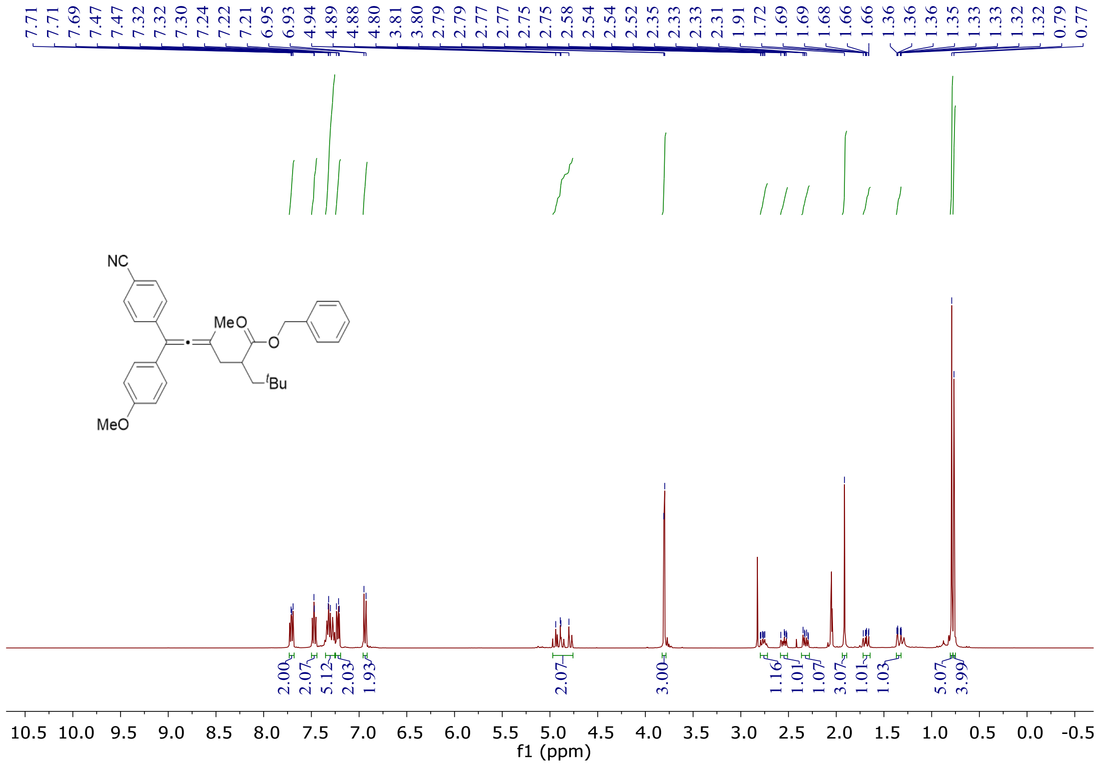

^{1}H NMR (400 MHz, acetone-d_6) of **26**

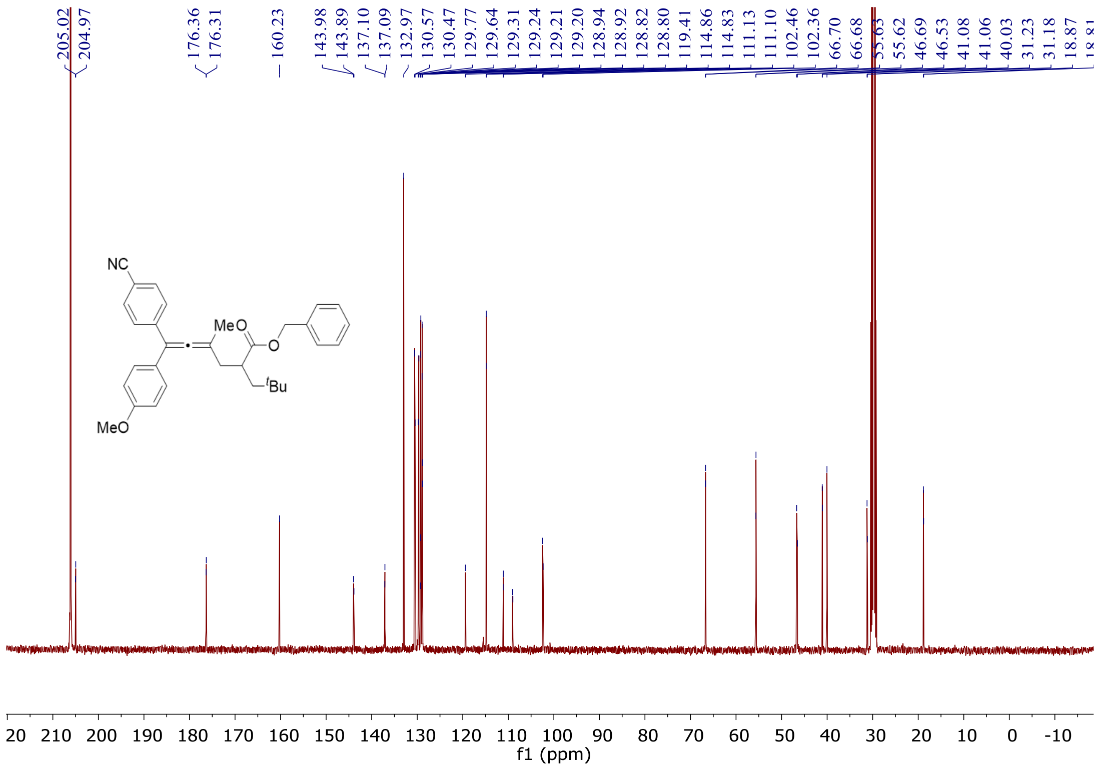

$^{13}C\{^1H\}$ NMR (100 MHz, acetone-d_6) of **26**

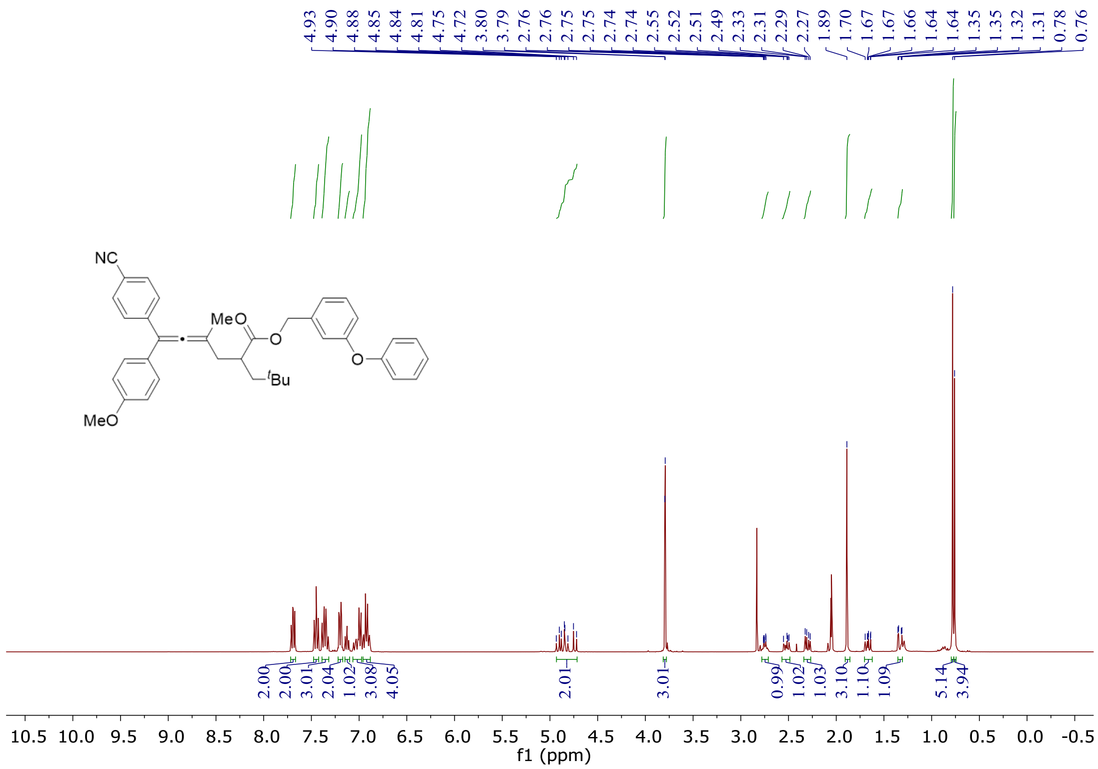

^1H NMR (400 MHz, acetone-d_6) of **27**

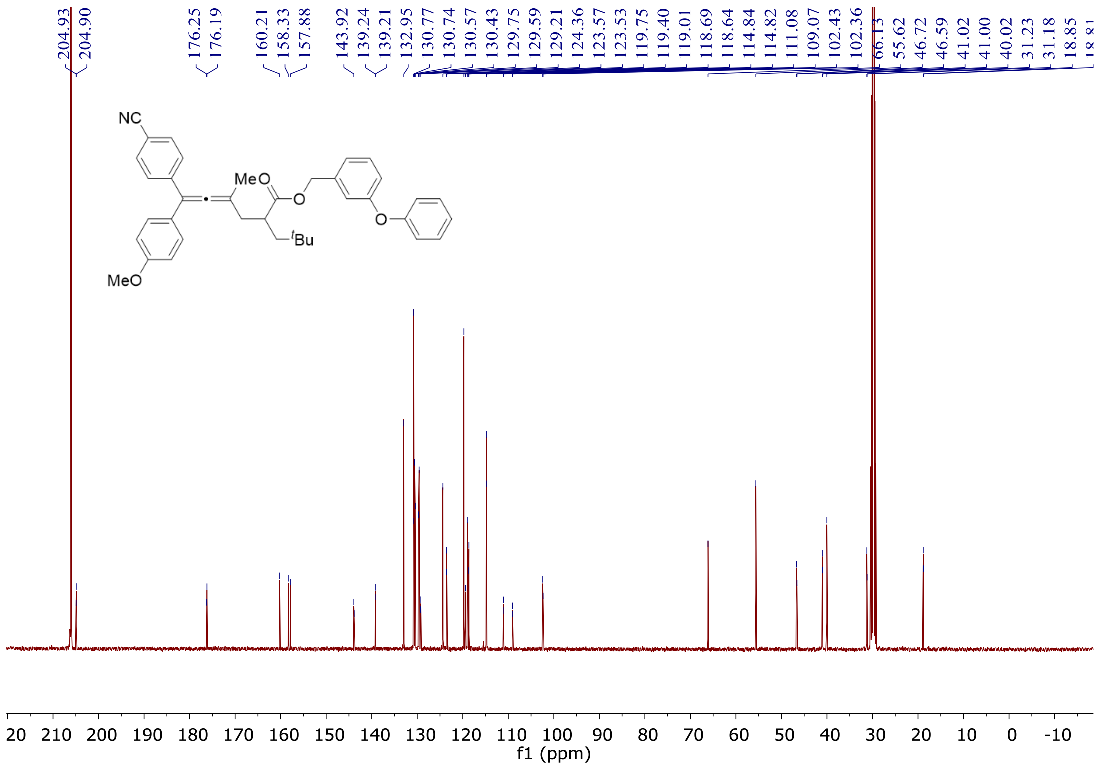

$^{13}C\{^{1}H\}$ **NMR** (100 MHz, acetone-d_6) of **27**

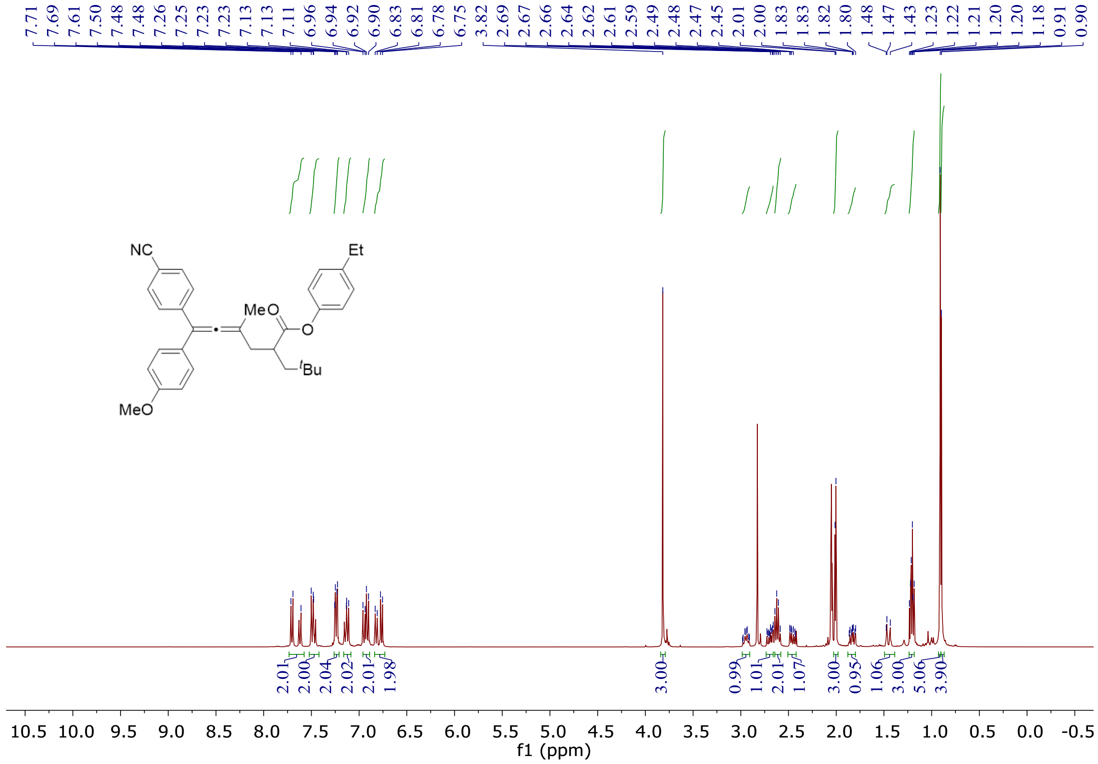

^{1}H **NMR** (400 MHz, acetone-d_6) of **28**

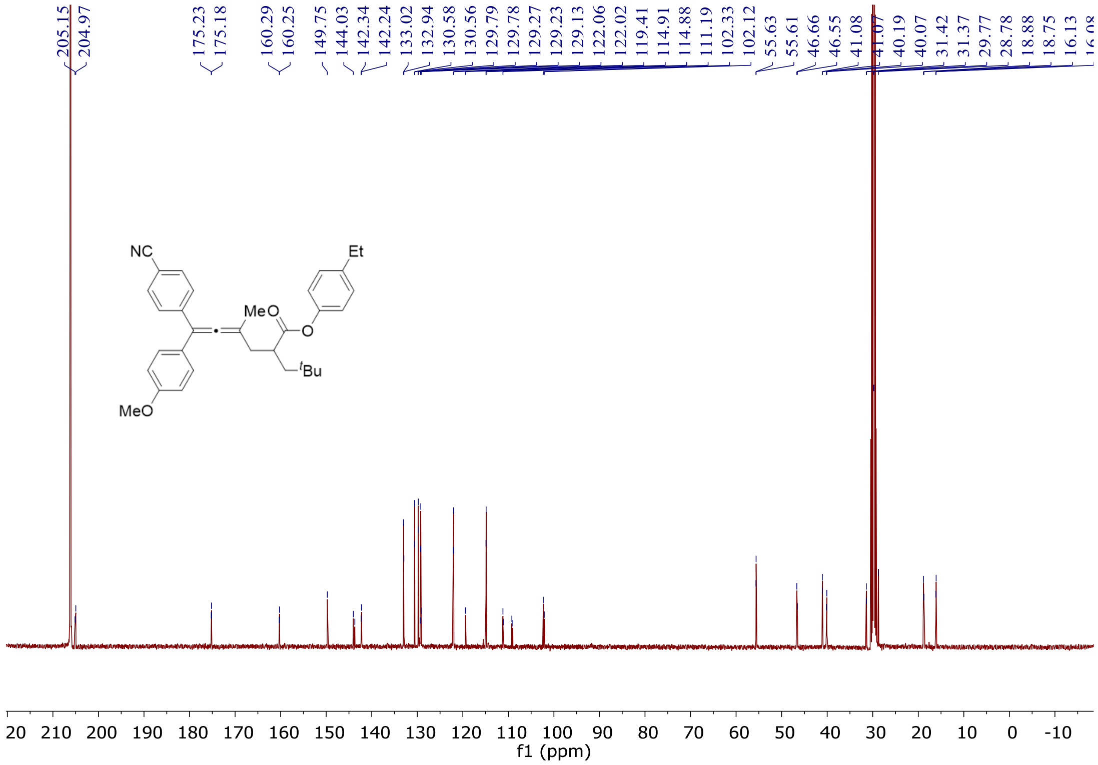

^{13}C{^{1}H} **NMR** (100 MHz, acetone-d_6) of **28**

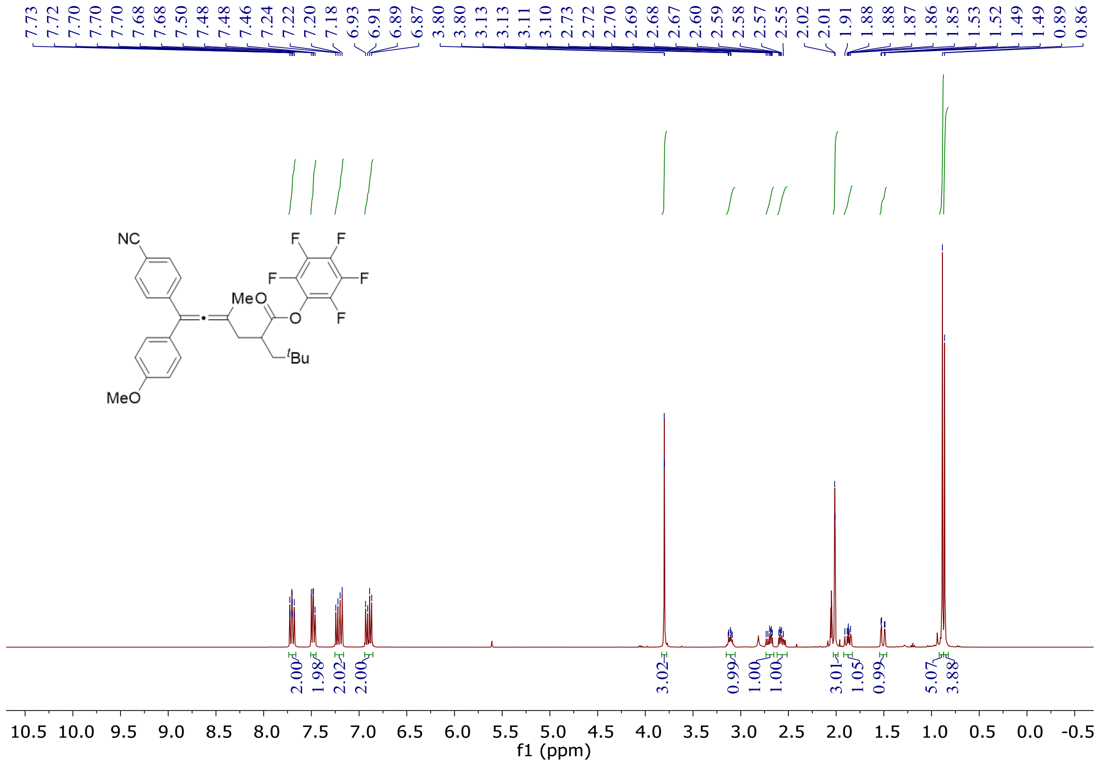

^{1}**H NMR** (400 MHz, acetone-d_6) of **29**

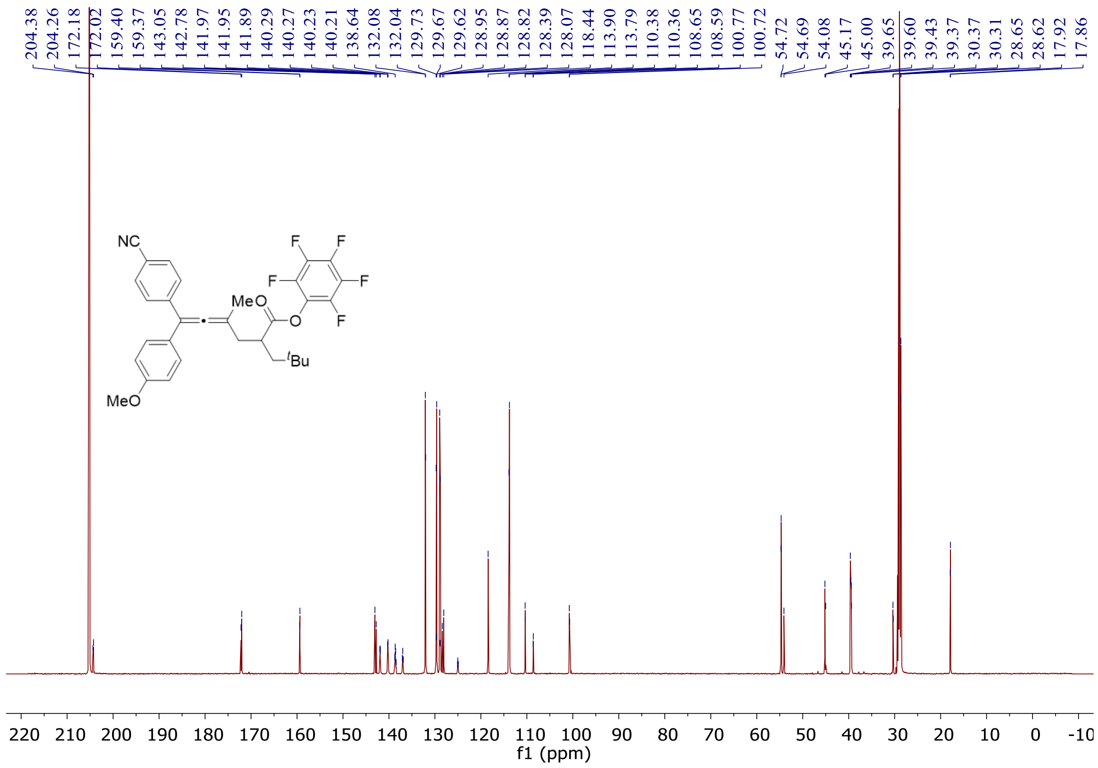

$^{13}C\{^1H\}$ **NMR** (100 MHz, acetone-d_6) of **29**

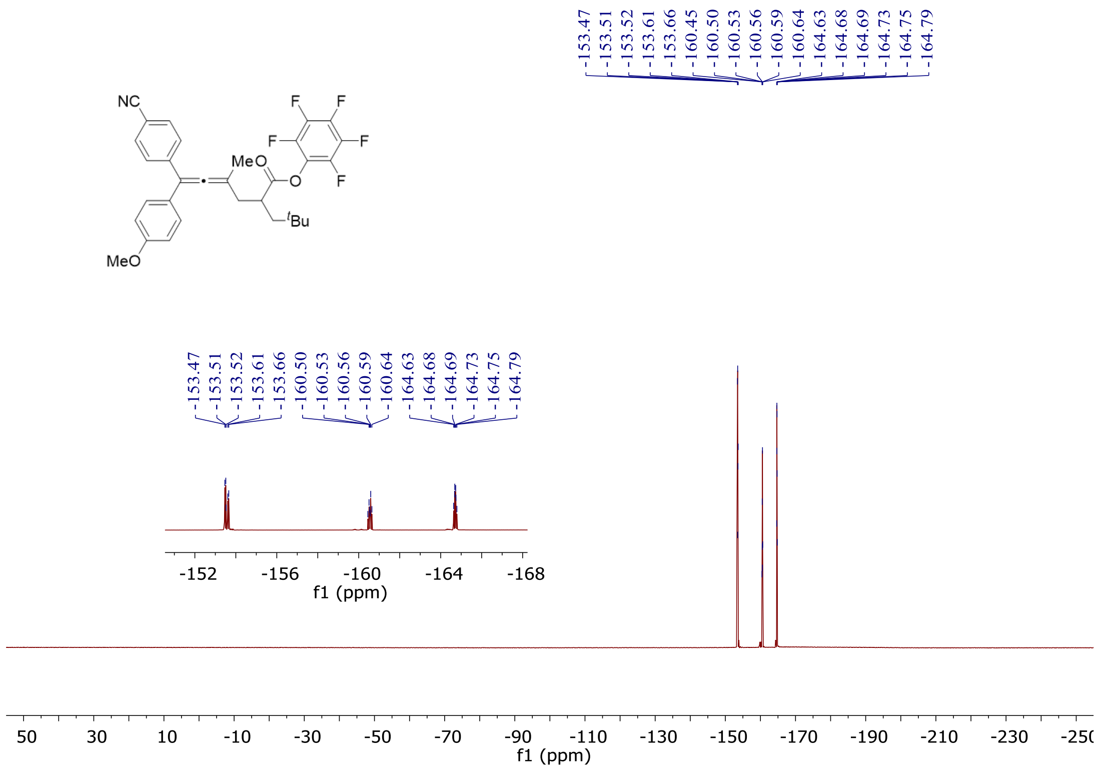

$^{19}F\{^1H\}$ **NMR** (377 MHz, acetone-d_6) of **29**

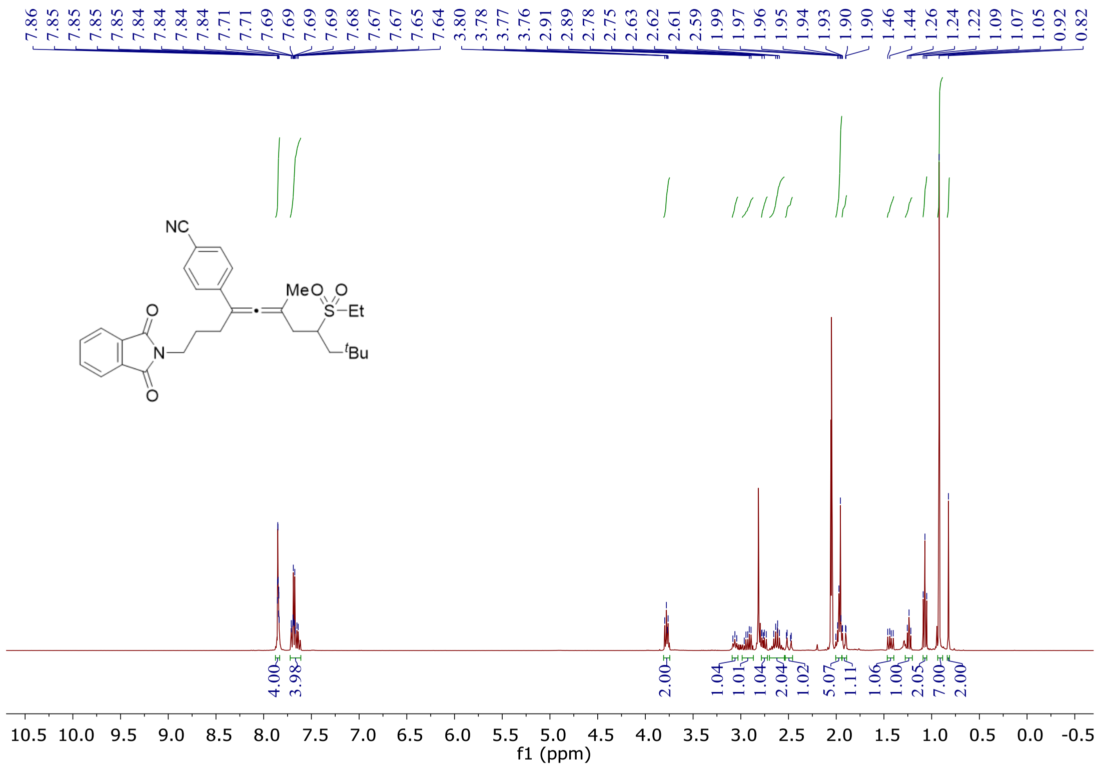

^{1}**H NMR** (400 MHz, acetone-d_6) of **30**

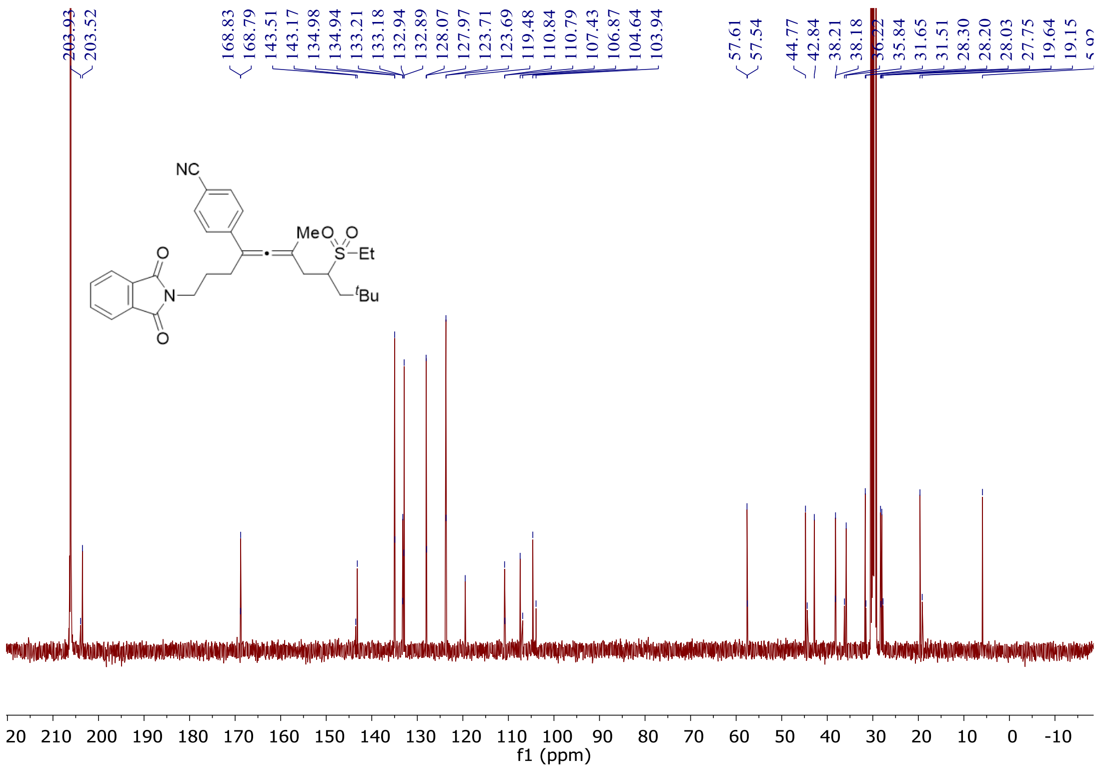

^{13}**C{^{1}H} NMR** (100 MHz, acetone-d_6) of **30**

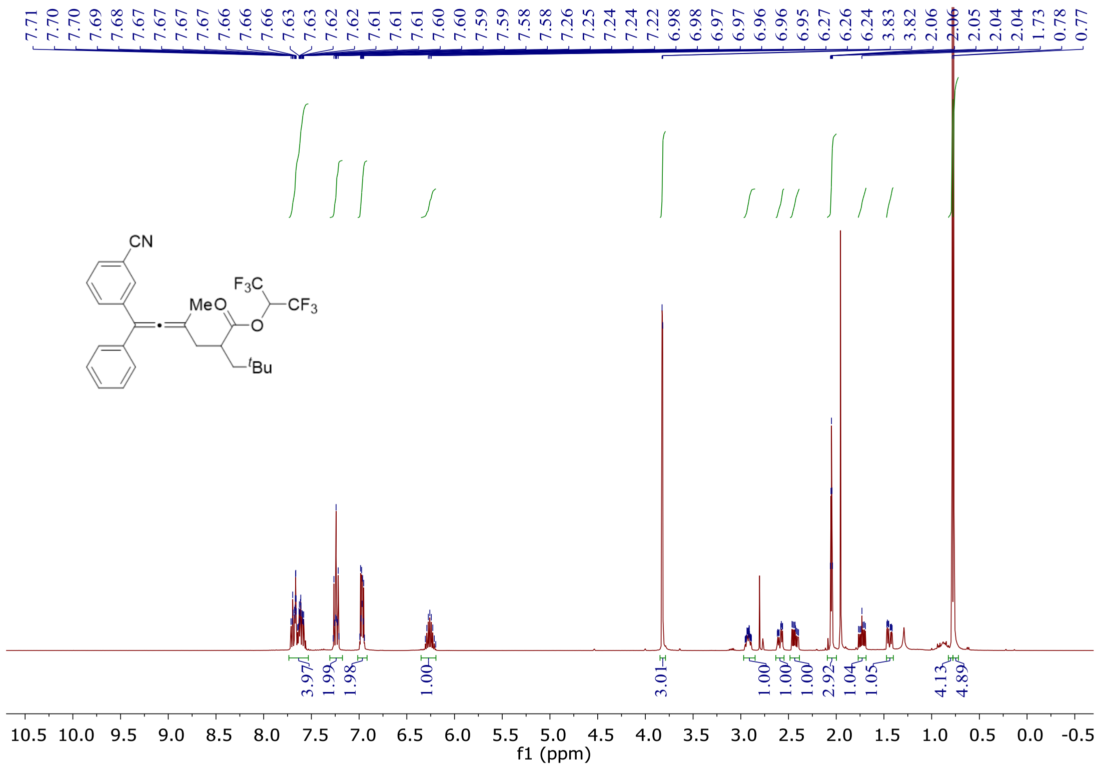

^{1}**H NMR** (400 MHz, acetone-d_6) of **31**

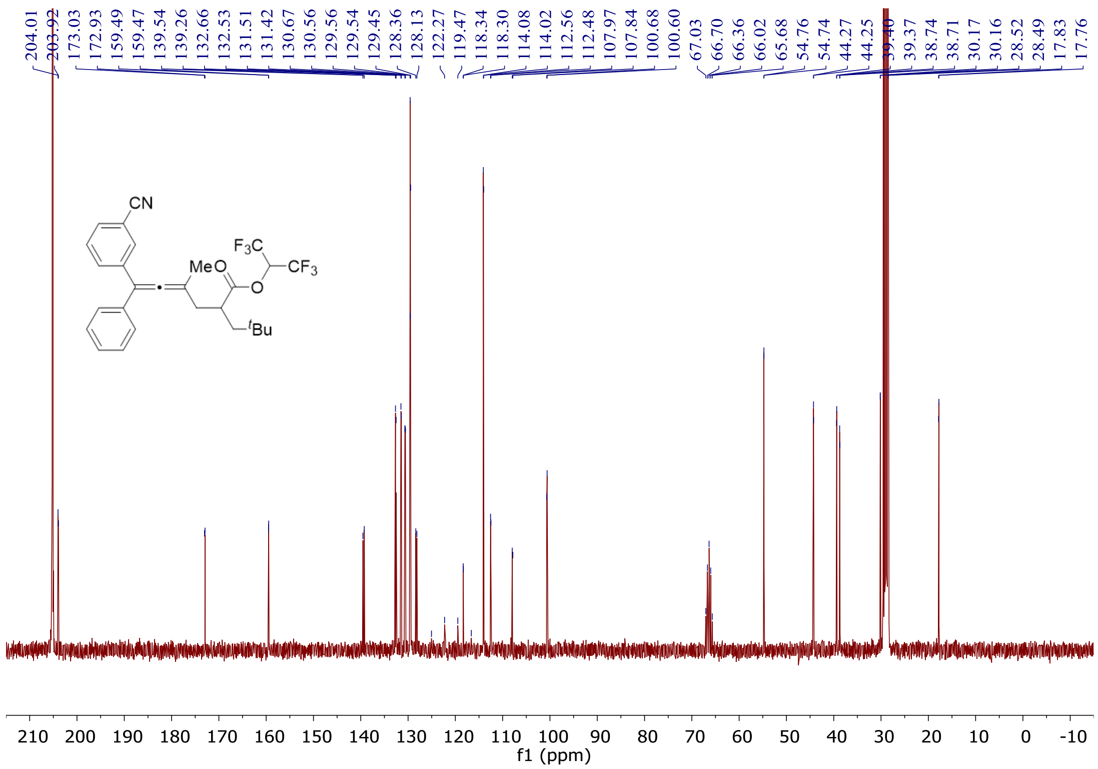

^{13}**C{^{1}H} NMR** (100 MHz, acetone-d_6) of **31**

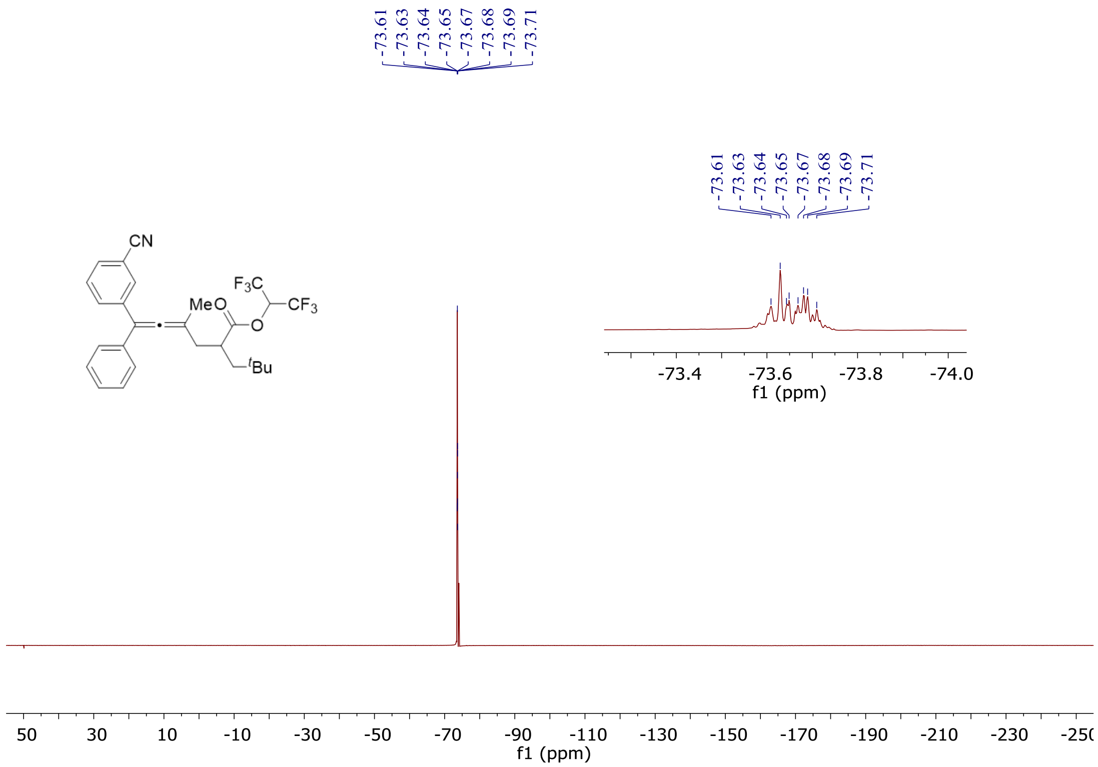

^{19}F{^{1}H} NMR (377 MHz, acetone-d_6) of **31**

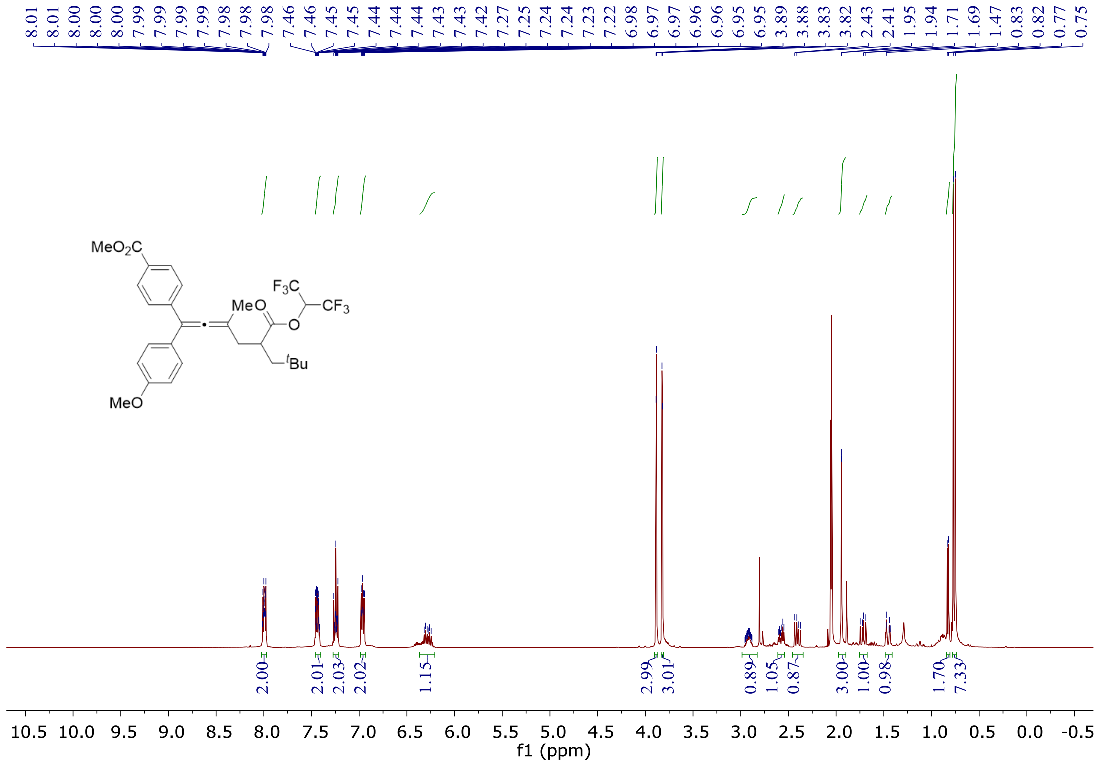

^{1}H NMR (400 MHz, acetone-d_6) of **32**

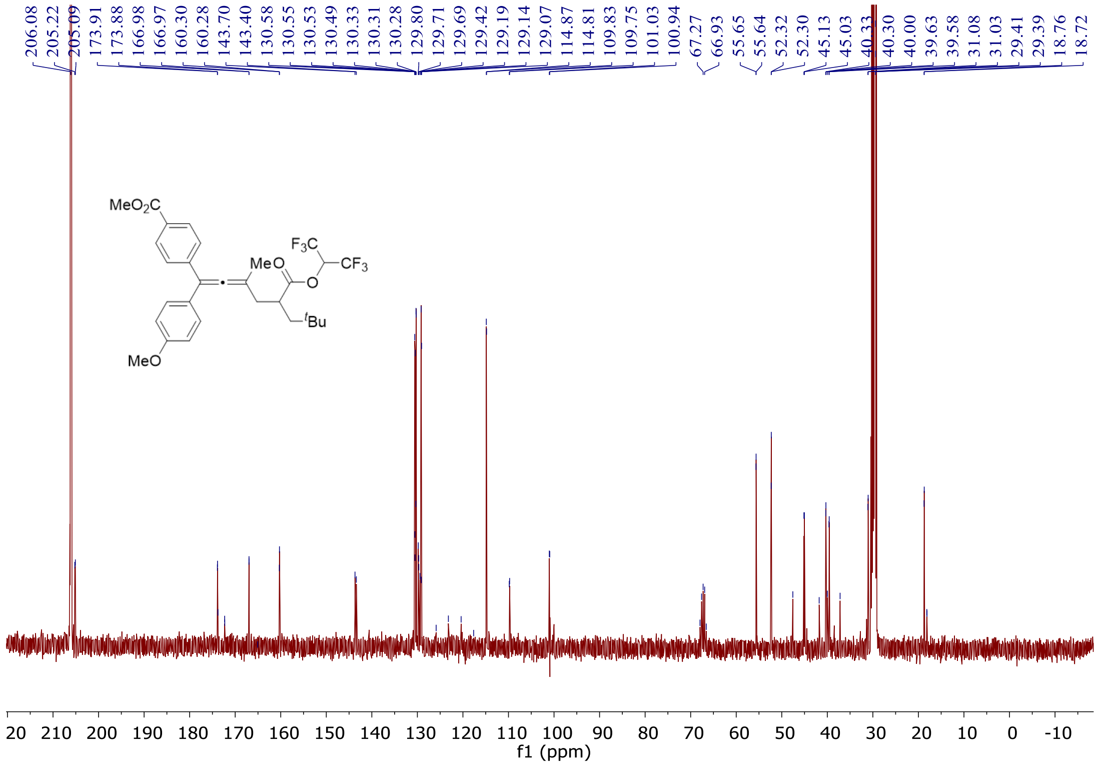

$^{13}C\{^1H\}$ NMR (100 MHz, acetone-d_6) of **32**

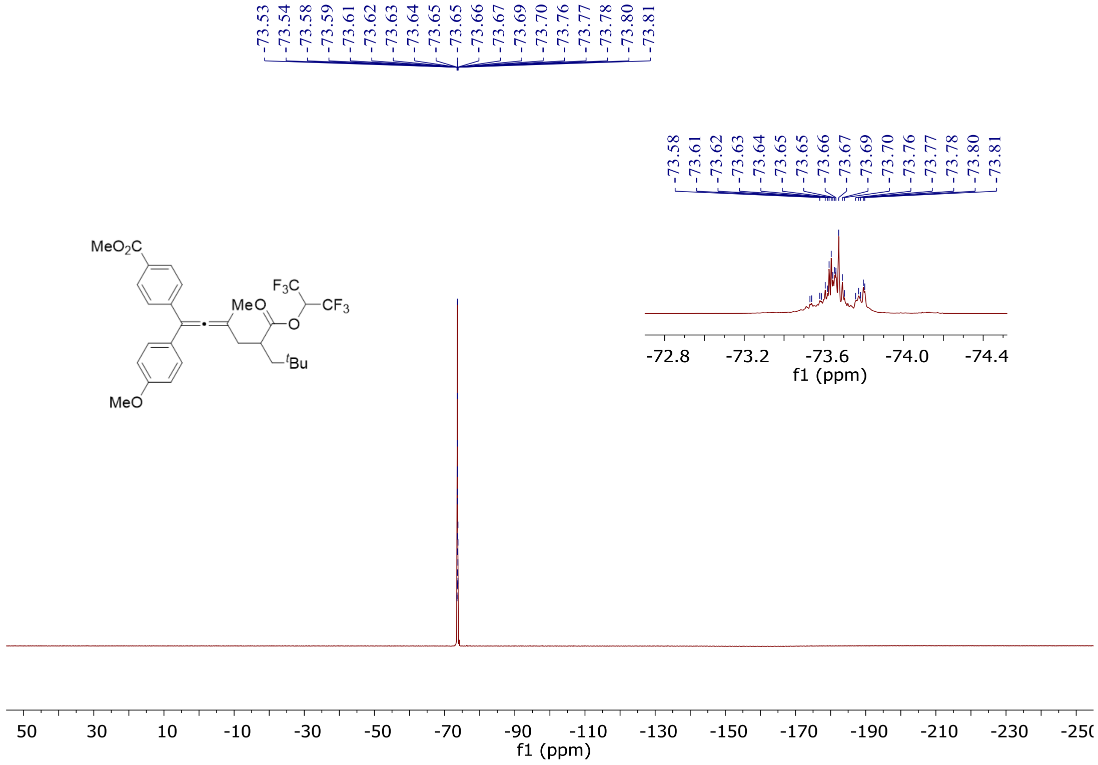

$^{19}F\{^1H\}$ NMR (377 MHz, acetone-d_6) of **32**

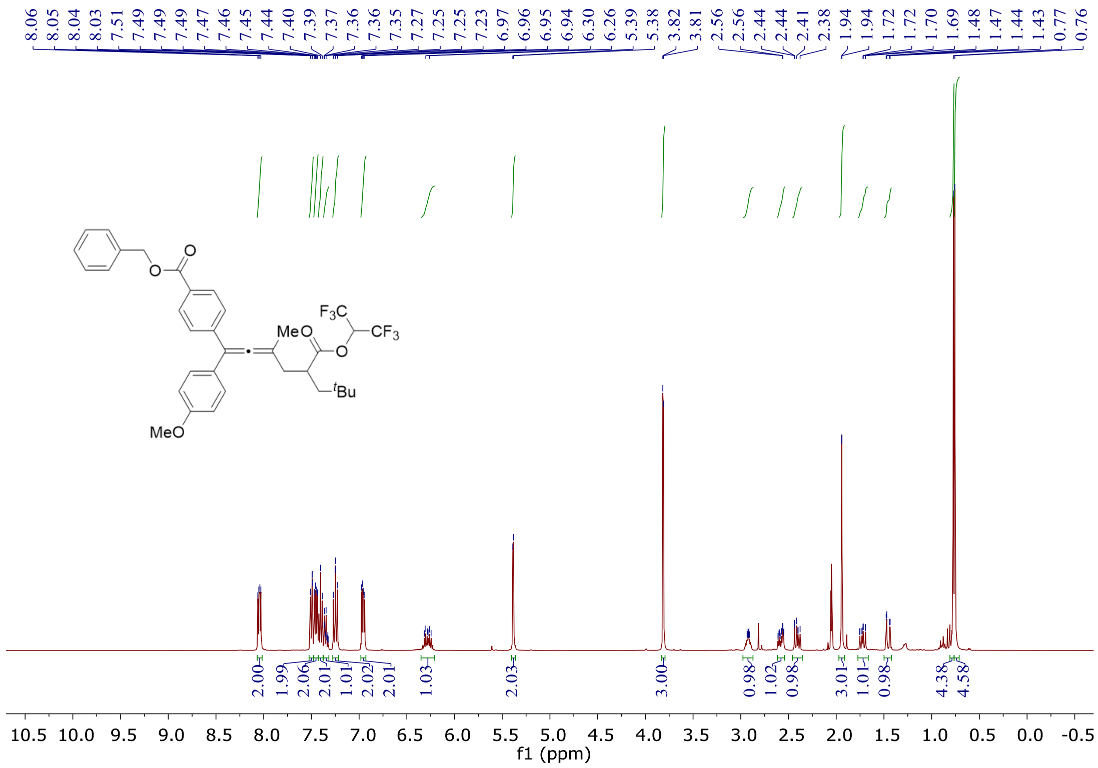

^{1}**H NMR** (400 MHz, acetone-d_6) of **33**

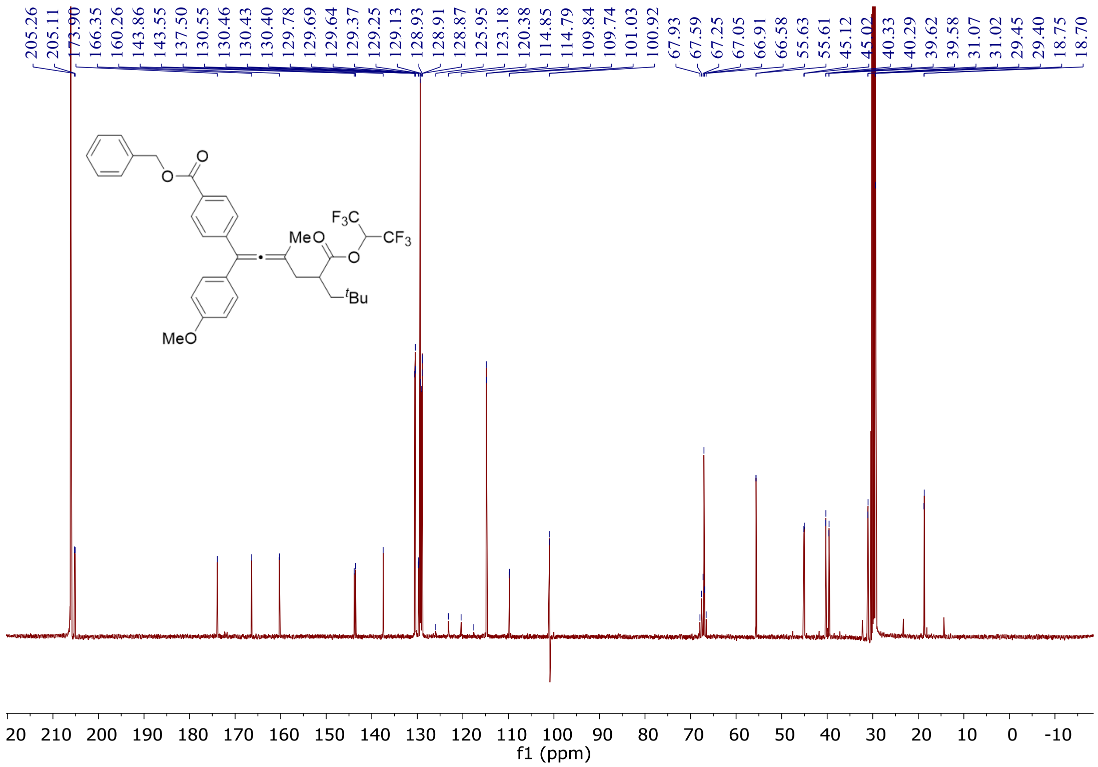

^{13}**C{**^{1}**H} NMR** (100 MHz, acetone-d_6) of **33**

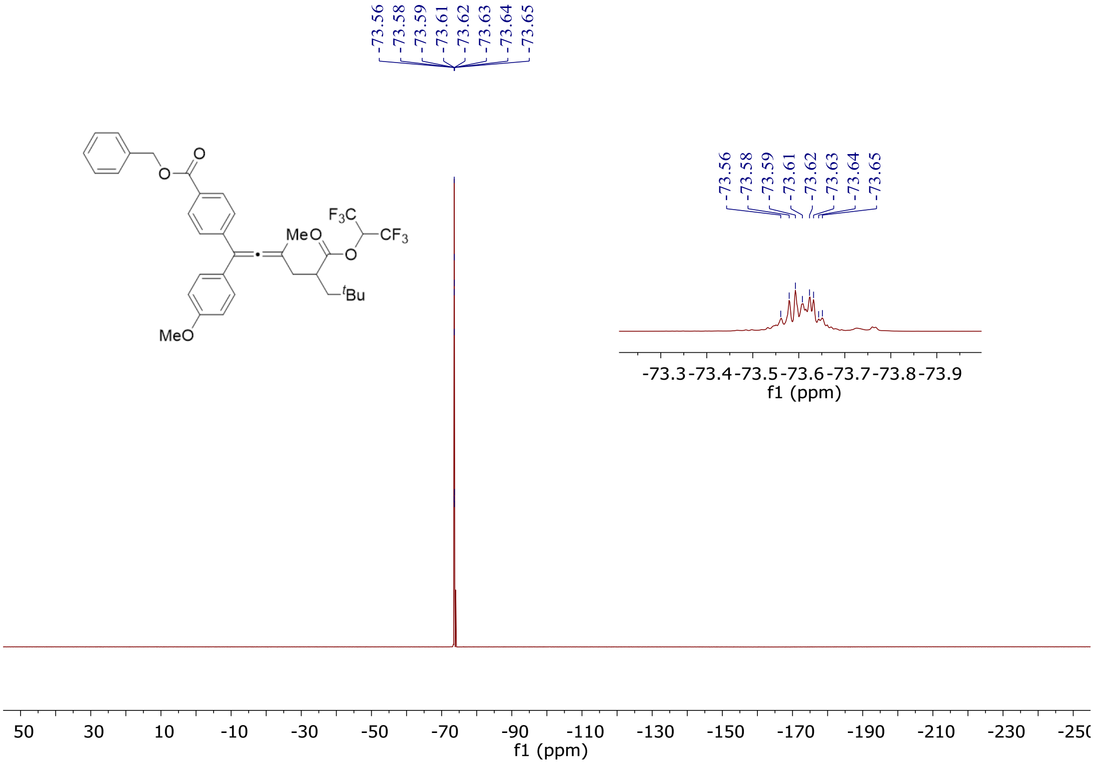

^{19}F{^{1}H} NMR (377 MHz, acetone-d_6) of **33**

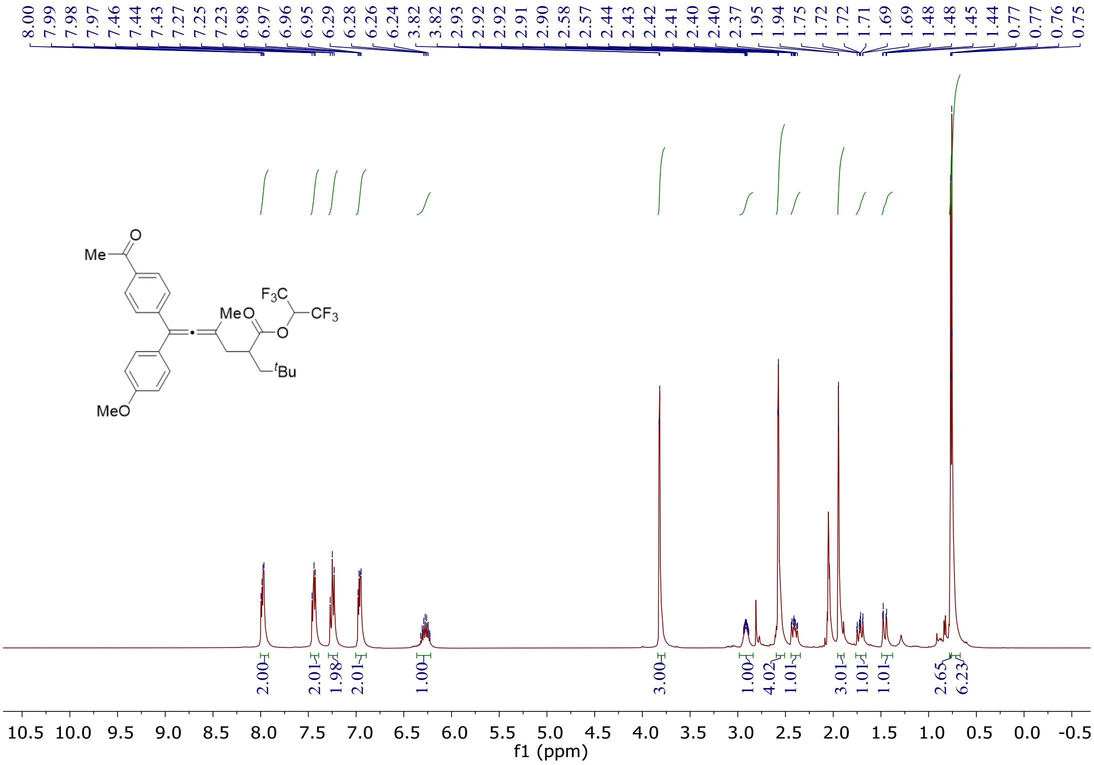

^{1}H NMR (400 MHz, acetone-d_6) of **34**

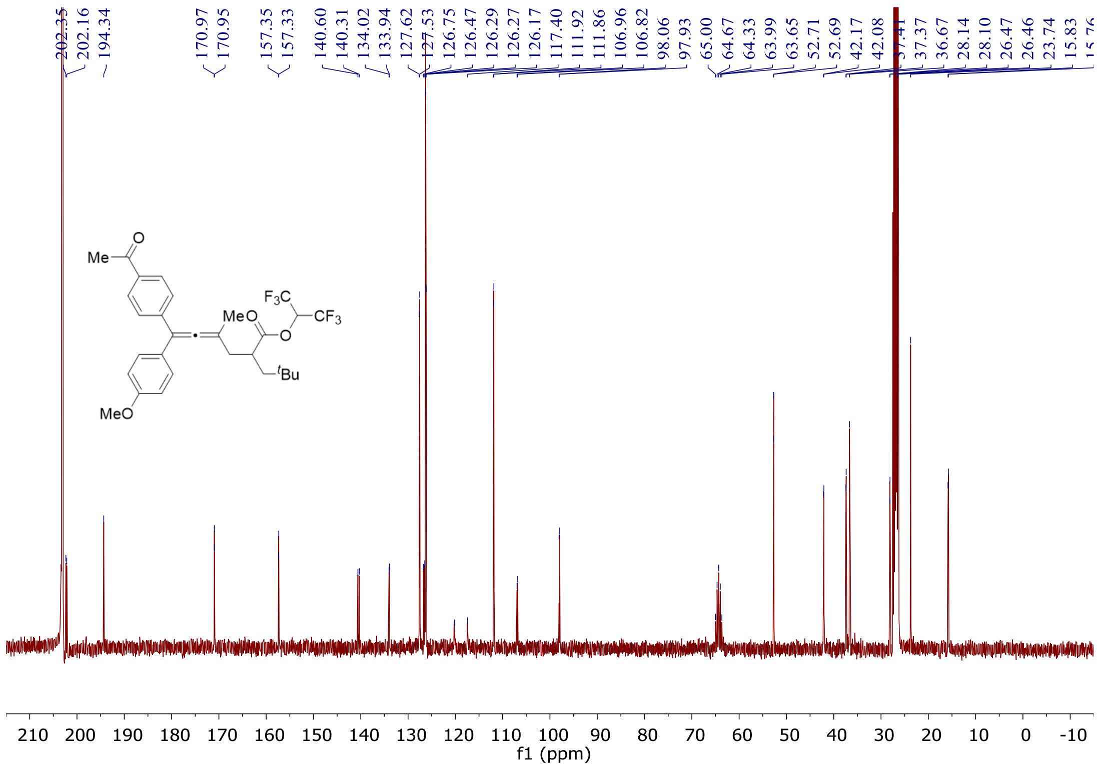

$^{13}C\{^1H\}$ **NMR** (100 MHz, acetone-d_6) of **34**

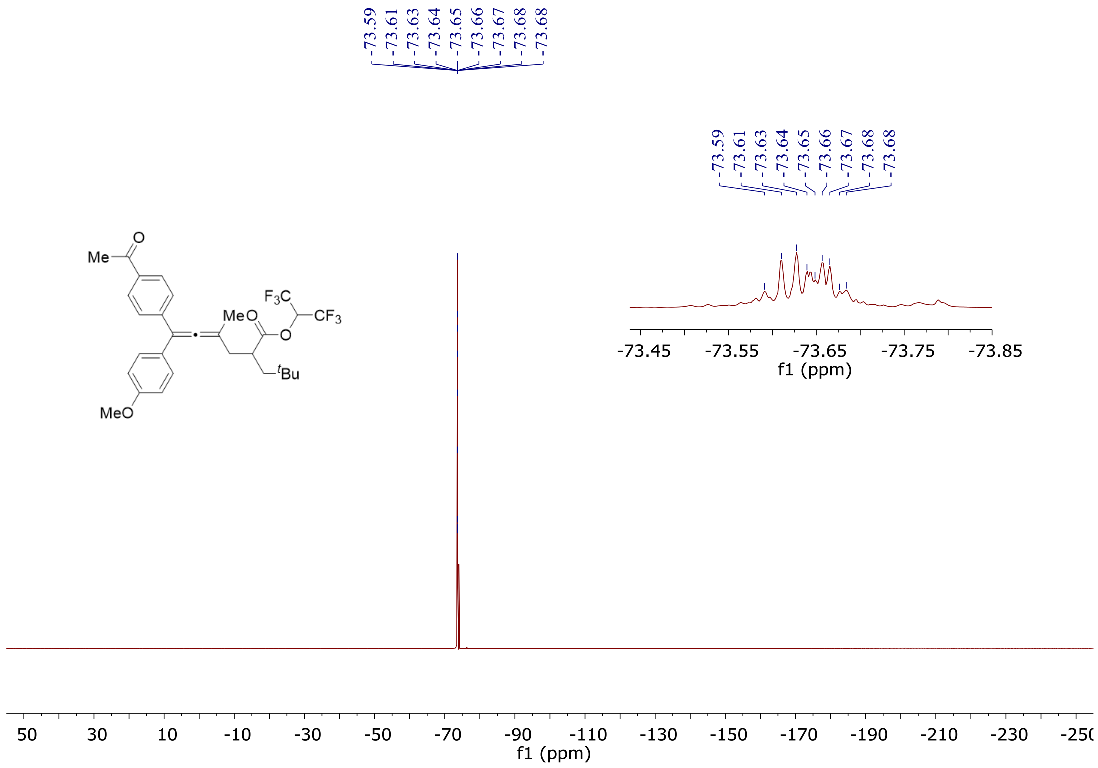

$^{19}F\{^1H\}$ **NMR** (377 MHz, acetone-d_6) of **34**

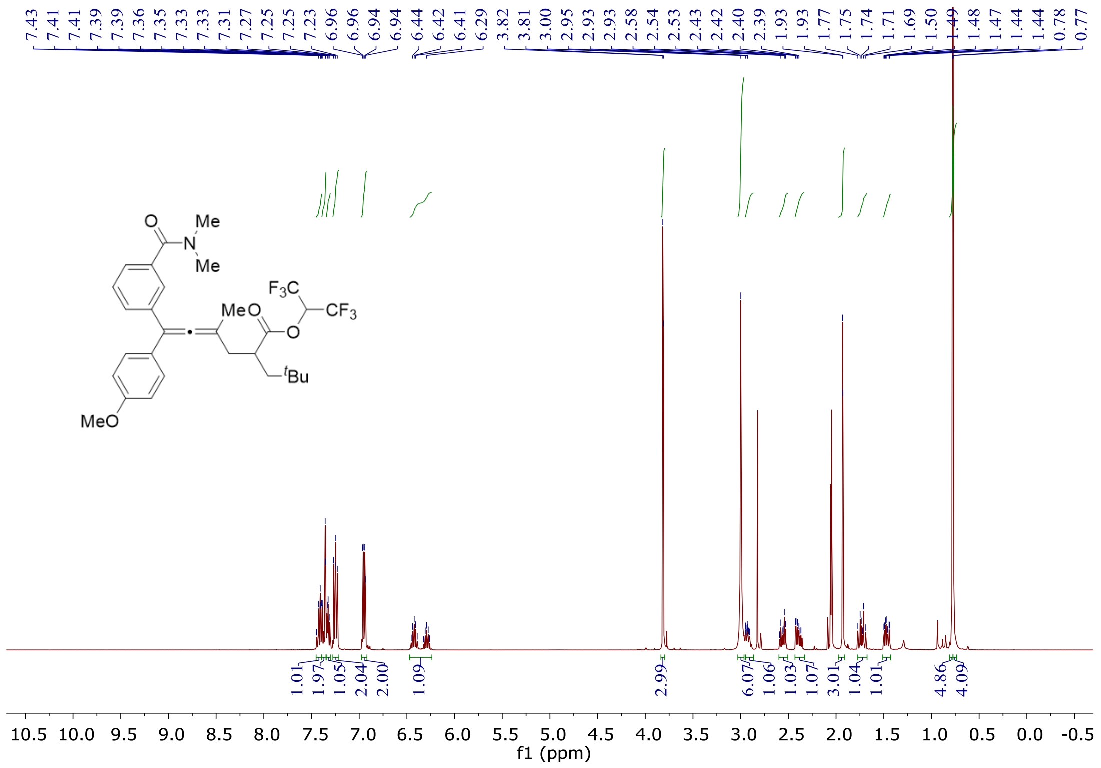

^{1}**H NMR** (400 MHz, acetone-d_6) of **35**

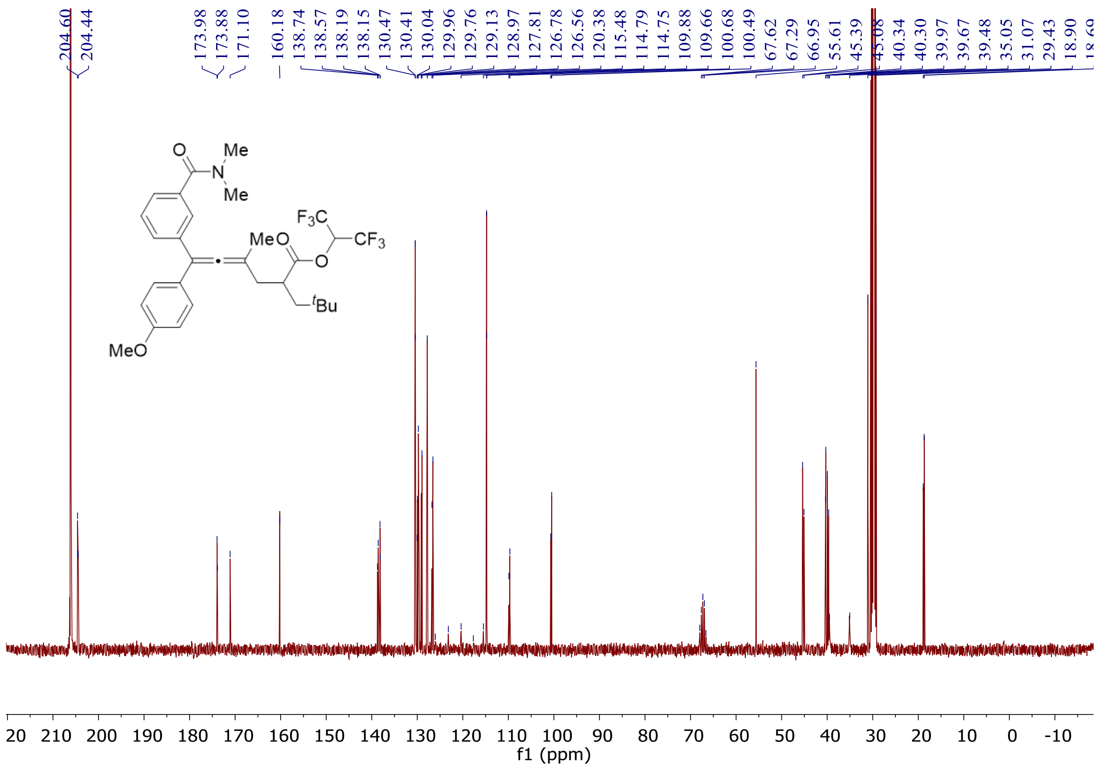

^{13}**C{^{1}H} NMR** (100 MHz, acetone-d_6) of **35**

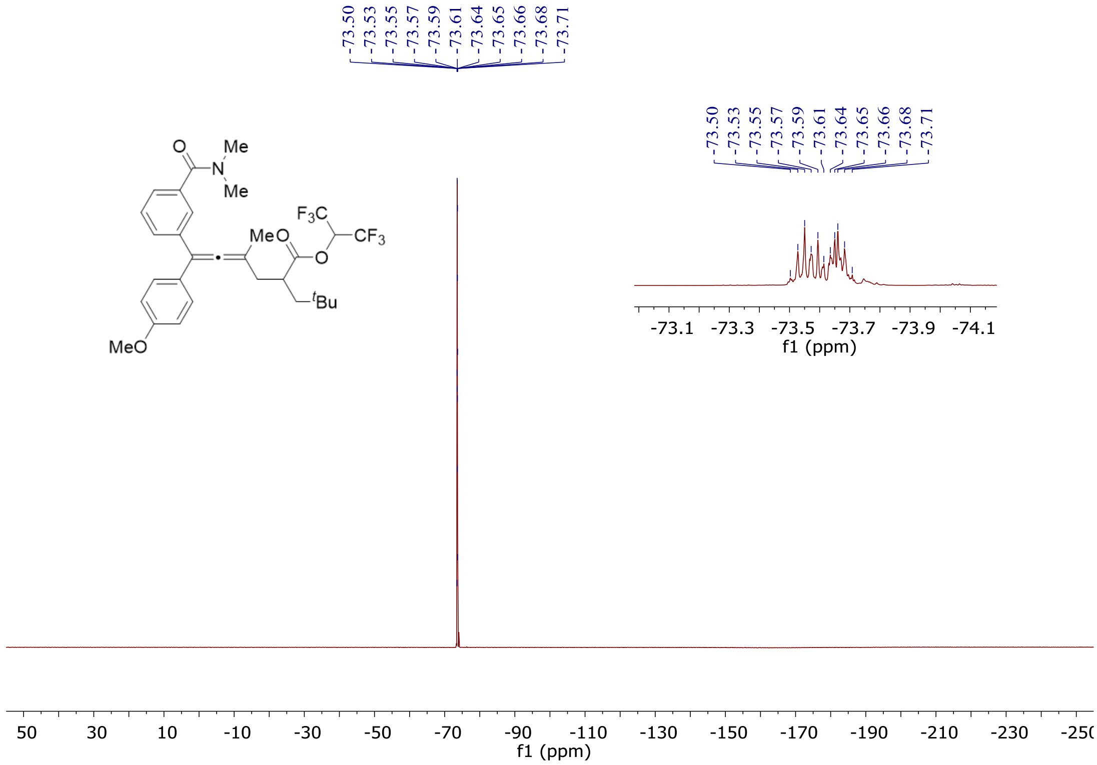

$^{19}F\{^{1}H\}$ **NMR** (377 MHz, acetone-d_6) of **35**

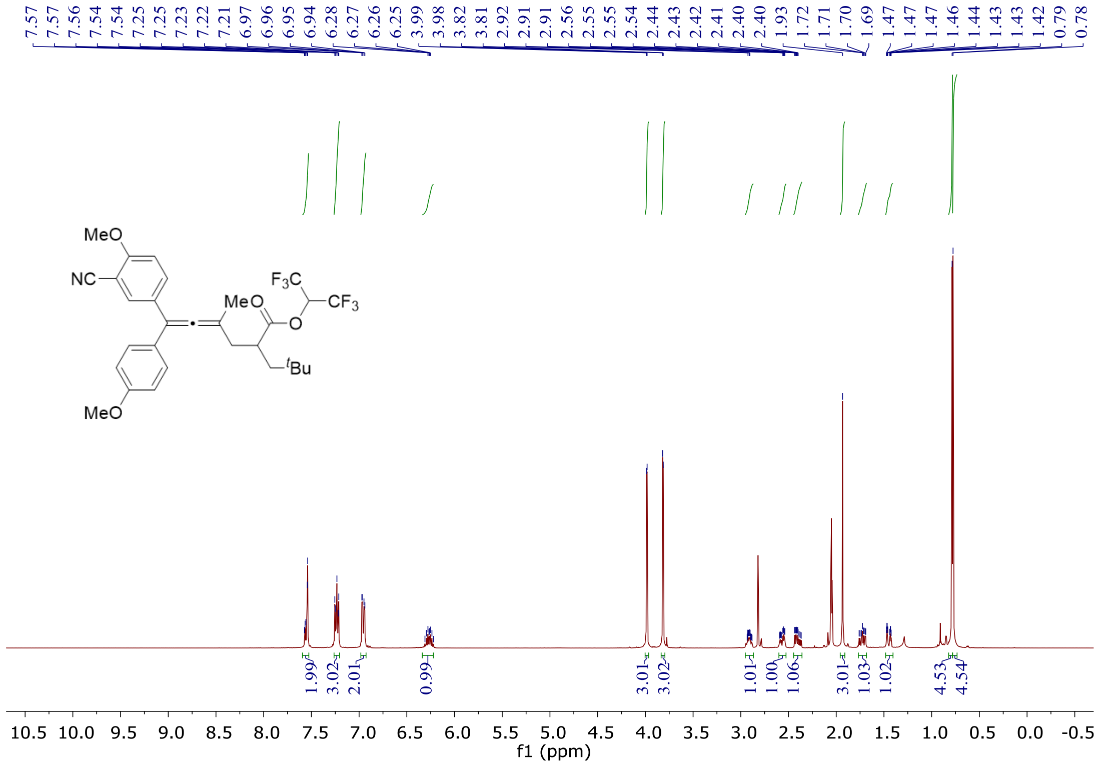

^{1}H **NMR** (400 MHz, acetone-d_6) of **36**

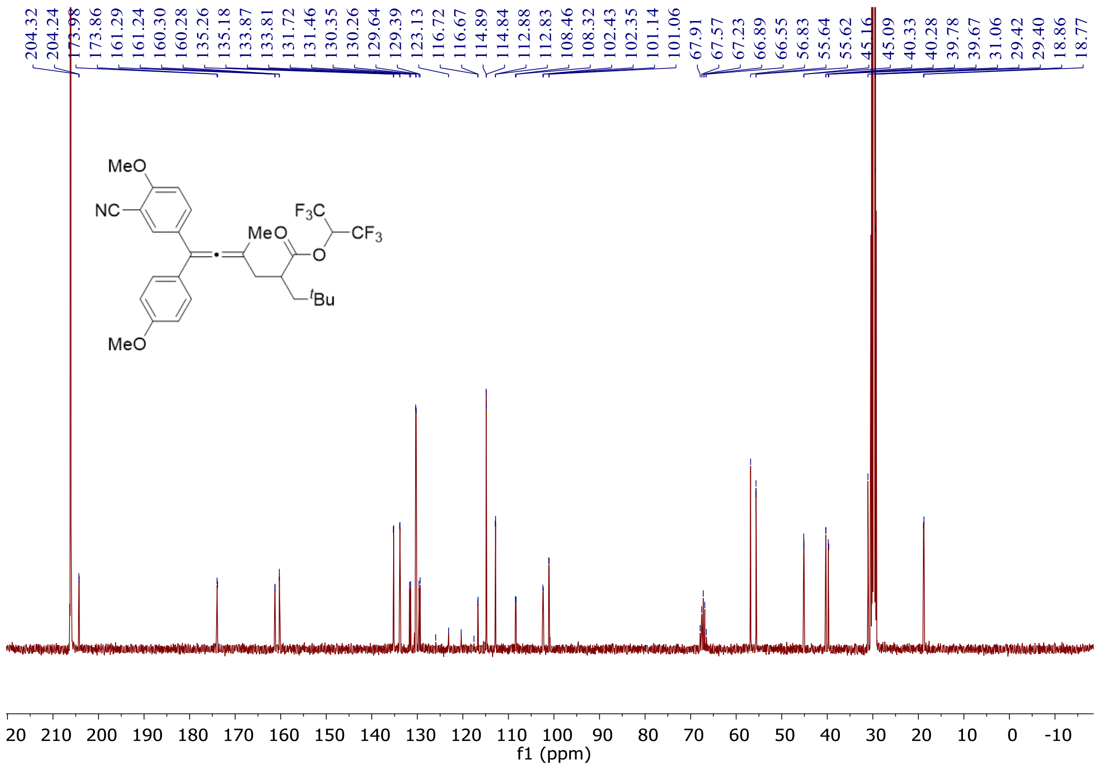

$^{13}C\{^{1}H\}$ NMR (100 MHz, acetone-d_6) of **36**

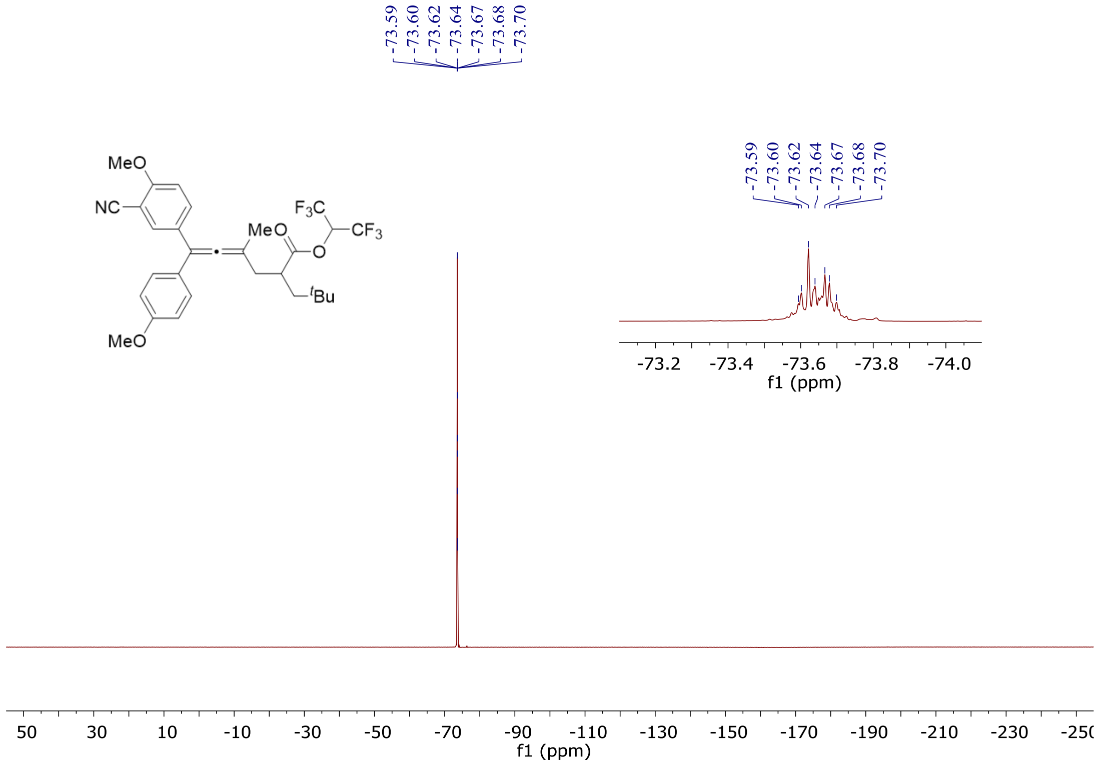

$^{19}F\{^{1}H\}$ NMR (377 MHz, acetone-d_6) of **36**

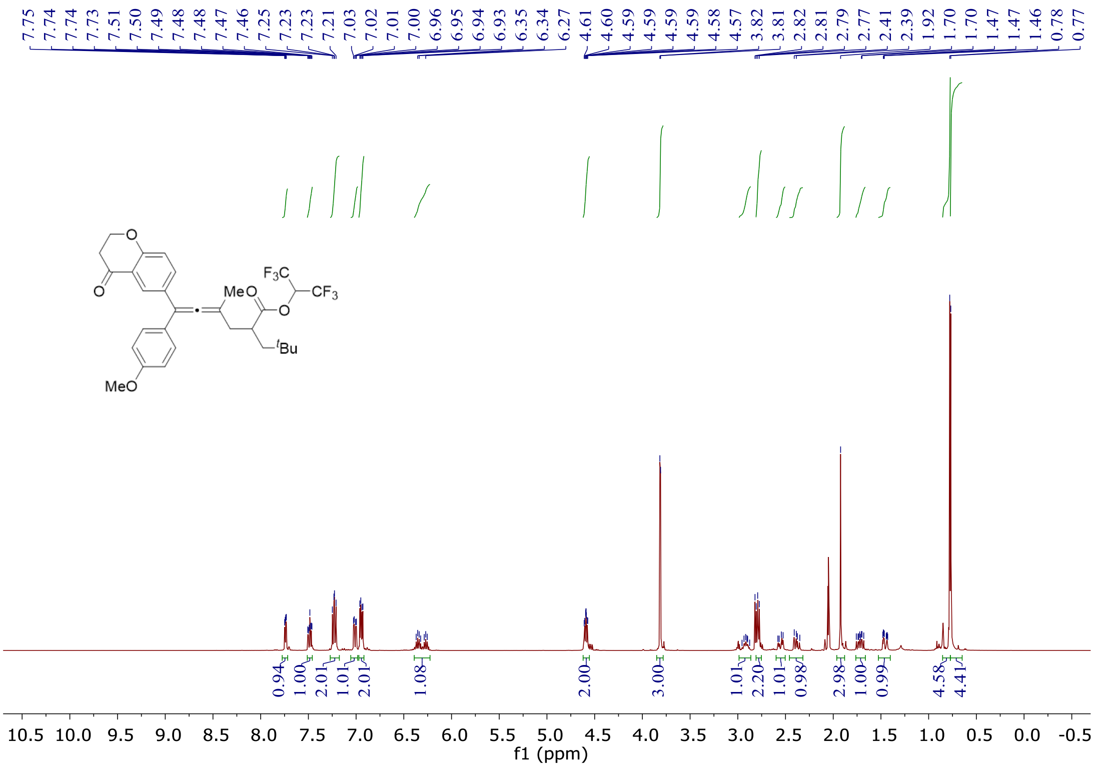

^{1}H NMR (400 MHz, acetone-d_6) of **37**

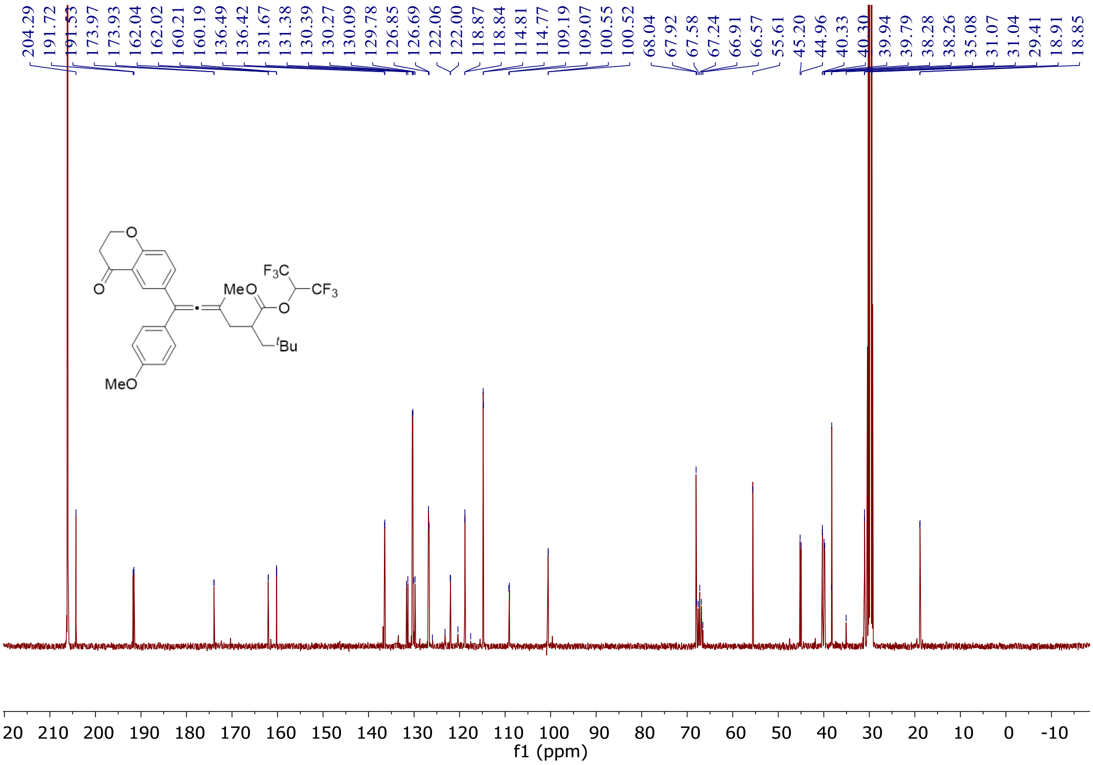

^{13}C{^{1}H} NMR (100 MHz, acetone-d_6) of **37**

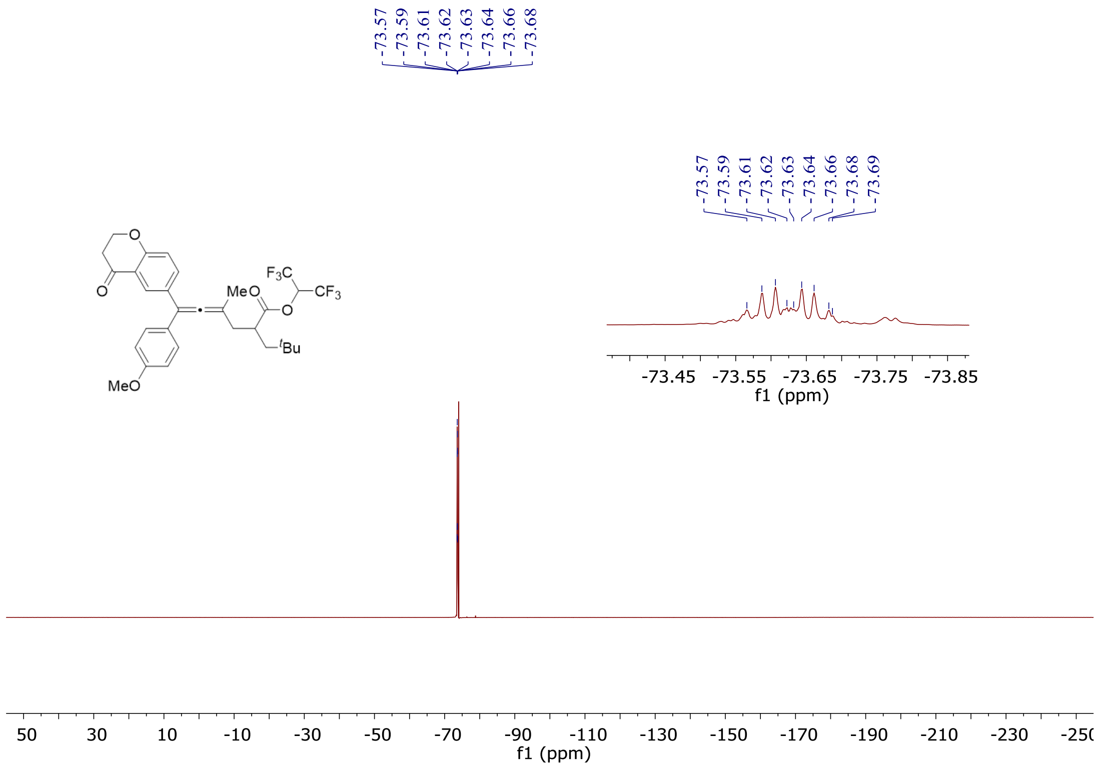

^{19}F{^{1}H} NMR (377 MHz, acetone-d_6) of **37**

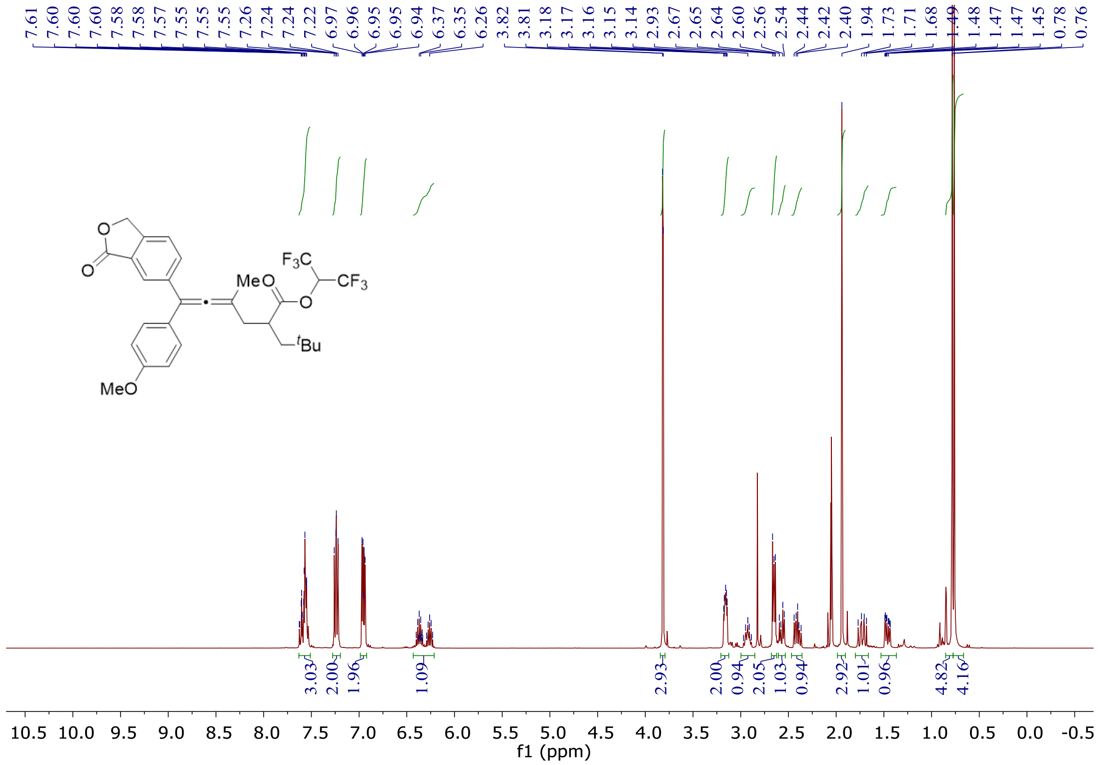

^{1}H NMR (400 MHz, acetone-d_6) of **38**

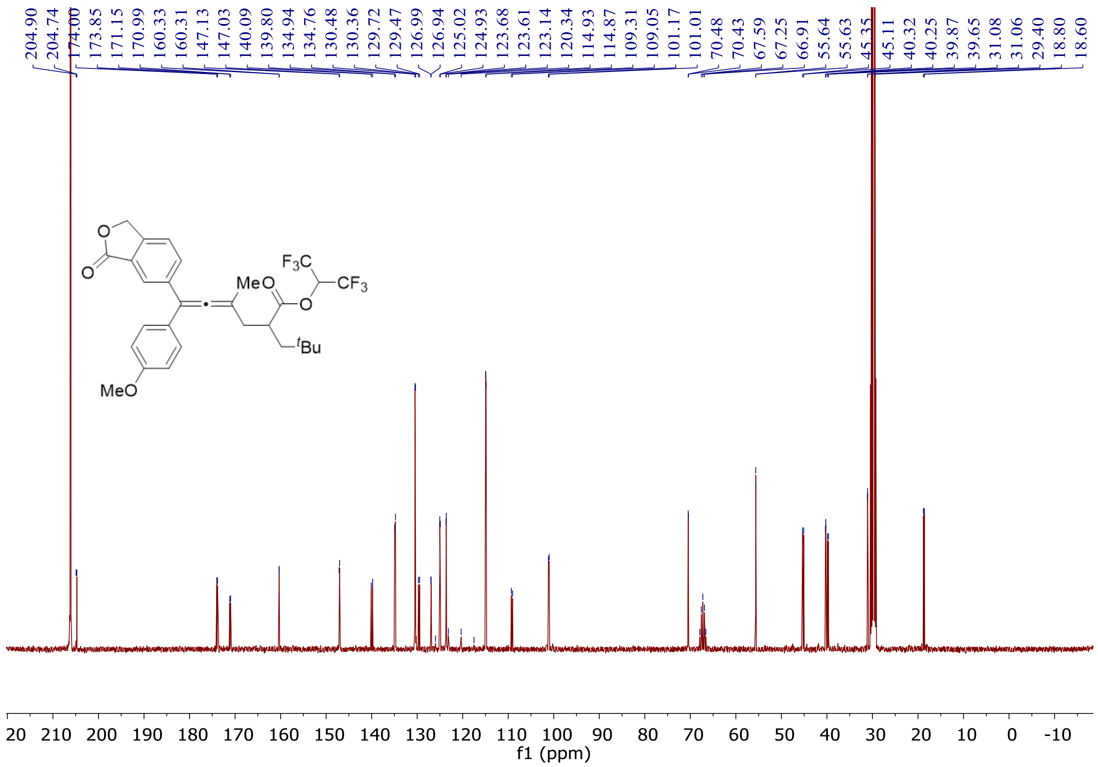

$^{13}C\{^{1}H\}$ NMR (100 MHz, acetone-d_6) of **38**

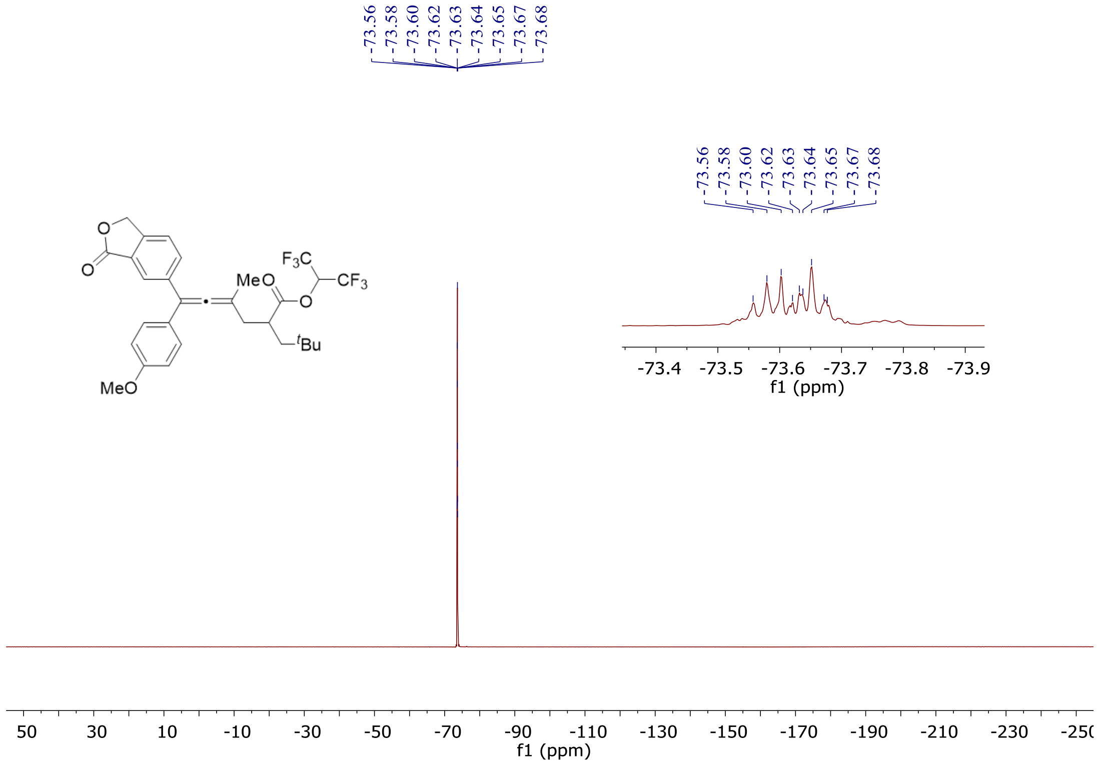

$^{19}F\{^{1}H\}$ NMR (377 MHz, acetone-d_6) of **38**

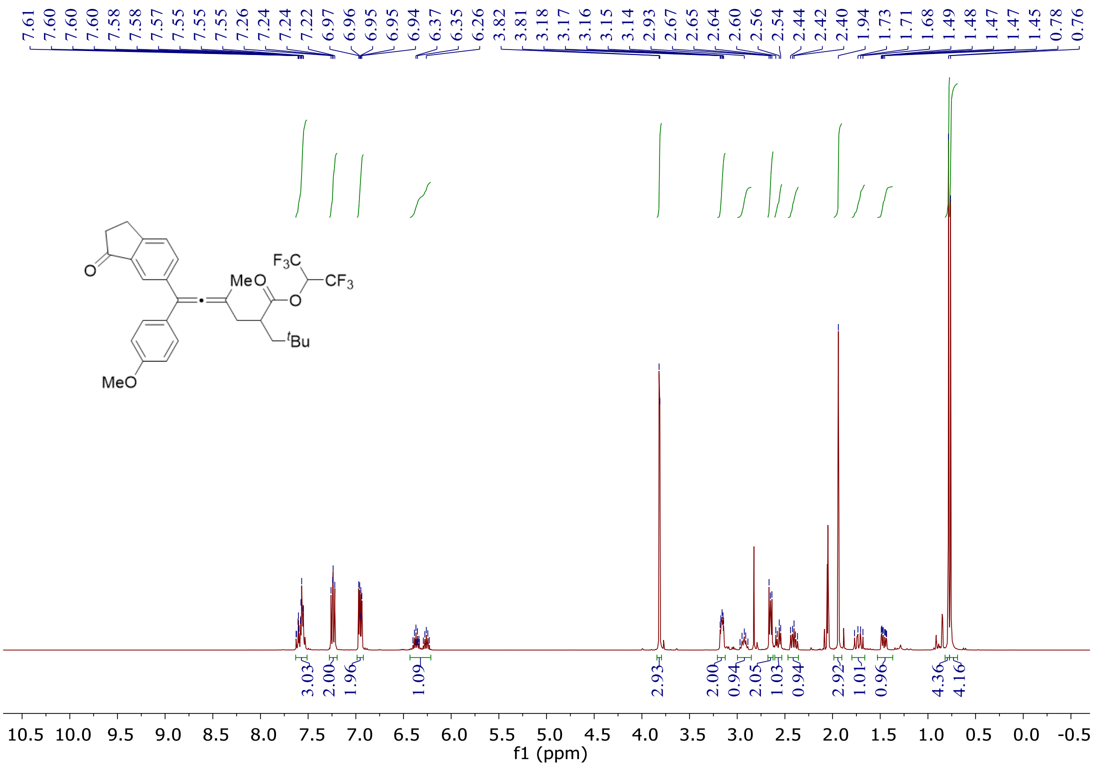

^{1}H NMR (400 MHz, acetone-d_6) of **39**

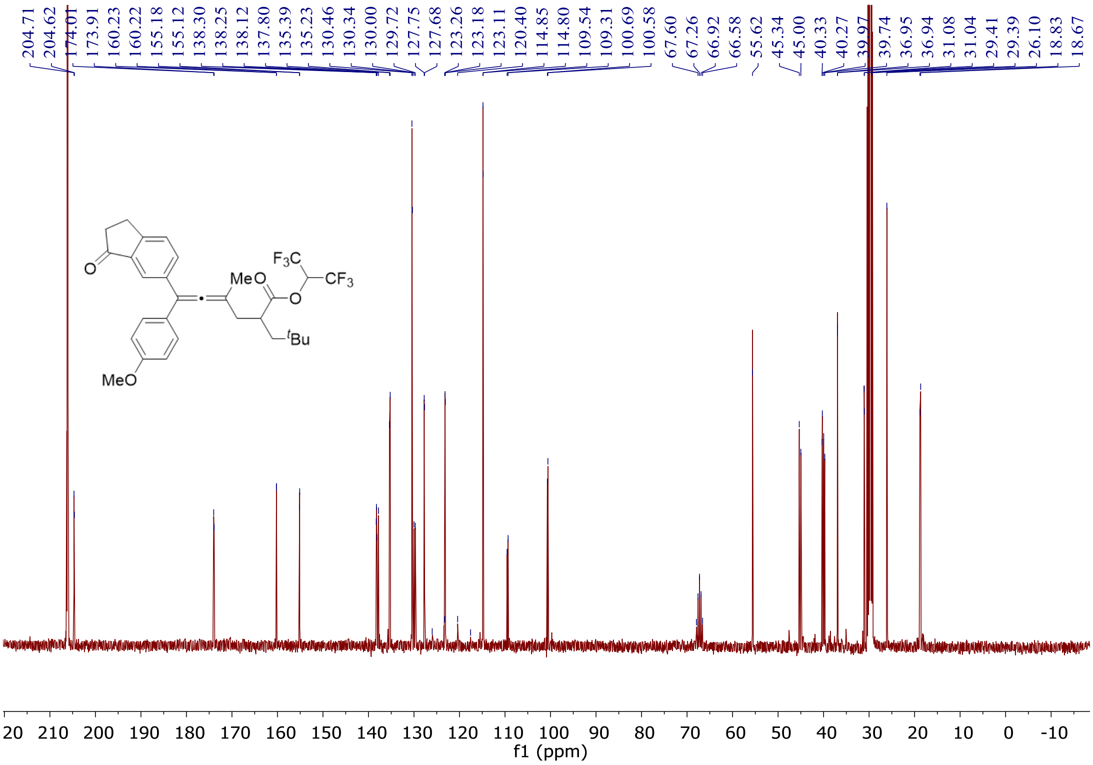

^{13}C{^{1}H} NMR (100 MHz, acetone-d_6) of **39**

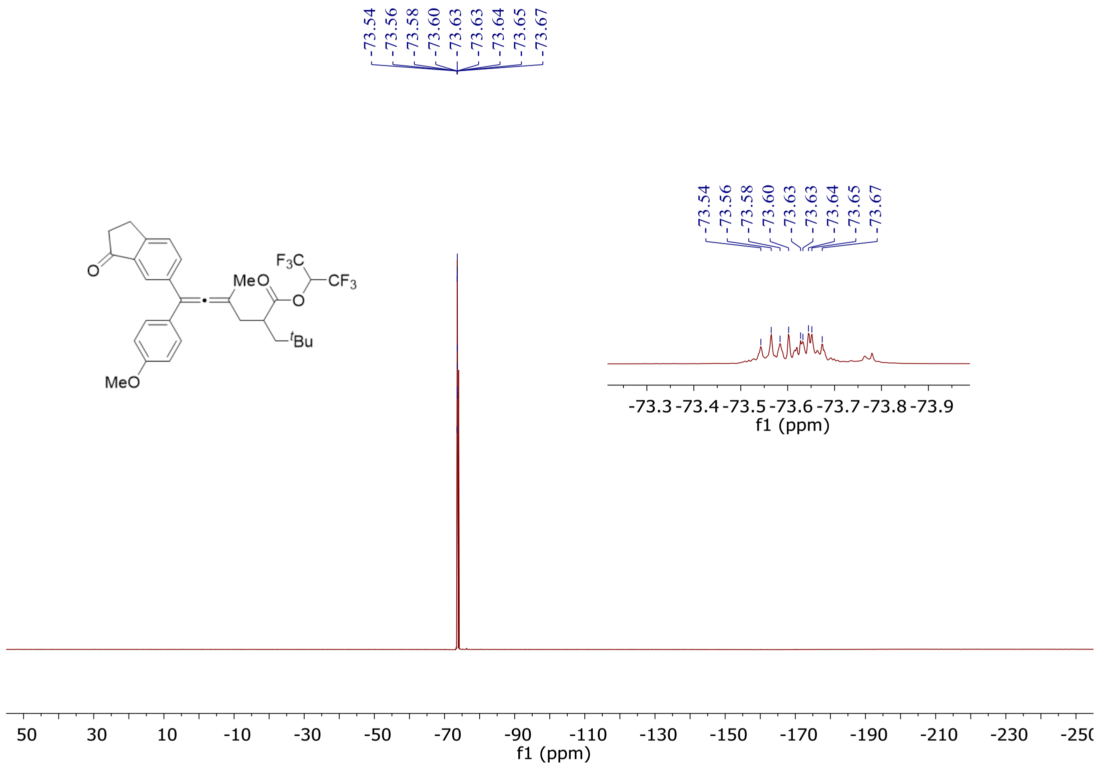

$^{19}F\{^{1}H\}$ NMR (377 MHz, acetone-d_6) of **39**

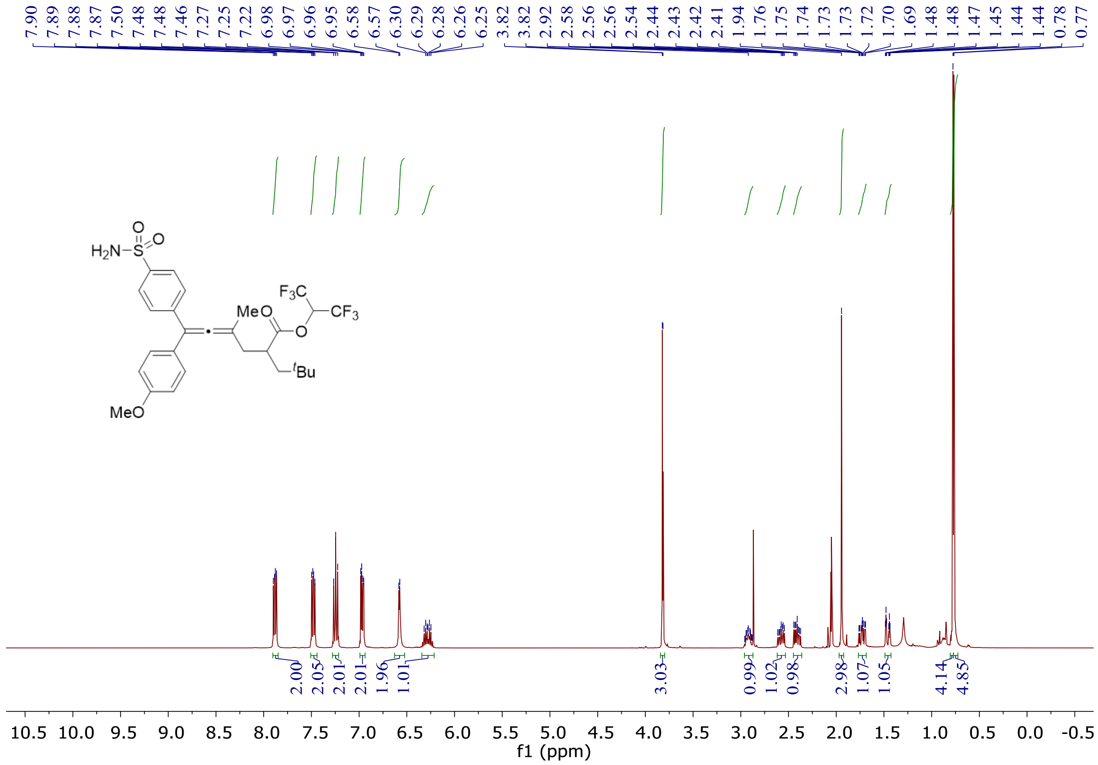

^{1}H NMR (400 MHz, acetone-d_6) of **40**

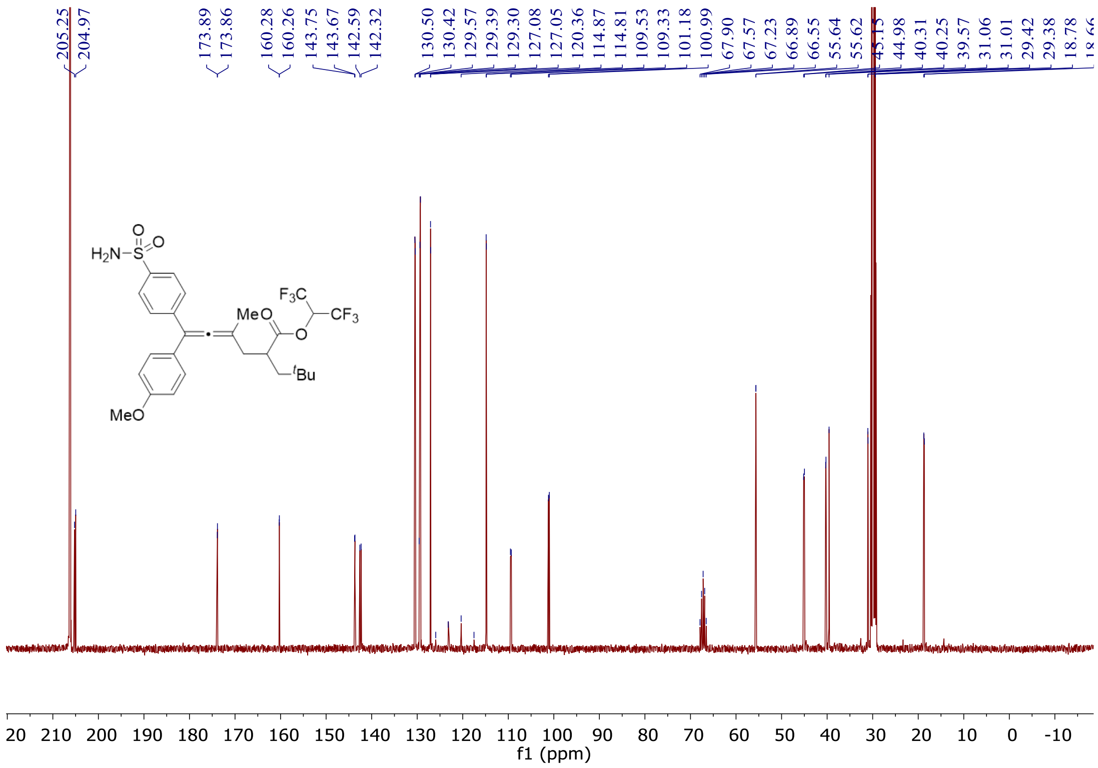

$^{13}C\{^{1}H\}$ **NMR** (100 MHz, acetone-d_6) of **40**

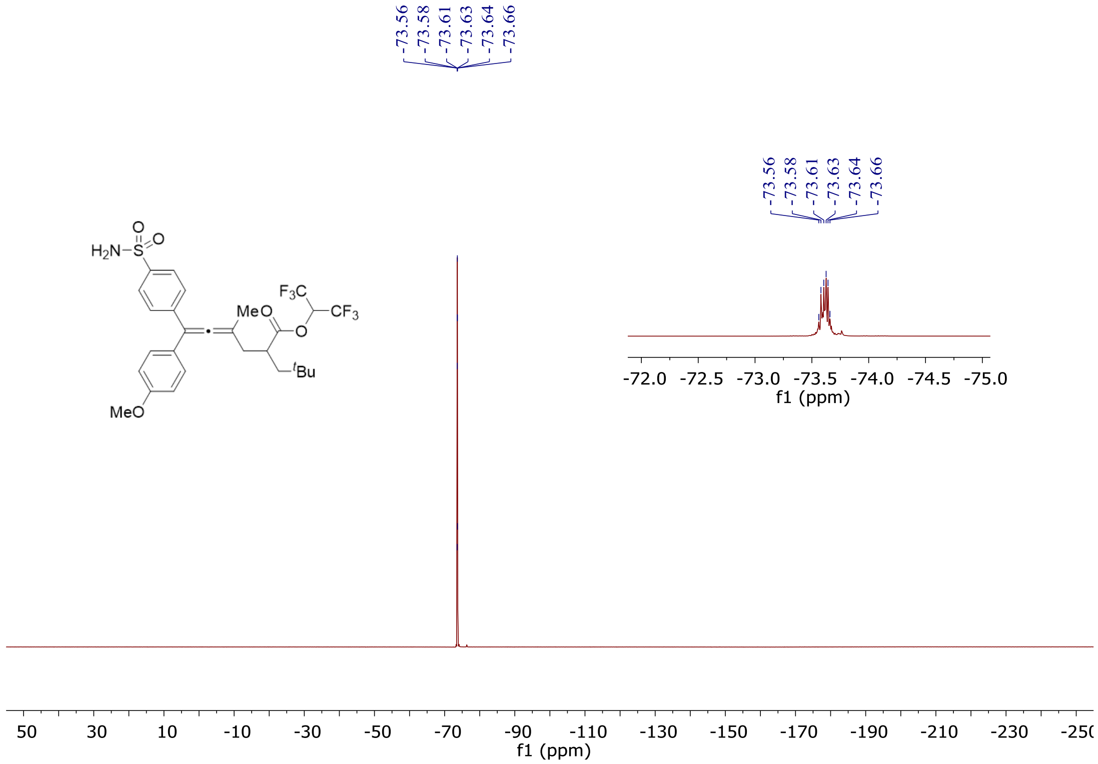

$^{19}F\{^{1}H\}$ **NMR** (377 MHz, acetone-d_6) of **40**

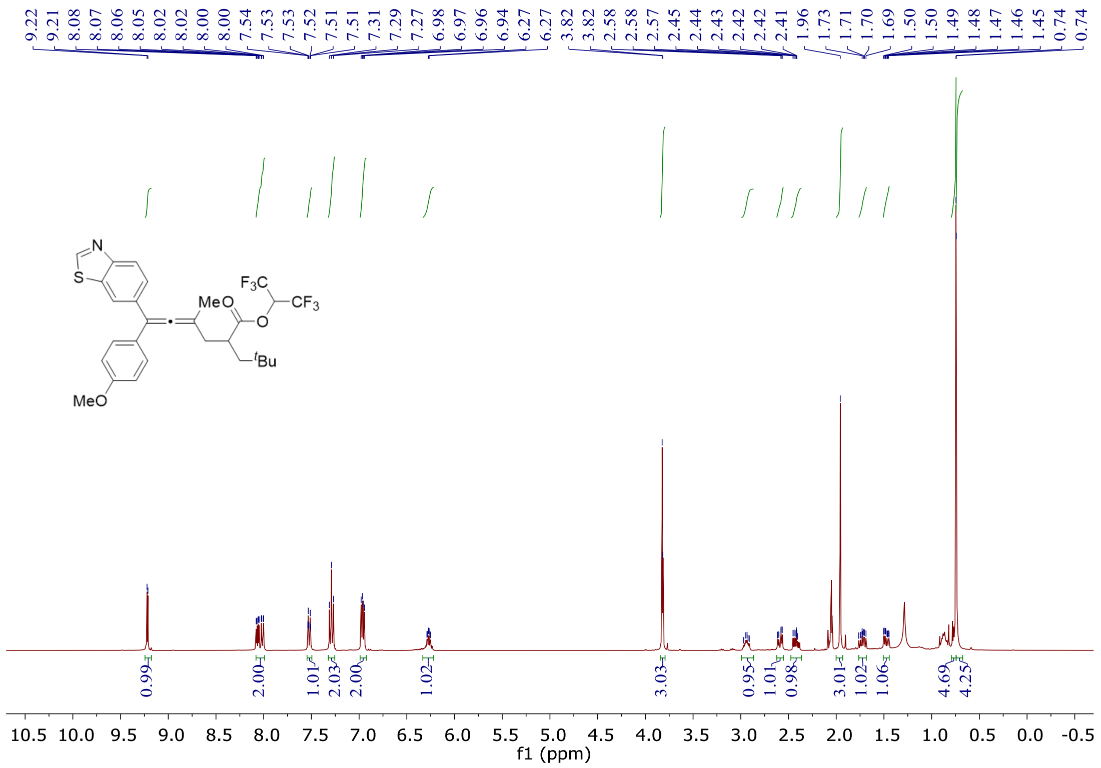

^{1}**H NMR** (400 MHz, acetone-d_6) of **41**

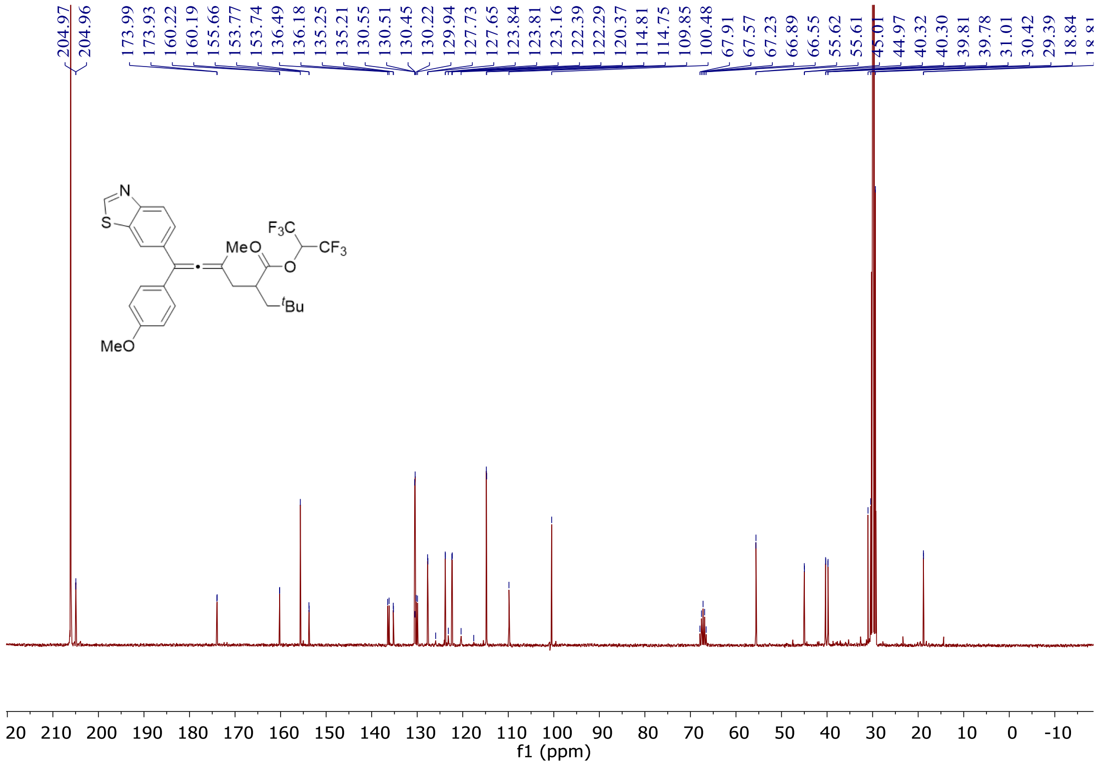

^{13}**C{^{1}H} NMR** (100 MHz, acetone-d_6) of **41**

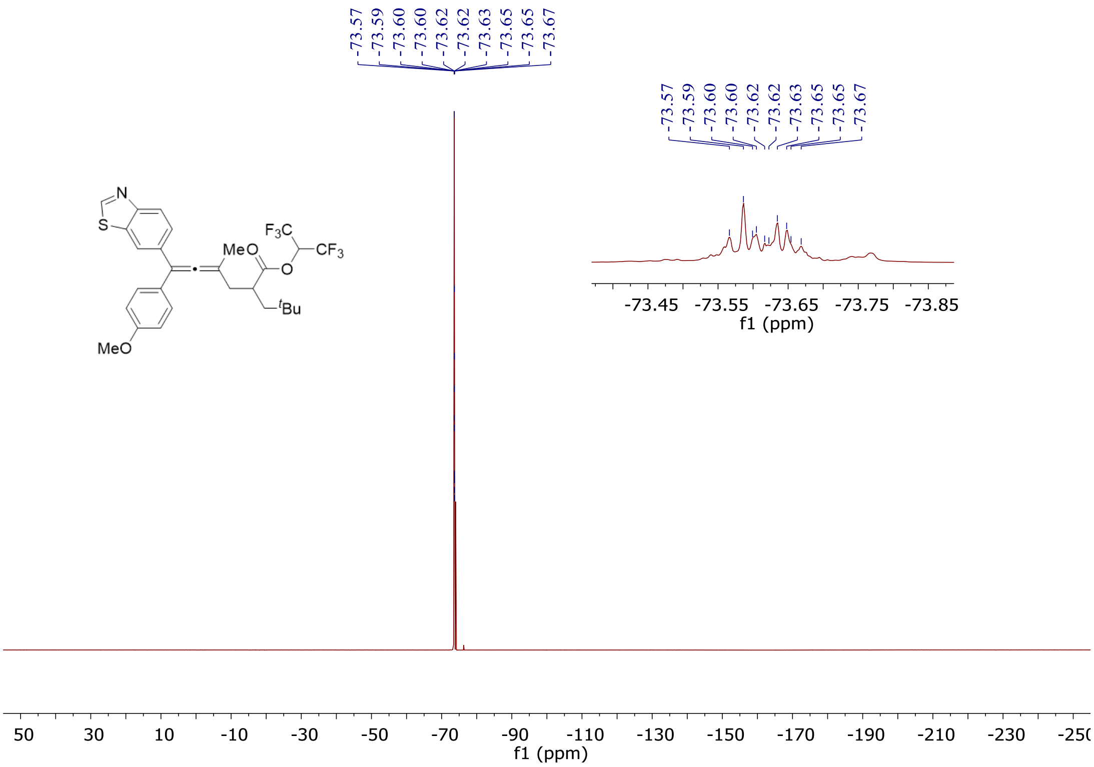

$^{19}F\{^{1}H\}$ **NMR** (377 MHz, acetone-d_6) of **41**

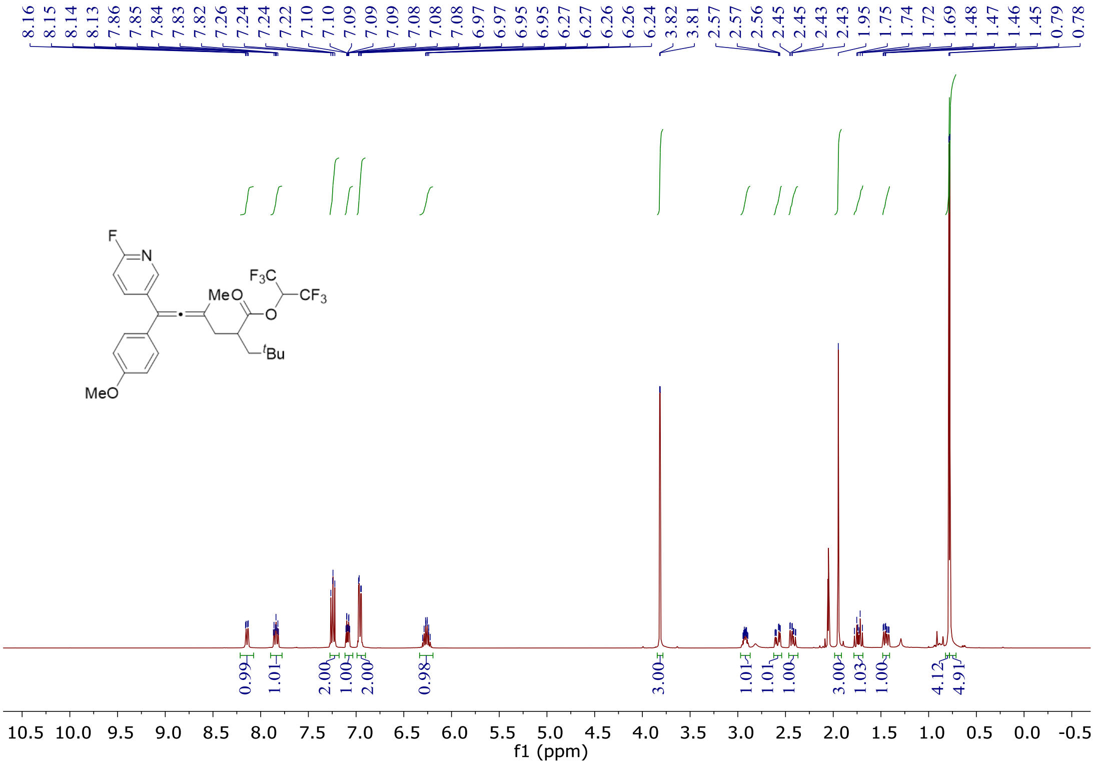

^{1}H **NMR** (400 MHz, acetone-d_6) of **42**

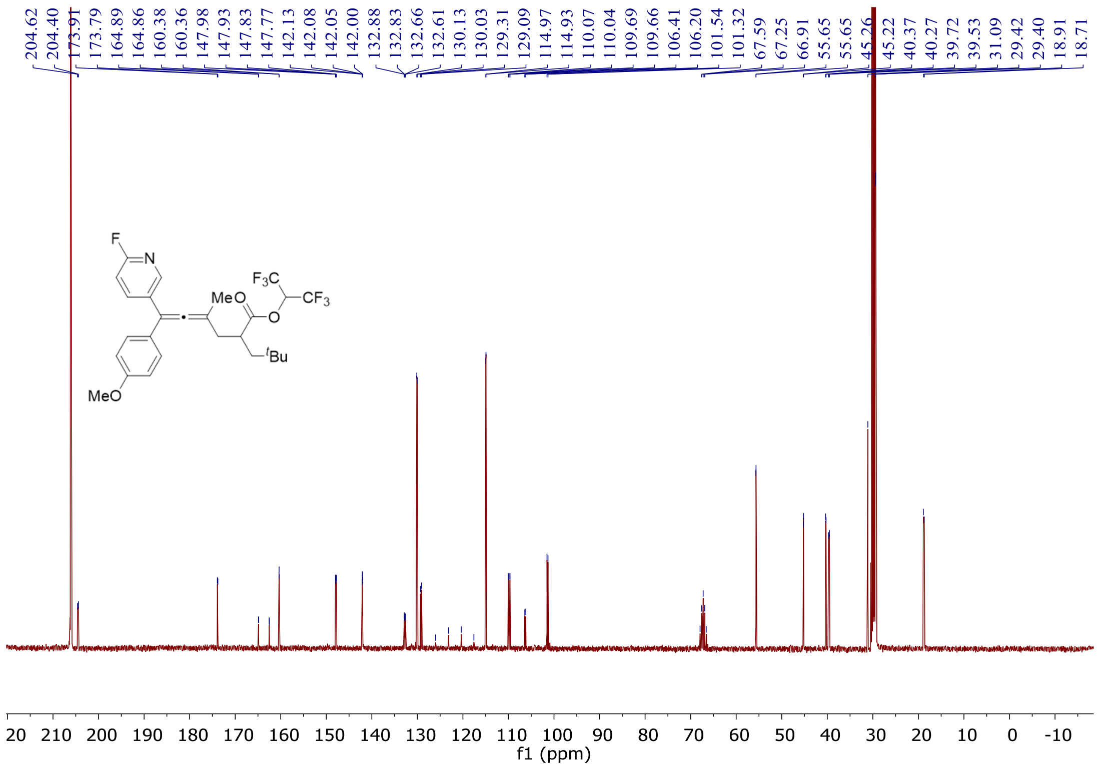

^{13}C{^{1}H} NMR (100 MHz, acetone-d_6) of **42**

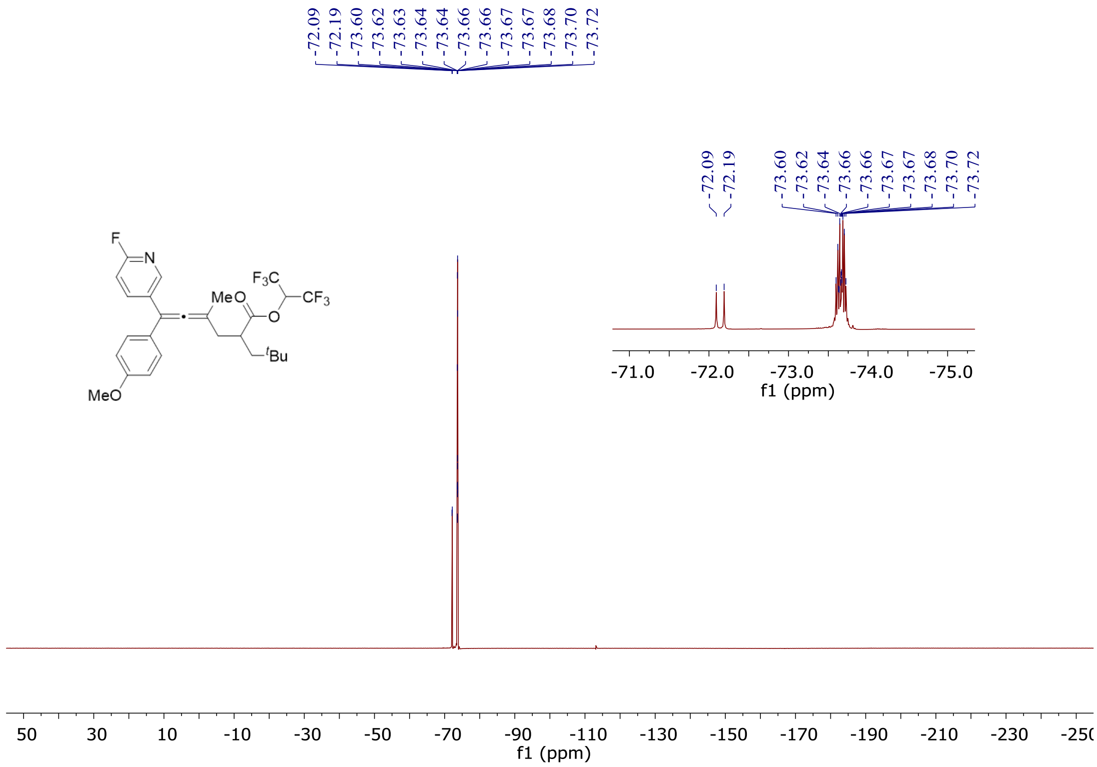

^{19}F{^{1}H} NMR (377 MHz, acetone-d_6) of **42**

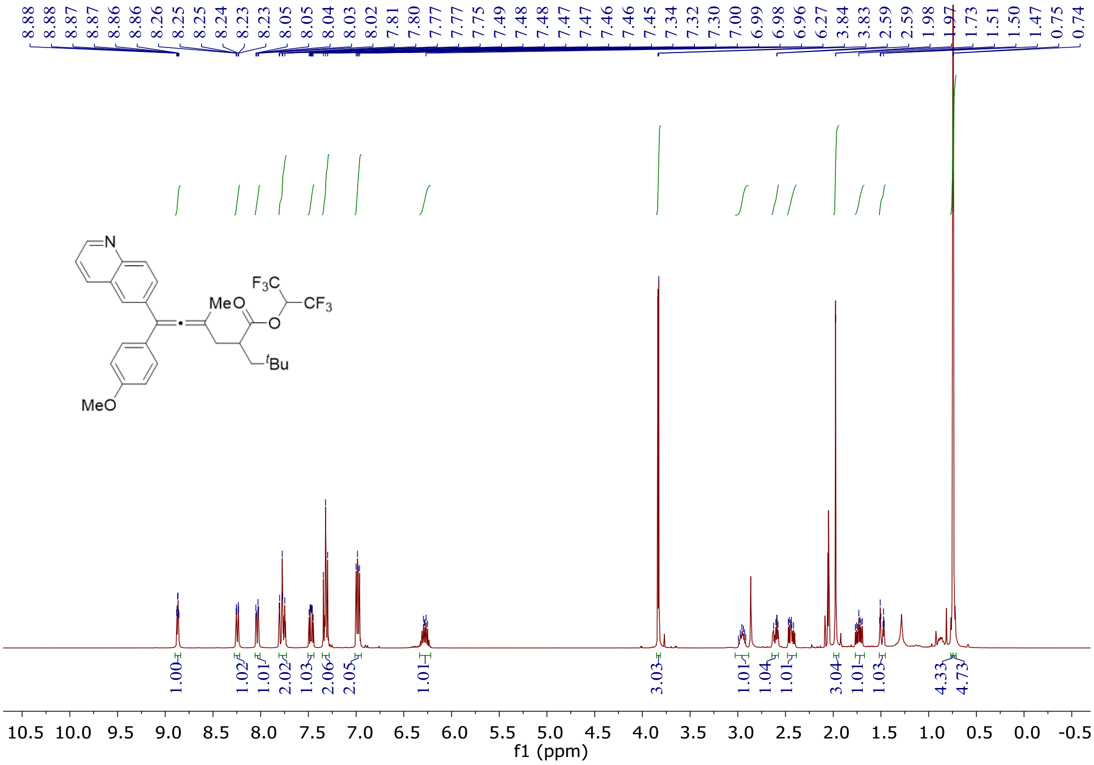

^{1}H NMR (400 MHz, acetone-d_6) of **43**

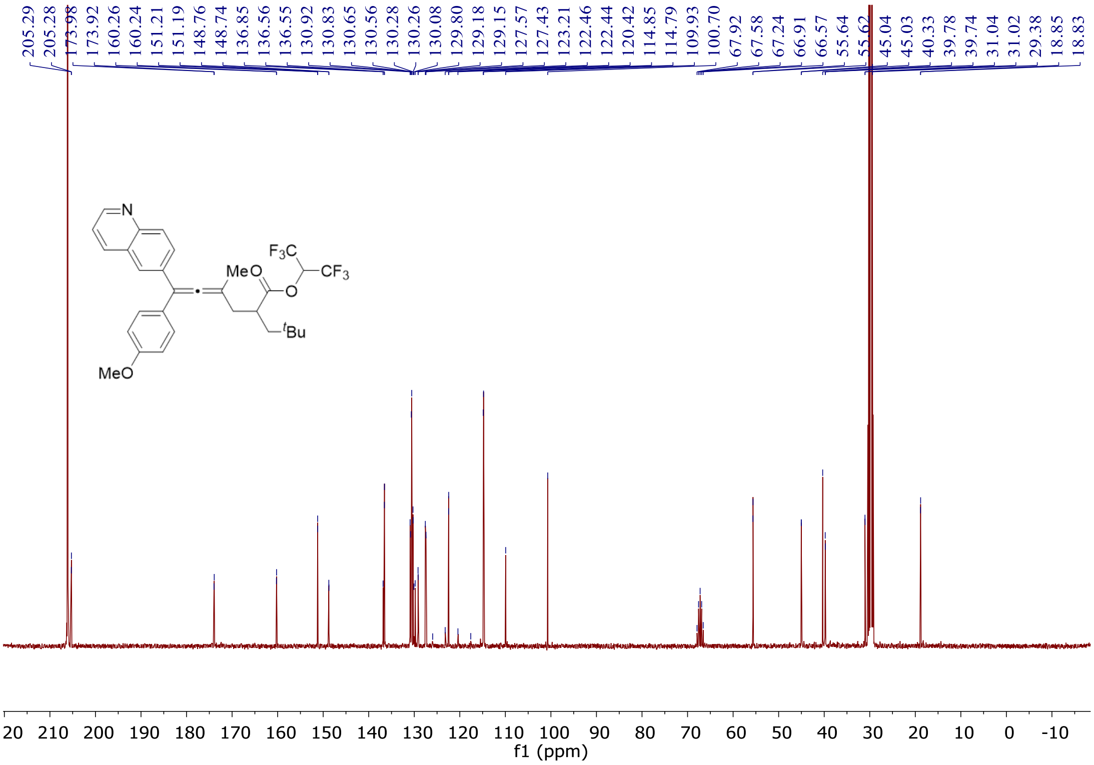

^{13}C{^{1}H} NMR (100 MHz, acetone-d_6) of **43**

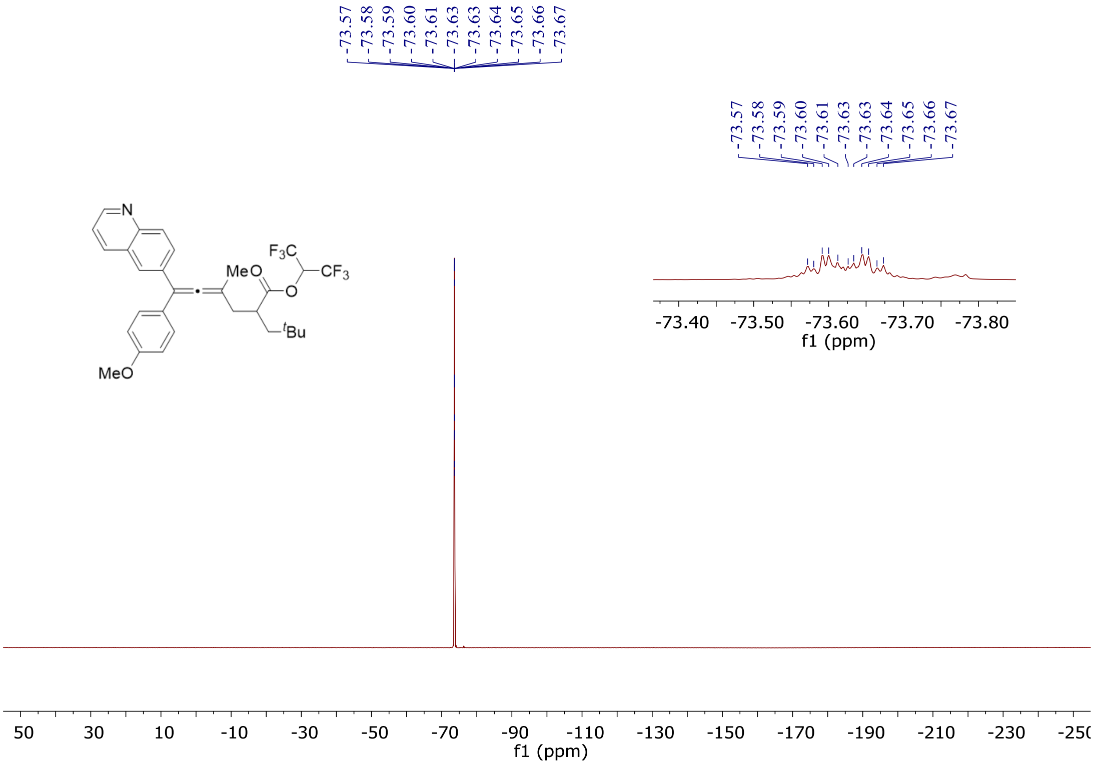

^{19}F{^{1}H} NMR (377 MHz, acetone-d_6) of **43**

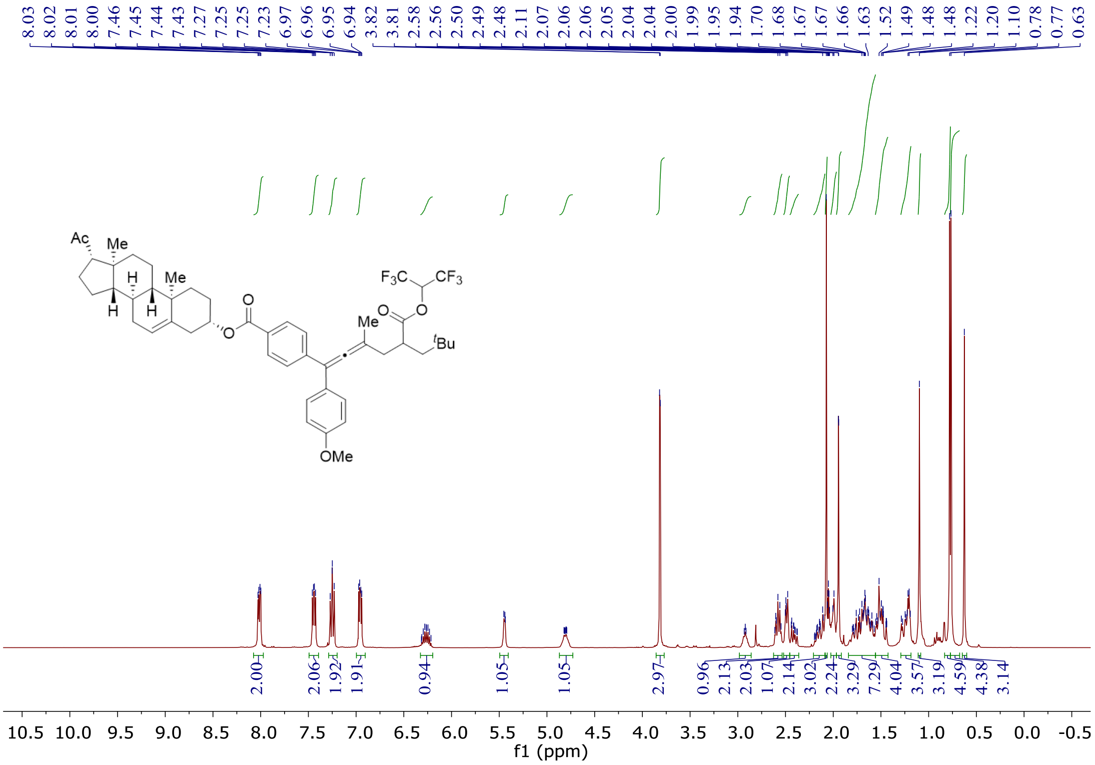

^{1}H NMR (400 MHz, acetone-d_6) of **44**

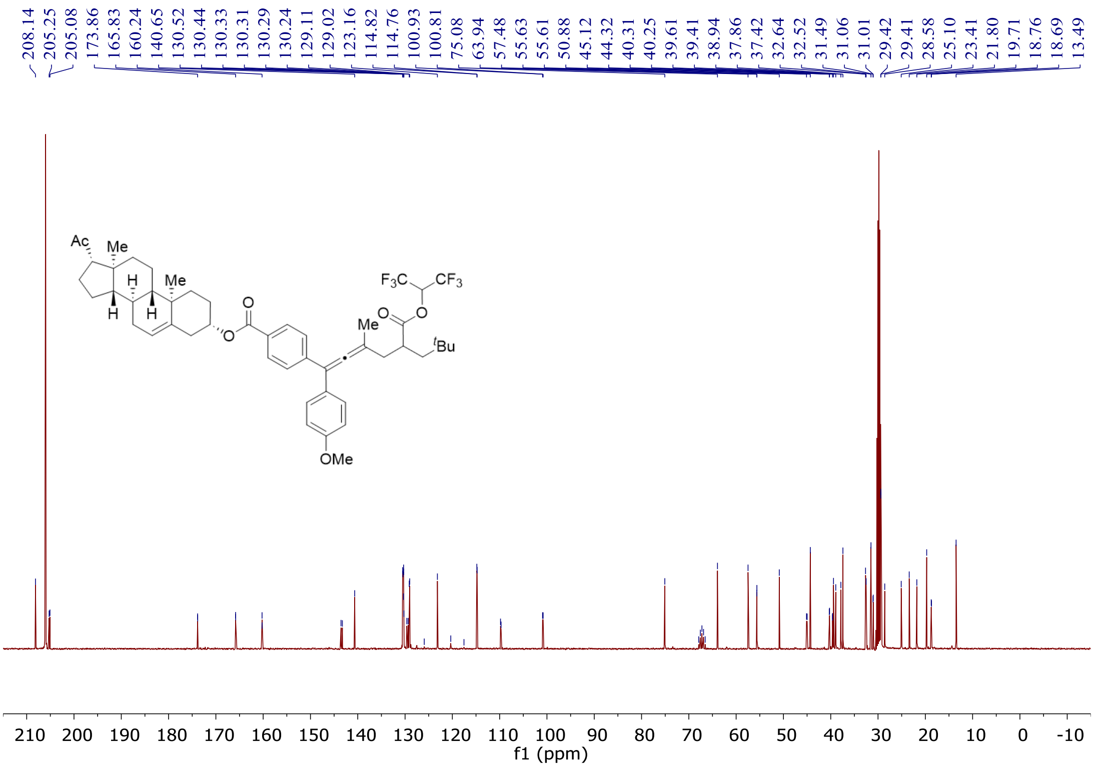

$^{13}C\{^1H\}$ NMR (100 MHz, acetone-d_6) of **44**

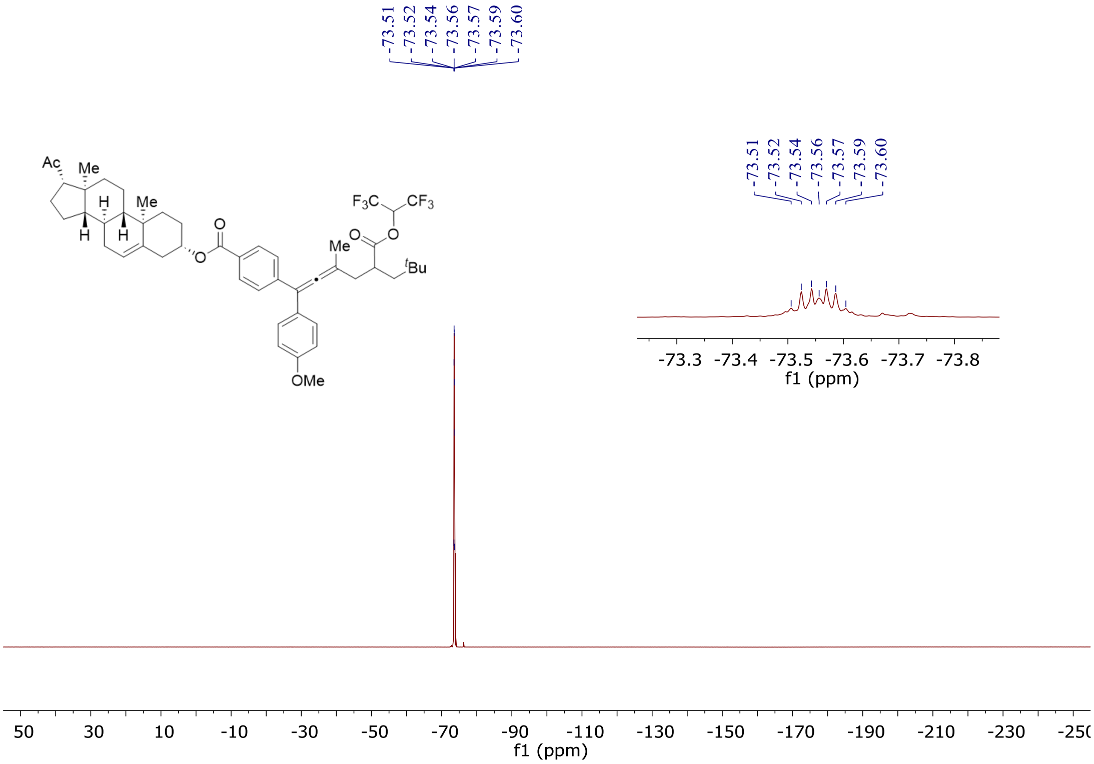

$^{19}F\{^1H\}$ NMR (377 MHz, acetone-d_6) of **44**

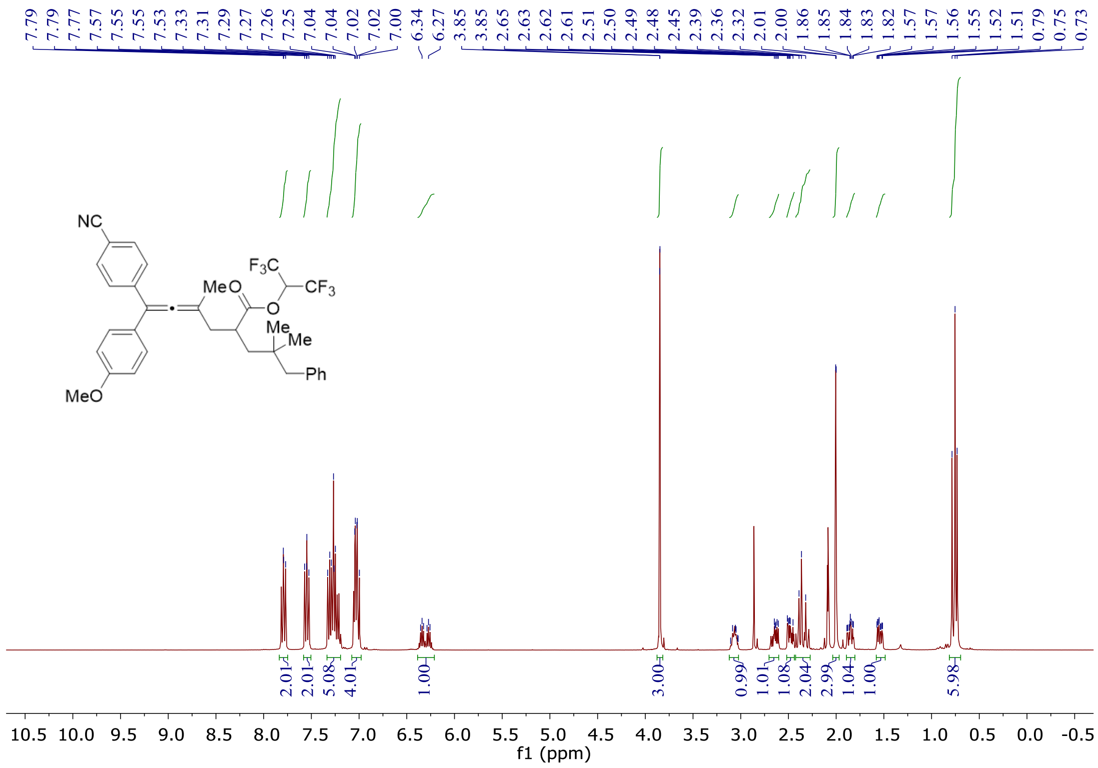

^{1}**H NMR** (400 MHz, acetone-d_6) of **45**

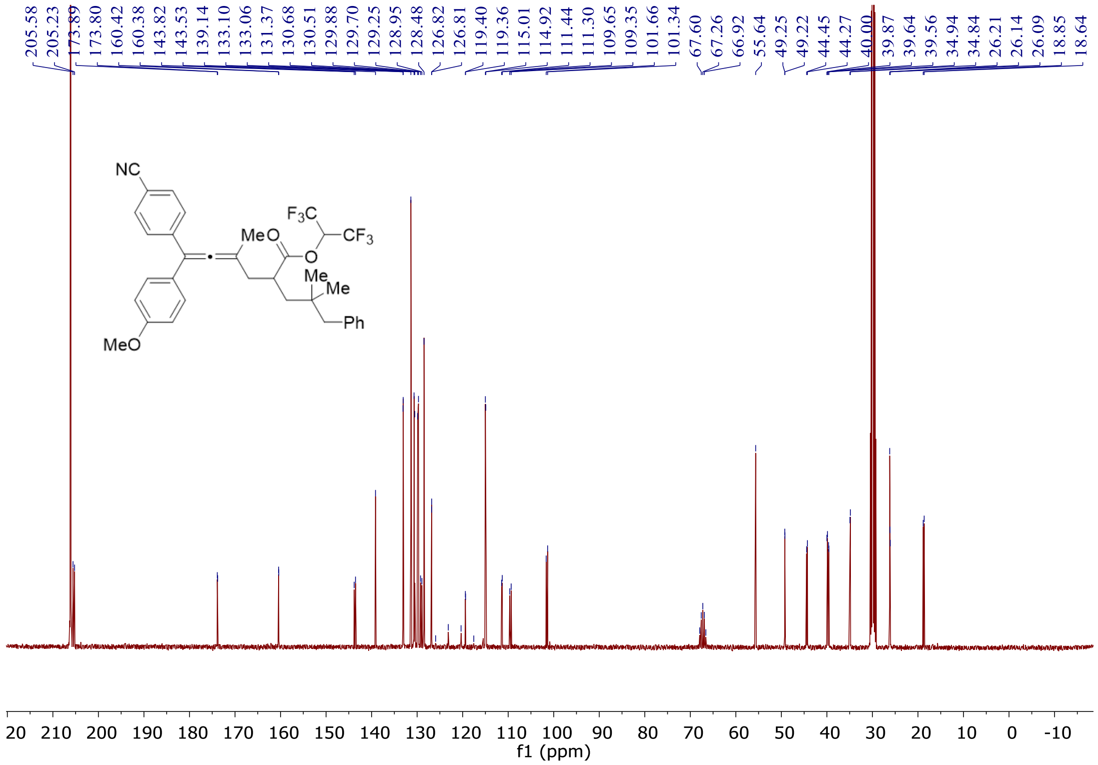

^{13}**C{^{1}H} NMR** (100 MHz, acetone-d_6) of **45**

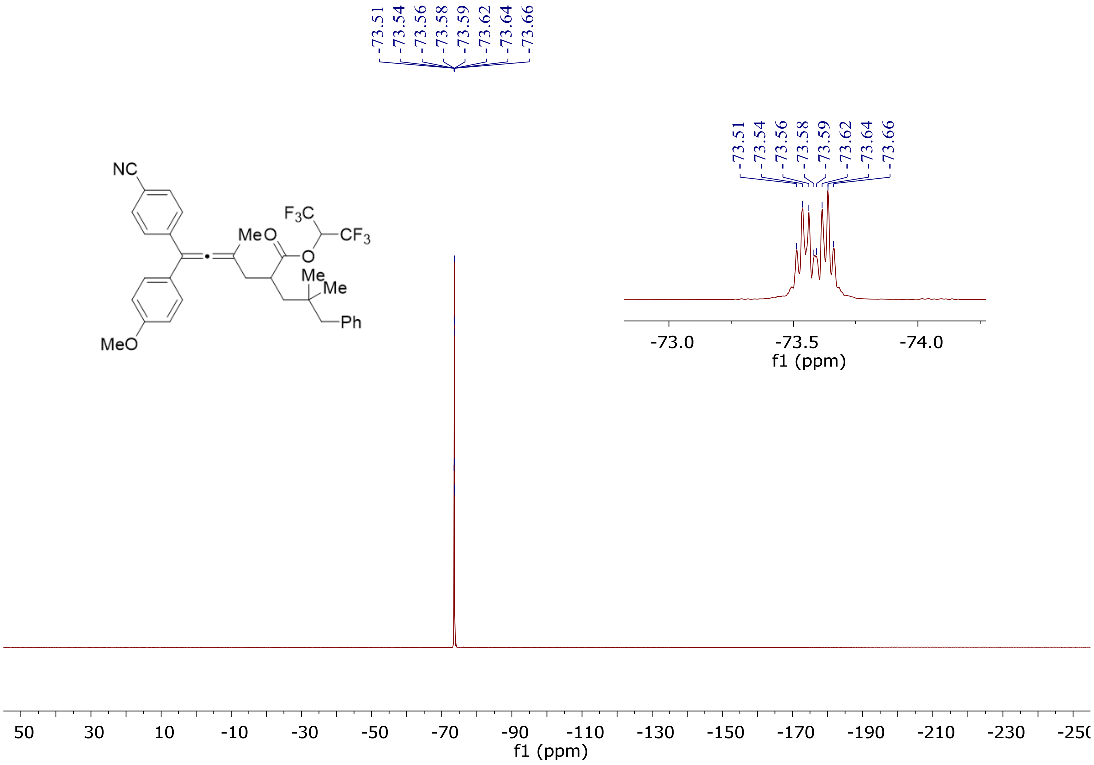

$^{19}F\{^1H\}$ NMR (377 MHz, acetone-d_6) of **45**

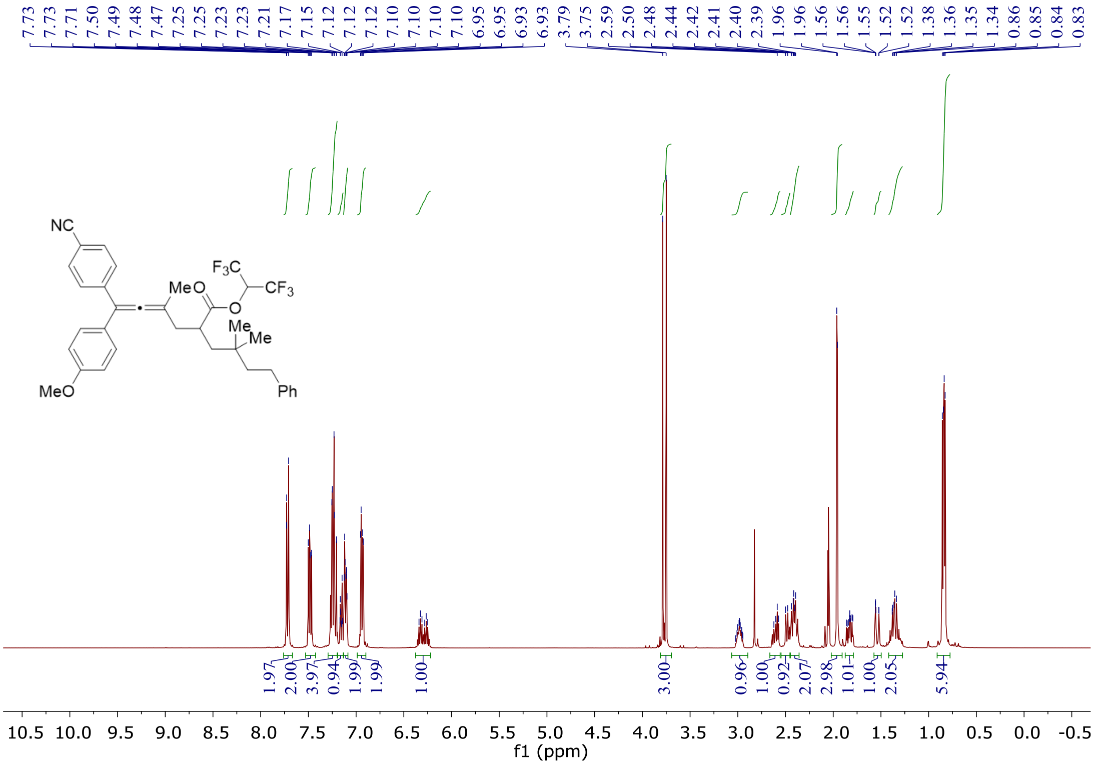

^1H NMR (400 MHz, acetone-d_6) of **46**

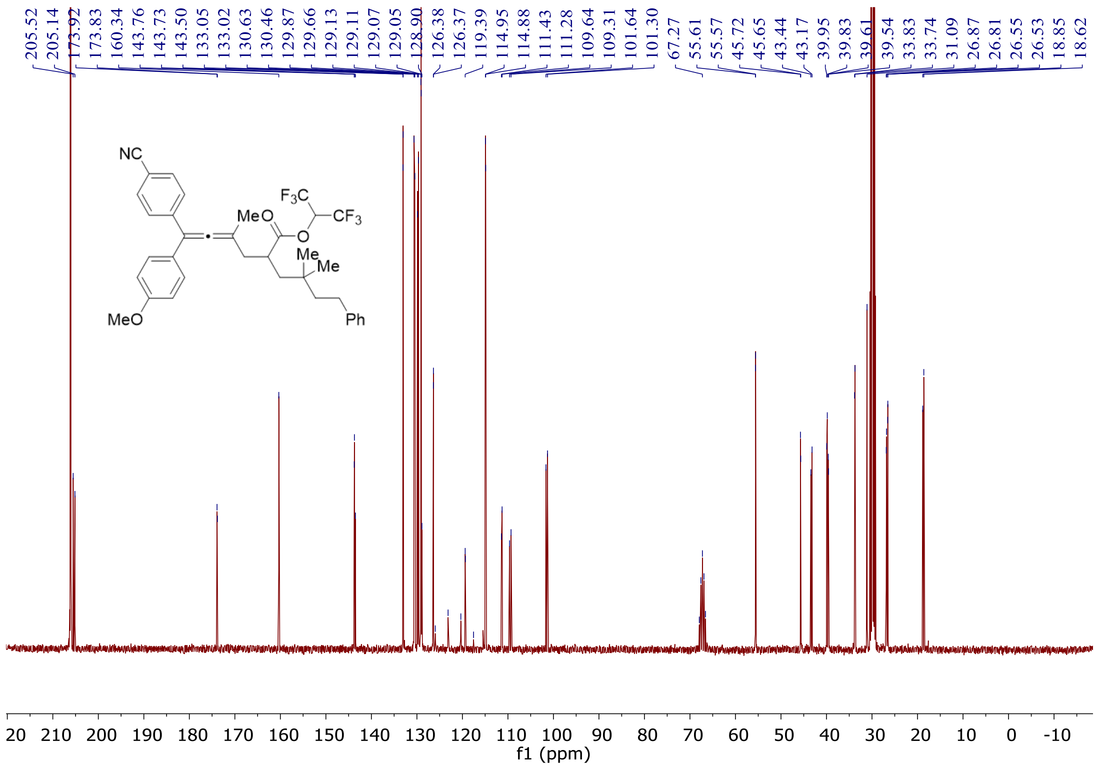

^{13}**C{^{1}H} NMR** (100 MHz, acetone-d_6) of **46**

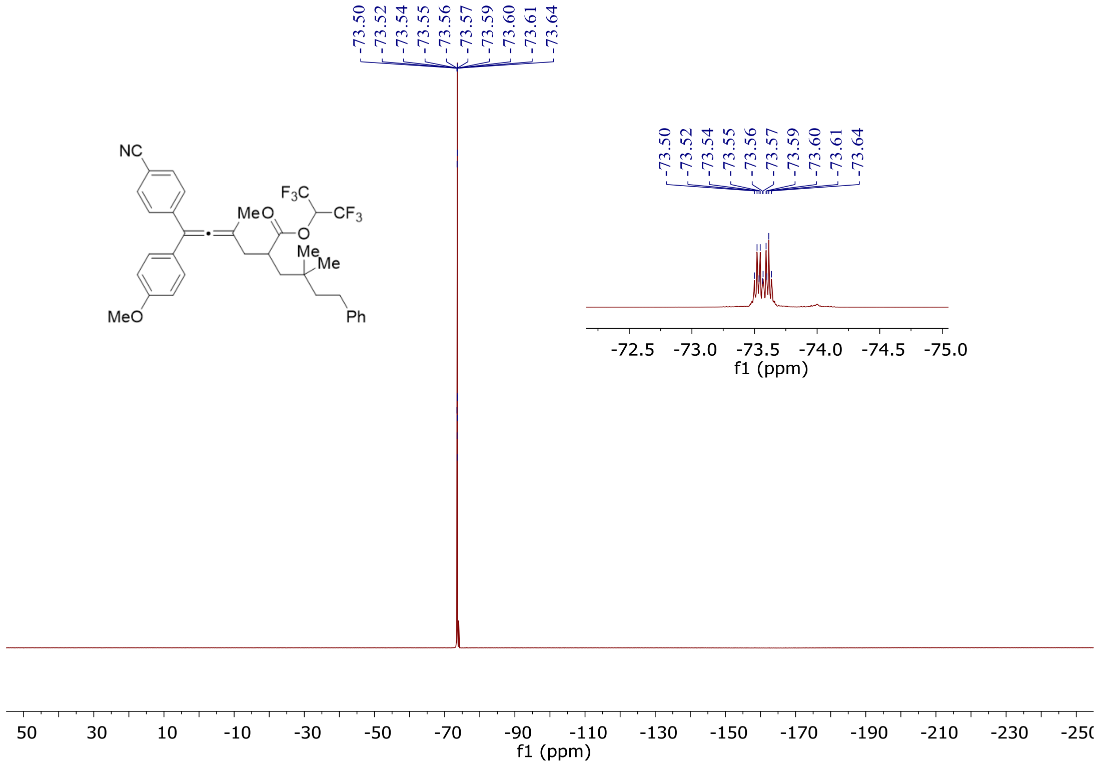

^{19}**F{^{1}H} NMR** (377 MHz, acetone-d_6) of **46**

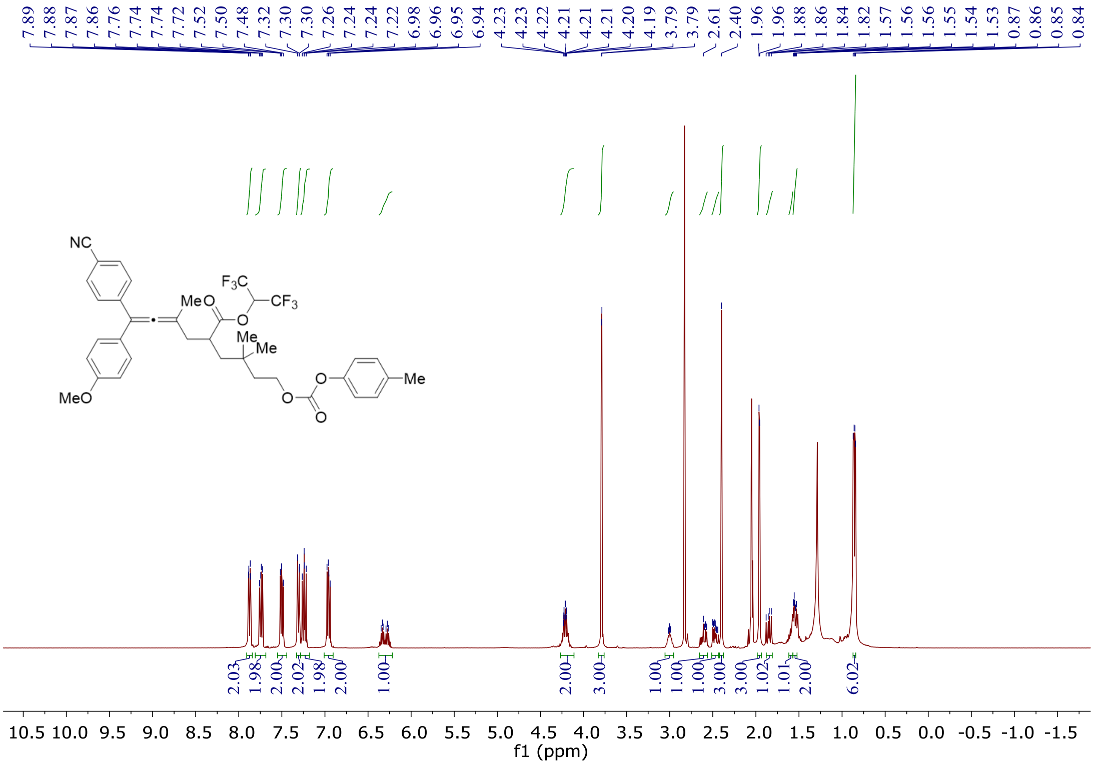

¹H NMR (400 MHz, acetone-d_6) of **47**

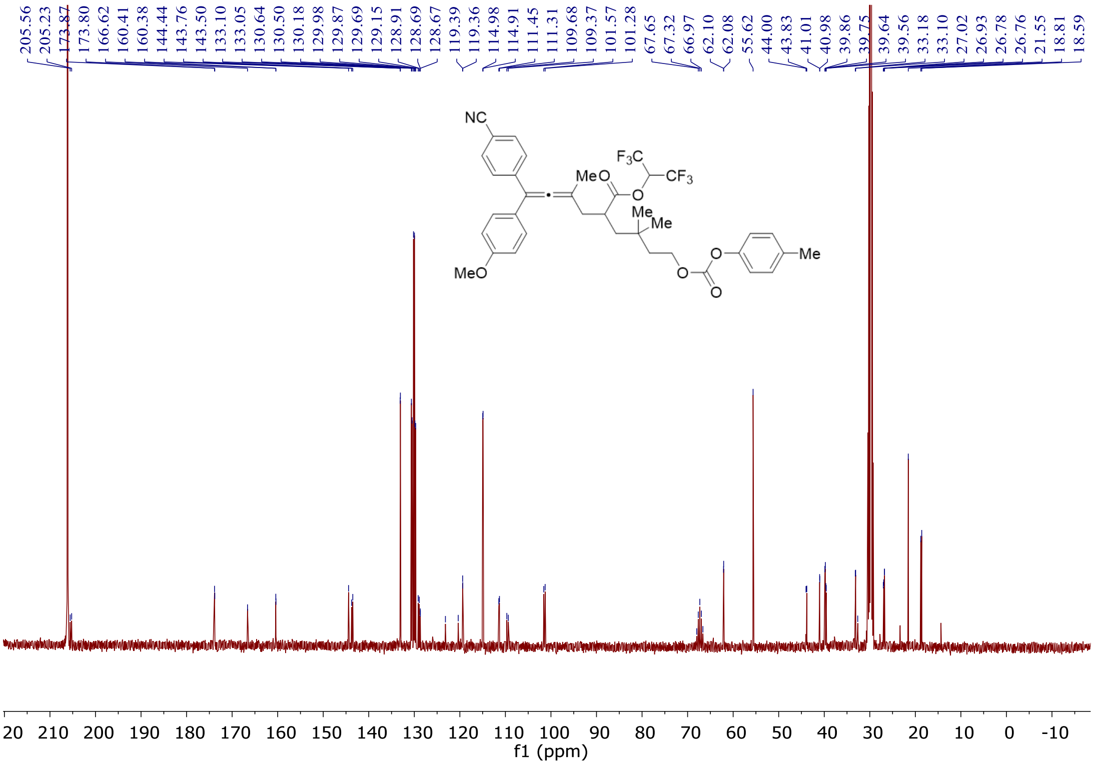

^{13}C{^{1}H} NMR (100 MHz, acetone-d_6) of **47**

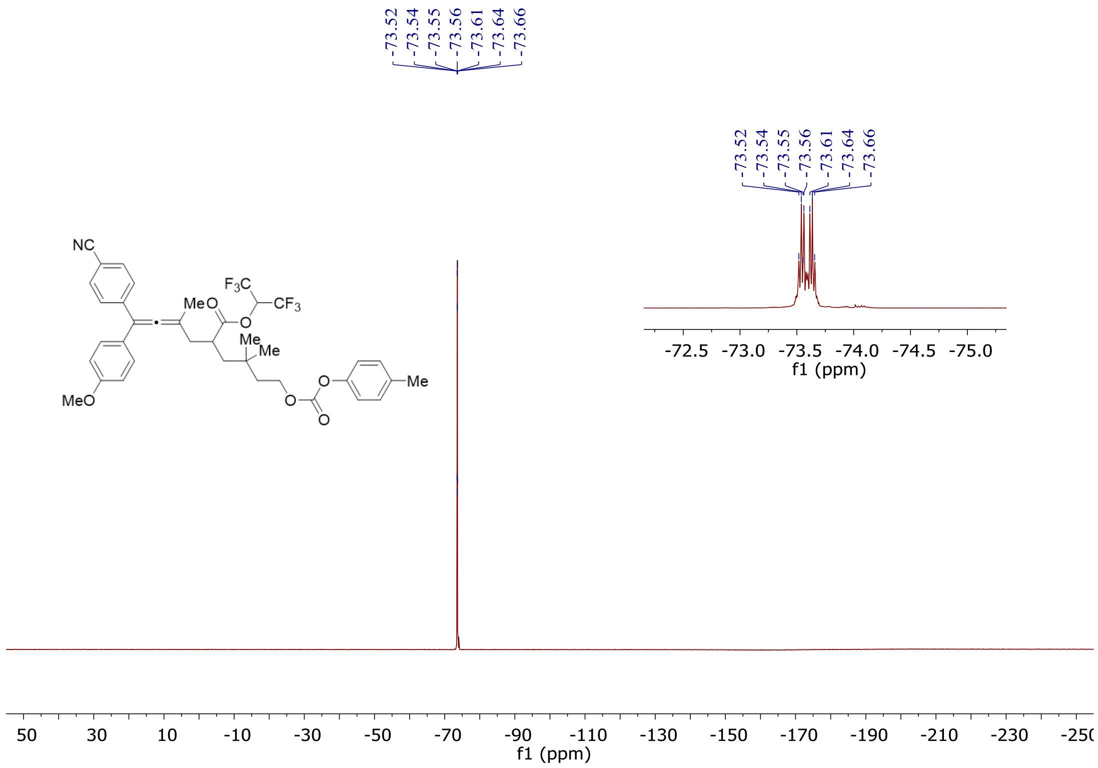

$^{19}F\{^{1}H\}$ NMR (377 MHz, acetone-d_6) of **47**

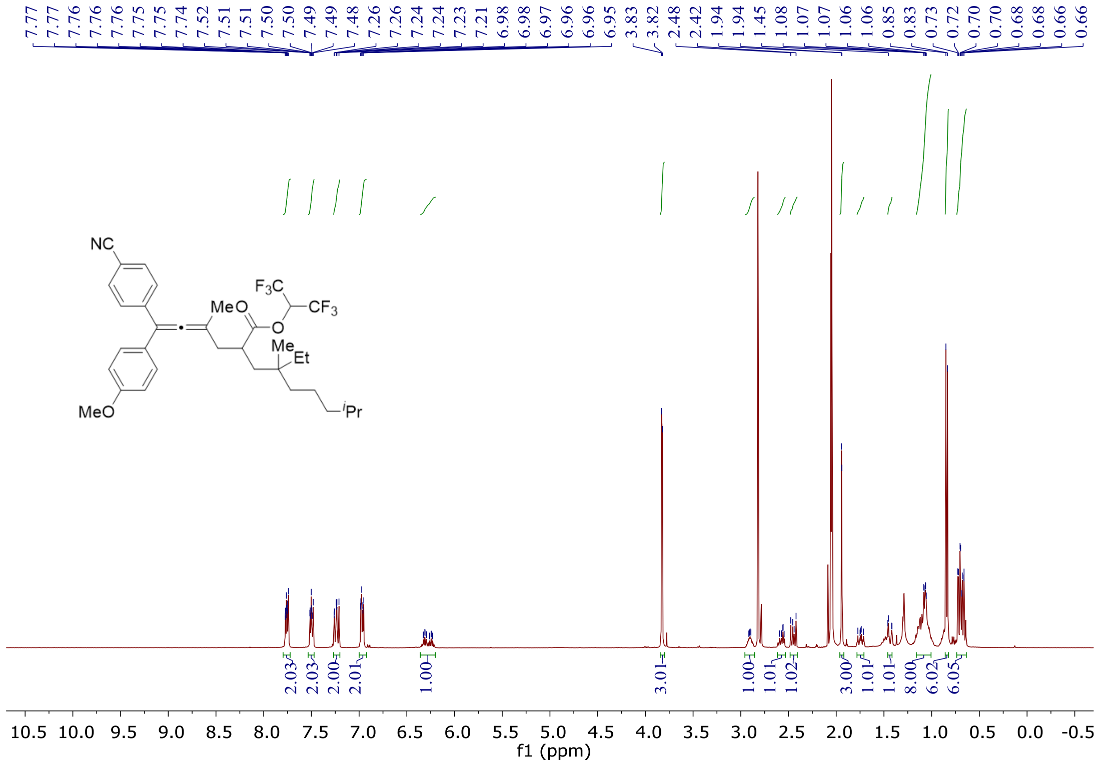

^{1}H NMR (400 MHz, acetone-d_6) of **48**

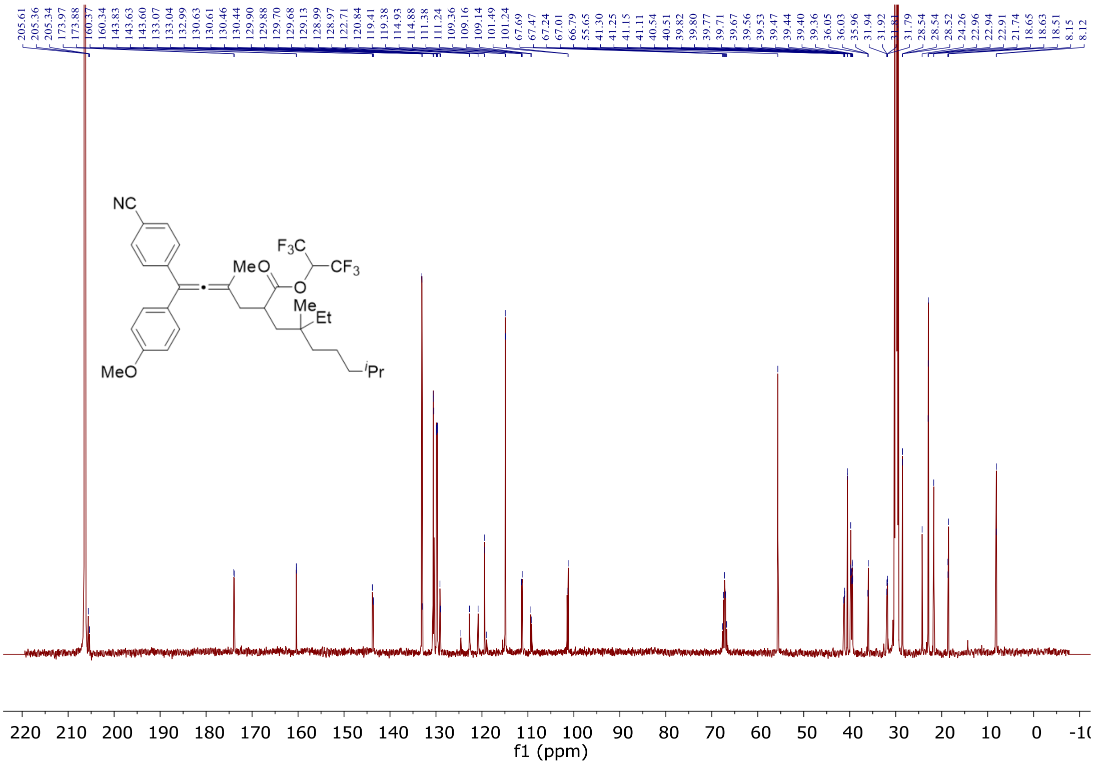

$^{13}C\{^1H\}$ NMR (100 MHz, acetone-d_6) of **48**

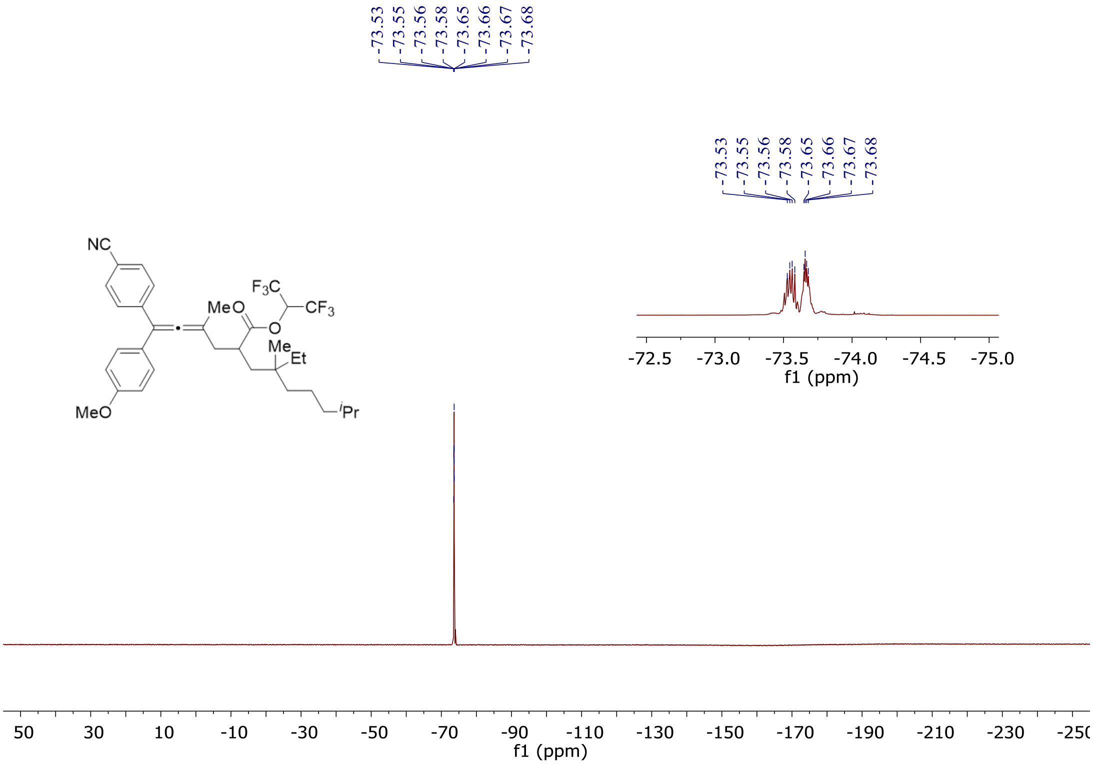

$^{19}F\{^1H\}$ NMR (377 MHz, acetone-d_6) of **48**

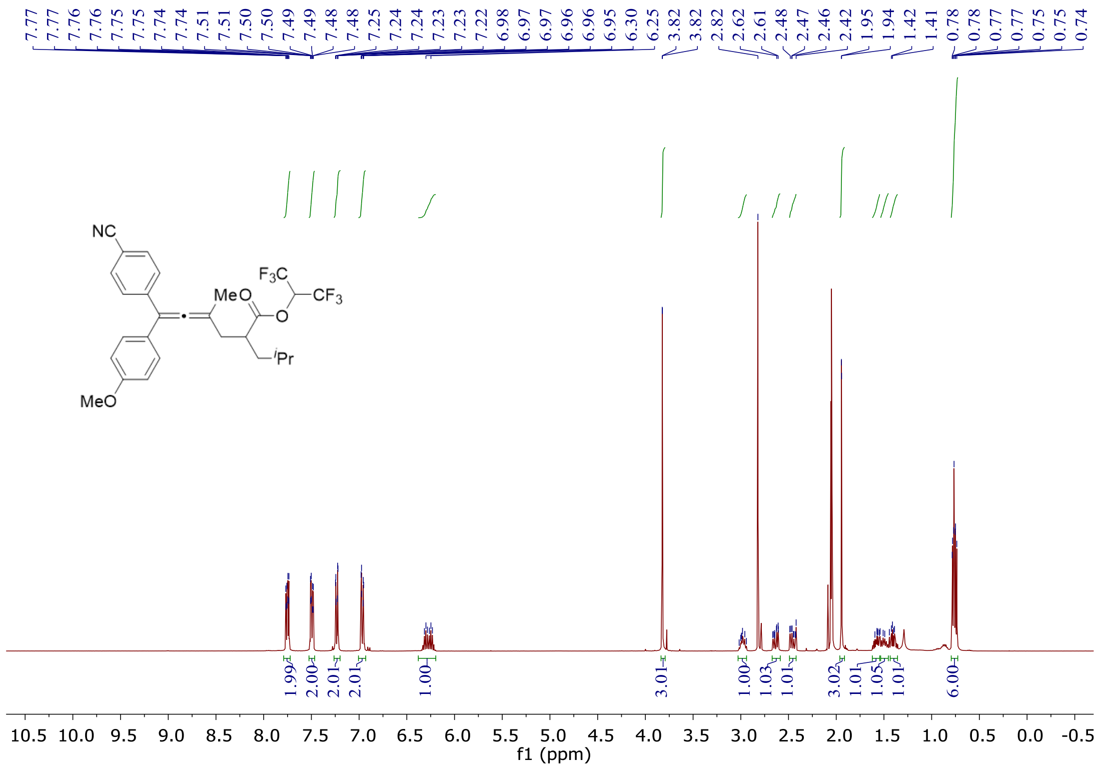

^{1}H NMR (400 MHz, acetone-d_6) of **49**

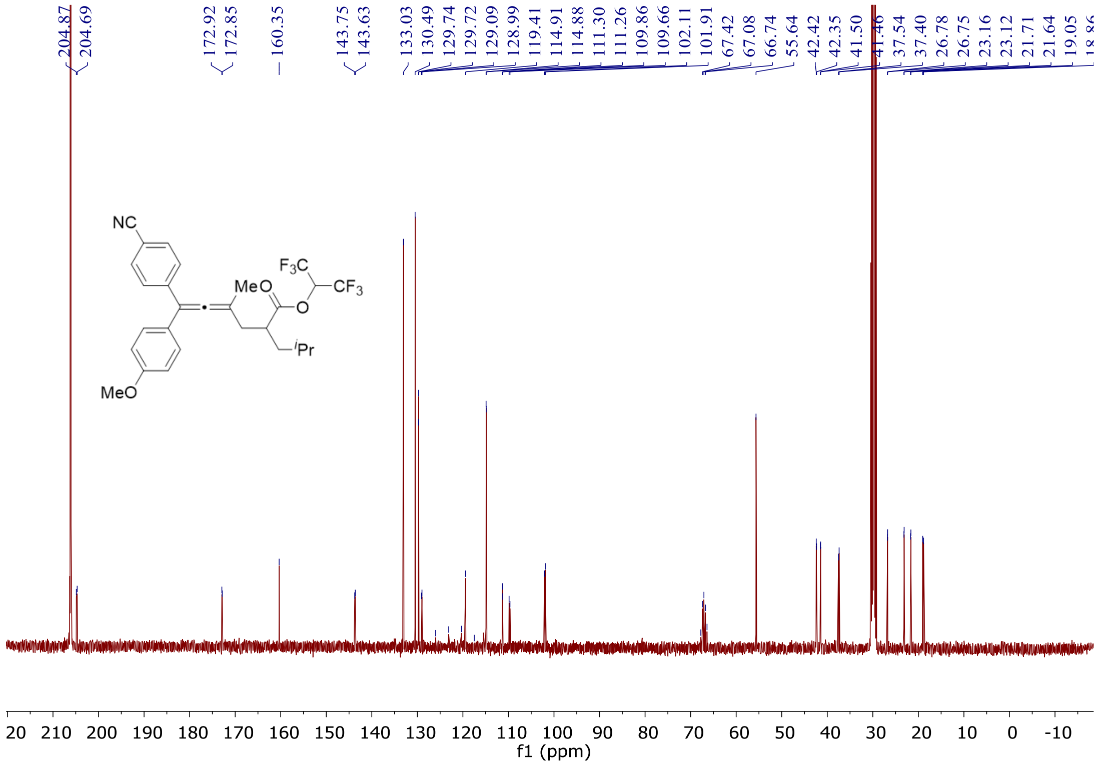

^{13}C{^{1}H} NMR (100 MHz, acetone-d_6) of **49**

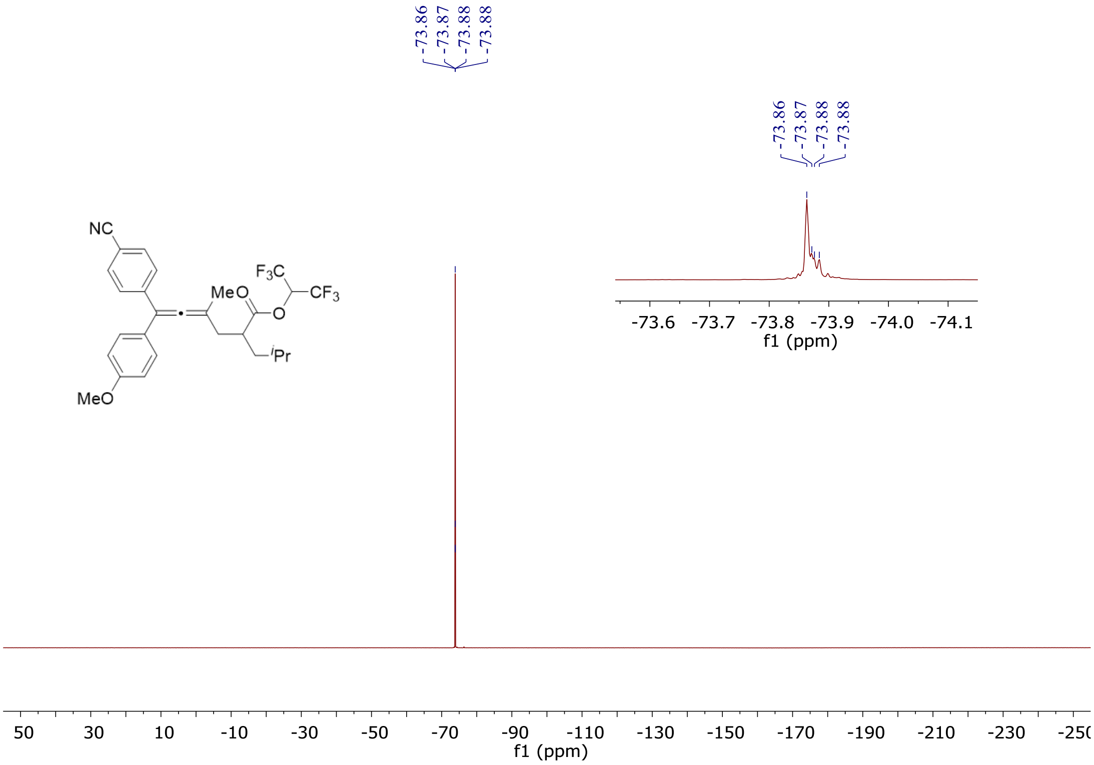

^{19}F{^{1}H} NMR (377 MHz, acetone-d_6) of **49**

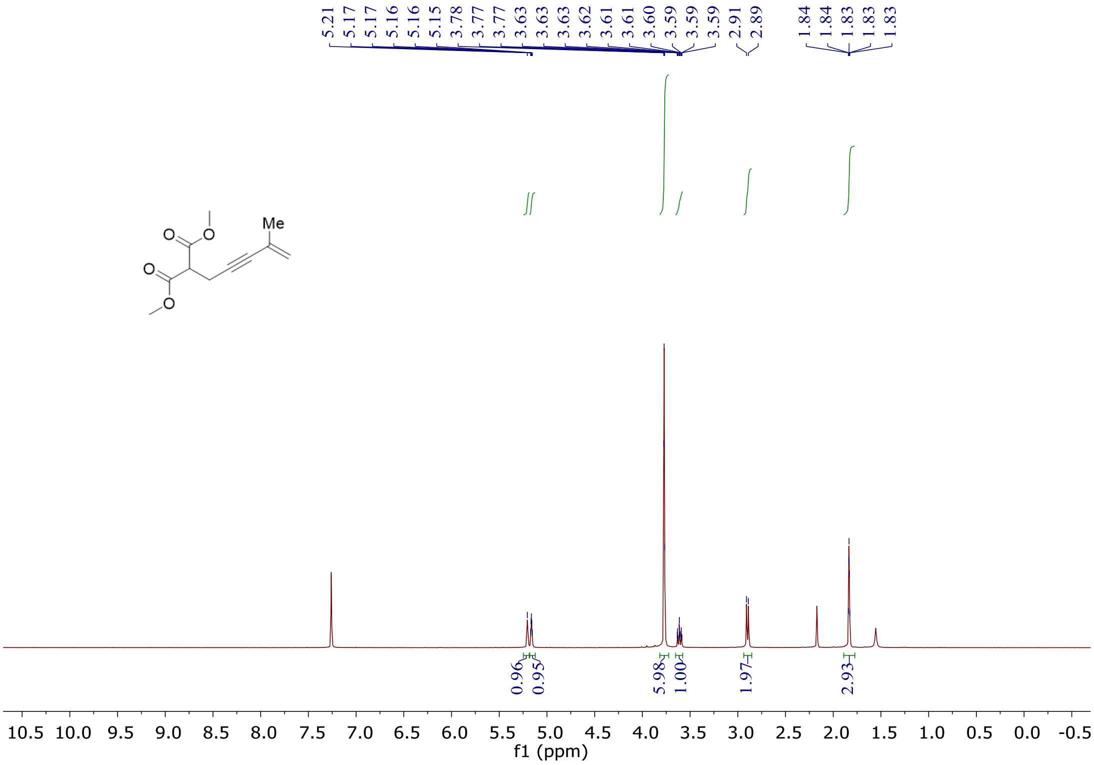

^{1}H NMR (400 MHz, $CDCl_3$) of **50**

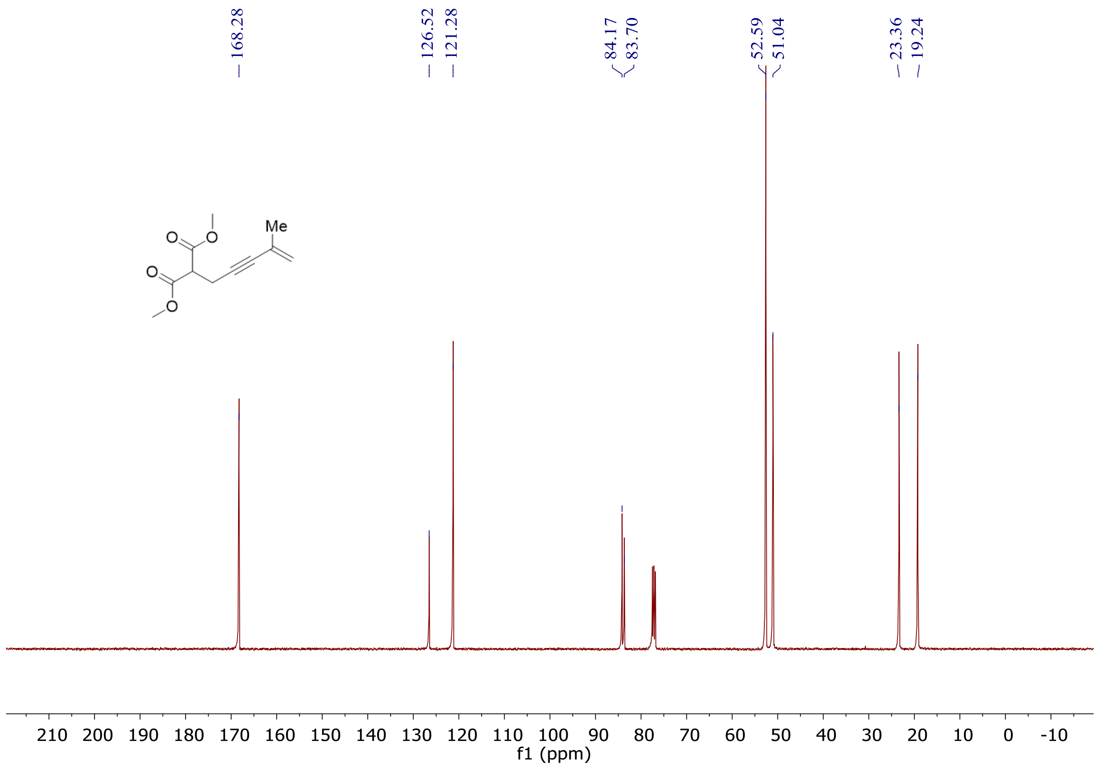

$^{13}C\{^1H\}$ **NMR** (100 MHz, $CDCl_3$) of **50**

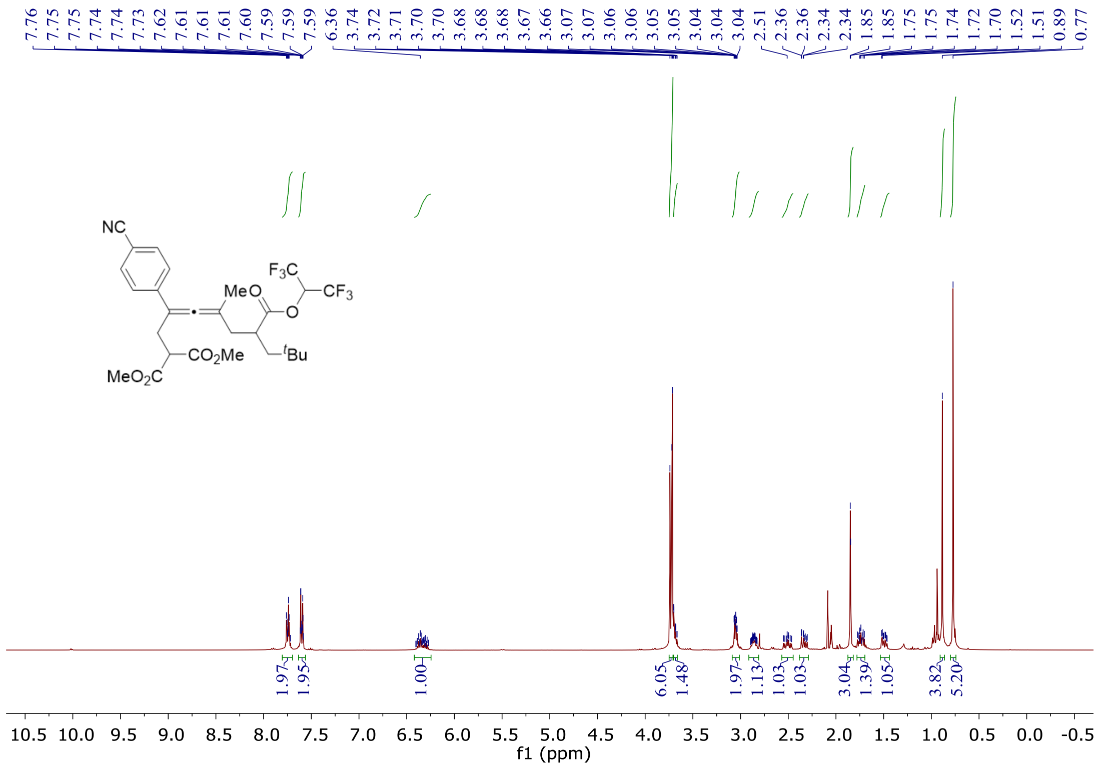

^1H **NMR** (400 MHz, acetone-d_6) of **51**

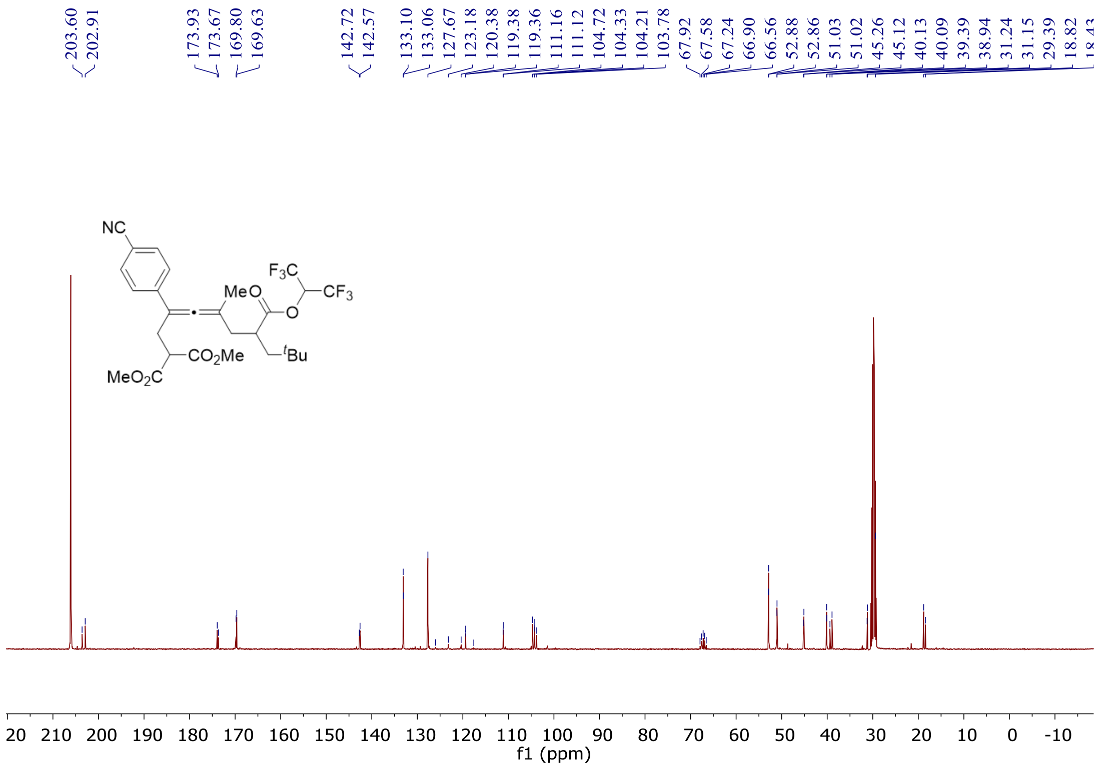

$^{13}C\{^1H\}$ **NMR** (100 MHz, acetone-d_6) of **51**

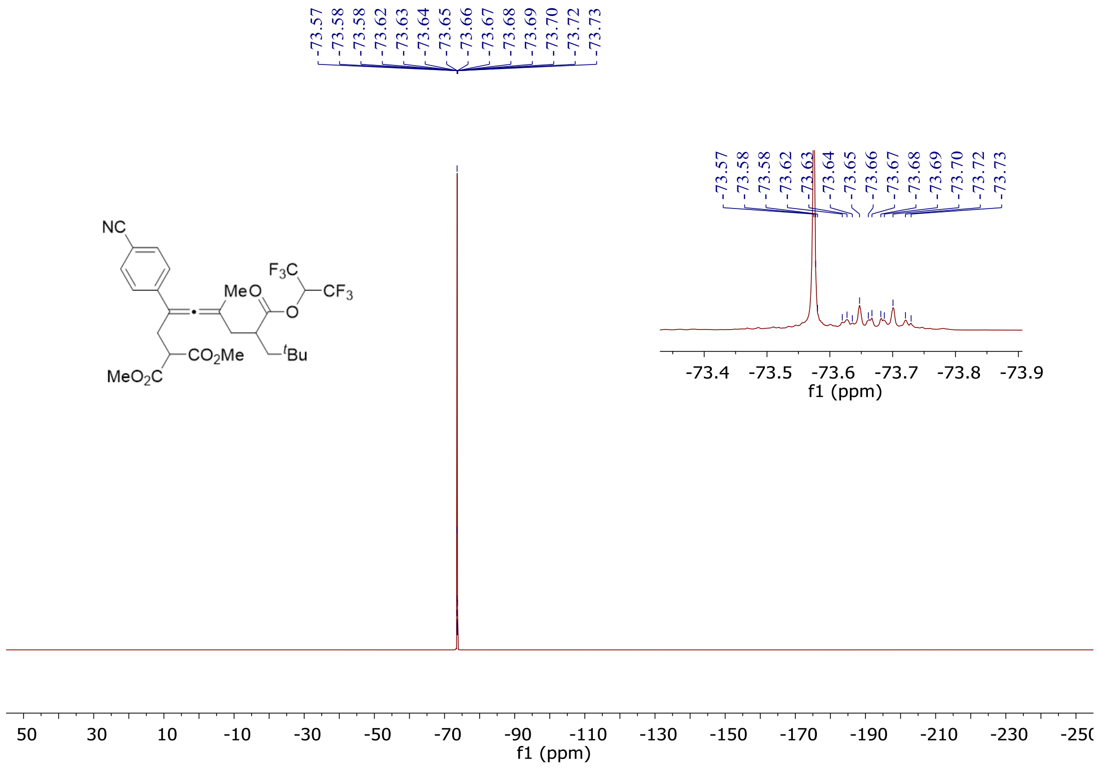

$^{19}F\{^1H\}$ **NMR** (377 MHz, acetone-d_6) of **51**

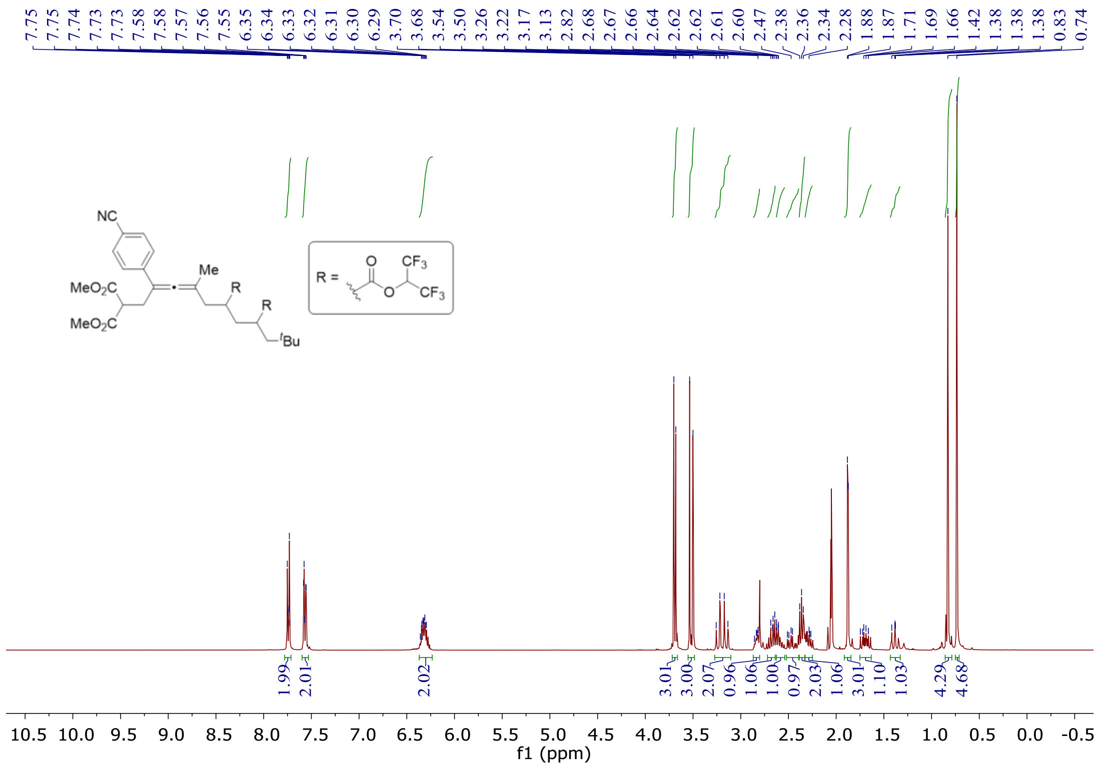

^{1}H NMR (400 MHz, acetone-d_6) of **52**

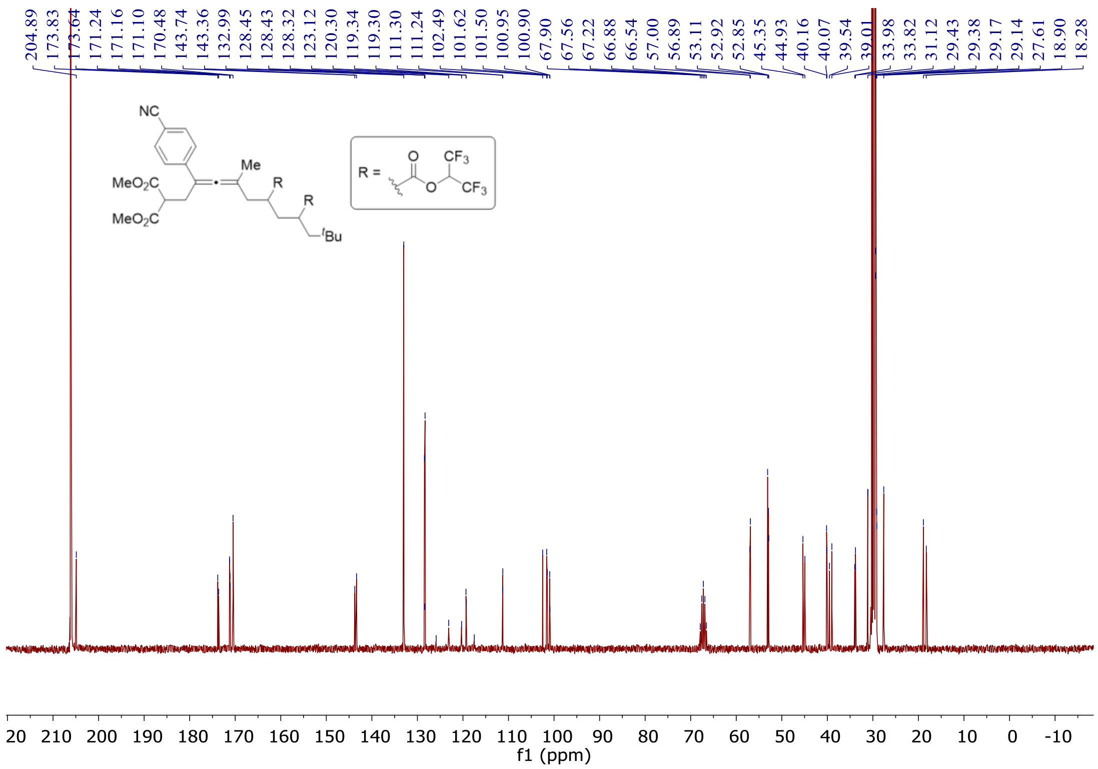

^{13}C{^{1}H} NMR (100 MHz, acetone-d_6) of **52**

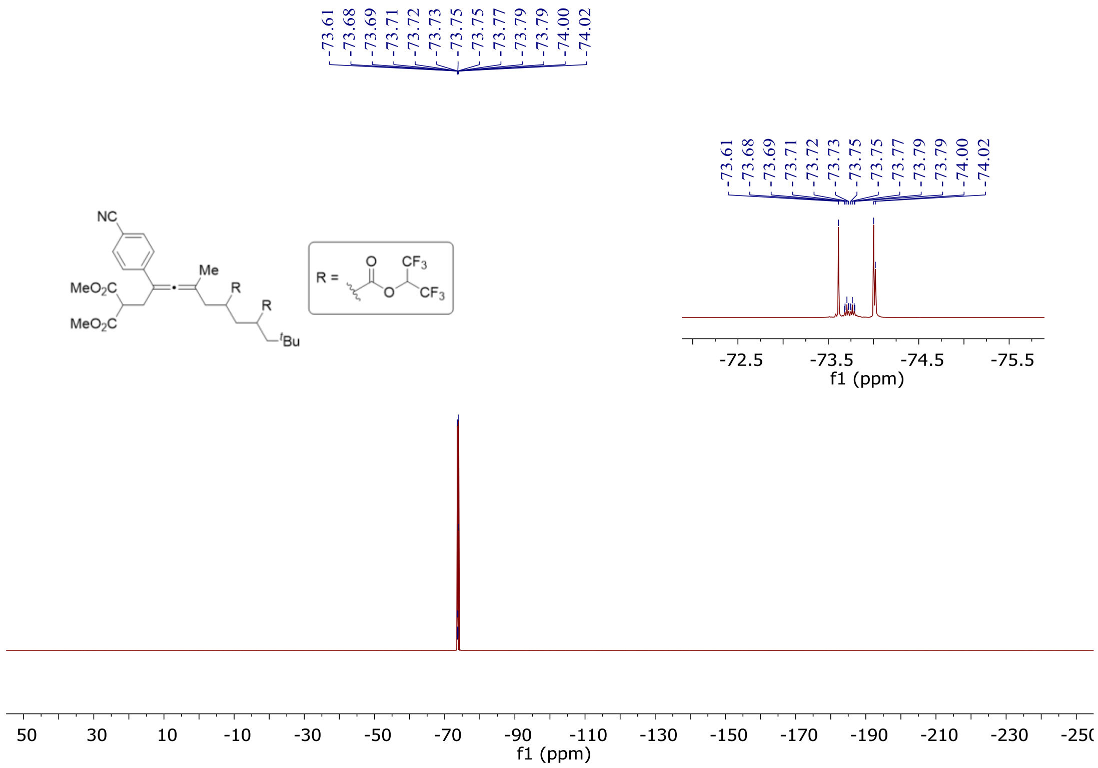

$^{19}F\{^{1}H\}$ NMR (377 MHz, acetone-d_6) of **52**
